# MEST promotes lung cancer invasion and metastasis by interacting with VCP to activate NF-κB signaling

**DOI:** 10.1186/s13046-021-02107-1

**Published:** 2021-09-24

**Authors:** Yang Wang, Jing Zhang, Yang-Jia Li, Nan-Nan Yu, Wan-Ting Liu, Jun-Ze Liang, Wen Wen Xu, Zheng-Hua Sun, Bin Li, Qing-Yu He

**Affiliations:** 1 MOE Key Laboratory of Tumor Molecular Biology and Key Laboratory of Functional Protein Research of Guangdong Higher Education Institutes, Institute of Life and Health Engineering, College of Life Science and Technology, Jinan University, Guangzhou, 510632 China; 2 MOE Key Laboratory of Tumor Molecular Biology and Guangdong Provincial Key Laboratory of Bioengineering Medicine, National Engineering Research Center of Genetic Medicine, Institute of Biomedicine, Jinan University, Guangzhou, 510632 China

**Keywords:** Lung cancer, MEST, VCP, NF-κB, Metastasis

## Abstract

**Background:**

Cell invasion is a hallmark of metastatic cancer, leading to unfavorable clinical outcomes. In this study, we established two highly invasive lung cancer cell models (A549-i8 and H1299-i8) and identified mesoderm-specific transcript (MEST) as a novel invasive regulator of lung cancer. We aim to characterize its biological function and clinical significance in lung cancer metastasis.

**Methods:**

Transwell invasion assay was performed to establish high-invasive lung cancer cell model. Immunohistochemistry (IHC) was used to detect MEST expression in tumor tissues. Mass spectrometry and bioinformatic analyses were used to identify MEST-regulated proteins and binding partners. Co-immunoprecipitation assay was performed to detect the interaction of MEST and VCP. The biological functions of MEST were investigated in vitro and in vivo. Immunofluorescence staining was conducted to explore the colocalization of MEST and VCP.

**Results:**

MEST overexpression promoted metastasis of lung cancer cells in vivo and in vitro by activating NF-κB signaling. MEST increased the interaction between VCP and IκBα, which accelerated IκBα degradation and NF-κB activation. Such acceleration was abrogated by VCP silencing, indicating that MEST is an upstream activator of the VCP/IκBα/NF-κB signaling pathway. Furthermore, high expressions of MEST and VCP were associated with poor survival of lung cancer patients.

**Conclusion:**

Collectively, these results demonstrate that MEST plays an important role in driving invasion and metastasis of lung cancer by interacting with VCP to coordinate the IκBα/NF-κB pathway. Targeting the MEST/VCP/IκBα/NF-κB signaling pathway may be a promising strategy to treat lung cancer.

**Supplementary Information:**

The online version contains supplementary material available at 10.1186/s13046-021-02107-1.

## Background

Lung cancer has become the leading killer among cancers, featuring high incidence and high rates of metastasis [1, 2]. Metastasis, the spread of cancer cells to distant organs, causes most human cancer-related deaths [3]. It occurs through a multistep process (including local invasion, intravasation, transport, extravasation, and colonization), which requires the concerted action of many genes and signal pathways [4, 5]. Currently, drug intervention that effectively targets cancer metastasis remains challenging, thus a better understanding of the molecular mechanisms involved in cancer metastasis is urgently needed. In view of the difficulty to investigate the molecular mechanisms of metastasis by using clinical samples, the establishment of highly invasive cancer cell lines enables us to mimic the acquisition of invasive phenotype [6]. In this study, we established two highly invasive lung cancer cell sublines by serial selections for invading through matrigel-coated invasion chamber and performed stable isotope labeling with amino acids in cell culture (SILAC)-based quantitative proteomics to identify the critical proteins that drive lung cancer invasion.

Among the upregulated proteins in the highly invasive cells, *MEST* (mesoderm-specific transcript)/*PEG1* (paternally expressed gene 1), a member of the α/β hydrolase superfamily located in the 7q32, drew our strong interest. *MEST* was reported as an imprinted gene that plays roles in the mesoderm development, which is only expressed from the paternally derived chromosome [7]. Loss of imprinting of *MEST* was frequently observed in invasive breast cancer [8] and lung cancer [9]. However, as its gene product, the role of MEST protein in lung cancer invasion and metastasis is unclear.

Mammalian NF-κB transcription factors consist of five homologous subunits: RelA/p65, c-Rel, RelB, p50/NF-κB1, and p52/NF-κB2. Among these, p65 and p50 are the most abundant. These NF-κBs are inactivated in the cytoplasm by inhibitors of NF-κBs (IκBs) [10]. IκB phosphorylation targets IκB for ubiquitination and then proteasome-mediated degradation. Valosin containing protein (VCP) is involved in degradation process of ubiquitinated IκBα, which increases degradation of cytoplasmic p-IκBα [11]. NF-κBs (such as p65) released from IκBs can translocate into the nucleus to regulate the transcription of a wide variety of genes [12–14]. The Ser-536 phosphorylation site in p65 is conserved in mammals and its phosphorylation has been reported to enhance p65 transcriptional activity [15]. Activation of NF-κB transcription factors is required for maintenance of the invasive phenotype in cancer [16], usually with involvement of matrix metalloproteinases (MMPs) [17], but the regulators that modulate this pathway are still unclear.

In this work, our immunohistochemical analysis of tumor tissue microarrays indicated that MEST is upregulated in lung cancer and its expression significantly correlates with lung cancer patient survival. A series of functional experiments showed that MEST promotes metastasis of lung cancer cells both in vitro and in vivo. Mechanistically, proteomics study suggested that NF-κB signaling is activated by MEST, and that VCP is the binding partner of MEST. Whether MEST exerts its oncogenic role through its binding to VCP and the subsequent activation of NF-κB signaling pathway was then investigated in detail. This study highlights the functional and clinical significance of MEST in lung cancer and the potential of MEST as a target for cancer therapeutics.

## Methods

### Cell lines and culture

**Fig. 1:**
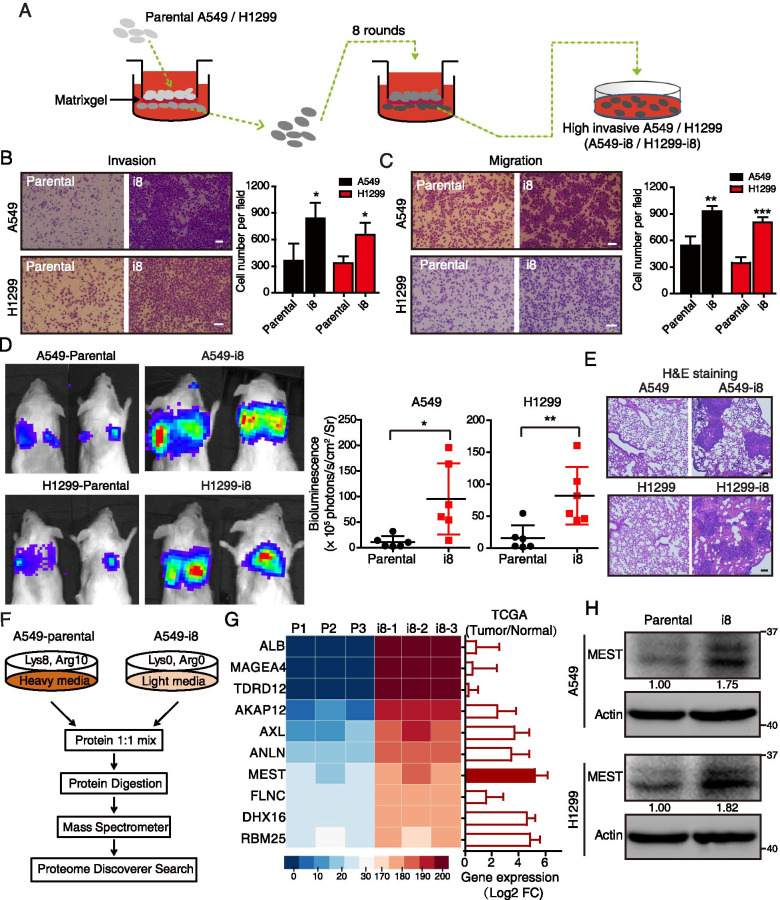
SILAC-based proteomics identifies MEST as an invasion regulator in lung cancer cells. **A** Diagram depicting the establishment of highly invasive cell lines (A549-i8 and H1299-i8) from lung cancer cells via eight rounds of invasion selection. **B**, **C** The abilities of invasion (**B**) and migration (**C**) of A549-i8 and H1299-i8 cell lines were determined by transwell assays, as compared to their parental cell lines, respectively. Scale bar, 100 μm. **D** NCG mice were transplanted with luciferase-labeled A549-i8 cells or H1299-i8 cells, or their corresponding luciferase-labeled parental cells (2 × 10^6^ cells per mouse) via tail vein injection (*n* = 6); mice were then visualized 1 month after transplantation by using an IVIS 200 Imaging System. Lungs harvested after imaging were histologically analyzed by H&E staining (**E**). Scale bar, 100 μm. **F** Scheme for the identification of differentially expressed proteins by SILAC-based quantitative proteomics. **G** The expression levels of the top 10 differentially expressed proteins in A549-i8 cells are represented by heatmap. Their expressions in lung adenocarcinoma and normal tissue were compared by using TCGA dataset (*n* = 503), showing that MEST is highly expressed in clinical lung cancer tissues, as indicated by red bars. **H** The expression of MEST in A549-i8 and H1299-i8 cells and in their parental cells was assessed by western blot analysis

Human lung cancer cell lines A549 and H1299 were obtained from the American Type Culture Collection (ATCC, Manassas, VA, USA). These cells were maintained in DMEM (Life Technologies, Beijing, China) with 10% fetal bovine serum (FBS, Life Technologies) in a humidified atmosphere of 5% CO_2_ at 37 °C. All cell lines were authenticated by short tandem repeat profiling. To establish highly invasive lung cancer cell lines, A549 or H1299 cells were seeded into the upper compartment (5 × 10^5^ cells/well) of an 8 μm pore-size matrigel invasion chamber (BD Biosciences, San Jose, CA, USA) and incubated for 10–16 h. The cells that invaded through the matrigel and adhered onto the lower surface of the chamber were detached with trypsin and expanded to produce enough cells for the next round of invasion selection. Expanded cells were inoculated into the upper compartment of a new invasion chamber [18]. This same procedure was repeated eight times to select highly invasive cells, designated as A549-i8 and H1299-i8 (Fig. 1A).

### Western blotting

Cell pellets were suspended in lysis buffer (Cell Signaling Technology, Danvers, MA, USA) and incubated on ice for 30 min. The cell lysates were subsequently centrifuged at 14,000 g for 30 min at 4 °C, after which supernatant was mixed with loading buffer and boiled for 5 min at 95 °C before being loaded onto a sodium dodecyl sulfate (SDS) polyacrylamide gel for electrophoresis. The proteins were subsequently transferred to polyvinylidene fluoride (PVDF) membranes (Bio-Rad, Hercules, CA, USA). After being blocked with 5% fat-free milk in Tris-buffered saline-Tween 20 (TBST), the membranes were probed with the appropriate primary antibodies, followed by corresponding horseradish peroxidase (HRP)-conjugated secondary antibodies (Cell Signaling Technology) and then the signals were detected by Clarity Western ECL Substrate (Bio-Rad).

Antibodies against the following proteins were used for the experiment: VCP (#10,736–1-AP), p65 (#10,745–1-AP), Lamin B1 (#66,095–1-Ig), MYC-tag (#60,003–2-Ig), Pan-Keratin (#26,411–1-AP) and ACTB (# 66,009–1-lg) were obtained from Proteintech Group (Chicago, IL, USA); IκBα (#4814), Phospho-IκBα (Ser32/36) (#9246), phospho-p65 (Ser536; #3033), IKKα (#11,930), Phospho-IKKα/β (Ser176/180; #2697), Phospho-Stat Antibody Sampler Kit (#9914) and MMP2 (#13,132) were obtained from Cell Signaling Technology; and MEST (#ab151564) and (orb247686) were obtained from Abcam (Cambridge, MA, USA) and Biorbyt, respectively. Anti-FLAG (F1804) was obtained from Sigma-Aldrich (St Louis, MO, USA). The bands of Western blotting were quantified by ImageJ software.

### Plasmids and agents

The pcDNA3.1-MEST-FLAG and pcDNA3.1-VCP-HA plasmids were generated by PCR using the human cDNA as template, cutting the PCR product by NheI and HindIII and ligating it into the pcDNA3.1. For the lentivirus plasmids construction, MEST-FLAG and IκBα-MYC was constructed into pLenti-CMV-Puro-DEST plasmids (addgene, #17,452). MEST-eGFP and VCP-mCherry were constructed using eGFP-N1 and mCherry-N1 plasmids. Correctness of the constructs was confirmed by inserting orientation check with restriction digestion, DNA sequencing and/or protein expression analysis. NF-κB inhibitor Bay11-7082 was purchased from Selleck Chemicals (Houston, TX) and the MMP-2 Inhibitor I was obtained from Cayman Chemical (Ann Arbor, Michigan, USA). Puromycin was obtained from TargetMol (Boston, MA, USA).

### SILAC labeling and LC–MS/MS analysis

To measure differences in protein expression between A549-i8 cells and parental A549 cells, a Stable isotope labeling in cell culture (SILAC)-based proteomics experiment was performed. To label A549 cells, cells were grown for 2 weeks in SILAC DMEM medium (Thermo Fisher Scientific, Waltham, MA, USA) containing “heavy” amino acids (Arg-8, Lys-10) (Cambridge Isotope Laboratories, Andover, MA) supplemented with dialyzed FBS (Thermo Fisher Scientific); A549-i8 cells were grown in ‘‘light medium” (Arg-0, Lys-0). The “heavy-labeled” and “light-labeled” cells were collected and mixed in equal amounts for digestion. In-gel digestion was performed for three biological replicate experiments, as previously described [19, 20]. Peptides were analyzed by using a Fusion Lumos mass spectrometer (Thermo Fisher Scientific), and MS parameters were set as previously described [21, 22]. Raw files were compared with a SwissProt database by using the Sequest HT engine of the Proteome Discoverer, version 2.1 (Thermo Fisher Scientific). Protein and peptide false discovery rates were set to 1%, and the abundance of ‘‘heavy” and ‘‘light” was automatically calculated by Proteome Discoverer program.

To profile the effect of MEST overexpression, another SILAC MS experiment was carried out in which either pcDNA3.1 plasmid or FLAG-tagged MEST-expression plasmid was transfected into A549 cells grown in “heavy media” (Lys-6) or “light media” (Lys-0) using Lip3000 (Life Technologies). Forty-eight hours after transfection, cells were collected and lysed separately (Fig. 3A). Lysates from “light-labeled” and “heavy-labeled” cells were mixed in equal amounts (by protein weight), and then in-gel digestion was performed. Peptides were analyzed by using an LTQ-Orbitrap mass spectrometer (Thermo Fisher Scientific) [23]. Protein identification and quantitation were performed by using MaxQuant software, version 1.2, to search the Uniprot-Swiss Human database, and normalized ratios of ‘‘heavy” versus ‘‘light” peptide abundance were automatically calculated by the MaxQuant program.

The mass spectrometry and proteomics data have been deposited to the ProteomeXchange Consortium (http://proteomecentral.proteomexchange.org) via the iProX partner repository (dataset identifier: IPX0003001000; ProteomeXchange number: PXD026409).

### Co-immunoprecipitation coupled with mass spectrometry analysis

The indicated plasmids were respectively transfected into cancer cell lines using Lip3000 (Life Technologies). After 48 h transfection, cells were harvested and lysed in IP lysate buffer (containing 1% NP-40, Beyotime, Jiangsu, China), and the lysates were cleaned by centrifugation at 4 °C. The extracts were incubated with anti-Flag antibody and protein A/G resin (Santa Cruz, CA, USA) at 4 °C for 1 h with slow rotation. Unbound proteins were washed away with IP buffer five times, and bead-bound proteins were subjected to SDS-PAGE separation and MS analysis (LTQ-Orbitrap) for protein identification.

### siRNA interference and transfection

For siRNA (GenePharma Corporation, Shanghai, China) interference, si-RNAs were transfected into cancer cells (in 6-well dishes) using Lipofectamine 3000 (Life Technologies). After 48 h, cells were harvested and lysed in lysis buffer. The human MEST specific siRNAs (#1 and #2), human VCP specific siRNAs (#1 and #2) and control siRNAs were purchased from GenePharma (Shanghai, China) with following primers. si-MEST#1: 5’-GAGUAGCUUCCCUGUAUUATT-3’; si-MEST#2: 5’-GGAUCAACCUUCUUUCUCATT-3’; si-VCP#1: 5’-GUGAGUCUGAGAGCAACCUUCGUAA-3’; si-VCP#2: 5’- GUAAUCUCUUCGAGGUAUA-3’; Negative Control: 5'-UUCUCCGAACGUGUCACGUTT-3’.

### In vitro cell migration, invasion, and cell viability assays

In vitro cell migration assays were performed by using uncoated, 8 μm pore-size Transwell chambers as described previously (BD Biosciences) [24]. Cells in serum-free medium were seeded into the upper chamber. Complete medium was added to the bottom wells of the chamber. After 24 h, the cells that had not migrated were removed from the upper surface of the filters by using cotton swabs. Cells that had migrated into the lower surface were fixed with methanol and stained with crystal violet (Sigma-Aldrich). Images of five different fields were captured using microscope (Olympus, Tokyo, Japan) from each membrane, and the numbers of migrated cells were counted. The mean of values from triplicate assays for each experimental condition was calculated. A similar protocol was used to measure invasive potential with the use of BD Bio Coat Matrigel Invasion Chambers (BD Biosciences). WST-1 assay (Beyotime) was used to measure cell viability, as previously described [25].

### Human lung cancer tissue arrays and immunohistochemical staining

Two human lung cancer tissue arrays (one containing 90 cases of human lung cancer tissues; the other, 87 cases of human lung cancer tissues) with survival data, and two tissue arrays (containing 30 pairs of primary lung cancer tissues and matched metastatic tissues) (Shanghai Outdo Biotech. Co. Ltd, Shanghai, China) were incubated with antibody against either MEST or VCP, and with biotin-conjugated secondary antibody. Assays were then incubated with avidin–biotin-peroxidase complex and visualization was performed with 3,3’-diaminobenzidine tetrahydrochloride. MEST and VCP immunoreactivities were semiquantitatively scored by using a well-established immunoreactivity score system, in which the immunoreactivity score was generated by incorporating both the percentage of positive tumor cells and the intensity of staining [26].

### Luciferase assay

To determine the effect of MEST on MMP2 promoter, A549 and H1299 cells were transfected with indicated plasmid or siRNA together with the pGL3-based construct containing MMP2 promoter plus Renilla luciferase plasmid. After 24 h, the reporter activity was measured by using a luciferase assay kit (Promega, WI, USA) and plotted after normalizing with respect to Renilla luciferase activity (mean ± SD).

### Zymography

To assess MMP2 activity, the collected supernatant was concentrated 40-fold using an Amicon Centricon (Millipore Co., Bedford, MA, USA), and electrophoretically separated onto 8% SDS–polyacrylamide gel containing 1 mg/mL of gelatin. After electrophoresis, the gel was stained with 0.2% Coomassie brilliant blue R-250 in a mixture of methanol: acetic acid: water (2:1:7) for 2 h, and then destained in a destaining solution. Clear zones against the blue background indicated the presence of gelatinolytic activity.

### Experimental in vivo metastasis model

Female BALB/c nude mice and NCG mice (aged 6–8 weeks) (Model Animal Research Center of Nanjing University, Nanjing, Jiangsu, China) were bred and maintained under standard conditions at the Animal Experiment Center of the College of Medicine (SPF grade), Jinan University. In vivo metastatic assays were performed as previously described with minor modifications [27, 28]. In brief, six mice in each experimental group were injected with luciferase-expressing A549 cells with indicated treatment (2 × 10^6^ cells per 100 μL of PBS) into tail veins. Metastatic foci in lungs were visualized using an IVIS 200 Imaging System (Xenogen) 4–8 weeks after injection. Animal experiments were approved by the Laboratory Animal Ethics Committee of Jinan University, and conformed to the legal mandates and national guidelines for the care and maintenance of laboratory animals.

### Bioinformatics analysis

Differential expression proteins or potential MEST partners were analyzed by Ingenuity Pathway Analysis (IPA; https://www.ingenuity.com, QIAGEN, Shanghai, China). A core analysis was performed as we described previously [29]. The values from SILAC-proteomics comparing A549-i8 and parental A549 were subjected to principal components analysis (PCA), which were computed using factoextra package of R software (4.0.5) [30].

### Analysis of gene expression and survival data from cancer patient datasets

The expression of top 10 high-invasive relevant proteins was subjected to Ordino online software (https://ordino.caleydoapp.org/) [31]for analysis of their expressions in 503 cases of paired lung adenocarcinoma tissue samples derived from TCGA database. MEST expression in lung cancer patient microarray datasets were analyzed by Oncomine (https://www.oncomine.org/resource/login.html). The datasets with survival data from patients with lung cancer were downloaded from the GEO database (accession numbers GSE14814, GSE8894, GSE13213, GSE31210). Gene expressions were further divided into high and low levels using median expression level as the cutoff point for Kaplan–Meier survival analyses.

### Statistical analysis

To identify differentially expressed proteins in A549-i8 cells and parental A549 cells, fold changes and *P* values were calculated by using R software (4.0.5), and *P* values were adjusted by using Benjamini–Hochberg false discovery rate approach. MATLAB 2016a software was used to perform clustering analysis. The data were expressed as the mean (standard deviation) and compared by using ANOVA. The tumor expression levels of MEST and VCP in tumor were compared with those in non-tumor tissues by using either paired or unpaired *t*-tests. The correlation between MEST expression levels and clinicopathological parameters was assessed by using Fisher exact test. Survival analysis was performed by using the Kaplan–Meier method with the log-rank test. A minimum of three observations per group was conducted. All the data were analyzed by two-tailed unpaired Student’s *t*-test using GraphPad Prism software, version 5.00 (San Diego, CA, USA). Significance was determined by an alpha level of 0.05.

## Results

### Establishment of a cell model for highly invasive lung cancer and identification of invasion-associated proteins

To mimic the progression of lung cancer invasion, we performed eight rounds of selection for invading A549 cells and H1299 cells by using the matrigel chamber invasion assay (Fig. 1A). This selection produced the A549-i8 and H1299-i8 cell lines that had significantly higher invasion and migration potential than the parental cell lines (Fig. 1B–C). Moreover, our in vivo assay showed that these highly invasive cell lines were dramatically more metastatic than the parental groups (Fig. 1D), which we confirmed by hematoxylin and eosin (H&E) and pan-keratin staining of metastatic lung nodules (Fig. 1E; Figure S1A). These data indicated that the invasion-selected cancer cell lines were more motile and an appropriate model for subsequent experiments.

To compare protein expression between A549-i8 cells (labeled with “light” chain amino acids) and parental A549 cells (labeled with “heavy” chain amino acids), a SILAC-based proteomics approach was performed (Fig. 1F). A total of 5166 proteins were identified, in which 2424 proteins were overlapped in three independent experiments (Figure S1B). Principal component analysis (PCA) of differentially expressed proteins (DEPs; *P* < 0.05, fold change (FC) ≥ 1.5; Supplementary Table S1) demonstrated a clear distinction between the A549-i8 and A549-parental samples, suggesting the protein expression pattern in highly invasive cancer cells is different from parental cells (Figure S1C). Correlation analysis revealed a certain reproducibility (Pearson’s *r* = 0.79–0.84; *P* < 0.0001) between independent biological replicates (Figure S1D). Ingenuity Pathway Analysis (IPA) showed that the molecular and cellular functions of DEPs were related to RNA post-transcriptional modification, cellular development, cellular growth and proliferation, and cell movement (Figure S1E).

To screen for novel regulators of cancer invasion, we measured expression levels of the ten most upregulated proteins (*P* < 0.01) in 503 cases of paired lung cancer tissue samples available in the TCGA database. MEST, DHX16 and RNPC7 were not only upregulated in highly invasive A549-i8 cells, but also highly expressed in lung cancer (Fig. 1G). We then applied Kaplan Meier-Plotter to evaluate these proteins, and found that only high expression of MEST was significantly relevant to poor prognosis of lung cancer patients (Figure S1F). Further, the up-regulation of MEST in A549-i8 and H1299-i8 cells was also verified by western blot analysis (Fig. 1H). These data suggest that MEST may be involved in lung cancer metastasis with significant clinical relevance.

### MEST increases lung cancer metastasis

**Fig. 2:**
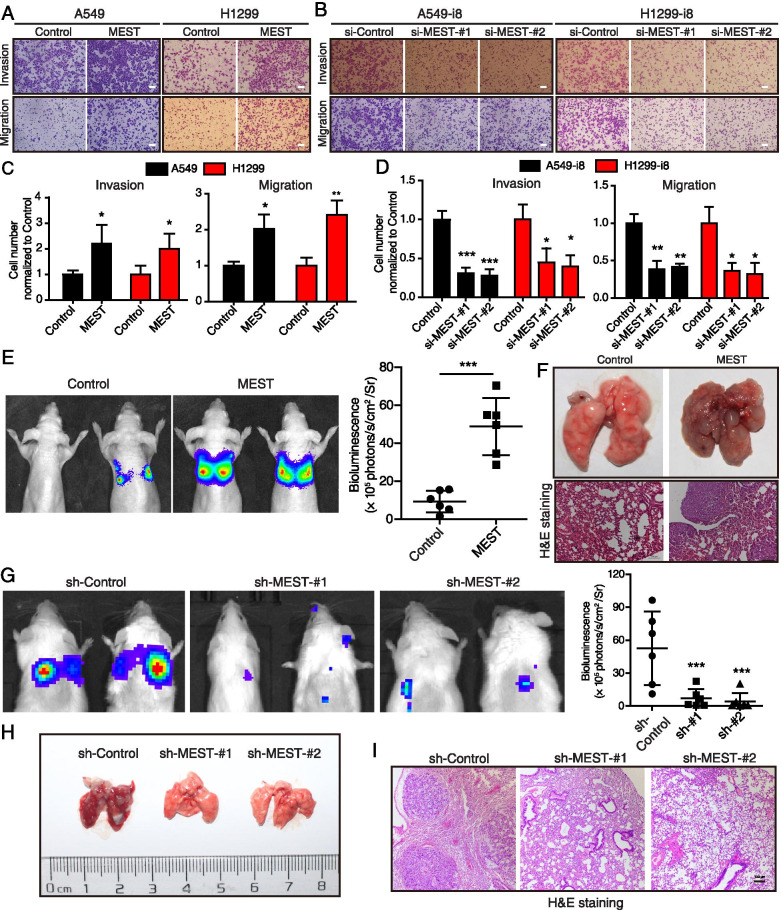
MEST promotes invasion and migration of lung cancer cells. **A**, **B** A549 and H1299 cells were overexpressed with MEST, while A549-i8 and H1299-i8 cells were transfected with two siRNAs against MEST (si-MEST#1 and si-MEST#2, 100 nM), their invasion and migration abilities were measured by transwell assay. Scale bar, 100 μm. All data are representative of three independent experiments (**C**, **D**). Bars, S.D. *P < 0.05, **P < 0.01, ***P < 0.001 (Student’s *t*-test). **E–F** Nude mice were transplanted with luciferase-labeled cells with or without MEST overexpression (2 × 10^6^ cells per mouse) via tail vein injection (*n* = 6); mice were then visualized 1 month after transplantation by using an IVIS 200 Imaging System. Lungs harvested after imaging are shown. Note that only MEST-overexpressing A549 cells formed large and more metastatic nodules in the lungs. The pulmonary metastases in the mouse model were histologically analyzed by H&E staining (**F**). Scale bar, 100 μm. **G-I** NCG mice were transplanted with the luciferase-labeled A549-i8 cells expressing two shRNA against MEST (1 × 10^6^ cells per mouse) via tail vein injection (*n* = 6); the mice were visualized 1.5 months post-transplantation by using an IVIS 200 Imaging System (**G**). Lungs harvested after imaging are shown (**H**). Note that only the control group cells formed large and more metastatic nodules in the lungs. The pulmonary metastases in the mouse model were histologically analyzed by H&E staining (**I**). Scale bar, 100 μm

To examine the biological function of MEST in cancer, we established MEST-overexpressing stable cell lines (from A549 and H1299 cells) and MEST-knockdown cell lines (from A549-i8 and H1299-i8 cells) (Figure S2A–B). Using a transwell assay, we found that MEST overexpression significantly enhanced invasion and migration of both A549 and H1299 cells, whereas MEST knockdown inhibited invasion and migration of highly invasive A549-i8 and H1299-i8 cells (Fig. 2A–D) without influencing cell viability (Figure S2C). Using an in vivo assay, we observed that MEST-overexpressing A549 cells metastasized to the lung more than control cells; We saw significantly higher bioluminescent imaging signal and more lung metastatic nodules in mice transplanted with MEST-overexpressing cells than in those transplanted with control cells (Fig. 2E). The metastatic lung nodules were confirmed by H&E staining and positive pan-keratin staining (Fig. 2F; Figure S2D-F), respectively, and the expression of MEST was confirmed by immunohistochemistry (IHC) staining (Figure S2D). Moreover, MEST-knockdown A549-i8 cells were less able to metastasize to the lung (rates of metastases decreased in the two groups of MEST-knockdown A549-i8 cells to 14.9% and 9.7%) (Fig. 2G). More metastatic nodules were formed in the lungs of mice injected with A549-i8 cells than in those injected with MEST-knockdown cells (Fig. 2H), as confirmed by H&E and pan-keratin staining (Fig. 2I; Figure S2G-I), and MEST IHC staining (Figure S2G). Collectively, these results illustrate that MEST may play a functional role in regulating cancer metastasis.

### MEST promotes the activation of NF-κB pathway

**Fig. 3:**
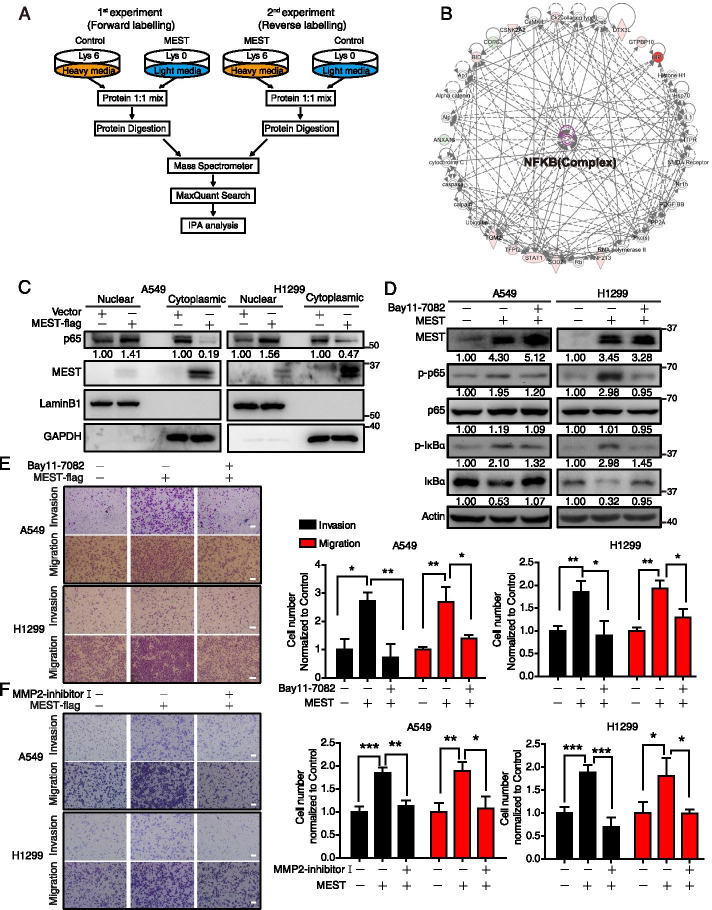
SILAC quantitative proteomics characterizes MEST-regulated NF-κB pathway in lung cancer. **A** The scheme of two independent SILAC experiments, including forward labeling and reverse labeling. MEST-regulated proteins were identified by two independent SILAC experiments, and the differentially expressed proteins were analyzed by Ingenuity Pathway Analysis (**B**). MEST-driven functional signaling networks indicate involvement in the NF-κB signaling pathway. Red, upregulated proteins; green, downregulated proteins; white, speculative proteins. **C** MEST overexpression promotes nuclear translocation of NF-κB subunits (p65) in both A549 and H1299 cells. LaminB1 and GAPDH were used as markers for nucleus and cytoplasma, respectively. **D** A549 and H1299 overexpressing MEST were treated with either 2.5 μM of Bay11-7082 or DMSO, as indicated. Expression of NF-κB markers (including p-p65, p65, p-IκBα, and IκBα) was assessed by western blot analysis. Invasion and migration of treated cells were measured by transwell assay (**E**). **F** A549 and H1299 cells overexpressing MEST were treated with either 25 μM of MMP2 inhibitor I or DMSO; their invasion and migration abilities were determined by transwell assay. Scale bar, 100 μm. All data are representative of three independent experiments. Bars, S.D. *P < 0.05, **P < 0.01, ***P < 0.001 (Student’s *t*-test)

We used a SILAC-based proteomics approach to identify MEST-regulated proteins. From two independent label-swap experiments (forward and reverse; Fig. 3A), we identified 127 proteins that were regulated by MEST overexpression (FC ≥ 1.3; Supplementary Table S2). IPA analysis suggested that the MEST-regulated proteins were involved in the NF-κB pathway (Fig. 3B). By western blot analysis, we validated that MEST expression upregulated both p-p65 (Figure S3A) and nuclear translocation of p65 (Fig. 3C) in both A549 and H1299 cell lines. Then, we used Bay11-7082, a NF-κB inhibitor (Figure S3B–C), to investigate whether NF-κB pathway functionally mediates the pro-metastasis effect of MEST. Bay11-7082 treatment significantly attenuated the expressions of p-p65 and p-IκBα that were enhanced by MEST (Fig. 3D). Moreover, the cell invasion and migration ability driven by MEST can be blocked by the NF-κB inhibitor (Fig. 3E). Matrix metalloproteinase 2 (MMP-2), responsible for the degradation of the extracellular matrix, can be activated by NF-κB signaling [32]. We observed that MEST increased MMP2 activity in both A549 and H1299 cells, as evidenced by zymography assay (Figure S3D). Conversely, the introduction of MMP2 inhibitor I (Figure S3E), was able to block the invasion and migration ability driven by MEST (Fig. 3F). These results indicate that MEST promotes cell invasion and migration via the NF-κB/MMP2 signaling pathway.

### MEST interacts with VCP to regulate the degradation of IκBα protein

**Fig. 4:**
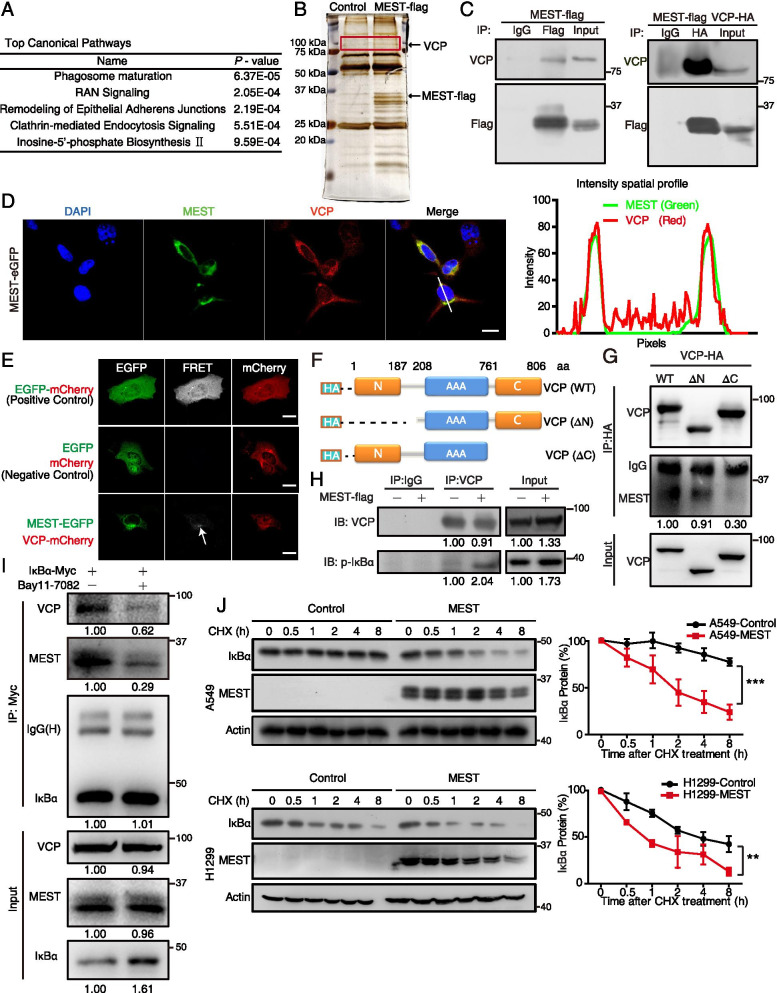
MEST interacts with VCP and accelerates IκBα degradation. **A** H1299 cells were transfected with MEST-FLAG expression plasmids, and MEST-binding proteins were identified by co-immunoprecipitation coupled with LC/MS analyses. A list of candidate proteins was uploaded for Ingenuity Pathway Analysis, which revealed that the top canonical pathways involved membrane trafficking. **B** Silver staining proteins coimmunoprecipitated with MEST show a specific 97 kDa band corresponding to VCP. **C** H1299 cells were transfected with MEST-FLAG and VCP-HA expression plasmids. The interaction between MEST and VCP was demonstrated by co-immunoprecipitation assays. **D** A549 cells transfected with MEST-eGFP (green) were stained with VCP antibody (red) for 48 h, and co-localization of MEST and VCP in the perinuclear area was imaged by using a confocal microscope; intensity spatial profiles are plotted. Scale bar, 20 μm. **E** Fluorescence resonance energy transfer experiment showed the energy transfers from MEST-eGFP (donor) to VCP-mCherry (acceptor). Expression of an eGFP-mCherry fusion protein is a positive control, and co-expression of eGFP-N1 and mCherry-N1 is a negative control. Excitation wavelength is 488 nm, and collection wavelength is 600 nm. Scale bar, 20 μm. **F** Schematics for the structural domains of VCP and for the N-terminal deletion (ΔN) and C-terminal deletion (ΔC) mutants. The blue line represents the AAA domain of VCP. **G** The interactions of VCP-truncation mutants (WT, ΔN, and ΔC) with MEST were detected by co-immunoprecipitation assay. **H** MEST increases the binding of VCP to p-IκBα. H1299 cells were transfected either with or without MEST-FLAG expression plasmids for 48 h; cell lysates were incubated with anti-VCP antibody, and expression of p-IκBα was assessed by western blot analysis. **I** Bay11-7082 abolishes interactions between MEST, VCP, and IκBα. A549 cells expressing Myc-tagged IκBα were treated with Bay11-7082 (2.5 μM) for 12 h. Cell lysates were incubated with anti-Myc antibody for co-immunoprecipitation assay, and VCP and MEST were detected by western blot analysis. **J** MEST-overexpressing H1299 cells, MEST-overexpressing A549 cells, and their respective vector control cells were treated with cycloheximide (CHX; 50 μg/mL). The cell lysates were collected at the indicated time points and compared for IκBα expression by western blot analysis. IκBα signals are quantified by densitometry, and the degradation rate is shown as the ratio of IκBα expression level at each time point to the original expression level (0 h). The half-life (t_1/2_) of IκBα was 0.9 and 3.6 h in MEST-overexpressing H1299 cells and corresponding vector control cells, respectively; t_1/2_ values were 1.8 and 28.6 h in MEST-overexpressing A549 cells and vector control cells, respectively

To investigate the molecular mechanisms for how MEST regulates NF-κB signaling, we identified binding partners of MEST by performing immunoprecipitation coupled with mass spectrometry analysis (IP-MS). In two independent IP-MS experiments, we identified 32 candidate proteins (Supplementary Table S3). IPA analysis showed that these proteins were mainly involved in phagosome maturation and membrane trafficking (Fig. 4A). Thus, we speculated that MEST exerts its function by influencing the intracellular membrane system. We detected the subcellular localization of MEST and found that it was located in Golgi-ER rather than in lysosomes (Figure S4A).

Among the 32 candidates, VCP (a specific band at ~ 100 kDa; Fig. 4B), was particularly interesting because it not only localizes to the Golgi-ER but also regulates both ER stress and the NF-κB signaling pathway [11, 33, 34]. The interaction between MEST and VCP was then confirmed by both immunoprecipitation (Fig. 4C) and immunofluorescence staining analysis, showing that MEST co-localized with VCP in the perinuclear area of lung cancer cells (Fig. 4D). Moreover, we detected fluorescence resonance energy transfer from MEST-EGFP (green) to VCP-mCherry (red) but not from EGFP to mCherry (Fig. 4E). To map the interacting regions of MEST and VCP in detail, we generated two truncation mutants of VCP (Fig. 4F), including an N-terminal deletion mutant (ΔN, with a deletion of amino acids 1–187) and a C-terminal deletion mutant (ΔC, with a deletion of amino acids 761–806). By performing IP assay in lung cancer cells, we found that MEST bound to the C-terminal domain of VCP (Fig. 4G), suggesting that MEST may be a cofactor by binding to the C-terminal domain of VCP.

Since VCP is a molecular chaperon that facilitates degradation of the phosphorylated form of IκBα [33], we speculated that MEST may increase the ability of VCP to capture and to accelerate its substrate p-IκBα degradation. To test this hypothesis, we performed co-IP experiments in H1299 cells with or without MEST overexpression, and found that the binding between VCP and p-IκBα was increased by MEST overexpression (Fig. 4H). Moreover, we found that treatment of Bay11-7082 could significantly abolish the interaction of MEST-VCP-IκBα (Fig. 4I), suggesting that VCP regulated by MEST has higher affinity to p-IκBα, rendering the destabilization of IκBα. To confirm this observation, both MEST-overexpressed cells and control cells were treated with cycloheximide (CHX) to study the effect of MEST expression on IκBα stability. The results showed that IκBα protein was rapidly decreased in MEST-overexpressing cells, as compared with control cells (Fig. 4J). Moreover, we found that MEST could enhance the ubiquitination of IκBα (Figure S4B). These results suggest that IκBα became more instable in MEST-overexpressing cells.

### VCP is essential for MEST-induced activation of NF-κB pathway and cancer invasion

**Fig. 5:**
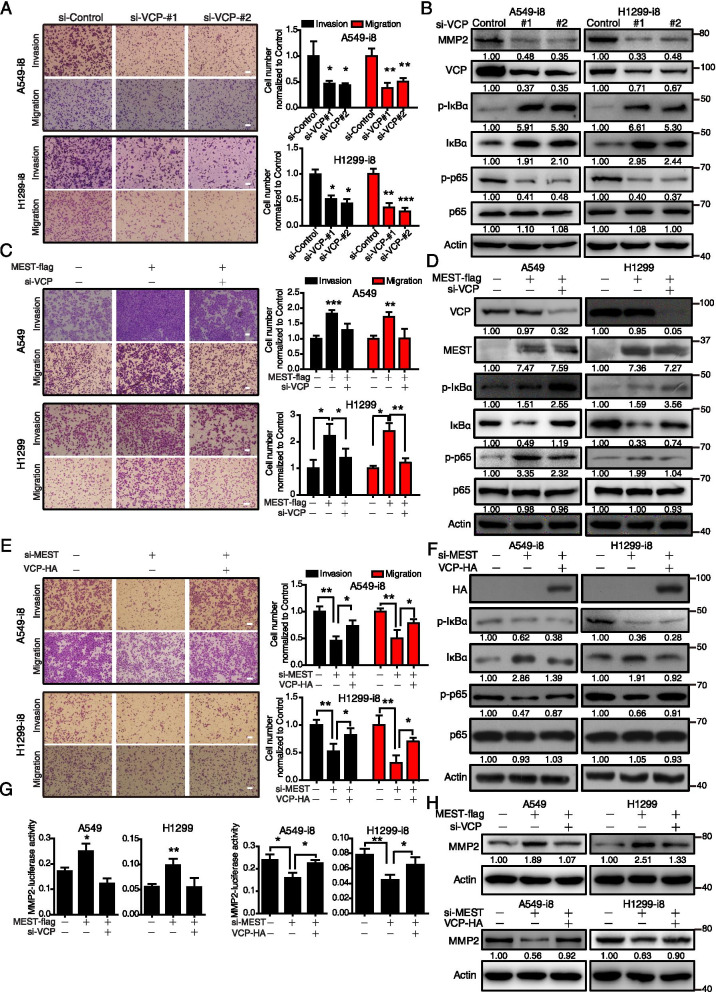
MEST and VCP promote lung cancer migration and invasion via activating the NF-κB pathway. **A** A549-i8 and H1299-i8 cells were transfected with two anti-VCP siRNAs, and the migration and invasion abilities were determined by using a transwell assay; MMP2 and NF-κB markers (p-IκBα, IκBα, p-p65, and p65) were analyzed by western blot analysis (**B**). **C** MEST-overexpressing A549 and H1299 cells were transfected with si-VCP and scramble si-RNA for 24 h. Transwell assay indicates that knockdown of VCP inhibits cell migration and invasion enhanced by MEST in A549 and H1299 cells. Expression of NF-κB markers (p-IκBα, IκBα, p-p65, and p65) were analyzed by western blot analysis (**D**). **E** A549-i8 and H1299-i8 were transfected with VCP-HA plasmid or si-MEST for 24 h, as indicated. Transwell assay shows that knockdown of MEST in A549-i8 and H1299-i8 cells decreases cell migration and invasion, and that transfection with VCP-expression plasmid partially restores this effect. The corresponding NF-κB markers (p-IκBα, IκBα, p-p65, and p65) were analyzed by western blot analysis (**F**). **G** The indicated cell lines were co-transfected with MMP2 promoter-driven luciferase reporter, with pRL-TK (loading control), as well as with either the indicated plasmids or siRNA. Luciferase activity was measured (*n* = 3). **H** Western blot analysis indicates that MMP2 expression is regulated by MEST and VCP. Bars, SD. *, *P* < 0.05; **, *P* < 0.01; ***, *P* < 0.001 compared with control cells unless otherwise indicated. Scale bar, 100 μm

Previous reports showed that VCP contributes to cancer progression by regulating NF-κB signaling [11]. In this study, we found that VCP knockdown suppressed cell migration and invasion as well as NF-κB signaling in A549-i8 and H1299-i8, as evidenced by the decrease of p-p65 and MMP2 and the accumulation of p-IκBα and IκBα (Fig. 5A-B). We then tested whether VCP is required for MEST-driven cell invasion and migration by using VCP-targeting siRNA. The results showed that inhibition of VCP blocked MEST-induced cell invasion and migration (Fig. 5C) as well as NF-κB activation, as indicated by a decrease of p-p65 expression. Note that VCP inhibition blocked the degradation of p-IκBα and IκBα, showing significant accumulation of p-IκBα and IκBα in siRNA-treated cells (Fig. 5D). Conversely, MEST knockdown inhibited cell invasion and migration, and decreased p-p65 expression, while these could be restored by overexpression of VCP (Fig. 5E-F). These results suggest that MEST acts as an upstream regulator of VCP that influences NF-κB signaling by sustaining IκBα in phosphorylation form. Moreover, luciferase reporter assay and immunoblotting assays revealed that MEST increases MMP2 expression at both mRNA and protein levels, which could be abolished by knockdown of VCP (Fig. 5G-H). Note that VCP overexpression could significantly restore the inhibitory effect of MEST knockdown on MMP2 expression.

**Fig. 6:**
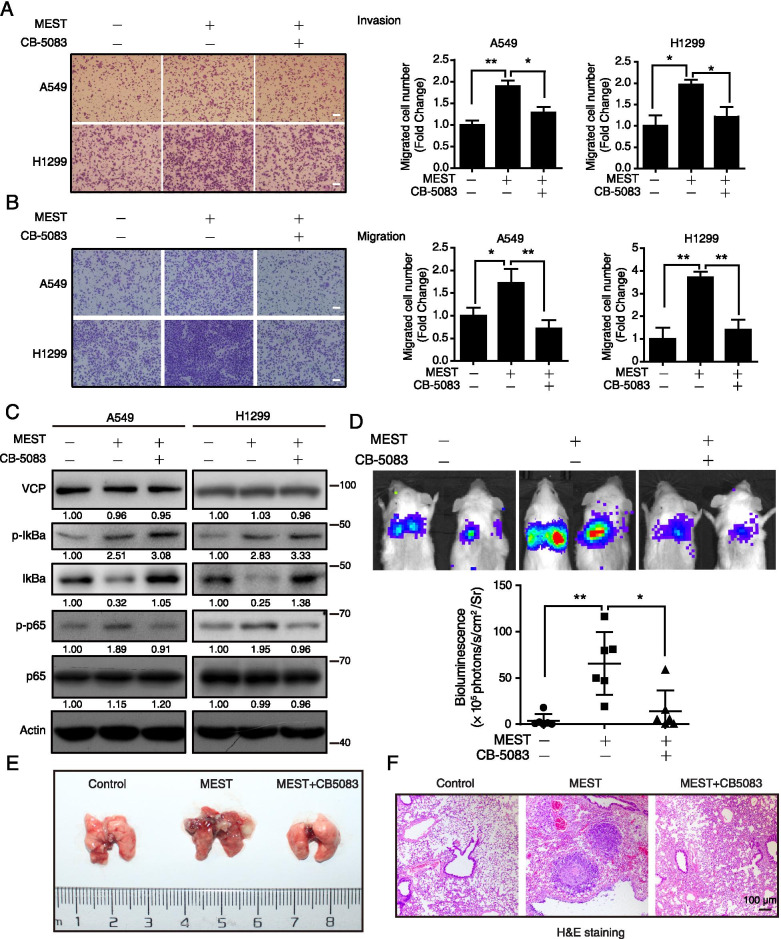
Inhibition of VCP suppresses the pro-metastatic effect of MEST. **A**, **B** MEST-overexpressing A549 and H1299 cells were treated with either 0.5 μM of CB-5083 or DMSO for 48 h and subjected to transwell assays to detect their invasion (**A**) and migration (**B**) abilities. Scale bar, 100 μm. **C** The NF-κB markers including p-IκBα, IκBα, p-p65, and p65 were determined by western blot analysis. **D-F** NCG mice were transplanted with luciferase-labeled cells that either with or without MEST overexpression (1 × 10^6^ cells per mouse) via tail vein injection (*n* = 6). The indicated treatment group or control group was orally administered CB-5083 (30 mg kg^−1^) or vehicle, respectively, every 2 days. Mice were visualized 1.5 months after transplantation by using an IVIS 200 Imaging System (**D**). Lungs harvested after imaging are shown (**E**). Note that CB-5083 suppresses the metastatic nodules formed by MEST-overexpressing A549 cells in the lungs. Pulmonary metastases in the mouse model were histologically analyzed by H&E staining (**F**); scale bar, 100 μm

Moreover, we employed CB-5083 (Figure S5A), a specific inhibitor of VCP, to study the role of VCP in MEST-mediated NF-κB activation and invasive phenotypes. WST-1 assays showed that CB-5083 did not exhibit anticancer effect on A549 and H1299 until 1 μM (Figure S5B). However, CB-5083 treatment significantly suppressed cell invasion and migration (Figure S5C), as well as NF-κB signaling, as evidenced by the accumulation of p-IκBα and IκBα and the reduction of p-p65 (Figure S5D). On the basis of this dose-dependent response, we used 0.5 μM of CB-5083 for subsequent studies. MEST-driven cell invasion and migration was abolished by CB-5083 treatment in both A549 and H1299 cells (Fig. 6A–B). MEST-induced IκBα degradation was blocked by CB-5083 treatment, resulting in the accumulation of IκBα and p-IκBα, which attenuated NF-κB activation induced by MEST (Fig. 6C). Furthermore, after intravenously injecting either MEST-overexpressing cells or control cells into tail vein of NCG mice, we found that MEST overexpression markedly promoted lung metastasis and, more importantly, that these effects were significantly abrogated by oral administration of CB-5083 (Fig. 6D–E). The lung metastatic nodules (Figure S5E) were further confirmed by H&E staining (Fig. 6F) and pan-keratin positive ratio Figure S5F–G). These results suggest that VCP is required for MEST to activate NF-κB signaling and for subsequent tumor metastasis.

We further deciphered how MEST increases IκBα phosphorylation. According to the IPA analysis, we found evidence for STAT3 signaling in the MEST-regulated inflammatory response network (Figure S6A). We thus speculated that MEST enhances the p-IκBα via STAT3 signaling. Recent study reported that MEST is essential for STAT3 activation [35], and STAT3 directly increases IKKα activity for IκBα phosphorylation [36]. Based on these findings, we confirmed that MEST overexpression increased the levels of p-STAT3, p-IKK, and p-IκBα in both A549 and H1299 cells (Figure S6B). This suggested that MEST-induced, STAT3-mediated IKK activation is essential for subsequent phosphorylation of IκBα and p65 [37]. Collectively, our results demonstrate that MEST controls NF-κB signaling at multiple levels: enhancing p-IκBα via the STAT3-IKK pathway and promoting IκBα degradation by binding VCP.

### Clinical significance of MEST and VCP in human lung cancer

**Fig. 7:**
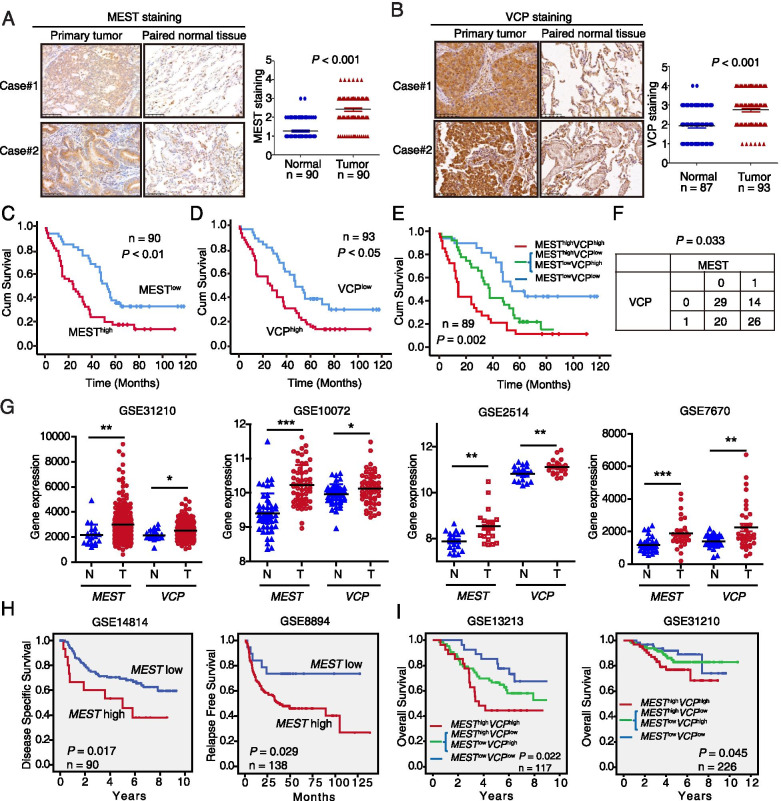
Overexpression of MEST and VCP are associated with poor prognosis in lung cancer patients. **A** Two representative immunohistochemistry (IHC) images of MEST expression in lung cancer tissues and in corresponding adjacent non-tumor lung tissues. Differences in MEST expression scores between lung cancer and corresponding adjacent non-tumor tissues are shown (*n* = 90). **B** Two representative IHC images of VCP expression in lung cancer tissues and in corresponding adjacent non-tumor lung tissues. Differences in VCP expression scores between lung cancer (*n* = 93) and non-cancerous tissues (*n* = 87) are shown. Kaplan–Meier survival analysis of lung cancer patients according to the expression of MEST (**C**) and VCP (**D**) by using median expression level as the cut-off point for survival analyses. High MEST and VCP expressions are significantly associated with shorter survival (**E**), and statistical significance was calculated by log-rank test (*P* = 0.002). **F** The correlation between the immunostaining intensity of the proteins MEST and VCP was determined by Fisher exact test. **G** MEST and VCP are highly expressed in lung cancer tissues as compared with non-tumor tissues. Analysis of *MEST* and *VCP* expression by using the GEO database. Plots derived from gene expression data in GEO (GSE31210, GSE10072, GSE2514, and GSE7670) comparing the expression of *MEST* and *VCP* gene in normal lung and lung cancer tissues. **H** Kaplan–Meier survival curves based on GEO datasets (GSE14814 and GSE8894) show the overall survival of patients with either high or low *MEST* expression levels. **I** Kaplan–Meier plots based on GEO datasets indicated that *MEST*^high^/*VCP*^high^ (red) is associated with worse overall survival in lung cancer patients compared with other groups. Survival curves of patients with tumor *MEST* and *VCP* expression above the median (green) and patients with tumor *MEST* and *VCP* expression below the median (blue) are indicated. Higher *MEST* expression predicts shorter overall survival in two lung cancer-associated GEO databases (GSE31210, GSE13213)

We measured the expressions of MEST and VCP by performing IHC of tissue microarrays containing 90 and 87 pairs of primary lung cancer tissues and adjacent normal tissues, respectively. We detected stronger MEST cytoplasmic staining in 68/90 (75.55%) lung cancer tissues as compared to corresponding normal tissues (Fig. 7A). A significant correlation between tumor MEST expression and pathological N-stage was observed (*P* = 0.036; Table 1). Moreover, up-regulation of VCP in cancer tissues was detected in 64.3% (56/87) cases, as compared with adjacent normal tissues (Fig. 7B). And a significant correlation between VCP expression and pathological N-stage is shown in Table 2 (*P* = 0.001). Multivariate Cox proportional hazard regression revealed that both N-stage (*P* < 0.05) and MEST expression (*P* = 0.042) were independent prognostic factors for overall survival of lung cancer patients (Tables S4, S5). More importantly, the patients with high MEST expression levels had significantly shorter survival (median survival = 25.0 months) than the patients with low MEST levels (median survival = 55.0 months). Analysis with log-rank showed that high MEST expression was significantly correlated with shorter survival (*P* < 0.01; Fig. 7C). Patients with high VCP expression level also had significantly shorter survival (median survival = 29 months, *P* < 0.05; Fig. 7D).

**Table 1:** Correlation between MEST expression levels and clinicopathological parameters in 90 cases of lung cancer

Variable	n	Low MEST	High MEST	*P* value
Age (years)				
≤ 55	23	11	12	
> 55	67	32	35	1.000
Gender				
Female	41	23	18	
Male	49	20	29	0.203
T-Stage				
1/2	67	35	32	
3/4	23	8	15	0.226
N-Stage				
N0	39	22	17	
N1	36	11	25	**0.036**
M-Stage				
M0	89	42	47	
M1	1	1	0	0.478
Pathologic stage				
Stages I & II	69	33	36	
Stages III & IV	21	10	11	1.000

Tumor histology: Lung adenocarcinoma

**Table 2:** Correlation between VCP expression levels and clinicopathological parameters in 93 cases of lung cancer

Variable	n	Low VCP	High VCP	*P* value
Age (years)				
≤ 55	24	9	15	
> 55	69	31	38	0.634
Gender				
Female	43	23	20	
Male	50	27	33	0.429
T-Stage				
1/2	70	31	39	
3/4	23	9	14	0.809
N-Stage				
N0	38	24	14	
N1	38	9	29	**0.001**
M-Stage				
M0	91	39	52	
M1	1	0	0	1.000
Pathologic stage				
Stages I & II	65	29	36	
Stages III & IV	28	11	17	0.656

Tumor histology: Lung adenocarcinoma

**Fig. 8:**
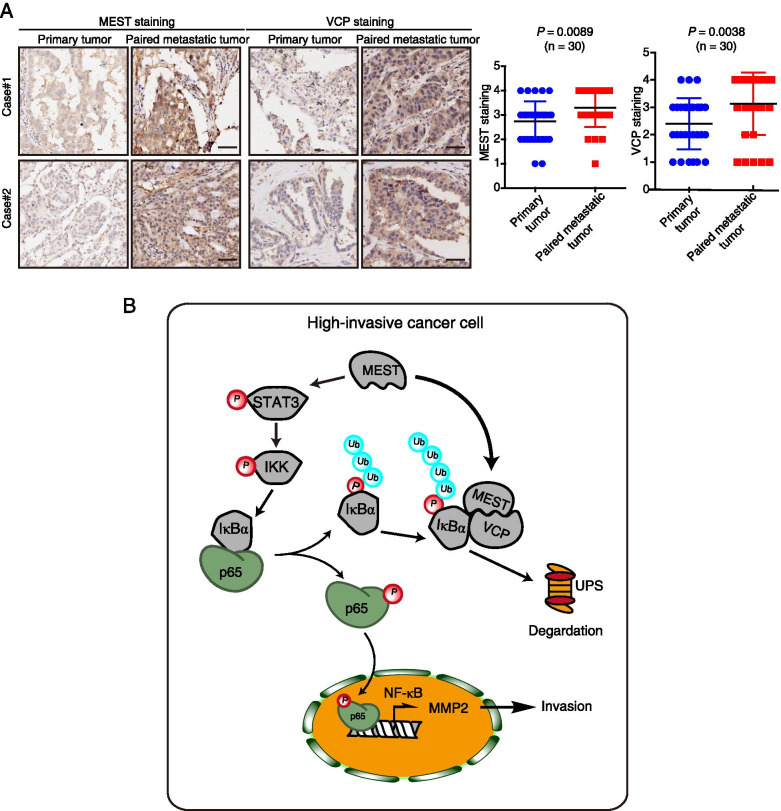
The expression of MEST and VCP in metastatic cancer and a schematic diagram of the action mechanism of MEST. **A** Two representative IHC images of MEST and VCP expression in primary lung cancer tissues and in corresponding metastatic tumor tissues. Differences in MEST and VCP expression scores between lung cancer and metastatic tumor tissues are shown (*n* = 30). Scale bar, 50 μm. **B** Working model of MEST on promotion of cancer metastasis. MEST is upregulated in highly invasive lung cancer cells, it interacts with VCP and increases VCP to capture IκBα for degradation, leading to the nuclear translocation of p65 to activate the NF-κB pathway for cancer metastasis. In addition, MEST can induce IκBα phosphorylation via the STAT3/IKK pathway, mediating NF-κB signaling activation at multiple levels

Interestingly, the patients with concomitant high MEST and VCP expression had even shorter survival (median survival = 15 months) than the patients with low MEST and VCP expression (median survival = 56 months; Fig. 7E). A positive correlation with statistical significance (*P* = 0.033) was found between MEST and VCP expression (Fig. 7F), highlighting a critical role of these two proteins in lung cancer progression. Furthermore, analyzing the expression of *MEST* and *VCP* by using the Gene Expression Omnibus database, we found that the expression levels of these genes in lung cancer tissues were markedly higher than in normal tissues (Fig. 7G). Kaplan–Meier analysis revealed that the patients with high *MEST* expression had shorter survival than the patients with low *MEST* expression (Fig. 7H). More importantly, the patients with *MEST*^high^/*VCP*^high^ showed poorer prognosis than the other groups (*P* < 0.05, Fig. 7I), corroborating our findings above. Another two tissue microarrays consisting of 30 primary lung cancer tissues and matched metastatic tissues were further used to determine MEST and VCP expression, respectively. The clinical features (age, gender, tumor laterality and pathologic stage) and analysis of lymph node metastasis were listed in Table S6. The immunohistochemistry data showed that the majority of the MEST and VCP expression levels in most metastatic tumors was higher than those in the matched primary tumors (Fig. 8A). Collectively, these results indicate that MEST and VCP could be prognostic biomarkers for patients with lung cancer.

## Discussion

Identification of key drivers in cancer metastasis is urgently needed to develop effective therapeutic strategies. Here, we established highly invasive lung cancer cell lines and performed a quantitative proteomics to identify novel regulatory proteins associated with cancer invasion. From our screen, the high-ranking candidate MEST drew our attention. We validated that MEST promotes lung cancer cell invasion and metastasis both in vitro and in vivo, and we confirmed that MEST is an important driver of lung cancer metastasis. Clinically, MEST is upregulated in lung cancer tissues, and high MEST expression significantly correlates with unfavorable clinicopathological features and with a shorter survival time of lung cancer patients. These results provide a strong rationale for further investigation of MEST as a prognostic biomarker in lung cancer.

NF-κB is increasingly recognized as a crucial player in many steps of cancer initiation and progression (including proliferation, survival, and metastasis) [38]. Constitutively active NF-κB is commonly observed in multiple human cancers but is rarely observed in normal tissues [39]. For example, NF-κB signaling promotes EMT progression [40] and endocrine therapy resistance [41] in breast cancer. Despite reluctance to use NF-κB inhibitors to treat cancer, combining classical chemotherapeutics seems to yield promising synergistic effects [42]. Therefore, NF-κBs and their upstream regulators are promising targets for therapeutic intervention. In this study, we demonstrated that the regulatory protein MEST sustains NF-κB signaling at multiple levels: not only by inducing IκBα phosphorylation through STAT3-IKK signaling, but also by accelerating IκBα degradation through VCP interaction. These findings reveal an essential role of MEST for modulating the NF-κB pathway in cancer. Activation of NF-κB imminently correlates with phosphorylation of IκBα [43]. IκBα phosphorylation at Ser-32 and Ser-36 signals for N-terminal poly-ubiquitination and subsequent degradation by the ubiquitin–proteasome pathway [44]. IκBα degradation releases NF-κBs to translocate into the nucleus and to initiate transcription of oncogenes (such as *MMP2*) [17]. Moreover, p65 (a critical NF-κB transcription factor subunit) is phosphorylated at Ser-536 and translocates into the nucleus to increase activity of the NF-κB transcription factor [15]. In this regard, we proved that MEST indeed induces nuclear localization of p65 (Fig. 3C), and upregulates p-p65 and p-IκBα (Figure S3A and Fig. 3D) to affect NF-κB signaling in lung cancer.

VCP is an important oncoprotein that mediates degradation of ubiquitin-labeled IκBα and HIF-1α [45, 46], but the regulatory mechanism remains to be elucidated. The ability of VCP to mediate degradation activity depends on a series of specific accessory proteins or cofactors; for example, the Npl4/Ufd1 heterodimer plays critical roles in the interaction between VCP and ubiquitinated client proteins [34]. We here identified VCP as a binding partner of MEST by using IP-MS (Fig. 4A-G). We then demonstrated that MEST can bind to VCP to increase the interaction between VCP and p-IκBα (Fig. 4H), therefore leading to IκBα degradation and subsequent NF-κB activation (Fig. 4J). More importantly, we observed that knockdown of VCP decreased MEST-driven cell migration, invasion, and NF-κB signaling, revealing that MEST requires VCP for NF-κB regulation. MEST knockdown-induced phenotypes are reversed by VCP overexpression, suggesting that MEST is an upstream regulator of VCP. In addition, inhibition of VCP (either by CB-5083 or by siRNA treatment) induced the accumulation of p-IκBα and IκBα (Fig. 5 and 6), confirming that VCP expression negatively correlates with p-IκBα expression [11]. Blockade of the 26S proteasome and VCP could conceivably lead to dramatic accumulation of ubiquitinated proteins. A schematic mechanism to describe the interactions among MEST, VCP, and IκBα, and how these interactions regulate cancer invasive transformation, is shown in Fig. 8B. In brief, MEST promotes the interaction between VCP and IκBα, which facilitates the degradation of IκBα and which induces p65-mediated NF-κB activation of cancer invasion.

Since the pleiotropic NF-κB signaling pathway is pivotal for lung cancer metastasis, NF-κB signaling has been targeted for the inhibition of lung cancer, for example, by using proteosome inhibitors like bortezomib and carfilzomib [47]. Apart from suppressing IKK activity, directly modulating IκBα stability is a promising strategy for clinical intervention because IκBα modulation does not activate numerous downstream NF-κB pathways that redundantly promote cancer cell survival. Our findings revealed a novel molecular event that the interaction between MEST and VCP is imperative for IκBα degradation. We propose that antagonists of this interaction may be effective for the treatment of metastatic lung cancer. In addition, our study highlights the value of considering both MEST and VCP expression levels for lung cancer prognosis, as evidenced by the correlation of their levels with lung cancer N-stage and patient survival. It would be interesting to further identify the upstream regulation of MEST to ask how cancer cell acquires MEST expression. It is also interesting to induce another cell metastatic model by injecting subcutaneously lung cancer cells and analyzing tumor cells disseminating in the lung to further evaluate the pro-metastasis effect of MEST on various metastasis progresses including extravasation, infiltration and dissemination.

## Conclusions

We report the discovery that MEST regulates lung cancer metastasis by activating the VCP/NF-κB/MMP2 signaling pathway. These results increase our understanding of how the NF-κB signaling pathway is dysregulated in metastatic cancer (Fig. 8B). High expression of both MEST and VCP correlate with unfavorable tumor stages and with shorter survival of patients. Therefore, MEST and VCP may be useful prognostic biomarkers and drug targets for lung cancer.

## Supplementary Information

**Additional file 1.** Supplementary material files.

## Data Availability

All data and materials supporting the findings of this work are available from its supplementary information files and from the corresponding author upon reasonable request.

## Abbreviations

MEST: Mesoderm-specific transcript
VCP: Valosin containing protein
MMP2: Matrix metalloproteinase 2
qRT-PCR: Quantitative real-time PCR
IHC: Immunohistochemistry
DEPs: Differentially expressed proteins
FRET: Fluorescence resonance energy transfer
CHX: Cycloheximide
SDS: Sodium dodecyl sulfate
IPA: Ingenuity Pathway Analysis
Co-IP: Co-immunoprecipitation
MS: Mass spectrometry
TCGA: Cancer Genome Atlas
GEO: Gene Expression Omnibus
EMT: Epithelial-mesenchymal transition
SILAC: Stable isotope labeling with amino acids in cell culture
