# Supplementary material for: MEST promotes lung cancer invasion and metastasis by interacting with VCP to activate NF-κB signaling
Source: J Exp Clin Cancer Res. 2021 Sep 24;40:301. doi: 10.1186/s13046-021-02107-1 (PMC8464132; doi:10.1186/s13046-021-02107-1)
Supplement: Supplementary file 1 — Additional file 1. Supplementary material files. [file 13046_2021_2107_MOESM1_ESM.pdf]

MEST Promotes Lung Cancer Invasion and Metastasis by Interacting with VCP to Activate NF-κB Signaling

Yang Wang[1], Jing Zhang[1], Yang-Jia Li[1], Nan-Nan Yu[1], Wan-Ting Liu[1], Jun-Ze Liang[1], Wen-Wen Xu[2], Zheng-Hua Sun[1], Bin Li[*, 1], Qing-Yu He[*, 1]

[1]MOE Key Laboratory of Tumor Molecular Biology and Key Laboratory of Functional Protein Research of Guangdong Higher Education Institutes, Institute of Life and Health Engineering, Jinan University, Guangzhou 510632, China; [2]MOE Key Laboratory of Tumor Molecular Biology and Guangdong Provincial Key Laboratory of Bioengineering Medicine, National Engineering Research Center of Genetic Medicine, Institute of Biomedicine, Jinan University, Guangzhou 510632, China.

Supplementary Materials

Supplementary Figure S1.

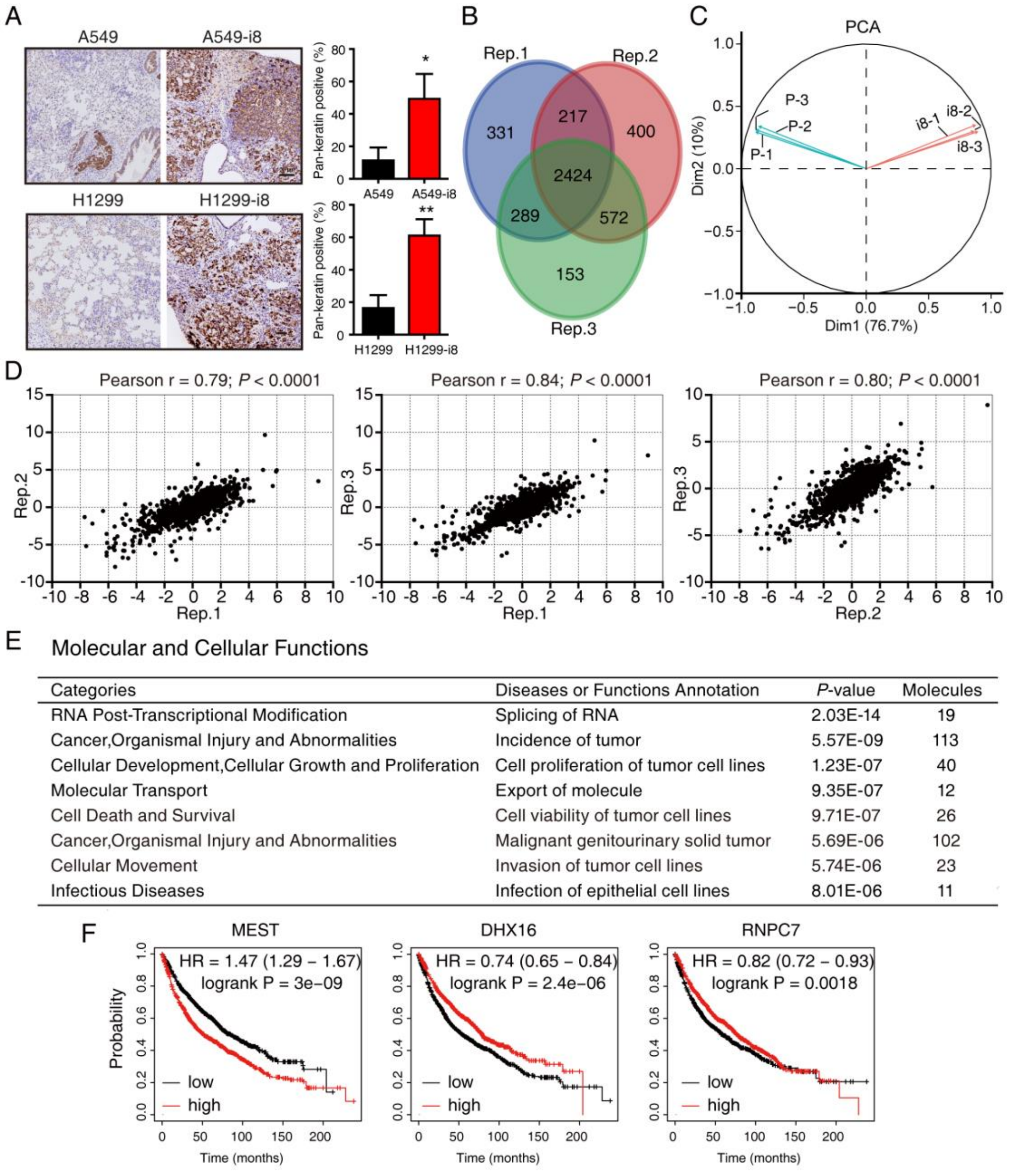

Categories	Diseases or Functions Annotation	*P*-value	Molecules
RNA Post-Transcriptional Modification	Splicing of RNA	2.03E-14	19
Cancer,Organismal Injury and Abnormalities	Incidence of tumor	5.57E-09	113
Cellular Development,Cellular Growth and Proliferation	Cell proliferation of tumor cell lines	1.23E-07	40
Molecular Transport	Export of molecule	9.35E-07	12
Cell Death and Survival	Cell viability of tumor cell lines	9.71E-07	26
Cancer,Organismal Injury and Abnormalities	Malignant genitourinary solid tumor	5.69E-06	102
Cellular Movement	Invasion of tumor cell lines	5.74E-06	23
Infectious Diseases	Infection of epithelial cell lines	8.01E-06	11

Figure S1

Figure S1. IPA analysis of the differentially expressed proteins in A549-i8. (A) NCG mice were transplanted with A549-i8 cells or H1299-i8 cells, or their corresponding parental cells *via* tail vein injection, lungs harvested after imaging were histologically analyzed by pan-keratin staining, and the cytokeratin-positive cells were quantified. Scale bar, 100 μm. Bars, SD. *, *P* < 0.05; **, *P* < 0.01 compared with control group unless otherwise indicated. (B) Venn diagram of the protein numbers identified in three independent SILAC experiments. (C) Principal component

analysis (PCA) of A549 parental and A549-i8 in three independent experiments. Dim 1 and Dim 2: principal component 1 and principal component 2. Red lines (i8) and blue lines (parental) represent 3 biological replicates, respectively. (D) Correlation between independent biological replicates. Pearson correlation coefficients (r) and their *P* values were shown as indicated. (E) The differentially expressed proteins in A549-i8 were uploaded to IPA analysis, showing that the top five molecular and cellular functions were relevant to cell development, cell growth, cell movement and cell survival. (F) The expressions of MEST, DHX16 and RNPC7 in lung cancer versus the survival of patients were plotted using Kaplan-Meier plotter software.

Supplementary Figure S2.

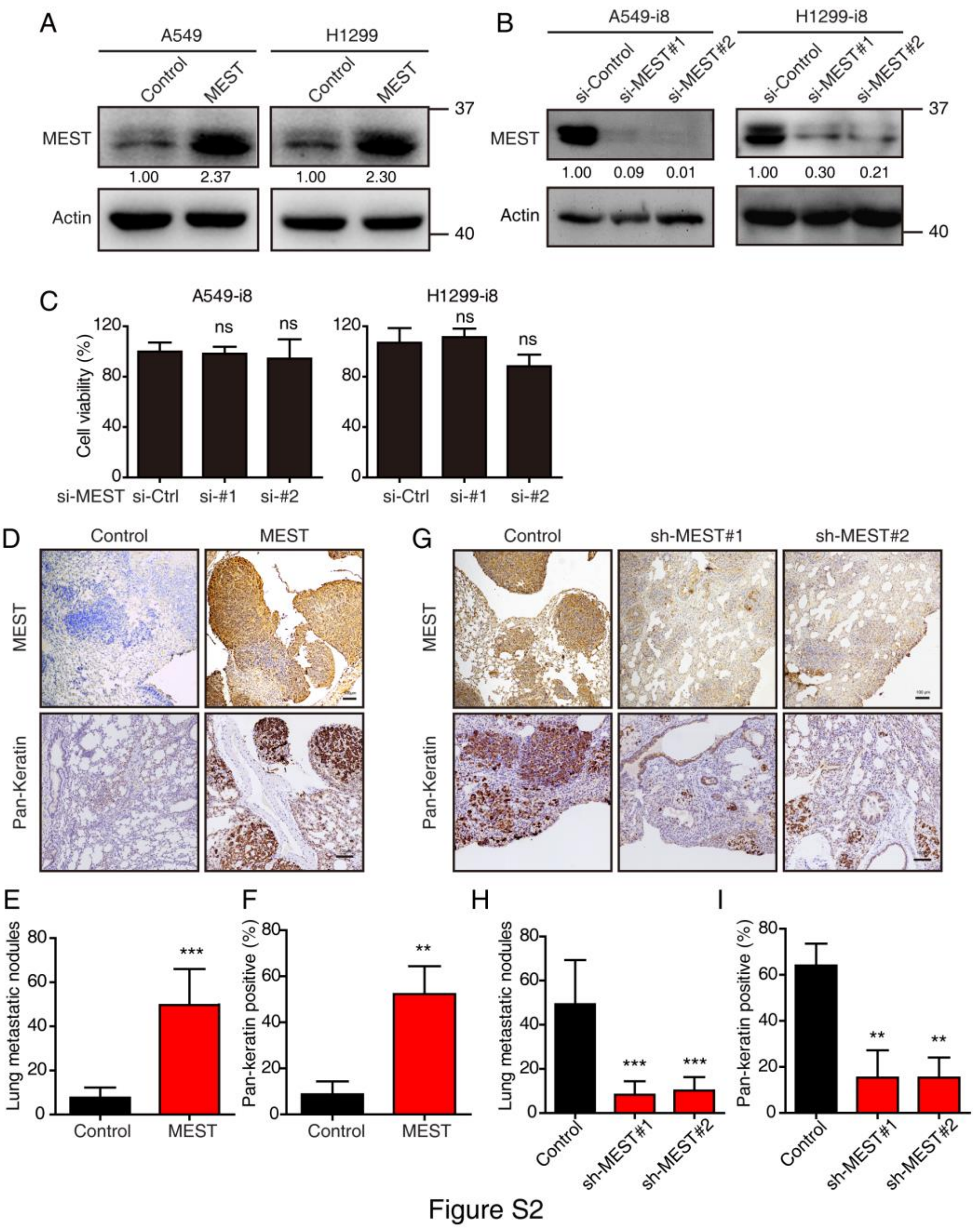

Figure S2. The overexpression and knockdown effects of MEST in lung cancer cells. (A, B) A549 and H1299 cells were overexpressed with MEST, while A549-i8 and H1299-i8 cells were transfected with two anti-MEST siRNA (si-MEST#1 and si-MEST#2), and the overexpression and knockdown effects were detected by western blot analysis. (C) Knockdown of MEST do not influence cell viability. A549-i8 and H1299-i8 were transfected with two anti-MEST siRNA (100 nM) or control siRNA for 48 h, and the cell viability was detected by WST-1. NS, no

significant difference, as compared to control group. (D-F) Nude mice were transplanted with the luciferase-labeled cells with or without MEST overexpression *via* tail vein injection, the lungs of mice were harvested 1 month post-transplantation. The expression of MEST and pan-keratin on pulmonary metastases in the mouse model was histologically analyzed by IHC staining (D). Lungs from mice injected with MEST-overexpressing cells showed significantly more incidence of lung metastasis, as evidenced by the increase of lung metastatic nodules (E) and pan-keratin positive ratio (F). (G-I) NCG mice were transplanted with the luciferase-labeled cells expressing two shRNA against MEST *via* tail vein injection; the mice were visualized 1.5 months post-transplantation using the IVIS 200 Imaging System. The expression of MEST and pan-keratin in metastatic nodes in the mouse model was histologically analyzed by IHC staining (G). Lungs from mice injected with MEST knockdown cells showed significantly reduced incidence of lung metastasis, as evidenced by the decrease of lung metastatic nodules (H) and pan-keratin positive ratio (I). Bars, SD. ***, $P < 0.001$ compared with control group unless otherwise indicated. Scale bar, 100 μm.

Supplementary Figure S3.

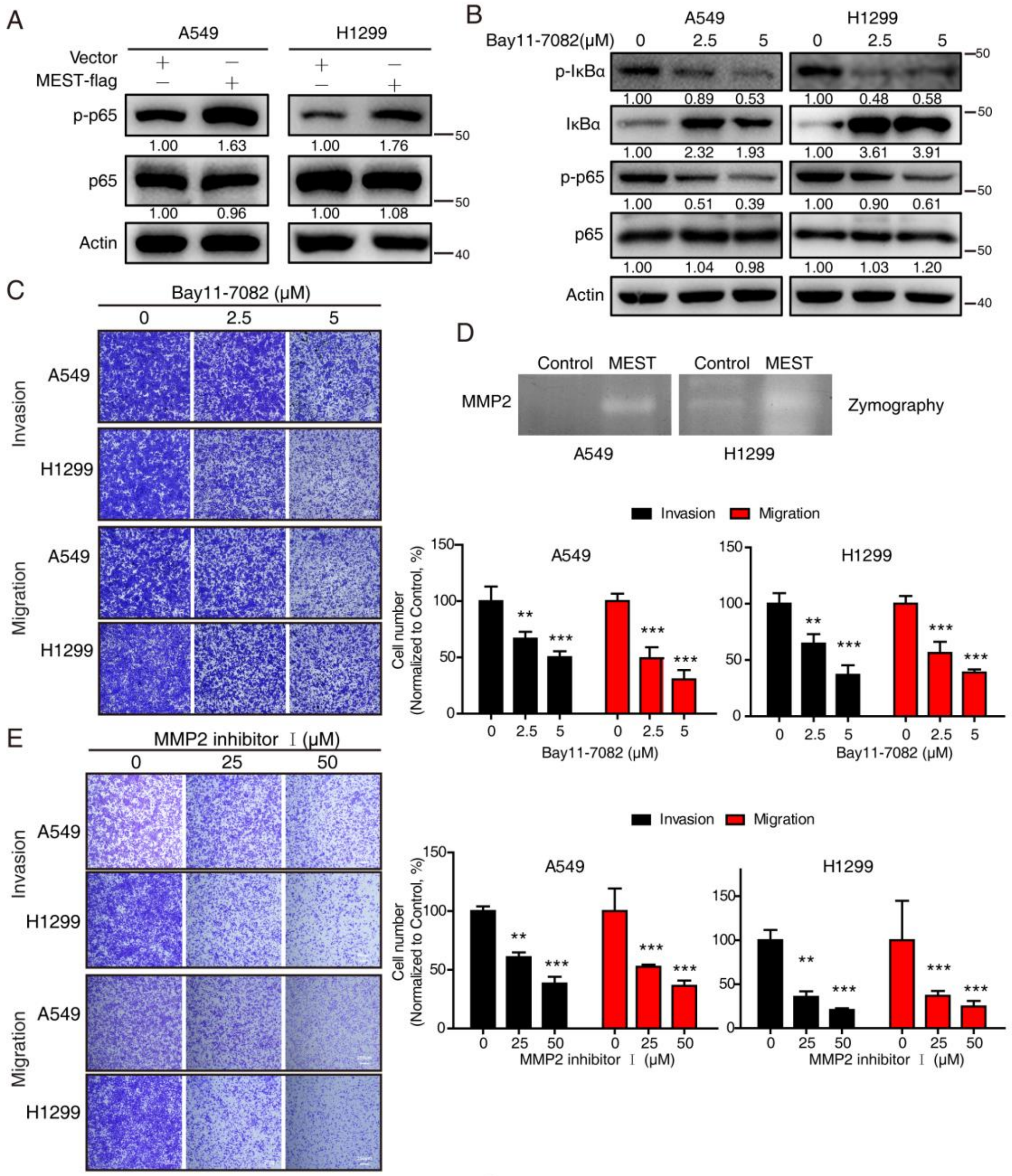

Figure S3

Figure S3. Inhibitory effect of NF-κB inhibitor Bay11-7082 on NF-κB signaling, cell invasion and migration. (A) MEST overexpression promotes the phosphorylation of NF-κB subunits (p65) in both A549 and H1299 cells. (B) A549 and H1299 were treated with 2.5 and 5 μM of Bay11-7082 for 24 h, and the cell lysates were subjected to western blot analysis for the detection of p-IκBα, IκBα, p-p65 and p65, and the cell invasion and migration were detected by transwell assays (C). (D) Ectopic MEST expression induces enzymatic activities of MMP-2 in both

cell lines, as determined by zymography assay. (E) A549 and H1299 were treated with 25 and 50 μM of MMP2 inhibitor I for 24 h, and the cell invasion and migration were detected by transwell assays. Bars, SD. **, $P < 0.01$; ***, $P < 0.001$ compared with control group unless otherwise indicated. Scale Bar, 250 μm.

Supplementary Figure S4.

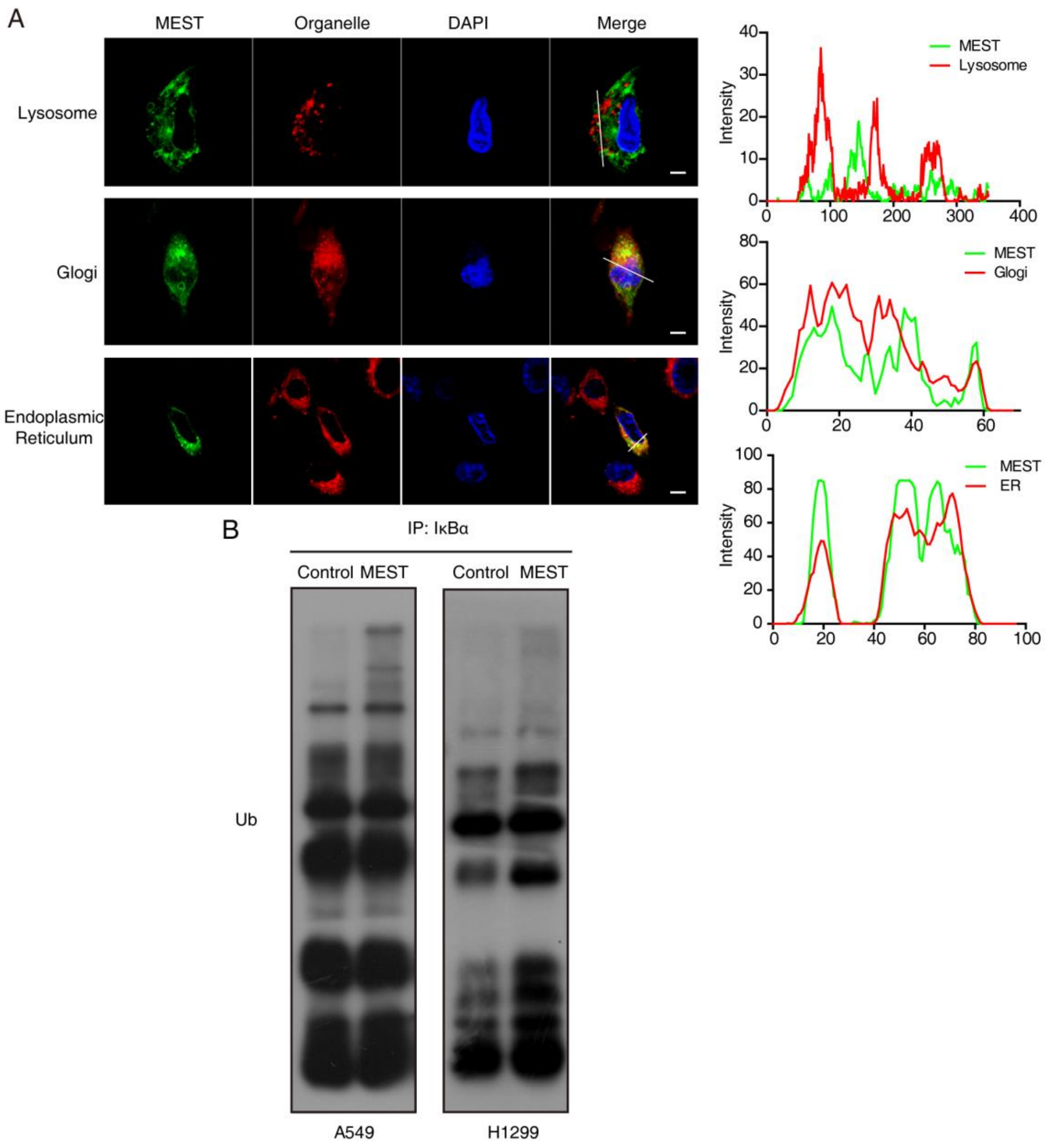

Figure S4

Figure S4. Cellular localization of MEST. (A) A549 cells transfected with MEST-eGFP plasmid for 24 h were stained with lysosome tracker, Glogi tracker and Endoplasmic Reticulum tracker, respectively, and imaged by confocal microscope. Scale Bar, 5 µm. The intensity profiles of MEST and lysosome/Glogi/ER along the white line are plotted in right panels. (B) A549 and H1299 transfected with vector or MEST-flag plasmids for 24 h, the ubiquitination of IκBα was determined by co-IP assay using anti-IκBα antibody.

Supplementary Figure S5.

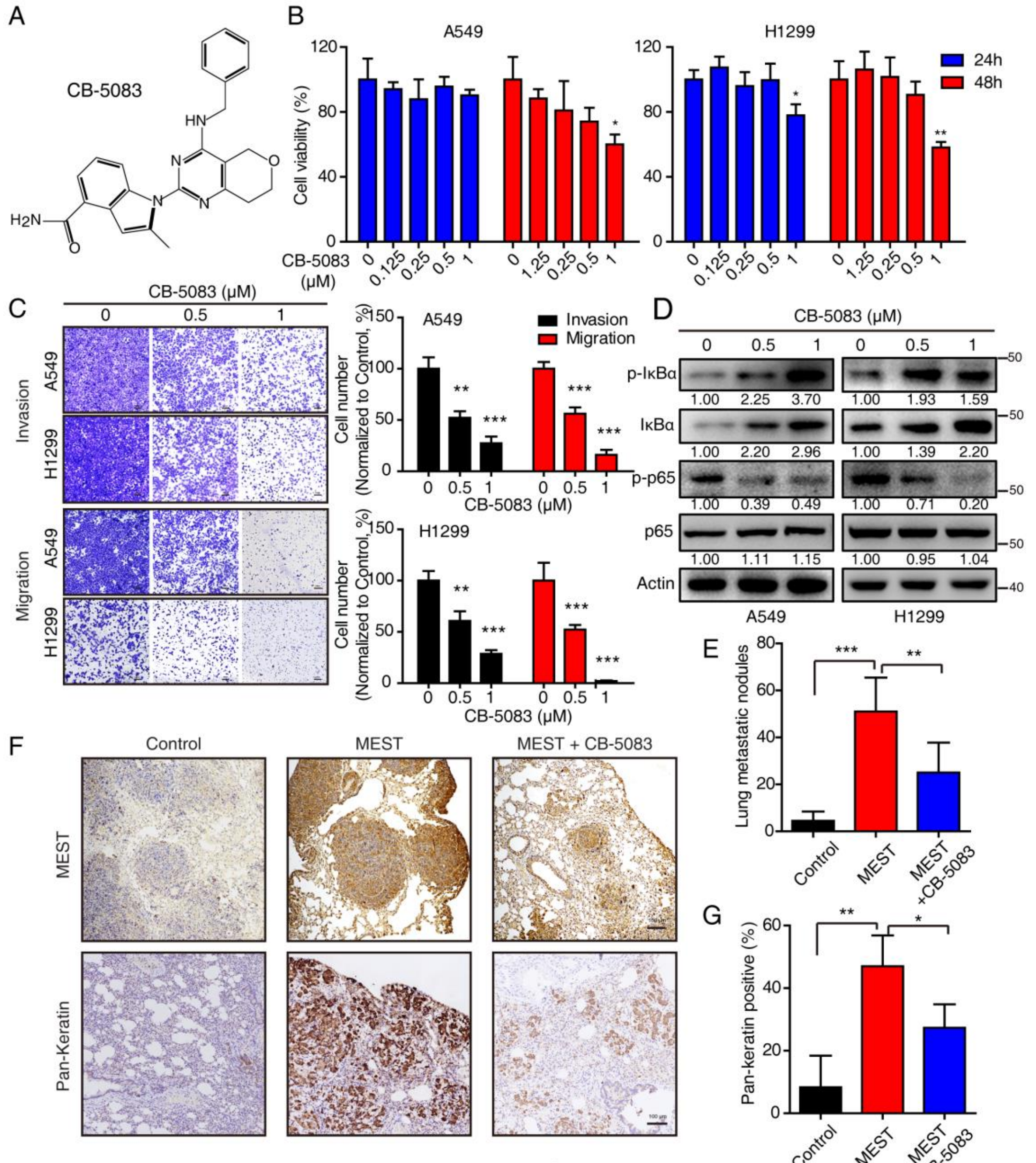

Figure S5

Figure S5. Effect of CB-5083 on cell invasion and migration, and NF-κB signaling. (A) The chemical structure of CB-5083, a VCP specific inhibitor. (B) Both A549 and H1299 cells were treated with elevating concentration of CB-5083 (up to 1 μM) or DMSO for 24 and 48 h, respectively, and the cell viability was detected by WST-1 assay. (C) A549 and H1299 were treated with 0.5 and 1 μM of CB-5083 for 48 h, and the cell invasion and migration were detected by transwell assays, and the protein expressions of p-IκBα, IκBα, p-p65 and p65 were determined by western blot

analysis (D). (E-G) NCG mice were transplanted with A549 cells with or without MEST overexpression *via* tail vein injection, the indicated treatment group and control group were respectively orally administered either CB-5083 (30 mg kg^{-1}) or vehicle every two days. The lungs were harvested 1.5 months post-transplantation. The expression of MEST and pan-keratin in metastatic nodules was determined by IHC staining (F). Lungs from mice injected with MEST-expressing cells showed significantly more incidence of lung metastasis, while treatment of CB-5083 suppressed this effect, as evidenced by the lung metastatic nodules (E) and pan-keratin positive ratio (G). scale bar, 100 μm. Bars, SD. *, $P < 0.05$; **, $P < 0.01$; ***, $P < 0.001$ compared with control group unless otherwise indicated.

Supplementary Figure S6.

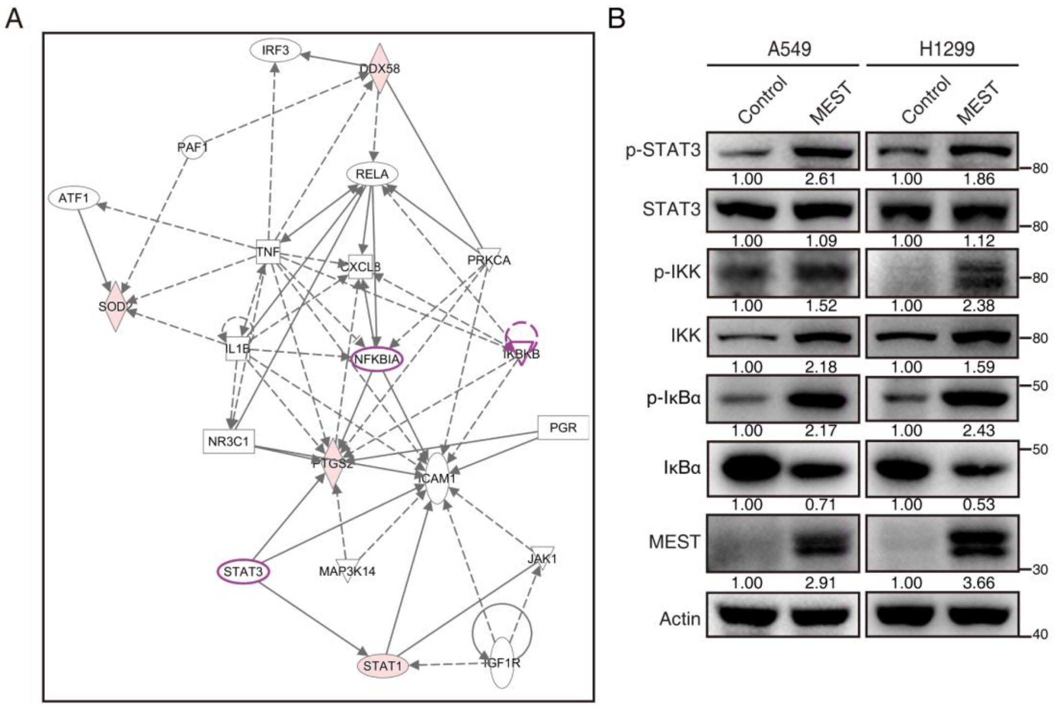

Figure S6

Figure S6. SILAC proteomics reveals that MEST activates STAT3-IKK signaling. (A) Two independent label-swap experiments including forward labeling (MEST, “Light”) and reverse labeling (MEST, “High”) were performed. The MEST regulated differentially expressed proteins were analyzed by IPA, showing that MEST-regulated

proteins are mainly involved in the regulation of cell survival and inflammatory response, including STAT3 and NF-κB signaling. (B) MEST induces IκBα phosphorylation by activating STAT3-IKK signaling. Cell lysates of A549 and H1299 with or without MEST overexpression were subjected to western blot analysis for the detection of p-STAT3, STAT3, p-IKK, IKK, p-IκBα and IκBα.

Supplementary Table

Supplementary Table. S1 Overview of proteins identifications and quantifications for the comparisons of A549-i8 (labeled with "light" chain) verse parental A549 (labeled with "heavy" chain).

See separate excel file.

Supplementary Table. S2 Differentially expressed proteins regulated by MEST overexpression.

See separate excel file.

Supplementary Table. S3 Potential MEST binding proteins identified by co-immunoprecipitation/ MS.

See separate excel file.

Supplementary Table. S4. Cox proportional hazard regression analyses of MEST in lung cancer patients

	Clinicopathological features	HR	95% CI	*P* value
Gender	Female *vs*. Male	0.899	0.488-1.658	0.734
Age	＞55 years *vs*. ≤55 years	1.083	0.564-2.079	0.811
stage	Stages I & II *vs*. Stages III & IV	0.899	0.448-1.804	0.764
T	T1/2 *vs*. T3/4	1.067	0.559-2.037	0.844
N	N0 *vs*. N1	0.307	0.165-0.572	**0.0002**
M	M0 *vs*. M1	0.364	0.042-3.165	0.36
MEST expression	Absent *vs*. Present	0.541	0.299-0.979	**0.042**

Supplementary Table S5. Cox proportional hazard regression analyses of VCP in lung cancer patients

	Clinicopathological features	HR	95% CI	*P* value
Gender	Female *vs*. Male	1.039	0.548-1.972	0.906
Age	＞55 years *vs*. ≤55 years	0.899	0.469-1.725	0.749
stage	Stages I & II *vs*. Stages III & IV	0.85	0.457-1.581	0.608
T	T1/2 *vs*. T3/4	0.85	0.446-1.619	0.621
N	N0 *vs*. N1	0.475	0.237-0.95	**0.035**
M	M0 *vs*. M1	0.521	0.061-4.413	0.55
VCP score	Absent *vs*. Present	1.539	0.773-3.065	0.22

Supplementary Table. S6. The characteristics of patients in tissue microarray consisting of 30 primary lung cancer tissues and matched metastatic tissues

Characteristics	No. Cases (%)
**Age (y)**	
≤55	16 (53.33%)
＞55	14 (46.67%)
**Gender**	
Male	16 (53.33%)
Female	14 (46.67%)
**Tumour laterality**	
RUL	11 (36.67%)
RML	4 (13.33%)
RLL	4 (13.33%)
LUL	7 (23.33%)
LLL	4 (13.33%)
**Pathologic stage**	
＜ II	20 (66.67%)
≥ II	10 (33.33%)
**No. Lymph node metastasis**	
≤3	17 (56.67%)
＞3	13 (43.33%)
**Lymph Node Metastasis**	
Suprackavuclar zone (#1)	4 (13.33%)
Upper zone (#2, #3, #4)	9 (30.00%)
AP zone (#5, #6)	5 (16.67%)
lower zone (#7, #8, #9)	4 (13.33%)
N1 nodes (#10, #11, #12, #13, #14)	8 (26.67%)

RUL, right upper lobe; RML, right middle lobe; RLL, right lower lobe; LUL, left upper lobe; LLL, left lower lobe. The lymph node metastasis analysis is based on IASLC lymph node map.

Supplementary Table. S1 Overview of proteins identifications and quantifications for the comparisons of A549-i8 (labeled with "light" chain) verse parental A549 (labeled with "heavy" chain)

Uniprot ID	Gene name	Unique peptides	Abundance						Abundance Ratio (Light/Heavy)				*P* -value	Adjusted *P* -value
			Light			Heavy								
			I8-1	I8-2	I8-3	P-1	P-2	P-3	(I8-1) /(P-1)	(I8-2) /(P-2)	(I8-2) /(P-3)	Mean FC		
P02768	ALB	9	199.9	200	199.9	0.1	0.1	0.1	1999.00	2000.00	1999.00	1999.33	1.86E-14	1.09E-07
Q9NQW6	ANLN	18	182.7	182.4	182	17.3	17.6	18	10.56	10.36	10.11	10.35	5.51E-11	0.000161819
O14683	TP53I11	3	3.8	2.8	3.2	196.2	197.2	196.8	0.02	0.01	0.02	0.02	1.22E-10	0.000239254
P40222	TXLNA	18	151.5	151.5	150.9	48.5	48.5	49.1	3.12	3.12	3.07	3.11	3.47E-10	0.000290862
P31327	CPS1	56	9	7.8	8.2	191	192.2	191.8	0.05	0.04	0.04	0.04	3.29E-10	0.000290862
Q13740	ALCAM	15	5.4	6.5	6.4	194.6	193.5	193.6	0.03	0.03	0.03	0.03	2.93E-10	0.000290862
Q14315	FLNC	147	177	177.7	176.8	23	22.3	23.2	7.70	7.97	7.62	7.76	2.34E-10	0.000290862
Q15424	SAFB	8	169.2	169.3	168.3	30.8	30.7	31.7	5.49	5.51	5.31	5.44	6.79E-10	0.000498788
O60506	SYNCRIP	26	166.7	165.7	166.9	33.3	34.3	33.1	5.01	4.83	5.04	4.96	1.46E-09	0.000954296
Q5BKZ1	ZNF326	11	172.5	172.8	171.3	27.5	27.2	28.7	6.27	6.35	5.97	6.20	2.43E-09	0.001430171
O14497	ARID1A	27	159.8	160.4	161.3	40.2	39.6	38.7	3.98	4.05	4.17	4.06	4.04E-09	0.002158611
P98179	RBM3	4	163.1	161.4	162	36.9	38.6	38	4.42	4.18	4.26	4.29	6.16E-09	0.002786216
Q96KR1	ZFR	23	165.8	167.4	167.4	34.2	32.6	32.6	4.85	5.13	5.13	5.04	6.07E-09	0.002786216
P36639	NUDT1	4	157.2	156.7	155.5	42.8	43.3	44.5	3.67	3.62	3.49	3.60	9.55E-09	0.003586653
Q02952	AKAP12	85	190.6	188.6	191.4	9.4	11.4	8.6	20.28	16.54	22.26	19.69	1.09E-08	0.003586653
Q9H307	PNN	10	167.6	165.8	167.5	32.4	34.2	32.5	5.17	4.85	5.15	5.06	8.68E-09	0.003586653
P06703	S100A6	4	5.9	8.6	6.2	194.1	191.4	193.8	0.03	0.04	0.03	0.04	1.06E-08	0.003586653
Q587J7	TDRD12	32	197.7	194.9	195.2	2.3	5.1	4.8	85.96	38.22	40.67	54.95	1.10E-08	0.003586653
Q14195	DPYSL3	12	159.7	157.8	159	40.3	42.2	41	3.96	3.74	3.88	3.86	1.19E-08	0.003666527
P04264	KRT1	40	195.5	195	192.5	4.5	5	7.5	43.44	39.00	25.67	36.04	1.40E-08	0.004125191
P21980	TGM2	29	18.7	16.9	19.7	181.3	183.1	180.3	0.10	0.09	0.11	0.10	1.53E-08	0.00426903
Q15758	SLC1A5	13	50.4	49.5	48.6	149.6	150.5	151.4	0.34	0.33	0.32	0.33	1.68E-08	0.004488927
P14866	HNRNPL	23	160.3	159.5	161.7	39.7	40.5	38.3	4.04	3.94	4.22	4.07	1.91E-08	0.004884775
P48163	ME1	14	20.7	23.4	21.1	179.3	176.6	178.9	0.12	0.13	0.12	0.12	2.00E-08	0.004900772
P30530	AXL	15	182.3	185.5	184.6	17.7	14.5	15.4	10.30	12.79	11.99	11.69	2.47E-08	0.005795856
P0DJ93	SMIM13	2	130.2	131.2	130	69.8	68.8	70	1.87	1.91	1.86	1.88	3.30E-08	0.00746495
P33240	CSTF2	3	133.3	133.8	134.8	66.7	66.2	65.2	2.00	2.02	2.07	2.03	4.26E-08	0.008297028
P43358	MAGEA4	13	195.9	199.8	199.7	4.1	0.2	0.3	47.78	999.00	665.67	570.82	4.33E-08	0.008297028
O14684	PTGES	2	13.2	9.8	13.2	186.8	190.2	186.8	0.07	0.05	0.07	0.06	4.14E-08	0.008297028
Q9Y520	PRRC2C	39	153.8	151.5	153	46.2	48.5	47	3.33	3.12	3.26	3.24	3.99E-08	0.008297028
O60231	DHX16	14	173	176.2	173.7	27	23.8	26.3	6.41	7.40	6.60	6.81	4.38E-08	0.008297028
Q5EB52	MEST	4	175.3	178.8	177.5	24.7	21.2	22.5	7.10	8.43	7.89	7.81	4.59E-08	0.008435874
O60701	UGDH	43	22.8	19.2	21.5	177.2	180.8	178.5	0.13	0.11	0.12	0.12	4.76E-08	0.008481063
P29401	TKT	50	28.5	25.4	28.2	171.5	174.6	171.8	0.17	0.15	0.16	0.16	5.11E-08	0.008838539

P08133	ANXA6	49	169.6	167.5	170.9	30.4	32.5	29.1	5.58	5.15	5.87	5.54	6.24E-08	0.009697422
Q07666	KHDRBS1	11	164.7	163.2	166.4	35.3	36.8	33.6	4.67	4.43	4.95	4.68	6.22E-08	0.009697422
P49756	RBM25	11	173.5	170.8	174.2	26.5	29.2	25.8	6.55	5.85	6.75	6.38	6.15E-08	0.009697422
O95352	ATG7	11	145.2	143.2	145	54.8	56.8	55	2.65	2.52	2.64	2.60	6.27E-08	0.009697422
Q06323	PSME1	15	60.4	62.2	61.9	139.6	137.8	138.1	0.43	0.45	0.45	0.44	6.56E-08	0.009878014
P33316	DUT	12	145.8	146.9	148.2	54.2	53.1	51.8	2.69	2.77	2.86	2.77	7.13E-08	0.00997524
A0FGR8	ESYT2	11	62.5	64	64.2	137.5	136	135.8	0.45	0.47	0.47	0.47	7.05E-08	0.00997524
P48507	GCLM	9	57.4	55.2	56.5	142.6	144.8	143.5	0.40	0.38	0.39	0.39	6.88E-08	0.00997524
Q9H2H9	SLC38A1	3	171.2	174.2	170.8	28.8	25.8	29.2	5.94	6.75	5.85	6.18	7.36E-08	0.010061529
O95864	FADS2	7	3.9	8.8	4.9	196.1	191.2	195.1	0.02	0.05	0.03	0.03	9.53E-08	0.012443553
P17096	HMGA1	4	181.1	177.1	177.7	18.9	22.9	22.3	9.58	7.73	7.97	8.43	9.43E-08	0.012443553
Q8NDI1	EHBP1	17	28.7	26.1	24.6	171.3	173.9	175.4	0.17	0.15	0.14	0.15	1.05E-07	0.013188108
O00267	SUPT5H	38	165.4	165.5	168.7	34.6	34.5	31.3	4.78	4.80	5.39	4.99	1.05E-07	0.013188108
O15438	ABCC3	12	4.3	5.7	9.5	195.7	194.3	190.5	0.02	0.03	0.05	0.03	1.14E-07	0.013454496
P06396	GSN	30	170.9	167.1	170	29.1	32.9	30	5.87	5.08	5.67	5.54	1.12E-07	0.013454496
P55011	SLC12A2	28	27.7	24.2	28	172.3	175.8	172	0.16	0.14	0.16	0.15	1.14E-07	0.013454496
Q96QD8	SLC38A2	5	171.4	170.9	174.9	28.6	29.1	25.1	5.99	5.87	6.97	6.28	1.37E-07	0.015752142
P15144	ANPEP	31	178.2	178.9	174.5	21.8	21.1	25.5	8.17	8.48	6.84	7.83	1.46E-07	0.015937572
O00244	ATOX1	2	170.8	168.5	172.8	29.2	31.5	27.2	5.85	5.35	6.35	5.85	1.43E-07	0.015937572
P84103	SRSF3	8	152.6	150.8	149.5	47.4	49.2	50.5	3.22	3.07	2.96	3.08	1.45E-07	0.015937572
O95239	KIF4A	29	185.7	182.7	188	14.3	17.3	12	12.99	10.56	15.67	13.07	1.56E-07	0.016333973
P46821	MAP1B	93	40.9	37.9	41.3	159.1	162.1	158.7	0.26	0.23	0.26	0.25	1.54E-07	0.016333973
P18887	XRCC1	12	172.6	169.8	168.1	27.4	30.2	31.9	6.30	5.62	5.27	5.73	1.83E-07	0.018664485
O60502	MGEA5	32	166.6	162.4	164.7	33.4	37.6	35.3	4.99	4.32	4.67	4.66	1.87E-07	0.018664485
Q9UKL0	RCOR1	3	151.3	151.4	154.3	48.7	48.6	45.7	3.11	3.12	3.38	3.20	1.87E-07	0.018664485
Q05682	CALD1	19	51	49.6	52.8	149	150.4	147.2	0.34	0.33	0.36	0.34	1.93E-07	0.018931461
O15344	MID1	3	14.2	14.8	19.3	185.8	185.2	180.7	0.08	0.08	0.11	0.09	2.03E-07	0.019091406
O60341	KDM1A	11	148	145.3	145.4	52	54.7	54.6	2.85	2.66	2.66	2.72	2.00E-07	0.019091406
Q9UQE7	SMC3	40	157.8	154.1	156.4	42.2	45.9	43.6	3.74	3.36	3.59	3.56	2.05E-07	0.019091406
O14786	NRP1	6	3.8	3.7	9.3	196.2	196.3	190.7	0.02	0.02	0.05	0.03	2.21E-07	0.019984699
P62328	TMSB4X	6	43.3	44.8	47	156.7	155.2	153	0.28	0.29	0.31	0.29	2.19E-07	0.019984699
Q9H7B2	RPF2	5	138.7	138.8	136.5	61.3	61.2	63.5	2.26	2.27	2.15	2.23	2.28E-07	0.020297592
Q8IYB3	SRRM1	8	147.9	148.6	145.5	52.1	51.4	54.5	2.84	2.89	2.67	2.80	2.32E-07	0.020319152
Q9HAV4	XPO5	36	166.8	162.3	164.7	33.2	37.7	35.3	5.02	4.31	4.67	4.66	2.46E-07	0.021227795
O00425	IGF2BP3	11	144.4	143.6	146.7	55.6	56.4	53.3	2.60	2.55	2.75	2.63	2.75E-07	0.02339157
P55061	TMBIM6	2	146	144.1	147.4	54	55.9	52.6	2.70	2.58	2.80	2.69	2.84E-07	0.023488599
Q6IA69	NADSYN1	6	25.9	29.9	30.7	174.1	170.1	169.3	0.15	0.18	0.18	0.17	2.84E-07	0.023488599
Q12797	ASPH	21	51.9	48.4	51	148.1	151.6	149	0.35	0.32	0.34	0.34	3.01E-07	0.024551549
P18583	SON	9	174.2	174.8	169.8	25.8	25.2	30.2	6.75	6.94	5.62	6.44	3.27E-07	0.026297358
Q10570	CPSF1	24	146.2	144	147.5	53.8	56	52.5	2.72	2.57	2.81	2.70	3.67E-07	0.028393685
O95479	H6PD	9	52	53.7	50	148	146.3	150	0.35	0.37	0.33	0.35	3.66E-07	0.028393685

P43243	MATR3	30	165.8	161.7	161.5	34.2	38.3	38.5	4.85	4.22	4.19	4.42	3.66E-07	0.028393685
O43143	DHX15	30	129.8	128.1	130.3	70.2	71.9	69.7	1.85	1.78	1.87	1.83	3.94E-07	0.028575957
Q9BVG4	PBDC1	6	112.2	111.6	111.3	87.8	88.4	88.7	1.28	1.26	1.25	1.27	3.92E-07	0.028575957
P04179	SOD2	12	27.7	32.3	27.3	172.3	167.7	172.7	0.16	0.19	0.16	0.17	3.92E-07	0.028575957
Q92922	SMARCC1	19	174.3	168.9	170.5	25.7	31.1	29.5	6.78	5.43	5.78	6.00	3.82E-07	0.028575957
P26640	VARS	47	147.1	147.6	150.6	52.9	52.4	49.4	2.78	2.82	3.05	2.88	3.88E-07	0.028575957
P40429	RPL13A	12	127.8	125.7	127.2	72.2	74.3	72.8	1.77	1.69	1.75	1.74	4.35E-07	0.030790636
P42892	ECE1	12	161.1	162.8	158	38.9	37.2	42	4.14	4.38	3.76	4.09	4.32E-07	0.030790636
Q15185	PTGES3	7	139.4	137.3	140.4	60.6	62.7	59.6	2.30	2.19	2.36	2.28	4.49E-07	0.031045412
O43290	SART1	13	159.8	155.2	157	40.2	44.8	43	3.98	3.46	3.65	3.70	4.45E-07	0.031045412
O95340	PAPSS2	13	71.8	72.2	70	128.2	127.8	130	0.56	0.56	0.54	0.55	4.65E-07	0.031547814
Q16719	KYNU	17	12.8	11	5.7	187.2	189	194.3	0.07	0.06	0.03	0.05	4.67E-07	0.031547814
Q9BYX4	IFIH1	13	181.5	175.3	180.1	18.5	24.7	19.9	9.81	7.10	9.05	8.65	4.78E-07	0.03193465
P46939	UTRN	31	141.2	138.5	141.6	58.8	61.5	58.4	2.40	2.25	2.42	2.36	5.03E-07	0.033218694
Q8N357	SLC35F6	2	34.8	33.2	29.3	165.2	166.8	170.7	0.21	0.20	0.17	0.19	5.11E-07	0.033376272
O43491	EPB41L2	42	181.2	175	180.1	18.8	25	19.9	9.64	7.00	9.05	8.56	5.18E-07	0.033415757
P80723	BASP1	18	29.2	23.6	28.6	170.8	176.4	171.4	0.17	0.13	0.17	0.16	5.27E-07	0.033676103
P98088	MUC5AC	19	1.4	6.6	9.3	198.6	193.4	190.7	0.01	0.03	0.05	0.03	5.48E-07	0.034645499
P48506	GCLC	24	31.5	35.3	29.5	168.5	164.7	170.5	0.19	0.21	0.17	0.19	5.90E-07	0.036462193
P41223	BUD31	6	166.1	160.7	164.4	33.9	39.3	35.6	4.90	4.09	4.62	4.54	5.86E-07	0.036462193
P09525	ANXA4	35	42.4	39.3	44.4	157.6	160.7	155.6	0.27	0.24	0.29	0.27	6.42E-07	0.037017692
O43633	CHMP2A	11	75.1	73.9	76.1	124.9	126.1	123.9	0.60	0.59	0.61	0.60	6.30E-07	0.037017692
Q12860	CNTN1	41	13	7	14.4	187	193	185.6	0.07	0.04	0.08	0.06	6.46E-07	0.037017692
P78347	GTF2I	30	132.5	129.8	130.9	67.5	70.2	69.1	1.96	1.85	1.89	1.90	6.07E-07	0.037017692
O94776	MTA2	12	142.8	139.2	140.7	57.2	60.8	59.3	2.50	2.29	2.37	2.39	6.35E-07	0.037017692
Q9BZQ8	FAM129A	8	164.8	164.1	159.7	35.2	35.9	40.3	4.68	4.57	3.96	4.41	6.22E-07	0.037017692
Q96D71	REPS1	8	178.7	172.5	178	21.3	27.5	22	8.39	6.27	8.09	7.58	6.49E-07	0.037017692
P08240	SRPRA	11	189.4	181.9	185.2	10.6	18.1	14.8	17.87	10.05	12.51	13.48	6.21E-07	0.037017692
Q8WY22	BRI3BP	3	47.6	42.7	44.8	152.4	157.3	155.2	0.31	0.27	0.29	0.29	6.65E-07	0.037019103
P26358	DNMT1	46	155.8	151.5	151.9	44.2	48.5	48.1	3.52	3.12	3.16	3.27	6.68E-07	0.037019103
P17844	DDX5	24	148.5	147.9	152	51.5	52.1	48	2.88	2.84	3.17	2.96	6.68E-07	0.037019103
P51610	HCFC1	34	121.5	123.5	122.3	78.5	76.5	77.7	1.55	1.61	1.57	1.58	6.74E-07	0.037024449
P06756	ITGAV	23	53.5	57.5	55.1	146.5	142.5	144.9	0.37	0.40	0.38	0.38	6.88E-07	0.037453843
P00568	AK1	14	154.9	157.2	160.1	45.1	42.8	39.9	3.43	3.67	4.01	3.71	7.06E-07	0.037724878
Q9NUM4	TMEM106	5	24.9	31.4	27.5	175.1	168.6	172.5	0.14	0.19	0.16	0.16	7.06E-07	0.037724878
Q6UWE0	LRSAM1	4	156	160.8	156.5	44	39.2	43.5	3.55	4.10	3.60	3.75	7.24E-07	0.038162034
O14737	PDCD5	7	162.7	157.4	158.9	37.3	42.6	41.1	4.36	3.69	3.87	3.97	7.31E-07	0.038162034
Q5VYK3	ECM29	48	148.5	148.4	152.4	51.5	51.6	47.6	2.88	2.88	3.20	2.99	7.34E-07	0.038162034
Q15021	NCAPD2	29	149.2	146.7	151.3	50.8	53.3	48.7	2.94	2.75	3.11	2.93	8.07E-07	0.041581346
P35527	KRT9	25	197.6	194.3	188.8	2.4	5.7	11.2	82.33	34.09	16.86	44.43	8.47E-07	0.043105378
Q3KQU3	MAP7D1	14	173.3	171.2	178.1	26.7	28.8	21.9	6.49	5.94	8.13	6.86	8.58E-07	0.043105378

Q9NTZ6	RBM12	9	124.4	122.2	123.6	75.6	77.8	76.4	1.65	1.57	1.62	1.61	8.53E-07	0.043105378
Q9BXP5	SRRT	28	140.5	143.6	144.4	59.5	56.4	55.6	2.36	2.55	2.60	2.50	8.89E-07	0.044279586
P20591	MX1	11	10.6	2.2	9.1	189.4	197.8	190.9	0.06	0.01	0.05	0.04	9.07E-07	0.044681828
Q8ND24	RNF214	6	144.7	144.6	148.5	55.3	55.4	51.5	2.62	2.61	2.88	2.70	9.13E-07	0.044681828
P52272	HNRNPM	43	159.8	155.5	160.9	40.2	44.5	39.1	3.98	3.49	4.12	3.86	9.26E-07	0.044983802
P35659	DEK	9	138.7	138.4	141.9	61.3	61.6	58.1	2.26	2.25	2.44	2.32	9.51E-07	0.045797675
P35579	MYH9	94	175.5	169.3	175.6	24.5	30.7	24.4	7.16	5.51	7.20	6.62	9.68E-07	0.046230478
P49750	YLPM1	17	137.5	140.2	141.3	62.5	59.8	58.7	2.20	2.34	2.41	2.32	9.81E-07	0.046505268
O94822	LTN1	10	179.2	173	180	20.8	27	20	8.62	6.41	9.00	8.01	9.98E-07	0.04691489
Q7Z4S6	KIF21A	28	155.4	150.6	155	44.6	49.4	45	3.48	3.05	3.44	3.33	1.01E-06	0.047015187
P30838	ALDH3A1	26	18.4	9.9	13.1	181.6	190.1	186.9	0.10	0.05	0.07	0.07	1.02E-06	0.047308146
Q99943	AGPAT1	2	156.9	153.9	151.5	43.1	46.1	48.5	3.64	3.34	3.12	3.37	1.04E-06	0.047723382
Q92900	UPF1	43	163	157.3	162.1	37	42.7	37.9	4.41	3.68	4.28	4.12	1.07E-06	0.048844581
P05161	ISG15	5	188.5	187.8	180.6	11.5	12.2	19.4	16.39	15.39	9.31	13.70	1.13E-06	0.051082138
P27694	RPA1	17	146.2	142.1	142.6	53.8	57.9	57.4	2.72	2.45	2.48	2.55	1.15E-06	0.051477912
P46783	RPS10	11	124.1	123.9	126.2	75.9	76.1	73.8	1.64	1.63	1.71	1.66	1.17E-06	0.051697283
P46781	RPS9	20	121.2	121.3	123.2	78.8	78.7	76.8	1.54	1.54	1.60	1.56	1.17E-06	0.051697283
Q9UHY1	NRBP1	8	145.6	147.4	150.5	54.4	52.6	49.5	2.68	2.80	3.04	2.84	1.20E-06	0.051756645
P16989	YBX3	9	168.9	162.4	164.1	31.1	37.6	35.9	5.43	4.32	4.57	4.77	1.19E-06	0.051756645
Q86VM9	ZC3H18	12	164.6	158.5	160.1	35.4	41.5	39.9	4.65	3.82	4.01	4.16	1.20E-06	0.051756645
O60306	AQR	16	151.1	155.9	151.2	48.9	44.1	48.8	3.09	3.54	3.10	3.24	1.22E-06	0.051785744
O43379	WDR62	16	184.4	178.2	186.5	15.6	21.8	13.5	11.82	8.17	13.81	11.27	1.21E-06	0.051785744
Q8IUD2	ERC1	9	155.2	155.5	150.5	44.8	44.5	49.5	3.46	3.49	3.04	3.33	1.23E-06	0.052098398
P63313	TMSB10	3	145.3	140.8	143.6	54.7	59.2	56.4	2.66	2.38	2.55	2.53	1.27E-06	0.053215672
P14314	PRKCSH	17	144.1	140.8	145.2	55.9	59.2	54.8	2.58	2.38	2.65	2.54	1.29E-06	0.053819725
P52943	CRIP2	9	149.6	154.3	154.6	50.4	45.7	45.4	2.97	3.38	3.41	3.25	1.32E-06	0.054515085
O95155	UBE4B	21	177.8	181	186.4	22.2	19	13.6	8.01	9.53	13.71	10.41	1.33E-06	0.054515085
O75152	ZC3H11A	1	148.6	147.9	152.8	51.4	52.1	47.2	2.89	2.84	3.24	2.99	1.34E-06	0.054515085
P42345	MTOR	19	139.1	136.2	135.3	60.9	63.8	64.7	2.28	2.13	2.09	2.17	1.40E-06	0.056668111
Q9H4G0	EPB41L1	20	5.7	1	11.2	194.3	199	188.8	0.03	0.01	0.06	0.03	1.44E-06	0.05769102
P18206	VCL	83	150.5	146.2	151.1	49.5	53.8	48.9	3.04	2.72	3.09	2.95	1.44E-06	0.05769102
Q96G03	PGM2	8	171.2	174.2	166.5	28.8	25.8	33.5	5.94	6.75	4.97	5.89	1.51E-06	0.060117386
Q9UKV3	ACIN1	25	163.3	156.6	159.9	36.7	43.4	40.1	4.45	3.61	3.99	4.02	1.62E-06	0.063516488
P57088	TMEM33	5	146.4	143.3	148.4	53.6	56.7	51.6	2.73	2.53	2.88	2.71	1.61E-06	0.063516488
P24941	CDK2	7	141.1	137.6	141.8	58.9	62.4	58.2	2.40	2.21	2.44	2.35	1.64E-06	0.063655216
O60333	KIF1B	14	148.3	143.3	147	51.7	56.7	53	2.87	2.53	2.77	2.72	1.65E-06	0.063828207
P04792	HSPB1	18	66	65.5	62.3	134	134.5	137.7	0.49	0.49	0.45	0.48	1.72E-06	0.065958287
O75533	SF3B1	58	137.1	133.1	135.5	62.9	66.9	64.5	2.18	1.99	2.10	2.09	1.77E-06	0.067550569
Q8NCW5	NAXE	11	74.4	71.7	74.3	125.6	128.3	125.7	0.59	0.56	0.59	0.58	1.84E-06	0.06973948
Q66K74	MAP1S	10	152.6	153.5	158.5	47.4	46.5	41.5	3.22	3.30	3.82	3.45	1.87E-06	0.06999896
P00352	ALDH1A1	48	18	8.5	10.1	182	191.5	189.9	0.10	0.04	0.05	0.07	1.87E-06	0.06999896

P13987	CD59	3	125.4	128.2	125.7	74.6	71.8	74.3	1.68	1.79	1.69	1.72	1.90E-06	0.070642933
Q9UNI6	DUSP12	8	80.5	81	82.6	119.5	119	117.4	0.67	0.68	0.70	0.69	1.99E-06	0.072488899
O95163	IKBKAP	14	141.5	146.7	144.2	58.5	53.3	55.8	2.42	2.75	2.58	2.59	2.00E-06	0.072488899
P22033	MUT	7	134.5	130.8	133.7	65.5	69.2	66.3	2.05	1.89	2.02	1.99	2.01E-06	0.072488899
P52298	NCBP2	4	147	142	143.3	53	58	56.7	2.77	2.45	2.53	2.58	1.99E-06	0.072488899
Q9Y5S9	RBM8A	3	163.6	162.8	157	36.4	37.2	43	4.49	4.38	3.65	4.17	2.00E-06	0.072488899
P13645	KRT10	27	182.8	189	179.2	17.2	11	20.8	10.63	17.18	8.62	12.14	2.05E-06	0.073298714
P55196	AFDN	41	173	164.8	168.2	27	35.2	31.8	6.41	4.68	5.29	5.46	2.15E-06	0.076468729
P46379	BAG6	31	160.6	155.5	162.4	39.4	44.5	37.6	4.08	3.49	4.32	3.96	2.17E-06	0.076468729
Q9NQC3	RTN4	16	118.1	120.4	119.1	81.9	79.6	80.9	1.44	1.51	1.47	1.48	2.16E-06	0.076468729
P26447	S100A4	6	8.2	2.3	13.4	191.8	197.7	186.6	0.04	0.01	0.07	0.04	2.20E-06	0.076982424
Q9H2V7	SPNS1	2	60.2	61.7	64.7	139.8	138.3	135.3	0.43	0.45	0.48	0.45	2.24E-06	0.077912605
P08758	ANXA5	28	82.5	83.6	84.5	117.5	116.4	115.5	0.70	0.72	0.73	0.72	2.27E-06	0.078550564
Q7L014	DDX46	27	118.9	117.6	119.9	81.1	82.4	80.1	1.47	1.43	1.50	1.46	2.35E-06	0.079822643
O75044	SRGAP2	3	151.3	148.7	155	48.7	51.3	45	3.11	2.90	3.44	3.15	2.34E-06	0.079822643
Q96G46	DUS3L	10	176.6	179.7	170.6	23.4	20.3	29.4	7.55	8.85	5.80	7.40	2.32E-06	0.079822643
Q6P2Q9	PRPF8	76	146.1	141	145.2	53.9	59	54.8	2.71	2.39	2.65	2.58	2.41E-06	0.081359907
Q16658	FSCN1	33	144.5	150.3	148.4	55.5	49.7	51.6	2.60	3.02	2.88	2.83	2.44E-06	0.082052817
Q92823	NRCAM	13	8.9	4.3	15.5	191.1	195.7	184.5	0.05	0.02	0.08	0.05	2.49E-06	0.083206117
Q16531	DDB1	44	148.9	143.4	147.9	51.1	56.6	52.1	2.91	2.53	2.84	2.76	2.56E-06	0.085084715
P54727	RAD23B	11	139.6	134.9	137	60.4	65.1	63	2.31	2.07	2.17	2.19	2.67E-06	0.088203851
P52732	KIF11	7	164.4	162	170.1	35.6	38	29.9	4.62	4.26	5.69	4.86	2.70E-06	0.088663615
Q13523	PRPF4B	8	162.1	170.5	167.6	37.9	29.5	32.4	4.28	5.78	5.17	5.08	2.77E-06	0.090011776
P54578	USP14	23	149.2	143.6	148.3	50.8	56.4	51.7	2.94	2.55	2.87	2.78	2.77E-06	0.090011776
P11413	G6PD	52	24.3	14.1	17.3	175.7	185.9	182.7	0.14	0.08	0.09	0.10	2.79E-06	0.090202707
P52209	PGD	30	34.8	29.8	38.2	165.2	170.2	161.8	0.21	0.18	0.24	0.21	2.83E-06	0.090324537
P56945	BCAR1	14	178	168.6	174.3	22	31.4	25.7	8.09	5.37	6.78	6.75	2.84E-06	0.090324537
Q9Y450	HBS1L	14	168.4	160.8	167.8	31.6	39.2	32.2	5.33	4.10	5.21	4.88	2.84E-06	0.090324537
P50990	CCT8	47	144	140.1	145.5	56	59.9	54.5	2.57	2.34	2.67	2.53	2.88E-06	0.090845137
P23141	CES1	29	6.4	15.3	16.9	193.6	184.7	183.1	0.03	0.08	0.09	0.07	2.95E-06	0.092617732
Q8N3D4	EHBP1L1	20	143.1	139.4	144.8	56.9	60.6	55.2	2.51	2.30	2.62	2.48	2.97E-06	0.092934527
Q9NTJ3	SMC4	27	151.1	149.2	155.8	48.9	50.8	44.2	3.09	2.94	3.52	3.18	3.02E-06	0.093741845
Q9NWS8	RMND1	5	164.1	158.2	166.1	35.9	41.8	33.9	4.57	3.78	4.90	4.42	3.04E-06	0.093862183
Q9UQ80	PA2G4	29	139.4	139.3	144	60.6	60.7	56	2.30	2.29	2.57	2.39	3.08E-06	0.094794017
Q08211	DHX9	52	136.3	134	138.8	63.7	66	61.2	2.14	2.03	2.27	2.15	3.15E-06	0.095895896
P07900	HSP90AA1	47	155.6	149.3	155.2	44.4	50.7	44.8	3.50	2.94	3.46	3.30	3.17E-06	0.095895896
O95347	SMC2	36	159.3	154.3	161.9	40.7	45.7	38.1	3.91	3.38	4.25	3.85	3.15E-06	0.095895896
Q9UBU9	NXF1	7	135.6	131.4	132.4	64.4	68.6	67.6	2.11	1.92	1.96	1.99	3.19E-06	0.096072251
P11021	HSPA5	51	150.1	144.7	150.5	49.9	55.3	49.5	3.01	2.62	3.04	2.89	3.32E-06	0.09835574
P48960	CD97	6	169.3	161.6	169.4	30.7	38.4	30.6	5.51	4.21	5.54	5.09	3.35E-06	0.09835574
Q9Y4L1	HYOU1	50	142.8	139.3	144.9	57.2	60.7	55.1	2.50	2.29	2.63	2.47	3.31E-06	0.09835574

Q7Z5L9	IRF2BP2	6	69.7	68.5	72.4	130.3	131.5	127.6	0.53	0.52	0.57	0.54	3.35E-06	0.09835574
P10253	GAA	13	31.4	28.4	37.3	168.6	171.6	162.7	0.19	0.17	0.23	0.19	3.33E-06	0.09835574
P08174	CD55	5	14.4	24.7	16.2	185.6	175.3	183.8	0.08	0.14	0.09	0.10	3.43E-06	0.100301812
Q14204	DYNC1H1	167	143.4	138.6	143.6	56.6	61.4	56.4	2.53	2.26	2.55	2.45	3.47E-06	0.100376899
Q9NRX5	SERINC1	2	148.9	154.5	148.3	51.1	45.5	51.7	2.91	3.40	2.87	3.06	3.47E-06	0.100376899
Q16540	MRPL23	2	120.5	122.7	120	79.5	77.3	80	1.52	1.59	1.50	1.53	3.58E-06	0.102211011
P46063	RECQL	12	153.8	150.4	157.8	46.2	49.6	42.2	3.33	3.03	3.74	3.37	3.67E-06	0.102211011
O75976	CPD	29	64.1	63	67.6	135.9	137	132.4	0.47	0.46	0.51	0.48	3.64E-06	0.102211011
Q9H223	EHD4	11	159.4	155.6	151.8	40.6	44.4	48.2	3.93	3.50	3.15	3.53	3.62E-06	0.102211011
Q9Y262	EIF3L	12	133.2	129.5	133.4	66.8	70.5	66.6	1.99	1.84	2.00	1.94	3.66E-06	0.102211011
P08648	ITGA5	11	139	141.9	144.7	61	58.1	55.3	2.28	2.44	2.62	2.45	3.56E-06	0.102211011
P61326	MAGOH	8	167.5	161	159.4	32.5	39	40.6	5.15	4.13	3.93	4.40	3.65E-06	0.102211011
Q9HCE1	MOV10	12	186.3	175.5	183.3	13.7	24.5	16.7	13.60	7.16	10.98	10.58	3.59E-06	0.102211011
O43390	HNRNPR	23	148.2	143	149	51.8	57	51	2.86	2.51	2.92	2.76	3.91E-06	0.106986934
Q13438	OS9	6	154.7	147.8	153.1	45.3	52.2	46.9	3.42	2.83	3.26	3.17	3.90E-06	0.106986934
Q15428	SF3A2	9	144.4	139.3	144.5	55.6	60.7	55.5	2.60	2.29	2.60	2.50	3.89E-06	0.106986934
Q8TAQ2	SMARCC2	28	147	141.1	142.8	53	58.9	57.2	2.77	2.40	2.50	2.56	3.89E-06	0.106986934
O14979	HNRNPDL	9	131.4	132.8	128.5	68.6	67.2	71.5	1.92	1.98	1.80	1.90	4.21E-06	0.113989736
P61457	PCBD1	5	50.6	45.9	52.9	149.4	154.1	147.1	0.34	0.30	0.36	0.33	4.23E-06	0.113989736
Q99598	TSNAX	16	87.1	88.2	86.4	112.9	111.8	113.6	0.77	0.79	0.76	0.77	4.23E-06	0.113989736
Q8IWZ3	ANKHD1	9	130.5	134	135	69.5	66	65	1.88	2.03	2.08	1.99	4.27E-06	0.114212604
Q8IX12	CCAR1	28	147.7	141.7	146.8	52.3	58.3	53.2	2.82	2.43	2.76	2.67	4.28E-06	0.114212604
Q14157	UBAP2L	21	138.4	138.1	143.2	61.6	61.9	56.8	2.25	2.23	2.52	2.33	4.39E-06	0.116605639
Q86TU7	SETD3	5	179.5	185.6	191.8	20.5	14.4	8.2	8.76	12.89	23.39	15.01	4.41E-06	0.11664406
Q9Y2W1	THRAP3	11	162.5	156.1	164.5	37.5	43.9	35.5	4.33	3.56	4.63	4.17	4.43E-06	0.11664406
Q8N543	OGFOD1	3	138.4	140.3	134.9	61.6	59.7	65.1	2.25	2.35	2.07	2.22	4.54E-06	0.118492463
O95361	TRIM16	8	18.5	11.1	22.9	181.5	188.9	177.1	0.10	0.06	0.13	0.10	4.52E-06	0.118492463
Q6P1L8	MRPL14	2	121.8	123.2	125.2	78.2	76.8	74.8	1.56	1.60	1.67	1.61	4.71E-06	0.119823951
Q9Y5K6	CD2AP	19	151	156.8	149.6	49	43.2	50.4	3.08	3.63	2.97	3.23	4.64E-06	0.119823951
O43169	CYB5B	8	53.7	59.6	54.4	146.3	140.4	145.6	0.37	0.42	0.37	0.39	4.73E-06	0.119823951
Q14152	EIF3A	59	133.8	130.5	135.2	66.2	69.5	64.8	2.02	1.88	2.09	2.00	4.64E-06	0.119823951
Q16543	CDC37	28	170	161.1	162.8	30	38.9	37.2	5.67	4.14	4.38	4.73	4.73E-06	0.119823951
P42224	STAT1	25	5.6	12.4	18.4	194.4	187.6	181.6	0.03	0.07	0.10	0.07	4.68E-06	0.119823951
Q12874	SF3A3	12	140.7	136.4	135.6	59.3	63.6	64.4	2.37	2.14	2.11	2.21	4.71E-06	0.119823951
P35637	FUS	9	151	144.2	149.2	49	55.8	50.8	3.08	2.58	2.94	2.87	4.76E-06	0.11994167
Q92620	DHX38	17	156.4	165.2	162.5	43.6	34.8	37.5	3.59	4.75	4.33	4.22	4.83E-06	0.121159693
Q9UBC2	EPS15L1	9	131.1	135.4	131.4	68.9	64.6	68.6	1.90	2.10	1.92	1.97	4.85E-06	0.121322356
P14625	HSP90B1	63	142.6	137.1	141.9	57.4	62.9	58.1	2.48	2.18	2.44	2.37	4.93E-06	0.122761944
P48634	PRRC2A	21	165.3	175.6	169.4	34.7	24.4	30.6	4.76	7.20	5.54	5.83	4.96E-06	0.122981337
Q5PRF9	SAMD4B	9	165.4	171.8	162.1	34.6	28.2	37.9	4.78	6.09	4.28	5.05	5.03E-06	0.123698178
P17987	TCP1	29	146.4	143.8	150.7	53.6	56.2	49.3	2.73	2.56	3.06	2.78	5.02E-06	0.123698178

Q9UNF1	MAGED2	19	157.1	155	163.4	42.9	45	36.6	3.66	3.44	4.46	3.86	5.16E-06	0.12644768
O76094	SRP72	18	114.9	114.5	112.9	85.1	85.5	87.1	1.35	1.34	1.30	1.33	5.26E-06	0.128157186
O60216	RAD21	11	158.1	154.5	163.3	41.9	45.5	36.7	3.77	3.40	4.45	3.87	5.37E-06	0.129812515
Q9UHN6	TMEM2	11	26.6	19	14.7	173.4	181	185.3	0.15	0.10	0.08	0.11	5.36E-06	0.129812515
P84095	RHOG	7	73.2	73.8	69.9	126.8	126.2	130.1	0.58	0.58	0.54	0.57	5.47E-06	0.131738855
P30048	PRDX3	9	65.2	67.2	61.9	134.8	132.8	138.1	0.48	0.51	0.45	0.48	5.51E-06	0.13224641
Q7Z4V5	HDGFRP2	10	150.8	148	155.8	49.2	52	44.2	3.07	2.85	3.52	3.15	5.72E-06	0.136705347
O43242	PSMD3	15	126.5	123.6	127.4	73.5	76.4	72.6	1.72	1.62	1.75	1.70	5.78E-06	0.137523297
P63241	EIF5A	11	117.8	120.1	117.5	82.2	79.9	82.5	1.43	1.50	1.42	1.45	5.83E-06	0.137542783
Q13126	MTAP	13	199.7	186.6	198.8	0.3	13.4	1.2	665.67	13.93	165.67	281.75	5.82E-06	0.137542783
P29317	EPHA2	12	161.1	156.9	166.4	38.9	43.1	33.6	4.14	3.64	4.95	4.24	5.96E-06	0.138402783
P55060	CSE1L	36	135.8	137.3	141.5	64.2	62.7	58.5	2.12	2.19	2.42	2.24	5.93E-06	0.138402783
Q01844	EWSR1	7	128.1	125.7	124.1	71.9	74.3	75.9	1.78	1.69	1.64	1.70	5.98E-06	0.138402783
P11169	SLC2A3	5	168.7	158.9	163	31.3	41.1	37	5.39	3.87	4.41	4.55	5.96E-06	0.138402783
Q13586	STIM1	7	60.2	54.8	54	139.8	145.2	146	0.43	0.38	0.37	0.39	5.89E-06	0.138402783
Q8TF05	PPP4R1	8	161.5	154.5	163.3	38.5	45.5	36.7	4.19	3.40	4.45	4.01	6.06E-06	0.139658394
Q15643	TRIP11	31	154.1	149.6	158	45.9	50.4	42	3.36	2.97	3.76	3.36	6.12E-06	0.140560337
Q12906	ILF3	33	158.8	151.1	158.6	41.2	48.9	41.4	3.85	3.09	3.83	3.59	6.17E-06	0.140858266
P11498	PC	23	61.1	56.6	62.6	138.9	143.4	137.4	0.44	0.39	0.46	0.43	6.21E-06	0.140858266
Q9UGP8	SEC63	12	152.2	149.9	158	47.8	50.1	42	3.18	2.99	3.76	3.31	6.20E-06	0.140858266
Q9C0J8	WDR33	13	174.1	163.7	172.1	25.9	36.3	27.9	6.72	4.51	6.17	5.80	6.41E-06	0.144205715
O15498	YKT6	12	124.6	122	125.7	75.4	78	74.3	1.65	1.56	1.69	1.64	6.39E-06	0.144205715
Q99613	EIF3C	31	139.7	134	136	60.3	66	64	2.32	2.03	2.13	2.16	6.48E-06	0.144739866
P26038	MSN	52	138.6	133.5	138.5	61.4	66.5	61.5	2.26	2.01	2.25	2.17	6.48E-06	0.144739866
Q92945	KHSRP	32	137.5	132	135.4	62.5	68	64.6	2.20	1.94	2.10	2.08	6.57E-06	0.146223217
P25325	MPST	13	67.6	66.2	71.1	132.4	133.8	128.9	0.51	0.49	0.55	0.52	6.65E-06	0.146899885
Q9BUJ2	HNRNPUI	29	138	132.4	135.2	62	67.6	64.8	2.23	1.96	2.09	2.09	6.63E-06	0.146899885
Q96N67	DOCK7	13	164.3	157.9	167.9	35.7	42.1	32.1	4.60	3.75	5.23	4.53	6.75E-06	0.148651817
P83731	RPL24	12	123.7	121.3	125	76.3	78.7	75	1.62	1.54	1.67	1.61	6.93E-06	0.150878151
P27797	CALR	26	137.9	133.2	138.6	62.1	66.8	61.4	2.22	1.99	2.26	2.16	6.88E-06	0.150878151
P47813	EIF1AX	7	134	130.3	135.5	66	69.7	64.5	2.03	1.87	2.10	2.00	6.93E-06	0.150878151
P62750	RPL23A	15	127.3	123.7	127.4	72.7	76.3	72.6	1.75	1.62	1.75	1.71	7.00E-06	0.151862397
Q04917	YWHAH	19	91.1	90.6	92	108.9	109.4	108	0.84	0.83	0.85	0.84	7.10E-06	0.153307986
Q15942	ZYX	12	145.5	140.7	147.8	54.5	59.3	52.2	2.67	2.37	2.83	2.62	7.16E-06	0.154077081
Q92804	TAF15	6	119	117.8	120.9	81	82.2	79.1	1.47	1.43	1.53	1.48	7.22E-06	0.154785432
Q13310	PABPC4	18	162.4	158	168.2	37.6	42	31.8	4.32	3.76	5.29	4.46	7.26E-06	0.155034627
P08195	SLC3A2	22	43.4	33.7	35.9	156.6	166.3	164.1	0.28	0.20	0.22	0.23	7.33E-06	0.156058426
Q9UHQ4	BCAP29	7	143.6	141.5	148.6	56.4	58.5	51.4	2.55	2.42	2.89	2.62	7.42E-06	0.15686438
P49023	PXN	13	167.6	169.5	178.6	32.4	30.5	21.4	5.17	5.56	8.35	6.36	7.40E-06	0.15686438
Q9BTE3	MCMBP	13	159.6	151.1	158.6	40.4	48.9	41.4	3.95	3.09	3.83	3.62	7.60E-06	0.160007651
Q6L8Q7	PDE12	10	141.6	136.6	142.9	58.4	63.4	57.1	2.42	2.15	2.50	2.36	7.63E-06	0.160017392

P02751	FN1	17	34.5	23	30.5	165.5	177	169.5	0.21	0.13	0.18	0.17	7.71E-06	0.160547761
P46087	NOP2	15	162.9	154.4	155	37.1	45.6	45	4.39	3.39	3.44	3.74	7.70E-06	0.160547761
Q96RN5	MED15	2	152.7	145.8	145.8	47.3	54.2	54.2	3.23	2.69	2.69	2.87	7.78E-06	0.161583987
P83881	RPL36A	1	138.6	133.1	138.1	61.4	66.9	61.9	2.26	1.99	2.23	2.16	7.89E-06	0.161872855
O43432	EIF4G3	9	146.7	139.6	144.4	53.3	60.4	55.6	2.75	2.31	2.60	2.55	7.91E-06	0.161872855
Q9BS40	LXN	5	31.1	22.7	34	168.9	177.3	166	0.18	0.13	0.20	0.17	7.84E-06	0.161872855
Q9NX40	OCIAD1	14	151.2	144.4	151.7	48.8	55.6	48.3	3.10	2.60	3.14	2.95	7.87E-06	0.161872855
P33993	MCM7	30	147.4	140.1	144.2	52.6	59.9	55.8	2.80	2.34	2.58	2.58	7.98E-06	0.162540662
Q14C86	GAPVD1	21	157.7	164.3	154.7	42.3	35.7	45.3	3.73	4.60	3.42	3.92	7.99E-06	0.162540662
Q96I24	FUBP3	16	157.2	149.2	150.2	42.8	50.8	49.8	3.67	2.94	3.02	3.21	8.04E-06	0.162683989
Q15046	KARS	27	138.8	137.2	143.6	61.2	62.8	56.4	2.27	2.18	2.55	2.33	8.06E-06	0.162683989
P04062	GBA	9	63.1	56.7	57.8	136.9	143.3	142.2	0.46	0.40	0.41	0.42	8.18E-06	0.164109069
Q969H8	MYDGF	4	132.1	127.8	127.9	67.9	72.2	72.1	1.95	1.77	1.77	1.83	8.18E-06	0.164109069
P23786	CPT2	7	115	116	117.7	85	84	82.3	1.35	1.38	1.43	1.39	8.27E-06	0.164522096
Q16555	DPYSL2	25	55.1	48	54.7	144.9	152	145.3	0.38	0.32	0.38	0.36	8.29E-06	0.164522096
Q96TA1	FAM129B	26	138.8	135.4	141.9	61.2	64.6	58.1	2.27	2.10	2.44	2.27	8.24E-06	0.164522096
Q9BRA2	TXNDC17	4	66.5	60.7	61.6	133.5	139.3	138.4	0.50	0.44	0.45	0.46	8.32E-06	0.164522096
P47756	CAPZB	19	121.9	118.5	120.6	78.1	81.5	79.4	1.56	1.45	1.52	1.51	8.38E-06	0.165239848
O43707	ACTN4	48	140.2	134.9	140.9	59.8	65.1	59.1	2.34	2.07	2.38	2.27	8.57E-06	0.167421638
Q92598	HSPH1	42	136.5	132.9	139	63.5	67.1	61	2.15	1.98	2.28	2.14	8.58E-06	0.167421638
Q969E2	SCAMP4	2	118	115.3	117.5	82	84.7	82.5	1.44	1.36	1.42	1.41	8.56E-06	0.167421638
P11216	PYGB	47	69.7	64.5	68.7	130.3	135.5	131.3	0.53	0.48	0.52	0.51	8.73E-06	0.169889314
Q12792	TWF1	10	159.1	170.1	166.5	40.9	29.9	33.5	3.89	5.69	4.97	4.85	9.03E-06	0.175133044
Q9Y3B2	EXOSC1	5	147.5	140.7	147.4	52.5	59.3	52.6	2.81	2.37	2.80	2.66	9.14E-06	0.176613113
O95292	VAPB	12	73	70.2	74.9	127	129.8	125.1	0.57	0.54	0.60	0.57	9.30E-06	0.179133364
P53618	COPB1	20	119.7	122.2	123.4	80.3	77.8	76.6	1.49	1.57	1.61	1.56	9.35E-06	0.179521807
Q93052	LPP	17	61.4	59.1	65.6	138.6	140.9	134.4	0.44	0.42	0.49	0.45	9.39E-06	0.179665226
Q99832	CCT7	35	135.2	139.2	141.9	64.8	60.8	58.1	2.09	2.29	2.44	2.27	9.45E-06	0.180260705
Q05193	DNM1	6	23.8	17.2	30.5	176.2	182.8	169.5	0.14	0.09	0.18	0.14	9.60E-06	0.182611928
Q9H3P2	NELFA	5	173.8	187.9	180.3	26.2	12.1	19.7	6.63	15.53	9.15	10.44	9.68E-06	0.183488304
P62829	RPL23	8	137.2	133.6	140.1	62.8	66.4	59.9	2.18	2.01	2.34	2.18	9.95E-06	0.187958627
Q13283	G3BP1	14	136.8	140	143.9	63.2	60	56.1	2.16	2.33	2.57	2.35	1.01E-05	0.189836111
O00203	AP3B1	9	129.5	126.1	124.9	70.5	73.9	75.1	1.84	1.71	1.66	1.74	1.03E-05	0.193291649
P27816	MAP4	64	134.1	134.4	139.8	65.9	65.6	60.2	2.03	2.05	2.32	2.14	1.03E-05	0.193291649
Q9BSJ8	ESYT1	41	140.6	134.4	140	59.4	65.6	60	2.37	2.05	2.33	2.25	1.05E-05	0.195138746
Q96RT1	ERBIN	14	154.5	147.4	156.4	45.5	52.6	43.6	3.40	2.80	3.59	3.26	1.08E-05	0.20062819
P48643	CCT5	38	153.9	145	149.2	46.1	55	50.8	3.34	2.64	2.94	2.97	1.09E-05	0.202561979
P62191	PSMC1	16	158.4	150.1	159.3	41.6	49.9	40.7	3.81	3.01	3.91	3.58	1.12E-05	0.205053952
P55884	EIF3B	34	143.8	141.6	149.5	56.2	58.4	50.5	2.56	2.42	2.96	2.65	1.12E-05	0.205053952
Q00653	NFKB2	16	148.7	141	142.9	51.3	59	57.1	2.90	2.39	2.50	2.60	1.12E-05	0.205053952
Q13435	SF3B2	36	139.2	132.9	137.9	60.8	67.1	62.1	2.29	1.98	2.22	2.16	1.12E-05	0.205053952

Q9Y3F4	STRAP	19	143.6	138.4	136.7	56.4	61.6	63.3	2.55	2.25	2.16	2.32	1.13E-05	0.205360248
Q96DC9	OTUB2	1	132.4	128.1	133.5	67.6	71.9	66.5	1.96	1.78	2.01	1.92	1.14E-05	0.206679575
P18084	ITGB5	17	45	49.5	54.2	155	150.5	145.8	0.29	0.33	0.37	0.33	1.14E-05	0.207329934
Q9NS87	KIF15	3	141.4	149.1	143	58.6	50.9	57	2.41	2.93	2.51	2.62	1.15E-05	0.207525861
Q6ZUX7	LHFPL2	1	39.5	40	48.8	160.5	160	151.2	0.25	0.25	0.32	0.27	1.15E-05	0.207691125
P05198	EIF2S1	26	131.7	137.1	131.8	68.3	62.9	68.2	1.93	2.18	1.93	2.01	1.19E-05	0.213252581
Q53EP0	FNDC3B	2	81.9	79.3	78.3	118.1	120.7	121.7	0.69	0.66	0.64	0.66	1.19E-05	0.213252581
P15311	EZR	44	145.6	139.8	147.7	54.4	60.2	52.3	2.68	2.32	2.82	2.61	1.19E-05	0.213344399
P10620	MGST1	5	16	26.9	12.4	184	173.1	187.6	0.09	0.16	0.07	0.10	1.21E-05	0.215787618
Q14696	MESDC2	9	45.3	49.3	39.1	154.7	150.7	160.9	0.29	0.33	0.24	0.29	1.22E-05	0.21609183
Q96RK0	CIC	10	150.1	141.6	146.7	49.9	58.4	53.3	3.01	2.42	2.75	2.73	1.22E-05	0.21609183
Q9UHG3	PCYOX1	17	46.8	47	55	153.2	153	145	0.31	0.31	0.38	0.33	1.22E-05	0.21612108
P05787	KRT8	24	25.2	35.2	36.8	174.8	164.8	163.2	0.14	0.21	0.23	0.19	1.23E-05	0.217196551
P35908	KRT2	26	191.5	176	186.2	8.5	24	13.8	22.53	7.33	13.49	14.45	1.24E-05	0.218055154
P04040	CAT	13	62	67.6	67.5	138	132.4	132.5	0.45	0.51	0.51	0.49	1.26E-05	0.219957344
P35573	AGL	39	137.6	131.4	133.2	62.4	68.6	66.8	2.21	1.92	1.99	2.04	1.27E-05	0.220341365
Q96ST2	IWS1	12	126.3	122.5	122.4	73.7	77.5	77.6	1.71	1.58	1.58	1.62	1.27E-05	0.220341365
Q6VEQ5	WASH2P	3	144	138.3	137	56	61.7	63	2.57	2.24	2.17	2.33	1.27E-05	0.220341365
Q15003	NCAPH	3	143.8	146.1	138.3	56.2	53.9	61.7	2.56	2.71	2.24	2.50	1.28E-05	0.220702136
P05386	RPLP1	2	120.5	121	124.3	79.5	79	75.7	1.52	1.53	1.64	1.56	1.30E-05	0.223344494
O75400	PRPF40A	13	154.8	148.6	158.7	45.2	51.4	41.3	3.42	2.89	3.84	3.39	1.30E-05	0.223880679
Q9UN86	G3BP2	8	130.1	133.9	128.2	69.9	66.1	71.8	1.86	2.03	1.79	1.89	1.31E-05	0.224831953
O14976	GAK	8	172.5	162.8	161	27.5	37.2	39	6.27	4.38	4.13	4.93	1.32E-05	0.225125531
P63272	SUPT4H1	1	153	155.6	163.5	47	44.4	36.5	3.26	3.50	4.48	3.75	1.36E-05	0.232019016
Q07866	KLC1	14	153.3	144.5	152.1	46.7	55.5	47.9	3.28	2.60	3.18	3.02	1.37E-05	0.233120668
Q8WZA9	IRGQ	8	149.5	155.3	160	50.5	44.7	40	2.96	3.47	4.00	3.48	1.39E-05	0.234768071
Q99798	ACO2	29	119.7	123.8	121.2	80.3	76.2	78.8	1.49	1.62	1.54	1.55	1.41E-05	0.236743791
P14923	JUP	21	32.1	29	41.2	167.9	171	158.8	0.19	0.17	0.26	0.21	1.41E-05	0.236743791
O00469	PLOD2	17	51.8	46.7	56	148.2	153.3	144	0.35	0.30	0.39	0.35	1.40E-05	0.236743791
O76024	WFS1	4	161.7	159.5	171.1	38.3	40.5	28.9	4.22	3.94	5.92	4.69	1.41E-05	0.236743791
P35269	GTF2F1	9	151.2	144.9	142.6	48.8	55.1	57.4	3.10	2.63	2.48	2.74	1.42E-05	0.236832832
P53999	SUB1	11	132.6	127.8	127.6	67.4	72.2	72.4	1.97	1.77	1.76	1.83	1.43E-05	0.238143837
P49321	NASP	36	149.1	143.3	152.6	50.9	56.7	47.4	2.93	2.53	3.22	2.89	1.47E-05	0.244186838
Q14683	SMC1A	31	156	148.8	159.2	44	51.2	40.8	3.55	2.91	3.90	3.45	1.49E-05	0.246064136
P02545	LMNA	50	139.1	133.2	139.9	60.9	66.8	60.1	2.28	1.99	2.33	2.20	1.51E-05	0.248722598
P49368	CCT3	39	147	139	145.7	53	61	54.3	2.77	2.28	2.68	2.58	1.51E-05	0.248722598
O14617	AP3D1	8	145.2	138.8	147	54.8	61.2	53	2.65	2.27	2.77	2.56	1.56E-05	0.255331985
P13073	COX4I1	10	124.5	120.9	120.6	75.5	79.1	79.4	1.65	1.53	1.52	1.57	1.56E-05	0.255331985
O14974	PPP1R12A	30	169.6	157.1	163.3	30.4	42.9	36.7	5.58	3.66	4.45	4.56	1.56E-05	0.255331985
P60842	EIF4A1	18	126.6	124.5	129.8	73.4	75.5	70.2	1.72	1.65	1.85	1.74	1.58E-05	0.25643668
P56192	MARS	26	140.8	134.1	140.6	59.2	65.9	59.4	2.38	2.03	2.37	2.26	1.58E-05	0.25643668

Q10472	GALNT1	6	21.3	35.4	26.2	178.7	164.6	173.8	0.12	0.22	0.15	0.16	1.58E-05	0.25643668
P55072	VCP	80	127.3	124.1	129.4	72.7	75.9	70.6	1.75	1.64	1.83	1.74	1.59E-05	0.256622638
Q9NW82	WDR70	5	155.9	149.6	146	44.1	50.4	54	3.54	2.97	2.70	3.07	1.60E-05	0.257283943
Q9UHQ9	CYB5R1	10	67.2	72.7	71.7	132.8	127.3	128.3	0.51	0.57	0.56	0.55	1.61E-05	0.258622091
P49736	MCM2	29	146	138.2	139.8	54	61.8	60.2	2.70	2.24	2.32	2.42	1.63E-05	0.260496273
O14735	CDIPT	3	80.2	76.4	79.9	119.8	123.6	120.1	0.67	0.62	0.67	0.65	1.64E-05	0.261205858
Q92616	GCN1	108	127	122.8	127.5	73	77.2	72.5	1.74	1.59	1.76	1.70	1.66E-05	0.263902293
P38159	RBMX	19	154.2	161.1	166.3	45.8	38.9	33.7	3.37	4.14	4.93	4.15	1.67E-05	0.263902293
P62316	SNRPD2	9	141.7	147.2	138.8	58.3	52.8	61.2	2.43	2.79	2.27	2.50	1.66E-05	0.263902293
Q14694	USP10	9	156.2	150	161.2	43.8	50	38.8	3.57	3.00	4.15	3.57	1.68E-05	0.266097402
P16070	CD44	13	80.4	76.2	77	119.6	123.8	123	0.67	0.62	0.63	0.64	1.70E-05	0.266807139
Q14203	DCTN1	49	136.8	139.5	144.8	63.2	60.5	55.2	2.16	2.31	2.62	2.36	1.70E-05	0.266807139
Q9H6S3	EPS8L2	10	40.2	35.1	47	159.8	164.9	153	0.25	0.21	0.31	0.26	1.70E-05	0.266807139
O60869	EDF1	4	136.9	130.7	136.7	63.1	69.3	63.3	2.17	1.89	2.16	2.07	1.74E-05	0.271588422
Q8N8S7	ENAH	11	66.7	67.2	60.8	133.3	132.8	139.2	0.50	0.51	0.44	0.48	1.74E-05	0.271613332
P61978	HNRNPK	34	135.3	129.7	135.9	64.7	70.3	64.1	2.09	1.84	2.12	2.02	1.76E-05	0.273412345
O95394	PGM3	3	168.6	159.1	156.8	31.4	40.9	43.2	5.37	3.89	3.63	4.30	1.76E-05	0.273412345
P13639	EEF2	64	142.5	135.3	142.2	57.5	64.7	57.8	2.48	2.09	2.46	2.34	1.77E-05	0.273414106
Q01469	FABP5	15	163.1	155.9	168.6	36.9	44.1	31.4	4.42	3.54	5.37	4.44	1.77E-05	0.273414106
Q12888	TP53BP1	44	159	148.5	157.1	41	51.5	42.9	3.88	2.88	3.66	3.47	1.78E-05	0.27402883
Q9UBW8	COPS7A	10	146	137.6	143.7	54	62.4	56.3	2.70	2.21	2.55	2.49	1.80E-05	0.276080987
O75369	FLNB	145	67	61.1	67.5	133	138.9	132.5	0.50	0.44	0.51	0.48	1.80E-05	0.276080987
O60841	EIF5B	38	135.9	130.1	136.4	64.1	69.9	63.6	2.12	1.86	2.14	2.04	1.83E-05	0.278543698
Q9UBB6	NCDN	4	164.7	153	162.3	35.3	47	37.7	4.67	3.26	4.31	4.08	1.85E-05	0.281446306
P07237	P4HB	47	80.1	77.2	81.3	119.9	122.8	118.7	0.67	0.63	0.68	0.66	1.85E-05	0.281446306
Q5UIP0	RIF1	20	149.8	140.7	147.6	50.2	59.3	52.4	2.98	2.37	2.82	2.72	1.86E-05	0.282301166
Q9NVJ2	ARL8B	4	41.5	36.5	48.4	158.5	163.5	151.6	0.26	0.22	0.32	0.27	1.87E-05	0.282839714
Q9Y3D3	MRPS16	2	137.5	132.4	139.8	62.5	67.6	60.2	2.20	1.96	2.32	2.16	1.90E-05	0.285528458
Q6P1N0	CC2D1A	7	133.8	134.6	140.7	66.2	65.4	59.3	2.02	2.06	2.37	2.15	1.91E-05	0.285608145
Q13409	DYNC1I2	19	132.2	130.1	136.8	67.8	69.9	63.2	1.95	1.86	2.16	1.99	1.91E-05	0.285608145
Q709C8	VPS13C	26	48.7	50.1	57.9	151.3	149.9	142.1	0.32	0.33	0.41	0.35	1.91E-05	0.285608145
Q9BW30	TPPP3	4	12.6	28.7	16.5	187.4	171.3	183.5	0.07	0.17	0.09	0.11	1.93E-05	0.287682528
P62081	RPS7	18	121.7	117.6	120.1	78.3	82.4	79.9	1.55	1.43	1.50	1.49	1.95E-05	0.28952221
P24928	POLR2A	28	148.4	141.5	151	51.6	58.5	49	2.88	2.42	3.08	2.79	1.97E-05	0.28952221
Q6UB35	MTHFD1L	10	155.4	145.6	154.5	44.6	54.4	45.5	3.48	2.68	3.40	3.19	1.96E-05	0.28952221
Q96AY3	FKBP10	10	146.8	147.2	139	53.2	52.8	61	2.76	2.79	2.28	2.61	1.95E-05	0.28952221
Q9BZE9	ASPSCR1	7	128.9	124	124.5	71.1	76	75.5	1.81	1.63	1.65	1.70	1.96E-05	0.28952221
Q96JH7	VCPIP1	28	164.9	176	179.7	35.1	24	20.3	4.70	7.33	8.85	6.96	1.98E-05	0.290752067
Q7Z2Z2	EFL1	10	74.2	68.5	72.7	125.8	131.5	127.3	0.59	0.52	0.57	0.56	1.98E-05	0.290752067
Q5XKP0	MIC13	2	121.6	122.4	126.2	78.4	77.6	73.8	1.55	1.58	1.71	1.61	2.00E-05	0.292815564
O75822	EIF3J	12	154.9	145.7	146.3	45.1	54.3	53.7	3.43	2.68	2.72	2.95	2.01E-05	0.293066963

P08574	CYC1	10	120.6	122.1	117.9	79.4	77.9	82.1	1.52	1.57	1.44	1.51	2.03E-05	0.294365237
Q9Y6C9	MTCH2	10	92.8	92.8	91.4	107.2	107.2	108.6	0.87	0.87	0.84	0.86	2.03E-05	0.294365237
P14618	PKM	48	77.1	72.1	76	122.9	127.9	124	0.63	0.56	0.61	0.60	2.03E-05	0.294365237
P63172	DYNLT1	4	151.1	141.5	148.6	48.9	58.5	51.4	3.09	2.42	2.89	2.80	2.06E-05	0.297921783
Q13347	EIF3I	20	130.4	129.3	135.6	69.6	70.7	64.4	1.87	1.83	2.11	1.94	2.07E-05	0.298483585
P29144	TPP2	43	140.9	133.2	136	59.1	66.8	64	2.38	1.99	2.13	2.17	2.09E-05	0.300759505
Q04637	EIF4G1	48	136.3	130.5	137.4	63.7	69.5	62.6	2.14	1.88	2.19	2.07	2.14E-05	0.305346661
A6NHR9	SMCHD1	35	179.2	173.5	190.5	20.8	26.5	9.5	8.62	6.55	20.05	11.74	2.14E-05	0.305346661
Q14566	MCM6	26	139.1	132.2	138.9	60.9	67.8	61.1	2.28	1.95	2.27	2.17	2.15E-05	0.305741112
Q8WUI4	HDAC7	9	183.5	176.3	194.3	16.5	23.7	5.7	11.12	7.44	34.09	17.55	2.15E-05	0.305741112
Q9Y316	MEMO1	17	80	80.8	76.6	120	119.2	123.4	0.67	0.68	0.62	0.66	2.15E-05	0.305741112
Q16630	CPSF6	10	148.2	139.2	141.9	51.8	60.8	58.1	2.86	2.29	2.44	2.53	2.17E-05	0.307197633
P12270	TPR	102	133	127	132.4	67	73	67.6	1.99	1.74	1.96	1.89	2.18E-05	0.307984354
Q8IWJ2	GCC2	21	126.1	121.3	125.5	73.9	78.7	74.5	1.71	1.54	1.68	1.64	2.21E-05	0.311097094
Q9NZN4	EHD2	14	67.1	63.5	70.6	132.9	136.5	129.4	0.50	0.47	0.55	0.51	2.22E-05	0.312305166
O94874	UFL1	6	141.6	142.9	150.6	58.4	57.1	49.4	2.42	2.50	3.05	2.66	2.24E-05	0.314127365
Q8TEQ6	GEMIN5	15	139.8	132	136.2	60.2	68	63.8	2.32	1.94	2.13	2.13	2.27E-05	0.318252192
O75874	IDH1	27	27.7	32.6	41.8	172.3	167.4	158.2	0.16	0.19	0.26	0.21	2.28E-05	0.31838116
Q16881	TXNRD1	28	50.5	38.4	44.4	149.5	161.6	155.6	0.34	0.24	0.29	0.29	2.31E-05	0.32187443
P55786	NPEPPS	33	144.5	136.9	145.2	55.5	63.1	54.8	2.60	2.17	2.65	2.47	2.33E-05	0.323493559
Q9UII2	ATPIF1	1	138.1	132.6	140.5	61.9	67.4	59.5	2.23	1.97	2.36	2.19	2.34E-05	0.324842951
P16949	STMN1	10	165.9	161	175.5	34.1	39	24.5	4.87	4.13	7.16	5.39	2.35E-05	0.324842951
Q01780	EXOSC10	9	161.3	149.6	153.1	38.7	50.4	46.9	4.17	2.97	3.26	3.47	2.39E-05	0.327024266
P26599	PTBP1	24	138.9	144.6	148.5	61.1	55.4	51.5	2.27	2.61	2.88	2.59	2.38E-05	0.327024266
P49757	NUMB	4	151.6	142	150.6	48.4	58	49.4	3.13	2.45	3.05	2.88	2.39E-05	0.327024266
O95487	SEC24B	8	141.4	151.6	146.4	58.6	48.4	53.6	2.41	3.13	2.73	2.76	2.39E-05	0.327024266
P62314	SNRPD1	5	140.4	136.1	145	59.6	63.9	55	2.36	2.13	2.64	2.37	2.40E-05	0.327024266
Q15363	TMED2	5	90.1	88	87.9	109.9	112	112.1	0.82	0.79	0.78	0.80	2.37E-05	0.327024266
P52657	GTF2A2	1	167.8	178.8	163.9	32.2	21.2	36.1	5.21	8.43	4.54	6.06	2.42E-05	0.328908354
Q01433	AMPD2	11	142.2	134	136.8	57.8	66	63.2	2.46	2.03	2.16	2.22	2.47E-05	0.334065278
P53396	ACLY	50	67.8	64.5	71.6	132.2	135.5	128.4	0.51	0.48	0.56	0.52	2.49E-05	0.334065278
Q9NYF8	BCLAF1	13	157.5	149.6	146.5	42.5	50.4	53.5	3.71	2.97	2.74	3.14	2.48E-05	0.334065278
Q99459	CDC5L	7	171	156.8	164.2	29	43.2	35.8	5.90	3.63	4.59	4.70	2.49E-05	0.334065278
Q00839	HNRNPU	47	139.8	135.3	144.1	60.2	64.7	55.9	2.32	2.09	2.58	2.33	2.47E-05	0.334065278
Q8N1G4	LRRC47	10	130.3	137.4	132.1	69.7	62.6	67.9	1.87	2.19	1.95	2.00	2.49E-05	0.334065278
P40227	CCT6A	25	147.2	138	145.1	52.8	62	54.9	2.79	2.23	2.64	2.55	2.50E-05	0.334065278
P11142	HSPA8	34	130.4	124.4	128.7	69.6	75.6	71.3	1.87	1.65	1.81	1.77	2.51E-05	0.334568823
P85037	FOXK1	9	130.5	136.6	137.8	69.5	63.4	62.2	1.88	2.15	2.22	2.08	2.58E-05	0.34398649
P08238	HSP90AB1	41	143.1	135.8	144.3	56.9	64.2	55.7	2.51	2.12	2.59	2.41	2.59E-05	0.344088952
P07602	PSAP	13	73.5	66.9	70.9	126.5	133.1	129.1	0.58	0.50	0.55	0.54	2.63E-05	0.348506133
P08621	SNRNP70	15	139.1	132.2	139.7	60.9	67.8	60.3	2.28	1.95	2.32	2.18	2.65E-05	0.350127158

O60271	SPAG9	50	135.3	129.5	136.7	64.7	70.5	63.3	2.09	1.84	2.16	2.03	2.66E-05	0.350912778
Q96SB4	SRPK1	5	184.7	167.5	176.9	15.3	32.5	23.1	12.07	5.15	7.66	8.29	2.66E-05	0.350912778
Q9UBU6	FAM8A1	1	135.2	128.3	130.4	64.8	71.7	69.6	2.09	1.79	1.87	1.92	2.68E-05	0.352214217
P13807	GYS1	5	83.2	80.6	78.9	116.8	119.4	121.1	0.71	0.68	0.65	0.68	2.72E-05	0.355885049
Q6ZSR9	FLJ45252	6	177.9	167.8	162.4	22.1	32.2	37.6	8.05	5.21	4.32	5.86	2.72E-05	0.355885049
O00429	DNM1L	22	157.5	146.3	155.9	42.5	53.7	44.1	3.71	2.72	3.54	3.32	2.75E-05	0.359681878
P54136	RARS	36	135.6	129.3	136.3	64.4	70.7	63.7	2.11	1.83	2.14	2.02	2.80E-05	0.363525136
P19784	CSNK2A2	16	114	115.2	117.5	86	84.8	82.5	1.33	1.36	1.42	1.37	2.80E-05	0.363525136
Q9P2J5	LARS	51	149.7	139.6	146.6	50.3	60.4	53.4	2.98	2.31	2.75	2.68	2.80E-05	0.363525136
Q10567	AP1B1	13	140.3	138.5	147.6	59.7	61.5	52.4	2.35	2.25	2.82	2.47	2.81E-05	0.363845912
Q9BWD1	ACAT2	16	160.8	149.6	161.2	39.2	50.4	38.8	4.10	2.97	4.15	3.74	2.88E-05	0.372502297
P49792	RANBP2	41	125.1	127.1	131.4	74.9	72.9	68.6	1.67	1.74	1.92	1.78	2.92E-05	0.375227671
Q14498	RBM39	8	136.7	134.9	143.3	63.3	65.1	56.7	2.16	2.07	2.53	2.25	2.92E-05	0.375227671
Q9NNW5	WDR6	6	150.3	149.4	160.5	49.7	50.6	39.5	3.02	2.95	4.06	3.35	2.92E-05	0.375227671
Q9H4A4	RNPEP	23	57.2	49.1	58.8	142.8	150.9	141.2	0.40	0.33	0.42	0.38	2.94E-05	0.376038659
Q13200	PSMD2	34	144	137	146.5	56	63	53.5	2.57	2.17	2.74	2.49	2.96E-05	0.378070698
P04818	TYMS	10	159.3	146.9	154.1	40.7	53.1	45.9	3.91	2.77	3.36	3.35	3.03E-05	0.385927183
O00410	IPO5	38	137	129.8	136.5	63	70.2	63.5	2.17	1.85	2.15	2.06	3.05E-05	0.388032195
P00167	CYB5A	5	41.7	34.4	48.1	158.3	165.6	151.9	0.26	0.21	0.32	0.26	3.07E-05	0.388424829
P78527	PRKDC	167	134.8	131.3	139.5	65.2	68.7	60.5	2.07	1.91	2.31	2.09	3.06E-05	0.388424829
P61619	SEC61A1	5	141.4	139.8	149.3	58.6	60.2	50.7	2.41	2.32	2.94	2.56	3.07E-05	0.388424829
Q6VMQ6	ATF7IP	4	159.5	166.1	175.1	40.5	33.9	24.9	3.94	4.90	7.03	5.29	3.08E-05	0.388548535
Q9NRG0	CHRAC1	2	139.5	149.9	146.6	60.5	50.1	53.4	2.31	2.99	2.75	2.68	3.10E-05	0.389877022
Q9UM54	MYO6	27	159.2	174.7	165.5	40.8	25.3	34.5	3.90	6.91	4.80	5.20	3.11E-05	0.389877022
Q7LBC6	KDM3B	25	122.7	118.4	118.9	77.3	81.6	81.1	1.59	1.45	1.47	1.50	3.14E-05	0.392404218
Q9Y6A5	TACC3	16	182.2	165.3	171.2	17.8	34.7	28.8	10.24	4.76	5.94	6.98	3.15E-05	0.392404218
O14787	TNPO2	11	172.2	157	164.6	27.8	43	35.4	6.19	3.65	4.65	4.83	3.14E-05	0.392404218
Q6PIU2	NCEH1	13	147.5	138.2	146.9	52.5	61.8	53.1	2.81	2.24	2.77	2.60	3.16E-05	0.392861202
Q92538	GBF1	15	128.4	122.4	125.6	71.6	77.6	74.4	1.79	1.58	1.69	1.69	3.17E-05	0.393784468
Q9UMX0	UBQLN1	7	136.7	130.8	138.9	63.3	69.2	61.1	2.16	1.89	2.27	2.11	3.19E-05	0.395711078
P45974	USP5	31	134.2	128.1	135.2	65.8	71.9	64.8	2.04	1.78	2.09	1.97	3.21E-05	0.396944857
Q9UKG1	APPL1	8	146.7	139.7	137.3	53.3	60.3	62.7	2.75	2.32	2.19	2.42	3.23E-05	0.397887276
Q96RE7	NACC1	2	151.3	147.8	140.5	48.7	52.2	59.5	3.11	2.83	2.36	2.77	3.23E-05	0.397887276
Q16706	MAN2A1	10	116	120.2	119.2	84	79.8	80.8	1.38	1.51	1.48	1.45	3.27E-05	0.400992111
P20962	PTMS	4	132.2	138.1	140.8	67.8	61.9	59.2	1.95	2.23	2.38	2.19	3.26E-05	0.400992111
Q92733	PRCC	5	137.4	131.4	130	62.6	68.6	70	2.19	1.92	1.86	1.99	3.33E-05	0.407690051
Q00325	SLC25A3	15	120.1	116	119.5	79.9	84	80.5	1.50	1.38	1.48	1.46	3.34E-05	0.408394879
Q96HQ2	CDKN2AI	1	73.5	66.7	67.5	126.5	133.3	132.5	0.58	0.50	0.51	0.53	3.49E-05	0.425728798
Q9UPN3	MACF1	71	166.2	151.9	159.4	33.8	48.1	40.6	4.92	3.16	3.93	4.00	3.50E-05	0.42614389
P07910	HNRNPC	16	162	148.5	155.8	38	51.5	44.2	4.26	2.88	3.52	3.56	3.62E-05	0.439524296
O75083	WDR1	24	130.2	123.7	128.3	69.8	76.3	71.7	1.87	1.62	1.79	1.76	3.63E-05	0.439673961

O95757	HSPA4L	19	147.7	137.5	141.1	52.3	62.5	58.9	2.82	2.20	2.40	2.47	3.74E-05	0.44687208
Q9NQT8	KIF13B	18	61.9	54.3	63.5	138.1	145.7	136.5	0.45	0.37	0.47	0.43	3.70E-05	0.44687208
Q8NC51	SERBP1	13	147.7	137.4	144.9	52.3	62.6	55.1	2.82	2.19	2.63	2.55	3.74E-05	0.44687208
P51149	RAB7A	23	136.2	128.3	133.5	63.8	71.7	66.5	2.13	1.79	2.01	1.98	3.74E-05	0.44687208
P10599	TXN	7	59.7	51.8	61.6	140.3	148.2	138.4	0.43	0.35	0.45	0.41	3.73E-05	0.44687208
Q9BQ70	TCF25	2	147.9	153.3	161.1	52.1	46.7	38.9	2.84	3.28	4.14	3.42	3.71E-05	0.44687208
P22102	GART	41	143.8	136.6	146.7	56.2	63.4	53.3	2.56	2.15	2.75	2.49	3.72E-05	0.44687208
P30876	POLR2B	32	131.6	136.8	140.5	68.4	63.2	59.5	1.92	2.16	2.36	2.15	3.77E-05	0.449552982
Q68CP9	ARID2	6	184.5	194.7	173.9	15.5	5.3	26.1	11.90	36.74	6.66	18.43	3.79E-05	0.450241594
O60218	AKR1B10	22	22.4	35.4	17.5	177.6	164.6	182.5	0.13	0.22	0.10	0.15	3.81E-05	0.45118544
P04632	CAPNS1	13	88.1	87.4	85	111.9	112.6	115	0.79	0.78	0.74	0.77	3.81E-05	0.45118544
Q9Y639	NPTN	6	58.4	50.8	61.1	141.6	149.2	138.9	0.41	0.34	0.44	0.40	3.82E-05	0.45118544
P12814	ACTN1	42	143	133.9	141.5	57	66.1	58.5	2.51	2.03	2.42	2.32	3.83E-05	0.451543818
Q9UHD9	UBQLN2	6	147.6	145.2	156.9	52.4	54.8	43.1	2.82	2.65	3.64	3.04	3.85E-05	0.453884748
Q08380	LGALS3B	6	27.8	7.2	15.8	172.2	192.8	184.2	0.16	0.04	0.09	0.09	3.94E-05	0.460669481
Q5JRA6	MIA3	20	173.5	157.3	164.7	26.5	42.7	35.3	6.55	3.68	4.67	4.97	3.93E-05	0.460669481
O60664	PLIN3	26	173.1	156.9	165.4	26.9	43.1	34.6	6.43	3.64	4.78	4.95	3.93E-05	0.460669481
Q16513	PKN2	17	148.8	138.9	148.6	51.2	61.1	51.4	2.91	2.27	2.89	2.69	3.94E-05	0.460669481
P61964	WDR5	10	114.7	118.6	115.6	85.3	81.4	84.4	1.34	1.46	1.37	1.39	4.03E-05	0.470402093
P33176	KIF5B	49	124.2	118.8	122.3	75.8	81.2	77.7	1.64	1.46	1.57	1.56	4.11E-05	0.477924927
Q07812	BAX	9	58.6	48.8	58.5	141.4	151.2	141.5	0.41	0.32	0.41	0.38	4.12E-05	0.478369029
Q08379	GOLGA2	17	143.6	140.6	133.9	56.4	59.4	66.1	2.55	2.37	2.03	2.31	4.15E-05	0.480677959
Q86VP6	CAND1	36	125.8	120.2	124.8	74.2	79.8	75.2	1.70	1.51	1.66	1.62	4.20E-05	0.485783716
Q9BPX5	ARPC5L	5	162.4	170.6	154.7	37.6	29.4	45.3	4.32	5.80	3.42	4.51	4.27E-05	0.489803717
Q13011	ECH1	19	76.9	77.7	81.9	123.1	122.3	118.1	0.62	0.64	0.69	0.65	4.24E-05	0.489803717
Q05397	PTK2	4	155.5	157.9	170	44.5	42.1	30	3.49	3.75	5.67	4.30	4.28E-05	0.489803717
P22307	SCP2	14	67.6	74.4	68	132.4	125.6	132	0.51	0.59	0.52	0.54	4.28E-05	0.489803717
Q9Y3P9	RABGAP1	12	156.9	145.4	157.3	43.1	54.6	42.7	3.64	2.66	3.68	3.33	4.26E-05	0.489803717
Q9Y4E8	USP15	12	129.8	123.2	125.7	70.2	76.8	74.3	1.85	1.60	1.69	1.71	4.26E-05	0.489803717
Q15102	PAFAH1B	12	126.4	121.6	127.7	73.6	78.4	72.3	1.72	1.55	1.77	1.68	4.30E-05	0.490980998
Q8NE71	ABCF1	20	169.7	154.5	167.1	30.3	45.5	32.9	5.60	3.40	5.08	4.69	4.32E-05	0.492494779
O75947	ATP5H	10	118.4	115.1	119.4	81.6	84.9	80.6	1.45	1.36	1.48	1.43	4.34E-05	0.493334447
O15056	SYNJ2	13	142.1	147.7	154.4	57.9	52.3	45.6	2.45	2.82	3.39	2.89	4.41E-05	0.500197827
P18077	RPL35A	8	132.6	127.5	135.6	67.4	72.5	64.4	1.97	1.76	2.11	1.94	4.44E-05	0.503198419
Q8IZL8	PELP1	10	149.6	139.4	149.8	50.4	60.6	50.2	2.97	2.30	2.98	2.75	4.47E-05	0.503572847
P62306	SNRPF	1	135.7	127.7	133.7	64.3	72.3	66.3	2.11	1.77	2.02	1.96	4.48E-05	0.503572847
Q5BJF2	TMEM97	3	32.7	12.8	20.5	167.3	187.2	179.5	0.20	0.07	0.11	0.13	4.48E-05	0.503572847
Q92890	UFD1L	8	124	120.8	118.6	76	79.2	81.4	1.63	1.53	1.46	1.54	4.46E-05	0.503572847
P53801	PTTG1IP	2	131.6	140.9	137.8	68.4	59.1	62.2	1.92	2.38	2.22	2.17	4.50E-05	0.50489798
Q9NZI8	IGF2BP1	22	138.7	130.7	138.8	61.3	69.3	61.2	2.26	1.89	2.27	2.14	4.51E-05	0.505126758
O15397	IPO8	4	166.8	169.4	154.1	33.2	30.6	45.9	5.02	5.54	3.36	4.64	4.54E-05	0.507081558

P23381	WARS	25	148.1	137.4	141	51.9	62.6	59	2.85	2.19	2.39	2.48	4.55E-05	0.507081558
P68036	UBE2L3	9	140.9	132.3	140.7	59.1	67.7	59.3	2.38	1.95	2.37	2.24	4.57E-05	0.508675905
Q16181	7-Sep	17	127.7	124.3	131.5	72.3	75.7	68.5	1.77	1.64	1.92	1.78	4.59E-05	0.50963886
P52294	KPNA1	5	139.1	146.9	150.7	60.9	53.1	49.3	2.28	2.77	3.06	2.70	4.64E-05	0.511479469
P29966	MARCKS	9	129.9	125.4	133	70.1	74.6	67	1.85	1.68	1.99	1.84	4.65E-05	0.511479469
Q16186	ADRM1	4	132.7	131.6	139.9	67.3	68.4	60.1	1.97	1.92	2.33	2.07	4.64E-05	0.511479469
Q9H6T3	RPAP3	13	146.1	144.5	156.2	53.9	55.5	43.8	2.71	2.60	3.57	2.96	4.62E-05	0.511479469
Q9BQ52	ELAC2	22	117	114.4	118.7	83	85.6	81.3	1.41	1.34	1.46	1.40	4.63E-05	0.511479469
Q9NZ08	ERAP1	10	141.5	153	151.9	58.5	47	48.1	2.42	3.26	3.16	2.94	4.68E-05	0.513721859
Q14008	CKAP5	47	143.8	134.8	144.3	56.2	65.2	55.7	2.56	2.07	2.59	2.41	4.74E-05	0.51912124
Q04206	RELA	7	130.5	124.3	131	69.5	75.7	69	1.88	1.64	1.90	1.81	4.74E-05	0.51912124
A0AVT1	UBA6	23	140.6	133	142.6	59.4	67	57.4	2.37	1.99	2.48	2.28	4.78E-05	0.522243906
Q01201	RELB	4	159.1	149.7	146	40.9	50.3	54	3.89	2.98	2.70	3.19	4.80E-05	0.523148901
Q9UI26	IPO11	5	154.8	148.8	163.3	45.2	51.2	36.7	3.42	2.91	4.45	3.59	4.81E-05	0.523437815
P22314	UBA1	51	126.3	127.9	133.6	73.7	72.1	66.4	1.71	1.77	2.01	1.83	4.83E-05	0.524791091
Q5JPE7	NOMO2	2	120.5	117	122.1	79.5	83	77.9	1.52	1.41	1.57	1.50	4.86E-05	0.525757158
Q8TDY2	RB1CC1	11	155.8	172.6	166.9	44.2	27.4	33.1	3.52	6.30	5.04	4.96	4.85E-05	0.525757158
Q86Y56	DNAAF5	14	159	145.3	151.8	41	54.7	48.2	3.88	2.66	3.15	3.23	4.92E-05	0.527223557
P16144	ITGB4	22	48.5	51	60.2	151.5	149	139.8	0.32	0.34	0.43	0.36	4.93E-05	0.527223557
O60610	DIAPH1	18	144.7	139.5	134.3	55.3	60.5	65.7	2.62	2.31	2.04	2.32	4.91E-05	0.527223557
O75116	ROCK2	29	82.2	76.8	79.4	117.8	123.2	120.6	0.70	0.62	0.66	0.66	4.89E-05	0.527223557
Q9H2G2	SLK	31	131.6	125.3	132.5	68.4	74.7	67.5	1.92	1.68	1.96	1.85	4.91E-05	0.527223557
Q9Y6W5	WASF2	7	127.4	135.7	132.6	72.6	64.3	67.4	1.75	2.11	1.97	1.94	4.89E-05	0.527223557
P26639	TARS	39	129.5	122.8	128.1	70.5	77.2	71.9	1.84	1.59	1.78	1.74	4.94E-05	0.528192039
P46779	RPL28	11	129.4	123	129	70.6	77	71	1.83	1.60	1.82	1.75	4.98E-05	0.529240595
P06733	ENO1	41	118.8	123.3	124.3	81.2	76.7	75.7	1.46	1.61	1.64	1.57	5.02E-05	0.529240595
P33991	MCM4	36	136.8	135.3	144.9	63.2	64.7	55.1	2.16	2.09	2.63	2.30	5.03E-05	0.529240595
Q9BXW9	FANCD2	2	148.2	160.6	161.8	51.8	39.4	38.2	2.86	4.08	4.24	3.72	5.02E-05	0.529240595
P11940	PABPC1	12	137.4	130.3	139.2	62.6	69.7	60.8	2.19	1.87	2.29	2.12	4.97E-05	0.529240595
Q9P258	RCC2	22	143.9	134	142.2	56.1	66	57.8	2.57	2.03	2.46	2.35	5.00E-05	0.529240595
Q8IWW6	ARHGAP1	4	56.7	45.2	44.8	143.3	154.8	155.2	0.40	0.29	0.29	0.33	5.00E-05	0.529240595
O15294	OGT	17	144.9	135.6	145.5	55.1	64.4	54.5	2.63	2.11	2.67	2.47	4.99E-05	0.529240595
P18621	RPL17	13	132.8	125.1	129.5	67.2	74.9	70.5	1.98	1.67	1.84	1.83	5.05E-05	0.531172904
P40937	RFC5	15	133.7	131.3	140.3	66.3	68.7	59.7	2.02	1.91	2.35	2.09	5.08E-05	0.533037128
P62263	RPS14	11	128.5	123.3	122.4	71.5	76.7	77.6	1.80	1.61	1.58	1.66	5.13E-05	0.537824751
P53621	COPA	46	121.5	117.7	123.1	78.5	82.3	76.9	1.55	1.43	1.60	1.53	5.20E-05	0.542759945
Q9NQW7	XPNPEP1	23	139.8	130.4	135.8	60.2	69.6	64.2	2.32	1.87	2.12	2.10	5.19E-05	0.542759945
Q9UKF6	CPSF3	9	143.2	145.5	155.3	56.8	54.5	44.7	2.52	2.67	3.47	2.89	5.25E-05	0.546715069
Q09161	NCBP1	18	152.1	140.1	144.2	47.9	59.9	55.8	3.18	2.34	2.58	2.70	5.29E-05	0.550215411
Q641Q2	WASHC2A	12	132.4	129	137.8	67.6	71	62.2	1.96	1.82	2.22	2.00	5.30E-05	0.550215411
O43395	PRPF3	14	140.7	152.8	144.5	59.3	47.2	55.5	2.37	3.24	2.60	2.74	5.35E-05	0.554355617

P41250	GARS	35	142.9	134.3	144.5	57.1	65.7	55.5	2.50	2.04	2.60	2.38	5.46E-05	0.565009721
P10619	CTSA	7	136.4	143.9	134	63.6	56.1	66	2.14	2.57	2.03	2.25	5.51E-05	0.568291469
Q09666	AHNAK	325	134.1	126.6	133.9	65.9	73.4	66.1	2.03	1.72	2.03	1.93	5.51E-05	0.568291469
Q9GZP9	DERL2	1	59.2	64.5	68.9	140.8	135.5	131.1	0.42	0.48	0.53	0.47	5.53E-05	0.569339873
P30050	RPL12	6	127.5	130.8	123.4	72.5	69.2	76.6	1.76	1.89	1.61	1.75	5.61E-05	0.57598273
Q86UU0	BCL9L	5	126.3	120.1	124.8	73.7	79.9	75.2	1.71	1.50	1.66	1.63	5.63E-05	0.576761381
P41252	IARS	52	121.3	118	123.7	78.7	82	76.3	1.54	1.44	1.62	1.53	5.63E-05	0.576761381
Q5SW79	CEP170	9	158.1	154.4	170.3	41.9	45.6	29.7	3.77	3.39	5.73	4.30	5.68E-05	0.578939515
Q3ZCQ8	TIMM50	10	136.6	134.7	128	63.4	65.3	72	2.15	2.06	1.78	2.00	5.67E-05	0.578939515
Q9Y263	PLAA	13	138.3	132.4	129.3	61.7	67.6	70.7	2.24	1.96	1.83	2.01	5.78E-05	0.588342457
O43447	PPIH	6	147.2	157.5	162.1	52.8	42.5	37.9	2.79	3.71	4.28	3.59	5.79E-05	0.588369332
P50570	DNM2	6	120.6	124.4	127.2	79.4	75.6	72.8	1.52	1.65	1.75	1.64	5.86E-05	0.594513214
Q12905	ILF2	17	135.5	146.9	142.5	64.5	53.1	57.5	2.10	2.77	2.48	2.45	5.93E-05	0.600523498
Q9NRY5	FAM114A1	5	139.4	138.3	148.9	60.6	61.7	51.1	2.30	2.24	2.91	2.49	5.94E-05	0.600523498
P04844	RPN2	12	124.3	122.1	128.9	75.7	77.9	71.1	1.64	1.57	1.81	1.67	5.96E-05	0.601704152
P43246	MSH2	8	117.1	114	118.5	82.9	86	81.5	1.41	1.33	1.45	1.40	6.14E-05	0.617806972
Q96KG9	SCYL1	1	124.1	130.2	131.6	75.9	69.8	68.4	1.64	1.87	1.92	1.81	6.14E-05	0.617806972
O96005	CLPTM1	11	133.7	125.8	132.6	66.3	74.2	67.4	2.02	1.70	1.97	1.89	6.16E-05	0.618392733
Q96PK6	RBM14	7	159.3	156.6	173	40.7	43.4	27	3.91	3.61	6.41	4.64	6.20E-05	0.622073824
P62851	RPS25	10	116.2	118	121.3	83.8	82	78.7	1.39	1.44	1.54	1.46	6.23E-05	0.622770351
Q8NFJ5	GPRC5A	5	25	21.7	40.3	175	178.3	159.7	0.14	0.12	0.25	0.17	6.23E-05	0.622770351
P36915	GNL1	12	182.1	163.7	182.3	17.9	36.3	17.7	10.17	4.51	10.30	8.33	6.35E-05	0.632137898
Q9BUQ8	DDX23	9	153.3	140.3	149.7	46.7	59.7	50.3	3.28	2.35	2.98	2.87	6.36E-05	0.632137898
O14776	TCERG1	28	133.2	128.4	137.7	66.8	71.6	62.3	1.99	1.79	2.21	2.00	6.36E-05	0.632137898
O95202	LETM1	24	141.2	132.7	143	58.8	67.3	57	2.40	1.97	2.51	2.29	6.48E-05	0.642556187
Q8WXF1	PSPC1	6	155.3	141.7	151.2	44.7	58.3	48.8	3.47	2.43	3.10	3.00	6.48E-05	0.642556187
Q9HC38	GLOD4	18	87.7	84.3	87.5	112.3	115.7	112.5	0.78	0.73	0.78	0.76	6.50E-05	0.64330724
P31749	AKT1	5	160.4	150.4	167.1	39.6	49.6	32.9	4.05	3.03	5.08	4.05	6.58E-05	0.649420686
Q15029	EFTUD2	37	140.5	130.5	137.6	59.5	69.5	62.4	2.36	1.88	2.21	2.15	6.65E-05	0.653433764
P33992	MCM5	16	140.8	134	145.3	59.2	66	54.7	2.38	2.03	2.66	2.35	6.65E-05	0.653433764
Q6IN85	PPP4R3A	7	136.1	142.8	132.4	63.9	57.2	67.6	2.13	2.50	1.96	2.19	6.64E-05	0.653433764
Q9H9A6	LRRC40	3	165.2	150.2	164.8	34.8	49.8	35.2	4.75	3.02	4.68	4.15	6.68E-05	0.655428456
Q9UKD2	MRTO4	9	128.9	122.2	128.6	71.1	77.8	71.4	1.81	1.57	1.80	1.73	6.71E-05	0.657313695
P49454	CENPF	7	169.9	152.4	162.1	30.1	47.6	37.9	5.64	3.20	4.28	4.37	6.74E-05	0.659425084
O00625	PIR	11	69.5	74.9	66.5	130.5	125.1	133.5	0.53	0.60	0.50	0.54	6.88E-05	0.671057848
P35270	SPR	14	54.4	65.8	58	145.6	134.2	142	0.37	0.49	0.41	0.42	6.92E-05	0.673831203
O15027	SEC16A	14	149.2	142	156.1	50.8	58	43.9	2.94	2.45	3.56	2.98	6.93E-05	0.673835002
Q96EP5	DAZAP1	8	135.5	147.1	144.5	64.5	52.9	55.5	2.10	2.78	2.60	2.50	6.94E-05	0.674248037
P39023	RPL3	24	131.7	127.3	136.5	68.3	72.7	63.5	1.93	1.75	2.15	1.94	7.11E-05	0.689406828
Q14978	NOLC1	6	131.1	123.6	130.2	68.9	76.4	69.8	1.90	1.62	1.87	1.80	7.14E-05	0.691207508
P62910	RPL32	8	130.3	124.1	123.5	69.7	75.9	76.5	1.87	1.64	1.61	1.71	7.20E-05	0.695366453

P16435	POR	26	84	80.1	84.7	116	119.9	115.3	0.72	0.67	0.73	0.71	7.24E-05	0.69773058
Q5GLZ8	HERC4	8	142.8	138	150.6	57.2	62	49.4	2.50	2.23	3.05	2.59	7.24E-05	0.69773058
P51858	HDGF	10	76	78.2	82.1	124	121.8	117.9	0.61	0.64	0.70	0.65	7.30E-05	0.700396943
P67809	YBX1	11	163.2	147.2	154.7	36.8	52.8	45.3	4.43	2.79	3.42	3.55	7.29E-05	0.700396943
O96008	TOMM40	9	136.2	133	127	63.8	67	73	2.13	1.99	1.74	1.95	7.33E-05	0.701997462
O14936	CASK	5	62.6	50.4	58.7	137.4	149.6	141.3	0.46	0.34	0.42	0.40	7.34E-05	0.701997462
P62841	RPS15	4	128.9	123.5	131.5	71.1	76.5	68.5	1.81	1.61	1.92	1.78	7.38E-05	0.705136363
P09497	CLTB	6	124.1	118.3	123.6	75.9	81.7	76.4	1.64	1.45	1.62	1.57	7.42E-05	0.707316385
O43252	PAPSS1	8	134.8	125.9	131.5	65.2	74.1	68.5	2.07	1.70	1.92	1.90	7.48E-05	0.710000397
P08962	CD63	4	79.7	84.2	84	120.3	115.8	116	0.66	0.73	0.72	0.70	7.48E-05	0.710000397
Q9NS69	TOMM22	5	145.1	136.6	149.1	54.9	63.4	50.9	2.64	2.15	2.93	2.58	7.48E-05	0.710000397
O60762	DPM1	6	130.6	123.6	131	69.4	76.4	69	1.88	1.62	1.90	1.80	7.51E-05	0.710154747
P68371	TUBB4B	1	128.1	120.9	124.8	71.9	79.1	75.2	1.78	1.53	1.66	1.66	7.50E-05	0.710154747
Q03252	LMNB2	22	153.8	140	148.3	46.2	60	51.7	3.33	2.33	2.87	2.84	7.53E-05	0.711471018
P52630	STAT2	4	126.3	121.8	119.8	73.7	78.2	80.2	1.71	1.56	1.49	1.59	7.62E-05	0.718461958
O00151	PDLIM1	18	38.4	23.3	41.3	161.6	176.7	158.7	0.24	0.13	0.26	0.21	7.64E-05	0.718941489
P46060	RANGAP1	16	143.4	138.8	151.8	56.6	61.2	48.2	2.53	2.27	3.15	2.65	7.72E-05	0.725618817
P17931	LGALS3	10	59.1	60.6	48.7	140.9	139.4	151.3	0.42	0.43	0.32	0.39	7.75E-05	0.72762611
P68104	EEF1A1	20	141.1	131.2	140.6	58.9	68.8	59.4	2.40	1.91	2.37	2.22	7.85E-05	0.735268066
Q9UNN5	FAF1	7	142.8	133.8	145.3	57.2	66.2	54.7	2.50	2.02	2.66	2.39	7.98E-05	0.747091864
Q9NPB8	GPCPD1	4	36.5	32.8	16.5	163.5	167.2	183.5	0.22	0.20	0.09	0.17	8.02E-05	0.748670935
P60059	SEC61G	2	132.5	127	136.5	67.5	73	63.5	1.96	1.74	2.15	1.95	8.03E-05	0.748670935
P50991	CCT4	35	130	133.6	140.2	70	66.4	59.8	1.86	2.01	2.34	2.07	8.12E-05	0.755361113
Q15819	UBE2V2	4	139.3	131.9	143.1	60.7	68.1	56.9	2.29	1.94	2.51	2.25	8.12E-05	0.755361113
Q9H1C4	UNC93B1	3	37.4	29.6	16	162.6	170.4	184	0.23	0.17	0.09	0.16	8.17E-05	0.758511118
P80294	MT1H	1	183.9	169.8	162.6	16.1	30.2	37.4	11.42	5.62	4.35	7.13	8.29E-05	0.768292909
O14879	IFIT3	11	130.5	138.1	141.2	69.5	61.9	58.8	1.88	2.23	2.40	2.17	8.32E-05	0.770100439
Q9NUQ3	TXLNG	7	153	171.5	160.6	47	28.5	39.4	3.26	6.02	4.08	4.45	8.39E-05	0.774544047
P62979	RPS27A	10	137.4	148.8	149.6	62.6	51.2	50.4	2.19	2.91	2.97	2.69	8.40E-05	0.774544047
Q9BY44	EIF2A	11	137.7	128.1	131.3	62.3	71.9	68.7	2.21	1.78	1.91	1.97	8.45E-05	0.778625204
P49790	NUP153	18	156.1	175.2	172.1	43.9	24.8	27.9	3.56	7.06	6.17	5.60	8.49E-05	0.780731884
P49915	GMPS	31	141.5	134.3	146.6	58.5	65.7	53.4	2.42	2.04	2.75	2.40	8.55E-05	0.785239977
Q14839	CHD4	42	148.9	142.4	157.4	51.1	57.6	42.6	2.91	2.47	3.69	3.03	8.62E-05	0.790026396
Q9BT73	PSMG3	5	173.4	184.9	162.5	26.6	15.1	37.5	6.52	12.25	4.33	7.70	8.72E-05	0.797652935
O75368	SH3BGRL	5	68.9	58.2	65.4	131.1	141.8	134.6	0.53	0.41	0.49	0.47	8.73E-05	0.797652935
Q8IY81	FTSJ3	9	142.7	131.5	139.6	57.3	68.5	60.4	2.49	1.92	2.31	2.24	8.78E-05	0.800627257
P00374	DHFR	10	120.5	115.1	118.9	79.5	84.9	81.1	1.52	1.36	1.47	1.45	8.83E-05	0.804111934
Q96N66	MBOAT7	6	56.8	48.8	62.2	143.2	151.2	137.8	0.40	0.32	0.45	0.39	8.90E-05	0.809395371
P62195	PSMC5	19	134.6	125.9	128.1	65.4	74.1	71.9	2.06	1.70	1.78	1.85	8.94E-05	0.811849183
Q00534	CDK6	9	163.2	156.1	175.8	36.8	43.9	24.2	4.43	3.56	7.26	5.09	9.00E-05	0.815729788
O94966	USP19	11	135.5	135.4	145.8	64.5	64.6	54.2	2.10	2.10	2.69	2.30	9.04E-05	0.81874365

P61353	RPL27	8	131.1	123.1	125.6	68.9	76.9	74.4	1.90	1.60	1.69	1.73	9.10E-05	0.821293198
Q7L2E3	DHX30	7	137.9	127.8	132.8	62.1	72.2	67.2	2.22	1.77	1.98	1.99	9.09E-05	0.821293198
Q9NRF9	POLE3	4	144.1	138.7	152.5	55.9	61.3	47.5	2.58	2.26	3.21	2.68	9.18E-05	0.827077294
Q9UHX1	PUF60	12	134.6	131.8	142.6	65.4	68.2	57.4	2.06	1.93	2.48	2.16	9.19E-05	0.827201908
P21926	CD9	5	122.2	120.7	127.6	77.8	79.3	72.4	1.57	1.52	1.76	1.62	9.23E-05	0.82823153
P10515	DLAT	14	129	126	135.1	71	74	64.9	1.82	1.70	2.08	1.87	9.23E-05	0.82823153
O75886	STAM2	5	141.4	130.6	139.4	58.6	69.4	60.6	2.41	1.88	2.30	2.20	9.30E-05	0.833426629
P52907	CAPZA1	14	124.1	125.3	131.9	75.9	74.7	68.1	1.64	1.68	1.94	1.75	9.36E-05	0.837463968
P49411	TUFM	25	126.1	119.1	122.4	73.9	80.9	77.6	1.71	1.47	1.58	1.59	9.47E-05	0.845352256
P43897	TSFM	18	120.9	125.5	118.9	79.1	74.5	81.1	1.53	1.68	1.47	1.56	9.48E-05	0.845568248
Q9ULH1	ASAP1	5	129.6	137.7	140.3	70.4	62.3	59.7	1.84	2.21	2.35	2.13	9.51E-05	0.846067351
P20810	CAST	37	86.1	83.3	87.7	113.9	116.7	112.3	0.76	0.71	0.78	0.75	9.55E-05	0.846067351
P30043	BLVRB	10	81.6	75	76.4	118.4	125	123.6	0.69	0.60	0.62	0.64	9.54E-05	0.846067351
Q86TB9	PATL1	6	148.7	155.5	140.5	51.3	44.5	59.5	2.90	3.49	2.36	2.92	9.54E-05	0.846067351
Q86TI2	DPP9	17	158.8	145.1	145.6	41.2	54.9	54.4	3.85	2.64	2.68	3.06	9.59E-05	0.848346588
P09651	HNRNPA1	8	127.5	120.3	126.3	72.5	79.7	73.7	1.76	1.51	1.71	1.66	9.65E-05	0.851489367
Q13247	SRSF6	7	159.4	143.3	152.6	40.6	56.7	47.4	3.93	2.53	3.22	3.22	9.64E-05	0.851489367
P43490	NAMPT	30	25.5	39.2	44.8	174.5	160.8	155.2	0.15	0.24	0.29	0.23	9.70E-05	0.854381133
O43657	TSPAN6	4	62.5	68.5	72.5	137.5	131.5	127.5	0.45	0.52	0.57	0.51	9.72E-05	0.855410758
Q14651	PLS1	9	75.9	71.9	79.5	124.1	128.1	120.5	0.61	0.56	0.66	0.61	9.83E-05	0.862569596
Q96QE2	SLC2A13	1	163.4	150.7	147.4	36.6	49.3	52.6	4.46	3.06	2.80	3.44	9.84E-05	0.862569596
Q9NR30	DDX21	16	141.1	135.7	148.8	58.9	64.3	51.2	2.40	2.11	2.91	2.47	9.92E-05	0.868407173
Q13464	ROCK1	21	134.3	126.8	136.6	65.7	73.2	63.4	2.04	1.73	2.15	1.98	9.94E-05	0.8695515
P35268	RPL22	7	139.9	149.9	154.8	60.1	50.1	45.2	2.33	2.99	3.42	2.91	9.99E-05	0.872421311
P05091	ALDH2	27	32.8	10.4	10.3	167.2	189.6	189.7	0.20	0.05	0.05	0.10	0.00010041	0.875374114
Q9ULC4	MCTS1	10	126.9	119.8	122.1	73.1	80.2	77.9	1.74	1.49	1.57	1.60	0.00010095	0.878787844
P61981	YWHAG	17	86.5	90.1	87.2	113.5	109.9	112.8	0.76	0.82	0.77	0.79	0.00010151	0.882341882
P61254	RPL26	3	128.2	123	131.7	71.8	77	68.3	1.79	1.60	1.93	1.77	0.0001021	0.886075704
Q9UJS0	SLC25A13	14	131.3	133.3	141.9	68.7	66.7	58.1	1.91	2.00	2.44	2.12	0.0001027	0.886075704
Q16822	PCK2	12	73.6	73	80	126.4	127	120	0.58	0.57	0.67	0.61	0.00010251	0.886075704
Q8TCT8	SPPL2A	2	69.5	58.2	61	130.5	141.8	139	0.53	0.41	0.44	0.46	0.00010262	0.886075704
O75663	TIPRL	17	118.6	114.7	120.2	81.4	85.3	79.8	1.46	1.34	1.51	1.44	0.00010266	0.886075704
Q9BW60	ELOVL1	3	157.3	141.6	152.1	42.7	58.4	47.9	3.68	2.42	3.18	3.09	0.00010332	0.887587695
P42166	TMPO	5	125.5	119.7	126.9	74.5	80.3	73.1	1.68	1.49	1.74	1.64	0.00010319	0.887587695
Q6PJT7	ZC3H14	5	171.9	156	176.7	28.1	44	23.3	6.12	3.55	7.58	5.75	0.00010325	0.887587695
O43175	PHGDH	13	80.9	73.7	76	119.1	126.3	124	0.68	0.58	0.61	0.63	0.00010351	0.887853547
Q9H1B7	IRF2BPL	14	159.6	173	182.3	40.4	27	17.7	3.95	6.41	10.30	6.89	0.00010438	0.894043933
Q15075	EEA1	58	138.1	132.9	145.2	61.9	67.1	54.8	2.23	1.98	2.65	2.29	0.00010465	0.895026479
Q9H4M9	EHD1	19	146.6	134.2	138.2	53.4	65.8	61.8	2.75	2.04	2.24	2.34	0.00010501	0.896840356
Q93008	USP9X	37	117.7	115.7	112.8	82.3	84.3	87.2	1.43	1.37	1.29	1.37	0.00010614	0.90518739
P07711	CTSL	4	42.4	20.5	27.4	157.6	179.5	172.6	0.27	0.11	0.16	0.18	0.00010667	0.908368536

P00533	EGFR	30	72.6	62.8	69.9	127.4	137.2	130.1	0.57	0.46	0.54	0.52	0.00010713	0.910929084
Q9H2U1	DHX36	11	127.8	121	121.6	72.2	79	78.4	1.77	1.53	1.55	1.62	0.00010732	0.911229044
P61966	AP1S1	4	145.6	143.5	158.1	54.4	56.5	41.9	2.68	2.54	3.77	3.00	0.00010848	0.918877564
P34932	HSPA4	58	125.7	121.7	130	74.3	78.3	70	1.69	1.55	1.86	1.70	0.00010853	0.918877564
Q9H9B1	EHMT1	3	170	163.8	186.7	30	36.2	13.3	5.67	4.52	14.04	8.08	0.00010923	0.923431761
Q03001	DST	22	167.9	151.9	151.9	32.1	48.1	48.1	5.23	3.16	3.16	3.85	0.00010991	0.927851881
Q9Y676	MRPS18B	6	121.2	129.3	124.8	78.8	70.7	75.2	1.54	1.83	1.66	1.68	0.00011067	0.932959213
O00571	DDX3X	28	137.2	126.8	132.7	62.8	73.2	67.3	2.18	1.73	1.97	1.96	0.00011101	0.934441334
Q9NRN7	AASDHPP	8	136.6	136.4	147.8	63.4	63.6	52.2	2.15	2.14	2.83	2.38	0.00011163	0.938348438
P05204	HMGN2	4	154.5	139.7	151.9	45.5	60.3	48.1	3.40	2.32	3.16	2.96	0.00011222	0.941948878
Q9HAB8	PPCS	9	77.7	73	69	122.3	127	131	0.64	0.57	0.53	0.58	0.00011341	0.950566829
P22681	CBL	9	171.2	151.6	166.9	28.8	48.4	33.1	5.94	3.13	5.04	4.71	0.00011402	0.954364526
Q8IY67	RAVER1	6	148.8	143.6	159.8	51.2	56.4	40.2	2.91	2.55	3.98	3.14	0.00011435	0.954400719
Q9UJA5	TRMT6	9	133	125.7	124.7	67	74.3	75.3	1.99	1.69	1.66	1.78	0.00011422	0.954400719
P11766	ADH5	14	139.2	136.6	149.5	60.8	63.4	50.5	2.29	2.15	2.96	2.47	0.00011521	0.960183551
O15371	EIF3D	19	131.8	122.9	127	68.2	77.1	73	1.93	1.59	1.74	1.76	0.00011584	0.964125468
P62304	SNRPE	3	133.8	125.2	134.2	66.2	74.8	65.8	2.02	1.67	2.04	1.91	0.00011611	0.965010895
Q9H3H3	C11orf68	4	151.9	138.8	152.8	48.1	61.2	47.2	3.16	2.27	3.24	2.89	0.00011654	0.96716749
O14964	HGS	13	138.1	148.1	135.5	61.9	51.9	64.5	2.23	2.85	2.10	2.40	0.000117	0.969653032
O75150	RNF40	20	129	120.8	125.1	71	79.2	74.9	1.82	1.53	1.67	1.67	0.00011785	0.974989543
Q6P2E9	EDC4	16	139.8	129.9	140.9	60.2	70.1	59.1	2.32	1.85	2.38	2.19	0.00011798	0.974989543
P35580	MYH10	12	185	161	177	15	39	23	12.33	4.13	7.70	8.05	0.00011817	0.975175413
P62937	PPIA	12	130.1	122.3	130.2	69.9	77.7	69.8	1.86	1.57	1.87	1.77	0.00011879	0.978293105
Q13144	EIF2B5	6	143.8	133.3	145.9	56.2	66.7	54.1	2.56	2.00	2.70	2.42	0.00011888	0.978293105
P42677	RPS27	3	126.2	136.3	130.1	73.8	63.7	69.9	1.71	2.14	1.86	1.90	0.00011993	0.985538826
P23246	SFPQ	18	138.6	128	131.5	61.4	72	68.5	2.26	1.78	1.92	1.98	0.00012036	0.987716549
P07942	LAMB1	22	78.4	84.5	81.7	121.6	115.5	118.3	0.64	0.73	0.69	0.69	0.00012088	0.989615531
P40925	MDH1	20	71.2	79.3	75.8	128.8	120.7	124.2	0.55	0.66	0.61	0.61	0.00012093	0.989615531
P21589	NT5E	14	155.4	141.5	156.9	44.6	58.5	43.1	3.48	2.42	3.64	3.18	0.0001217	0.994562216
Q9C0C9	UBE2O	15	91.6	86.8	91.6	108.4	113.2	108.4	0.85	0.77	0.85	0.82	0.00090446	1
Q9BV57	ADI1	6	96.5	68.1	98.9	103.5	131.9	101.1	0.93	0.52	0.98	0.81	0.15691233	1
Q04446	GBE1	24	78.9	69.6	73.3	121.1	130.4	126.7	0.65	0.53	0.58	0.59	0.00016746	1
P61604	HSPE1	11	89.4	87.4	92.2	110.6	112.6	107.8	0.81	0.78	0.86	0.81	0.00046549	1
Q9NRX4	PHPT1	1	109.5	102.2	112.9	90.5	97.8	87.1	1.21	1.04	1.30	1.18	0.02131804	1
P31946	YWHAB	15	107.4	91	105.7	92.6	109	94.3	1.16	0.83	1.12	1.04	0.72929966	1
P62258	YWHAE	25	90.4	95	92	109.6	105	108	0.82	0.90	0.85	0.86	0.00138746	1
P31947	SFN	17	3.2	93.2	5	196.8	106.8	195	0.02	0.87	0.03	0.30	0.03445641	1
P27348	YWHAQ	22	72.7	88.4	72.5	127.3	111.6	127.5	0.57	0.79	0.57	0.64	0.00402086	1
P63104	YWHAZ	25	120.4	98.8	119.2	79.6	101.2	80.8	1.51	0.98	1.48	1.32	0.06114454	1
P15428	HPGD	10	20.7	116.7	24.2	179.3	83.3	175.8	0.12	1.40	0.14	0.55	0.10655134	1
Q9C0C2	TNKS1BP	52	77.5	71.7	81.2	122.5	128.3	118.8	0.63	0.56	0.68	0.63	0.00028868	1

Q01970	PLCB3	15	113.2	121.2	127	86.8	78.8	73	1.30	1.54	1.74	1.53	0.00193643		1
Q4KWH8	PLCH1	1	160.5	142	144.7	39.5	58	55.3	4.06	2.45	2.62	3.04	0.00027401		1
P19174	PLCG1	18	135.3	121.7	138.9	64.7	78.3	61.1	2.09	1.55	2.27	1.97	0.00099059		1
Q16698	DECR1	15	117.2	95.3	119	82.8	104.7	81	1.42	0.91	1.47	1.26	0.12305644		1
P17980	PSMC3	23	144.1	125	122.7	55.9	75	77.3	2.58	1.67	1.59	1.94	0.00309606		1
P43686	PSMC4	23	165.4	131.6	141.2	34.6	68.4	58.8	4.78	1.92	2.40	3.04	0.00292577		1
Q99460	PSMD1	32	134.8	124.4	135.5	65.2	75.6	64.5	2.07	1.65	2.10	1.94	0.00024021		1
O00487	PSMD14	15	111	103.8	106.6	89	96.2	93.4	1.25	1.08	1.14	1.16	0.00855935		1
P55036	PSMD4	14	89.3	109.8	114.8	110.7	90.2	85.2	0.81	1.22	1.35	1.12	0.44822315		1
P51665	PSMD7	10	106.9	106.8	112.3	93.1	93.2	87.7	1.15	1.15	1.28	1.19	0.00251718		1
P48556	PSMD8	11	109.2	106.3	114.2	90.8	93.7	85.8	1.20	1.13	1.33	1.22	0.00372489		1
O00233	PSMD9	6	115.9	102.2	119.4	84.1	97.8	80.6	1.38	1.04	1.48	1.30	0.0280867		1
Q13442	PDAP1	10	142.7	114.2	131.9	57.3	85.8	68.1	2.49	1.33	1.94	1.92	0.00728682		1
P82664	MRPS10	2	126.2	102.9	119.2	73.8	97.1	80.8	1.71	1.06	1.48	1.42	0.02997056		1
O60783	MRPS14	2	121.9	121.1	107.6	78.1	78.9	92.4	1.56	1.53	1.16	1.42	0.00678301		1
Q9Y399	MRPS2	7	108.8	110.6	118.2	91.2	89.4	81.8	1.19	1.24	1.44	1.29	0.00353917		1
P82921	MRPS21	1	128.9	116.4	128.2	71.1	83.6	71.8	1.81	1.39	1.79	1.66	0.00102981		1
P82663	MRPS25	5	127.5	123.3	143.3	72.5	76.7	56.7	1.76	1.61	2.53	1.96	0.00188634		1
Q9Y2Q9	MRPS28	4	111.6	141.4	161.4	88.4	58.6	38.6	1.26	2.41	4.18	2.62	0.02034441		1
P51398	DAP3	4	115.3	128.5	142.2	84.7	71.5	57.8	1.36	1.80	2.46	1.87	0.00642562		1
Q92665	MRPS31	5	117.8	86.7	75.4	82.2	113.3	124.6	1.43	0.77	0.61	0.93	0.49634173		1
P82930	MRPS34	10	112.8	104.6	106.3	87.2	95.4	93.7	1.29	1.10	1.13	1.17	0.01106361		1
P82673	MRPS35	7	134.5	101.5	65.5	65.5	98.5	134.5	2.05	1.03	0.49	1.19	0.97339048		1
P82909	MRPS36	4	112.2	71.4	129.8	87.8	128.6	70.2	1.28	0.56	1.85	1.23	0.73344581		1
Q9Y2R9	MRPS7	13	109.5	111.9	76.4	90.5	88.1	123.6	1.21	1.27	0.62	1.03	0.9322096		1
O43598	DNPH1	7	123.7	99.2	118.7	76.3	100.8	81.3	1.62	0.98	1.46	1.36	0.05856492		1
Q5HYK3	COQ5	5	125.6	86.1	73.6	74.4	113.9	126.4	1.69	0.76	0.58	1.01	0.68120649		1
Q02218	OGDH	32	133.8	126.8	137.6	66.2	73.2	62.4	2.02	1.73	2.21	1.99	0.0001268		1
P21953	BCKDHB	5	124	97.9	128.3	76	102.1	71.7	1.63	0.96	1.79	1.46	0.06737679		1
O95861	BPNT1	8	143.4	87.7	113.9	56.6	112.3	86.1	2.53	0.78	1.32	1.55	0.25775585		1
Q9BYD6	MRPL1	14	109.9	99.2	116.4	90.1	100.8	83.6	1.22	0.98	1.39	1.20	0.07457439		1
Q7Z7H8	MRPL10	6	80.5	113.2	79.2	119.5	86.8	120.8	0.67	1.30	0.66	0.88	0.31476316		1
Q9Y3B7	MRPL11	7	108.2	110.6	135.7	91.8	89.4	64.3	1.18	1.24	2.11	1.51	0.04317932		1
P52815	MRPL12	5	92.8	80.1	78.9	107.2	119.9	121.1	0.87	0.67	0.65	0.73	0.00693599		1
Q9BYD1	MRPL13	8	109.5	100.8	120.5	90.5	99.2	79.5	1.21	1.02	1.52	1.25	0.06348527		1
Q9P015	MRPL15	10	95.4	111.1	82.8	104.6	88.9	117.2	0.91	1.25	0.71	0.96	0.57110658		1
Q9NX20	MRPL16	7	75.8	107.8	113.6	124.2	92.2	86.4	0.61	1.17	1.31	1.03	0.91599252		1
P49406	MRPL19	11	94.2	95.1	79.4	105.8	104.9	120.6	0.89	0.91	0.66	0.82	0.04416967		1
Q7Z2W9	MRPL21	4	110.7	118.3	77.4	89.3	81.7	122.6	1.24	1.45	0.63	1.11	0.82197547		1
Q9NWU5	MRPL22	7	106.8	90.3	93	93.2	109.7	107	1.15	0.82	0.87	0.95	0.41274207		1
Q96A35	MRPL24	10	88.7	93.3	87.3	111.3	106.7	112.7	0.80	0.87	0.77	0.82	0.00133307		1

Q9P0M9	MRPL27	3	118.6	109.2	117.2	81.4	90.8	82.8	1.46	1.20	1.42	1.36	0.00192657		1
Q13084	MRPL28	10	103.2	100.2	103.4	96.8	99.8	96.6	1.07	1.00	1.07	1.05	0.03631555		1
P09001	MRPL3	8	73.2	95.6	94.1	126.8	104.4	105.9	0.58	0.92	0.89	0.79	0.07283531		1
Q9BYC8	MRPL32	2	110	109.7	120.2	90	90.3	79.8	1.22	1.21	1.51	1.31	0.00550584		1
Q9NYK5	MRPL39	13	110.3	96	97.6	89.7	104	102.4	1.23	0.92	0.95	1.04	0.70524185		1
Q9BYD3	MRPL4	9	98.7	82.8	108.1	101.3	117.2	91.9	0.97	0.71	1.18	0.95	0.54300531		1
Q9NQ50	MRPL40	4	111.6	92.4	104.8	88.4	107.6	95.2	1.26	0.86	1.10	1.07	0.50143369		1
Q8IXM3	MRPL41	5	116.8	95.5	164.4	83.2	104.5	35.6	1.40	0.91	4.62	2.31	0.15051344		1
Q8N983	MRPL43	4	109.9	101.2	125.5	90.1	98.8	74.5	1.22	1.02	1.68	1.31	0.07220133		1
Q9H9J2	MRPL44	14	88.8	101	93.4	111.2	99	106.6	0.80	1.02	0.88	0.90	0.08997132		1
Q9BRJ2	MRPL45	8	79.3	100.5	89	120.7	99.5	111	0.66	1.01	0.80	0.82	0.07432483		1
Q9H2W6	MRPL46	8	61.8	83	55.4	138.2	117	144.6	0.45	0.71	0.38	0.51	0.00486295		1
Q9HD33	MRPL47	6	122.4	133	100	77.6	67	100	1.58	1.99	1.00	1.52	0.05495205		1
Q96GC5	MRPL48	5	103.5	110.9	112.7	96.5	89.1	87.3	1.07	1.24	1.29	1.20	0.01051192		1
Q4U2R6	MRPL51	1	88.6	92.5	120	111.4	107.5	80	0.80	0.86	1.50	1.05	0.96066338		1
Q96EL3	MRPL53	1	81.7	55.6	68.3	118.3	144.4	131.7	0.69	0.39	0.52	0.53	0.00411459		1
Q9BYD2	MRPL9	8	110.9	118.1	135.3	89.1	81.9	64.7	1.24	1.44	2.09	1.59	0.0138321		1
Q9NVS2	MRPS18A	5	111.7	105.5	114.2	88.3	94.5	85.8	1.27	1.12	1.33	1.24	0.00461146		1
Q15125	EBP	1	84.6	101.8	105.9	115.4	98.2	94.1	0.73	1.04	1.13	0.97	0.60765603		1
Q99714	HSD17B1(	16	99.2	85.2	95	100.8	114.8	105	0.98	0.74	0.90	0.88	0.07927324		1
Q9BUT1	BDH2	3	99.9	75.7	97.5	100.1	124.3	102.5	1.00	0.61	0.95	0.85	0.17484124		1
P31937	HIBADH	13	35.9	53.5	48.6	164.1	146.5	151.4	0.22	0.37	0.32	0.30	0.00012935		1
P42765	ACAA2	13	74.7	92.2	93.4	125.3	107.8	106.6	0.60	0.86	0.88	0.78	0.03633393		1
P09110	ACAA1	5	40.7	97.4	78.1	159.3	102.6	121.9	0.26	0.95	0.64	0.62	0.07652753		1
Q06136	KDSR	3	136.8	88.2	139.3	63.2	111.8	60.7	2.16	0.79	2.29	1.75	0.14247386		1
P62280	RPS11	13	121	86.6	119.5	79	113.4	80.5	1.53	0.76	1.48	1.26	0.31862423		1
P25398	RPS12	7	125.8	104.4	125.8	74.2	95.6	74.2	1.70	1.09	1.70	1.49	0.02082184		1
P62277	RPS13	12	123.4	113.4	120	76.6	86.6	80	1.61	1.31	1.50	1.47	0.00080158		1
P62244	RPS15A	7	122.2	115	119.5	77.8	85	80.5	1.57	1.35	1.48	1.47	0.00021951		1
P62249	RPS16	15	122.7	130.1	119.4	77.3	69.9	80.6	1.59	1.86	1.48	1.64	0.00042315		1
P08708	RPS17	11	119.3	110.2	114.2	80.7	89.8	85.8	1.48	1.23	1.33	1.35	0.0014414		1
P62269	RPS18	13	118.6	109.5	124.6	81.4	90.5	75.4	1.46	1.21	1.65	1.44	0.0048041		1
P39019	RPS19	15	122.3	116.6	116.4	77.7	83.4	83.6	1.57	1.40	1.39	1.45	0.00017535		1
P15880	RPS2	15	89.9	110.1	97.1	110.1	89.9	102.9	0.82	1.22	0.94	0.99	0.82844393		1
P60866	RPS20	5	124	114.7	124.5	76	85.3	75.5	1.63	1.34	1.65	1.54	0.00072879		1
P63220	RPS21	5	122.8	106.4	112	77.2	93.6	88	1.59	1.14	1.27	1.33	0.01566423		1
P62266	RPS23	7	123.8	114.6	122.4	76.2	85.4	77.6	1.62	1.34	1.58	1.51	0.00055888		1
P62847	RPS24	5	120.7	118.4	112.3	79.3	81.6	87.7	1.52	1.45	1.28	1.42	0.00064038		1
P62854	RPS26	5	124.4	110.5	125.6	75.6	89.5	74.4	1.65	1.23	1.69	1.52	0.00416594		1
Q71UM5	RPS27L	2	71.7	61.5	93.3	128.3	138.5	106.7	0.56	0.44	0.87	0.63	0.02090788		1
P62857	RPS28	5	121.2	112.2	111.3	78.8	87.8	88.7	1.54	1.28	1.25	1.36	0.00263021		1

P62273	RPS29	3	118.3	108	128.6	81.7	92	71.4	1.45	1.17	1.80	1.47	0.01213797	1
P23396	RPS3	24	102.7	114.4	100.1	97.3	85.6	99.9	1.06	1.34	1.00	1.13	0.13900679	1
P62861	FAU	3	124.7	99.5	135.4	75.3	100.5	64.6	1.66	0.99	2.10	1.58	0.05756792	1
P61247	RPS3A	30	87.6	108.9	84.7	112.4	91.1	115.3	0.78	1.20	0.73	0.90	0.30998431	1
P62701	RPS4X	15	97.3	116.1	100.1	102.7	83.9	99.9	0.95	1.38	1.00	1.11	0.33825905	1
P46782	RPS5	14	118.8	118	109.6	81.2	82	90.4	1.46	1.44	1.21	1.37	0.00174848	1
P62753	RPS6	19	76.2	116	89.2	123.8	84	110.8	0.62	1.38	0.81	0.93	0.49587188	1
P62241	RPS8	17	95.9	110.4	88.9	104.1	89.6	111.1	0.92	1.23	0.80	0.98	0.73885277	1
P08865	RPSA	16	115.9	118.7	124	84.1	81.3	76	1.38	1.46	1.63	1.49	0.00031233	1
Q9H0D6	XRN2	25	105.3	91.8	99.4	94.7	108.2	100.6	1.11	0.85	0.99	0.98	0.69454759	1
Q13131	PRKAA1	7	132.8	128.9	114.4	67.2	71.1	85.6	1.98	1.81	1.34	1.71	0.00304566	1
P54619	PRKAG1	11	128.6	93.1	121.3	71.4	106.9	78.7	1.80	0.87	1.54	1.40	0.13440094	1
P49914	MTHFS	6	71.2	115.4	47.4	128.8	84.6	152.6	0.55	1.36	0.31	0.74	0.19338371	1
O14841	OPLAH	13	100.3	41.8	25.8	99.7	158.2	174.2	1.01	0.26	0.15	0.47	0.05136288	1
P56378	MP68	2	94.9	108.3	147.7	105.1	91.7	52.3	0.90	1.18	2.82	1.64	0.20453403	1
P10809	HSPD1	58	98.8	93.5	100.5	101.2	106.5	99.5	0.98	0.88	1.01	0.95	0.18264829	1
P05388	RPLP0	16	100.5	126.9	102.2	99.5	73.1	97.8	1.01	1.74	1.04	1.26	0.17724864	1
P05387	RPLP2	8	118.3	111.7	122.4	81.7	88.3	77.6	1.45	1.27	1.58	1.43	0.00137196	1
P27635	RPL10	14	111.8	106.3	96.4	88.2	93.7	103.6	1.27	1.13	0.93	1.11	0.20385077	1
P62906	RPL10A	13	86.7	129.5	94.8	113.3	70.5	105.2	0.77	1.84	0.90	1.17	0.71297822	1
P62913	RPL11	5	130.4	119.3	127.4	69.6	80.7	72.6	1.87	1.48	1.75	1.70	0.00039323	1
P26373	RPL13	18	115	122.8	85.8	85	77.2	114.2	1.35	1.59	0.75	1.23	0.37910205	1
P50914	RPL14	7	114.3	96.5	88.8	85.7	103.5	111.2	1.33	0.93	0.80	1.02	0.98127418	1
P61313	RPL15	10	123.9	143.5	109.4	76.1	56.5	90.6	1.63	2.54	1.21	1.79	0.02150078	1
Q07020	RPL18	10	123.1	112.3	126	76.9	87.7	74	1.60	1.28	1.70	1.53	0.00225914	1
Q02543	RPL18A	10	126.1	108.1	127	73.9	91.9	73	1.71	1.18	1.74	1.54	0.00939958	1
P84098	RPL19	10	103.1	117.6	86.8	96.9	82.4	113.2	1.06	1.43	0.77	1.09	0.7113538	1
P46778	RPL21	10	129.4	121.4	129.9	70.6	78.6	70.1	1.83	1.54	1.85	1.74	0.00015914	1
Q9UNX3	RPL26L1	2	147.2	158.7	115.1	52.8	41.3	84.9	2.79	3.84	1.36	2.66	0.0119477	1
P46776	RPL27A	6	127.3	116.5	138.9	72.7	83.5	61.1	1.75	1.40	2.27	1.81	0.00381759	1
P47914	RPL29	3	130.2	140.5	127.4	69.8	59.5	72.6	1.87	2.36	1.75	1.99	0.00031442	1
P62888	RPL30	9	129.1	120.6	127.2	70.9	79.4	72.8	1.82	1.52	1.75	1.70	0.00014791	1
P62899	RPL31	7	130	129.3	117.6	70	70.7	82.4	1.86	1.83	1.43	1.70	0.0008389	1
P49207	RPL34	7	130.3	121.1	131.4	69.7	78.9	68.6	1.87	1.53	1.92	1.77	0.0002807	1
P42766	RPL35	5	130.3	116.3	132.3	69.7	83.7	67.7	1.87	1.39	1.95	1.74	0.00178817	1
Q9Y3U8	RPL36	5	129.2	116.9	126.1	70.8	83.1	73.9	1.82	1.41	1.71	1.65	0.00077051	1
Q969Q0	RPL36AL	2	122.2	121.5	115.7	77.8	78.5	84.3	1.57	1.55	1.37	1.50	0.00016957	1
P61513	RPL37A	6	127.5	122.6	115.5	72.5	77.4	84.5	1.76	1.58	1.37	1.57	0.00088946	1
P63173	RPL38	5	135.7	139.8	116.4	64.3	60.2	83.6	2.11	2.32	1.39	1.94	0.00387086	1
P36578	RPL4	24	178.1	127.6	156.3	21.9	72.4	43.7	8.13	1.76	3.58	4.49	0.00641742	1
P46777	RPL5	27	107.1	125.2	109.7	92.9	74.8	90.3	1.15	1.67	1.21	1.35	0.02480168	1

Q02878	RPL6	20	99.5	130	97.7	100.5	70	102.3	0.99	1.86	0.96	1.27	0.28827053	1
P18124	RPL7	20	94.6	128.6	96.5	105.4	71.4	103.5	0.90	1.80	0.93	1.21	0.447224	1
P62424	RPL7A	18	91.4	123.4	86.6	108.6	76.6	113.4	0.84	1.61	0.76	1.07	0.95717448	1
P62917	RPL8	16	92.6	119.6	84.2	107.4	80.4	115.8	0.86	1.49	0.73	1.03	0.88143595	1
P32969	RPL9	6	127.9	99.9	131.3	72.1	100.1	68.7	1.77	1.00	1.91	1.56	0.04879099	1
Q9Y221	NIP7	3	179.3	190.8	130.7	20.7	9.2	69.3	8.66	20.74	1.89	10.43	0.00679438	1
O95336	PGLS	13	124.4	111.9	119	75.6	88.1	81	1.65	1.27	1.47	1.46	0.00196988	1
Q9UBM7	DHCR7	5	98.3	77.3	92.3	101.7	122.7	107.7	0.97	0.63	0.86	0.82	0.07252171	1
Q7L2J0	MEPCE	7	143.4	124.7	143.7	56.6	75.3	56.3	2.53	1.66	2.55	2.25	0.00110575	1
Q8IZP0	ABI1	6	102.6	112.2	85.8	97.4	87.8	114.2	1.05	1.28	0.75	1.03	0.97251136	1
Q86V21	AACS	3	85	109.3	95.2	115	90.7	104.8	0.74	1.21	0.91	0.95	0.52103842	1
A1L0T0	ILVBL	5	128.2	133	147.4	71.8	67	52.6	1.79	1.99	2.80	2.19	0.00089069	1
P24752	ACAT1	19	133.4	110	113.7	66.6	90	86.3	2.00	1.22	1.32	1.51	0.02071867	1
Q13085	ACACA	40	96.8	92.5	99.3	103.2	107.5	100.7	0.94	0.86	0.99	0.93	0.05374576	1
Q9NR19	ACSS2	4	130.7	108.4	102.8	69.3	91.6	97.2	1.89	1.18	1.06	1.38	0.0813231	1
P39687	ANP32A	10	114.8	100.5	109.1	85.2	99.5	90.9	1.35	1.01	1.20	1.19	0.05046209	1
Q92688	ANP32B	7	117.3	95.1	118.2	82.7	104.9	81.8	1.42	0.91	1.44	1.26	0.12882562	1
Q9BTT0	ANP32E	8	95.1	91.2	116.7	104.9	108.8	83.3	0.91	0.84	1.40	1.05	0.86713135	1
P68032	ACTC1	4	97	102.4	102.6	103	97.6	97.4	0.94	1.05	1.05	1.01	0.63434101	1
P60709	ACTB	10	100.1	100.6	102.8	99.9	99.4	97.2	1.00	1.01	1.06	1.02	0.11752165	1
P61160	ACTR2	11	114.4	108	115.5	85.6	92	84.5	1.34	1.17	1.37	1.29	0.00157618	1
O15143	ARPC1B	16	106.5	82.6	105.9	93.5	117.4	94.1	1.14	0.70	1.13	0.99	0.77944146	1
O15144	ARPC2	23	103.8	103	105.1	96.2	97	94.9	1.08	1.06	1.11	1.08	0.00078609	1
O15145	ARPC3	7	118	95.5	110.5	82	104.5	89.5	1.44	0.91	1.23	1.20	0.16235099	1
P59998	ARPC4	6	113	131.7	104.1	87	68.3	95.9	1.30	1.93	1.09	1.44	0.04741723	1
O15511	ARPC5	2	88.1	82.5	96.9	111.9	117.5	103.1	0.79	0.70	0.94	0.81	0.02166397	1
O95433	AHSA1	13	28.5	125.5	132.7	171.5	74.5	67.3	0.17	1.68	1.97	1.27	0.86104832	1
Q12979	ABR	6	113.7	95.6	168.9	86.3	104.4	31.1	1.32	0.92	5.43	2.55	0.16979783	1
P13798	APEH	9	118.2	110	114.5	81.8	90	85.5	1.44	1.22	1.34	1.34	0.00105529	1
O00767	SCD	4	110.2	113	95.8	89.8	87	104.2	1.23	1.30	0.92	1.15	0.16806642	1
Q96CM8	ACSF2	6	107.3	95.8	111.7	92.7	104.2	88.3	1.16	0.92	1.27	1.11	0.2150259	1
P07108	DBI	6	112.3	104.1	129.4	87.7	95.9	70.6	1.28	1.09	1.83	1.40	0.04424987	1
Q9NPJ3	ACOT13	3	84.7	113.8	136	115.3	86.2	64	0.73	1.32	2.13	1.39	0.33505853	1
P07311	ACYP1	3	140.9	138.8	95.8	59.1	61.2	104.2	2.38	2.27	0.92	1.86	0.07261809	1
O75608	LYPLA1	5	128.9	108.1	142.2	71.1	91.9	57.8	1.81	1.18	2.46	1.82	0.01973135	1
Q6P587	FAHD1	6	88.5	76.9	100.4	111.5	123.1	99.6	0.79	0.62	1.01	0.81	0.07628237	1
P46108	CRK	11	93	69.6	93.3	107	130.4	106.7	0.87	0.53	0.87	0.76	0.05709429	1
Q9NVZ3	NECAP2	7	165.8	119.9	147.9	34.2	80.1	52.1	4.85	1.50	2.84	3.06	0.0092041	1
P07741	APRT	9	117.3	100.7	121	82.7	99.3	79	1.42	1.01	1.53	1.32	0.04216583	1
Q96HN2	AHCYL2	2	127.5	106.2	122	72.5	93.8	78	1.76	1.13	1.56	1.48	0.0146958	1
P23526	AHCY	22	144.5	112	122.9	55.5	88	77.1	2.60	1.27	1.59	1.82	0.01725712	1

P54819	AK2	15	124.7	99	126.1	75.3	101	73.9	1.66	0.98	1.71	1.45	0.05610243	1
P27144	AK4	9	101.6	123	146.2	98.4	77	53.8	1.03	1.60	2.72	1.78	0.06058284	1
Q01518	CAP1	31	115.9	143.6	143.7	84.1	56.4	56.3	1.38	2.55	2.55	2.16	0.00625739	1
P12235	SLC25A4	2	63.7	87.8	90.7	136.3	112.2	109.3	0.47	0.78	0.83	0.69	0.03340933	1
P05141	SLC25A5	8	88.3	101.5	88.8	111.7	98.5	111.2	0.79	1.03	0.80	0.87	0.07975307	1
P12236	SLC25A6	3	98.8	114.3	90.8	101.2	85.7	109.2	0.98	1.33	0.83	1.05	0.80301346	1
P84077	ARF1	8	99.7	91	90.4	100.3	109	109.6	0.99	0.83	0.82	0.88	0.04135242	1
P18085	ARF4	6	127.2	197	120.7	72.8	3	79.3	1.75	65.67	1.52	22.98	0.04896588	1
P84085	ARF5	5	108.4	90.7	24.8	91.6	109.3	175.2	1.18	0.83	0.14	0.72	0.23123858	1
P62330	ARF6	4	73.8	67.1	79.9	126.2	132.9	120.1	0.58	0.50	0.67	0.58	0.00054067	1
Q8N6H7	ARFGAP2	7	145.3	129.6	139.2	54.7	70.4	60.8	2.66	1.84	2.29	2.26	0.00029808	1
P40616	ARL1	2	128.6	84.6	119.9	71.4	115.4	80.1	1.80	0.73	1.50	1.34	0.31062843	1
P36404	ARL2	4	98.3	100.5	63.9	101.7	99.5	136.1	0.97	1.01	0.47	0.82	0.21202404	1
Q15041	ARL6IP1	4	114.9	116	122.8	85.1	84	77.2	1.35	1.38	1.59	1.44	0.00051136	1
Q9UKK9	NUDT5	12	124.1	98.7	120.8	75.9	101.3	79.2	1.64	0.97	1.53	1.38	0.06148278	1
Q9Y4W6	AFG3L2	7	157.7	112.6	149	42.3	87.4	51	3.73	1.29	2.92	2.65	0.01520722	1
O43488	AKR7A2	10	110.7	91.4	108.9	89.3	108.6	91.1	1.24	0.84	1.20	1.09	0.44696804	1
O00170	AIP	15	111.4	99.3	108	88.6	100.7	92	1.26	0.99	1.17	1.14	0.07069665	1
Q92667	AKAP1	5	91.6	71	94	108.4	129	106	0.85	0.55	0.89	0.76	0.0486753	1
Q12802	AKAP13	19	95.8	85	89.4	104.2	115	110.6	0.92	0.74	0.81	0.82	0.01098718	1
Q99996	AKAP9	5	144	160.7	175.8	56	39.3	24.2	2.57	4.09	7.26	4.64	0.00075466	1
P49588	AARS	56	104.1	98.4	106.3	95.9	101.6	93.7	1.09	0.97	1.13	1.06	0.15280744	1
P14550	AKR1A1	10	77.7	57.9	116.6	122.3	142.1	83.4	0.64	0.41	1.40	0.81	0.2613153	1
Q8IZ83	ALDH16A	6	57.8	56.5	83.8	142.2	143.5	116.2	0.41	0.39	0.72	0.51	0.00568227	1
Q04828	AKR1C1	2	20.4	51	21.6	179.6	149	178.4	0.11	0.34	0.12	0.19	0.00061929	1
P52895	AKR1C2	7	22.4	65.3	17.8	177.6	134.7	182.2	0.13	0.48	0.10	0.24	0.00373885	1
P42330	AKR1C3	19	26.6	47	24.6	173.4	153	175.4	0.15	0.31	0.14	0.20	0.00018515	1
Q96C23	GALM	11	39.1	61.3	51.5	160.9	138.7	148.5	0.24	0.44	0.35	0.34	0.00040668	1
P15121	AKR1B1	23	19.4	60.6	24.7	180.6	139.4	175.3	0.11	0.43	0.14	0.23	0.00206283	1
O00116	AGPS	21	67.7	61.9	75.2	132.3	138.1	124.8	0.51	0.45	0.60	0.52	0.00030958	1
Q6NUM9	RETSAT	3	88.1	60.8	84.7	111.9	139.2	115.3	0.79	0.44	0.73	0.65	0.02188537	1
Q9H6U8	ALG9	1	131.9	108.7	146.8	68.1	91.3	53.2	1.94	1.19	2.76	1.96	0.02053277	1
P35611	ADD1	14	112.6	102.6	114.1	87.4	97.4	85.9	1.29	1.05	1.33	1.22	0.01867053	1
P49419	ALDH7A1	21	124.2	89.4	94.3	75.8	110.6	105.7	1.64	0.81	0.89	1.11	0.74926891	1
O43768	ENSA	5	114.6	90.7	111.7	85.4	109.3	88.3	1.34	0.83	1.27	1.15	0.34719172	1
P54920	NAPA	20	68.4	72.7	56.7	131.6	127.3	143.3	0.52	0.57	0.40	0.50	0.00054542	1
Q12904	AIMP1	18	92	130.4	89.3	108	69.6	110.7	0.85	1.87	0.81	1.18	0.69907015	1
Q13155	AIMP2	10	105	136.8	105.9	95	63.2	94.1	1.11	2.16	1.13	1.47	0.09786019	1
Q06481	APLP2	6	164.9	133.6	156.5	35.1	66.4	43.5	4.70	2.01	3.60	3.44	0.00144899	1
O75179	ANKRD17	6	123.3	113.3	132.6	76.7	86.7	67.4	1.61	1.31	1.97	1.63	0.00424914	1
Q01484	ANK2	5	184.7	147.5	134.1	15.3	52.5	65.9	12.07	2.81	2.03	5.64	0.00660926	1

P04083	ANXA1	30	79.7	92.9	82.2	120.3	107.1	117.8	0.66	0.87	0.70	0.74	0.00623976	1
P50995	ANXA11	21	92.5	81.9	85	107.5	118.1	115	0.86	0.69	0.74	0.76	0.00369292	1
P27216	ANXA13	13	13.6	4	33.2	186.4	196	166.8	0.07	0.02	0.20	0.10	0.00016578	1
P07355	ANXA2	41	49.3	88.7	50.4	150.7	111.3	149.6	0.33	0.80	0.34	0.49	0.01533317	1
P12429	ANXA3	24	21	90.7	21.4	179	109.3	178.6	0.12	0.83	0.12	0.36	0.02737541	1
P20073	ANXA7	19	76.8	80.9	85.1	123.2	119.1	114.9	0.62	0.68	0.74	0.68	0.00035519	1
Q4KMQ2	ANO6	8	70.5	59.9	68.4	129.5	140.1	131.6	0.54	0.43	0.52	0.50	0.0001239	1
O95994	AGR2	10	8.4	4.4	73.9	191.6	195.6	126.1	0.04	0.02	0.59	0.22	0.01113417	1
Q03518	TAP1	3	131.9	89.8	138.9	68.1	110.2	61.1	1.94	0.81	2.27	1.68	0.13592378	1
O43747	AP1G1	8	96.5	126.8	99.8	103.5	73.2	100.2	0.93	1.73	1.00	1.22	0.31991475	1
P56377	AP1S2	1	162.9	171	133.7	37.1	29	66.3	4.39	5.90	2.02	4.10	0.00222177	1
O95782	AP2A1	6	115.5	109.5	106.8	84.5	90.5	93.2	1.37	1.21	1.15	1.24	0.00431091	1
O94973	AP2A2	8	95.9	97.8	101.3	104.1	102.2	98.7	0.92	0.96	1.03	0.97	0.2103777	1
P63010	AP2B1	18	114.8	112.2	119.4	85.2	87.8	80.6	1.35	1.28	1.48	1.37	0.00048437	1
Q2M2I8	AAK1	7	160.5	190.5	179.2	39.5	9.5	20.8	4.06	20.05	8.62	10.91	0.00024274	1
Q9BQE5	APOL2	6	102.6	82.5	111.9	97.4	117.5	88.1	1.05	0.70	1.27	1.01	0.87841744	1
O95831	AIFM1	20	99.9	91.7	100.3	100.1	108.3	99.7	1.00	0.85	1.01	0.95	0.24469633	1
Q13625	TP53BP2	7	172.4	126	100.3	27.6	74	99.7	6.25	1.70	1.01	2.99	0.09210034	1
P53367	ARFIP1	8	149.1	97.6	87.1	50.9	102.4	112.9	2.93	0.95	0.77	1.55	0.45235189	1
P52594	AGFG1	6	88.6	77.9	82.3	111.4	122.1	117.7	0.80	0.64	0.70	0.71	0.00147669	1
Q9NVT9	ARMC1	3	119.8	75.9	164	80.2	124.1	36	1.49	0.61	4.56	2.22	0.3305445	1
Q8N2F6	ARMC10	8	83.8	73.7	65.6	116.2	126.3	134.4	0.72	0.58	0.49	0.60	0.00233111	1
P18440	NAT1	4	28.1	82.9	97.7	171.9	117.1	102.3	0.16	0.71	0.96	0.61	0.11181374	1
P08243	ASNS	17	163.3	139.7	156.6	36.7	60.3	43.4	4.45	2.32	3.61	3.46	0.00042992	1
O43776	NARS	26	122.6	115	124.3	77.4	85	75.7	1.58	1.35	1.64	1.53	0.00051932	1
P17174	GOT1	16	188	134.9	122.2	12	65.1	77.8	15.67	2.07	1.57	6.44	0.02742646	1
P00505	GOT2	21	84.7	84.1	89.2	115.3	115.9	110.8	0.73	0.73	0.81	0.76	0.00025078	1
P14868	DARS	30	182.9	149.9	157	17.1	50.1	43	10.70	2.99	3.65	5.78	0.00087254	1
Q6PI48	DARS2	16	116.9	107.9	121.4	83.1	92.1	78.6	1.41	1.17	1.54	1.37	0.00537112	1
Q99700	ATXN2	9	147.6	118.8	155.8	52.4	81.2	44.2	2.82	1.46	3.52	2.60	0.006817	1
Q8WWM7	ATXN2L	20	149.6	134.4	143.3	50.4	65.6	56.7	2.97	2.05	2.53	2.51	0.00016875	1
P24539	ATP5F1	15	112.5	116.8	105	87.5	83.2	95	1.29	1.40	1.11	1.26	0.0093804	1
P00846	MT-ATP6	1	113.4	104.2	113.8	86.6	95.8	86.2	1.31	1.09	1.32	1.24	0.00916568	1
P25705	ATP5A1	35	116.6	109.9	114.7	83.4	90.1	85.3	1.40	1.22	1.34	1.32	0.00062178	1
P06576	ATP5B	28	125	112.4	119.8	75	87.6	80.2	1.67	1.28	1.49	1.48	0.0018008	1
P56385	ATP5I	4	97.4	80	89.3	102.6	120	110.7	0.95	0.67	0.81	0.81	0.03543282	1
P56134	ATP5J2	2	114.3	103.8	113.5	85.7	96.2	86.5	1.33	1.08	1.31	1.24	0.01156198	1
O75964	ATP5L	5	109.3	102.4	101.4	90.7	97.6	98.6	1.21	1.05	1.03	1.09	0.06773269	1
P36542	ATP5C1	9	93.1	139.2	92.9	106.9	60.8	107.1	0.87	2.29	0.87	1.34	0.48351878	1
Q8NBU5	ATAD1	5	114.4	111.1	117.8	85.6	88.9	82.2	1.34	1.25	1.43	1.34	0.00045611	1
Q9NVI7	ATAD3A	8	158.8	115.6	172.4	41.2	84.4	27.6	3.85	1.37	6.25	3.82	0.01558446	1

Q96S55	WRNIP1	5	140.2	143.5	117.6	59.8	56.5	82.4	2.34	2.54	1.43	2.10	0.00421429	1
P61221	ABCE1	14	126.9	116.4	129.6	73.1	83.6	70.4	1.74	1.39	1.84	1.66	0.00103395	1
Q9UG63	ABCF2	3	112.5	127.4	127.6	87.5	72.6	72.4	1.29	1.75	1.76	1.60	0.00312636	1
P17858	PFKL	3	110.1	105.4	98.4	89.9	94.6	101.6	1.22	1.11	0.97	1.10	0.1261522	1
P08237	PFKM	9	119.7	106.2	112.9	80.3	93.8	87.1	1.49	1.13	1.30	1.31	0.00935479	1
Q01813	PFKP	34	92.3	85.5	91.4	107.7	114.5	108.6	0.86	0.75	0.84	0.82	0.00243197	1
Q16740	CLPP	8	137.3	80.2	97.5	62.7	119.8	102.5	2.19	0.67	0.95	1.27	0.69721346	1
Q92499	DDX1	31	98.4	98.7	105.7	101.6	101.3	94.3	0.97	0.97	1.12	1.02	0.60943092	1
Q9GZR7	DDX24	5	147.4	109	101	52.6	91	99	2.80	1.20	1.02	1.67	0.13183167	1
Q86XP3	DDX42	15	109.2	78.8	98.7	90.8	121.2	101.3	1.20	0.65	0.97	0.94	0.52064018	1
Q7Z478	DHX29	9	116	96.4	103.1	84	103.6	96.9	1.38	0.93	1.06	1.13	0.27281178	1
Q14562	DHX8	2	94.2	153.3	133.5	105.8	46.7	66.5	0.89	3.28	2.01	2.06	0.09279989	1
Q8IYB8	SUPV3L1	6	118.3	122.3	151.1	81.7	77.7	48.9	1.45	1.57	3.09	2.04	0.01387213	1
Q96BJ3	AIDA	7	27.3	78.8	87.7	172.7	121.2	112.3	0.16	0.65	0.78	0.53	0.05643521	1
Q9NR09	BIRC6	8	116.5	104.5	121.6	83.5	95.5	78.4	1.40	1.09	1.55	1.35	0.01664888	1
O95817	BAG3	10	136.5	127.5	125.4	63.5	72.5	74.6	2.15	1.76	1.68	1.86	0.00024476	1
P50895	BCAM	5	101.8	39.4	107.5	98.2	160.6	92.5	1.04	0.25	1.16	0.81	0.32973049	1
P35613	BSG	7	99.1	101.1	111.2	100.9	98.9	88.8	0.98	1.02	1.25	1.09	0.22459265	1
P51572	BCAP31	23	107.1	89.8	106.3	92.9	110.2	93.7	1.15	0.81	1.13	1.03	0.802271	1
Q07817	BCL2L1	2	139.3	85.6	122.4	60.7	114.4	77.6	2.29	0.75	1.58	1.54	0.23229774	1
O15155	BET1	2	79.8	102	109.3	120.2	98	90.7	0.66	1.04	1.21	0.97	0.66089092	1
Q8WYA6	CTNNBL1	6	146.2	127.6	142.4	53.8	72.4	57.6	2.72	1.76	2.47	2.32	0.00064385	1
P16278	GLB1	6	62.6	62.1	87.8	137.4	137.9	112.2	0.46	0.45	0.78	0.56	0.00827018	1
P06865	HEXA	7	44.1	79.7	80.3	155.9	120.3	119.7	0.28	0.66	0.67	0.54	0.01947939	1
P07686	HEXB	14	131.4	121	114.4	68.6	79	85.6	1.92	1.53	1.34	1.59	0.00312565	1
P55957	BID	4	72.4	62.6	76.1	127.6	137.4	123.9	0.57	0.46	0.61	0.55	0.00048178	1
Q13057	COASY	9	83.3	81.9	89.6	116.7	118.1	110.4	0.71	0.69	0.81	0.74	0.00084462	1
P07814	EPRS	64	116.4	111.3	121.5	83.6	88.7	78.5	1.39	1.25	1.55	1.40	0.00140434	1
P13995	MTHFD2	10	129.9	118.9	129	70.1	81.1	71	1.85	1.47	1.82	1.71	0.00048262	1
P31939	ATIC	38	91.8	89.5	98.5	108.2	110.5	101.5	0.85	0.81	0.97	0.88	0.02428868	1
Q9Y223	GNE	8	120.5	93	134.4	79.5	107	65.6	1.52	0.87	2.05	1.48	0.13698777	1
P53004	BLVRA	10	81.4	78.3	91.9	118.6	121.7	108.1	0.69	0.64	0.85	0.73	0.00517568	1
P78537	BLOC1S1	2	113.7	97.5	72.4	86.3	102.5	127.6	1.32	0.95	0.57	0.95	0.55494204	1
Q8NFC6	BOD1L1	31	121	112.1	122.9	79	87.9	77.1	1.53	1.28	1.59	1.47	0.00136868	1
P07738	BPGM	5	159.5	74.6	79.8	40.5	125.4	120.2	3.94	0.59	0.66	1.73	0.8232166	1
Q9H3K6	BOLA2	5	134.1	100.8	142.3	65.9	99.2	57.7	2.03	1.02	2.47	1.84	0.04556324	1
Q9UHR4	BAIAP2L1	9	106.3	143.6	131.7	93.7	56.4	68.3	1.13	2.55	1.93	1.87	0.02496581	1
Q6PJG6	BRAT1	9	135.6	124.6	146.3	64.4	75.4	53.7	2.11	1.65	2.72	2.16	0.00131499	1
Q9Y6D6	ARFGEF1	22	143.7	134.5	156.4	56.3	65.5	43.6	2.55	2.05	3.59	2.73	0.00056329	1
Q9Y6D5	ARFGEF2	5	115.5	129.1	119.6	84.5	70.9	80.4	1.37	1.82	1.49	1.56	0.00167918	1
Q5TH69	ARFGEF3	5	117.3	118	155.1	82.7	82	44.9	1.42	1.44	3.45	2.10	0.02694547	1

P25440	BRD2	7	152	138	154	48	62	46	3.17	2.23	3.35	2.91	0.00017489	1
O60885	BRD4	13	140.5	144.2	102.3	59.5	55.8	97.7	2.36	2.58	1.05	2.00	0.03757446	1
P11586	MTHFD1	45	118.8	111.3	117.9	81.2	88.7	82.1	1.46	1.25	1.44	1.38	0.00066599	1
O75844	ZMPSTE24	5	125.9	101.9	108.9	74.1	98.1	91.1	1.70	1.04	1.20	1.31	0.07214782	1
P27708	CAD	62	102.3	104.2	112.6	97.7	95.8	87.4	1.05	1.09	1.29	1.14	0.04662132	1
Q8IWX8	CHERP	11	165.1	144.5	193.5	34.9	55.5	6.5	4.73	2.60	29.77	12.37	0.00252509	1
Q9UM00	TMCO1	5	96.4	69.7	96.8	103.6	130.3	103.2	0.93	0.53	0.94	0.80	0.12291536	1
P49069	CAMLG	4	72	176.2	149.4	128	23.8	50.6	0.56	7.40	2.95	3.64	0.21480193	1
Q8NE86	MCU	9	44.7	93.8	56.1	155.3	106.2	143.9	0.29	0.88	0.39	0.52	0.02859645	1
Q9Y376	CAB39	13	110.5	115.7	113	89.5	84.3	87	1.23	1.37	1.30	1.30	0.0002504	1
P98194	ATP2C1	3	67.2	92.1	66	132.8	107.9	134	0.51	0.85	0.49	0.62	0.01438379	1
Q9HB71	CACYBP	20	119.4	102.6	116.6	80.6	97.4	83.4	1.48	1.05	1.40	1.31	0.02486068	1
Q96JQ2	CLMN	3	87.4	97.1	63.3	112.6	102.9	136.7	0.78	0.94	0.46	0.73	0.07052391	1
P62158	CALM1	5	110.4	88.1	76.6	89.6	111.9	123.4	1.23	0.79	0.62	0.88	0.3023034	1
Q5T5Y3	CAMSAP1	5	159.9	149.3	193.1	40.1	50.7	6.9	3.99	2.94	27.99	11.64	0.00194314	1
P27824	CANX	33	106.2	103.2	106.1	93.8	96.8	93.9	1.13	1.07	1.13	1.11	0.00175412	1
P07384	CAPN1	35	78.7	72.5	82.5	121.3	127.5	117.5	0.65	0.57	0.70	0.64	0.00042846	1
P17655	CAPN2	29	105.2	101	105.5	94.8	99	94.5	1.11	1.02	1.12	1.08	0.01915337	1
Q99439	CNN2	13	124.2	114.4	124.8	75.8	85.6	75.2	1.64	1.34	1.66	1.54	0.00089402	1
Q15417	CNN3	7	144.3	132.9	127.5	55.7	67.1	72.5	2.59	1.98	1.76	2.11	0.00056916	1
O43852	CALU	7	179.2	108.2	108.4	20.8	91.8	91.6	8.62	1.18	1.18	3.66	0.12861571	1
P56211	ARPP19	2	92.3	84.8	107.2	107.7	115.2	92.8	0.86	0.74	1.16	0.92	0.32379623	1
P30622	CLIP1	39	115.8	114.5	122.3	84.2	85.5	77.7	1.38	1.34	1.57	1.43	0.0005055	1
Q9UDT6	CLIP2	5	116.1	56.3	71	83.9	143.7	129	1.38	0.39	0.55	0.78	0.21220809	1
Q14444	CAPRIN1	12	141.7	130.8	151.8	58.3	69.2	48.2	2.43	1.89	3.15	2.49	0.00064157	1
Q8N1G2	CMTR1	5	175.6	76.3	92.2	24.4	123.7	107.8	7.20	0.62	0.86	2.89	0.53662637	1
O43570	CA12	9	66.1	4	3.1	133.9	196	196.9	0.49	0.02	0.02	0.18	0.00685086	1
P00918	CA2	9	141.7	113.3	183.1	58.3	86.7	16.9	2.43	1.31	10.83	4.86	0.03251536	1
P16152	CBR1	17	71	95.3	65.2	129	104.7	134.8	0.55	0.91	0.48	0.65	0.02484407	1
O75828	CBR3	10	96.4	100.6	106.5	103.6	99.4	93.5	0.93	1.01	1.14	1.03	0.60334897	1
Q96DG6	CMBL	3	76.4	98.9	94.3	123.6	101.1	105.7	0.62	0.98	0.89	0.83	0.10505584	1
P43155	CRAT	7	108.1	95.9	99	91.9	104.1	101	1.18	0.92	0.98	1.03	0.71895283	1
P50416	CPT1A	11	92	78.3	88.2	108	121.7	111.8	0.85	0.64	0.79	0.76	0.0087059	1
P48729	CSNK1A1	13	109	128.2	125.1	91	71.8	74.9	1.20	1.79	1.67	1.55	0.00784447	1
P67870	CSNK2B	7	110.9	128.5	110.7	89.1	71.5	89.3	1.24	1.80	1.24	1.43	0.01609322	1
P42574	CASP3	8	133.6	103	114.5	66.4	97	85.5	2.01	1.06	1.34	1.47	0.05412903	1
P55212	CASP6	4	126.2	99.2	107.2	73.8	100.8	92.8	1.71	0.98	1.16	1.28	0.12737293	1
P55210	CASP7	4	63.7	90.8	133.9	136.3	109.2	66.1	0.47	0.83	2.03	1.11	0.80228704	1
P21964	COMT	15	110.6	87.3	115.8	89.4	112.7	84.2	1.24	0.77	1.38	1.13	0.50201311	1
P35221	CTNNA1	29	78	69.8	76.1	122	130.2	123.9	0.64	0.54	0.61	0.60	0.00013239	1
P35222	CTNNB1	13	94.7	112.8	93.9	105.3	87.2	106.1	0.90	1.29	0.89	1.03	0.91998061	1

O60716	CTNND1	21	75.3	74.6	83.7	124.7	125.4	116.3	0.60	0.59	0.72	0.64	0.00043129		1
P07858	CTSB	11	60.8	66.6	77.4	139.2	133.4	122.6	0.44	0.50	0.63	0.52	0.00076685		1
P07339	CTSD	17	88.1	65.2	91.1	111.9	134.8	108.9	0.79	0.48	0.84	0.70	0.03276196		1
P20645	M6PR	5	125.8	91.2	88.6	74.2	108.8	111.4	1.70	0.84	0.80	1.11	0.83651994		1
P11717	IGF2R	48	112.1	103.3	112.1	87.9	96.7	87.9	1.28	1.07	1.28	1.21	0.0115161		1
Q03135	CAV1	8	137.3	165.2	138.8	62.7	34.8	61.2	2.19	4.75	2.27	3.07	0.00182319		1
A5YKK6	CNOT1	21	106.3	100.2	109.3	93.7	99.8	90.7	1.13	1.00	1.21	1.11	0.04971627		1
Q9UIV1	CNOT7	3	105.8	134.8	58.2	94.2	65.2	141.8	1.12	2.07	0.41	1.20	0.98100175		1
P48509	CD151	6	113.8	93.5	101.7	86.2	106.5	98.3	1.32	0.88	1.03	1.08	0.51158714		1
P60033	CD81	3	83.6	87.7	78.9	116.4	112.3	121.1	0.72	0.78	0.65	0.72	0.00076447		1
Q15642	TRIP10	11	139.7	125.1	145.1	60.3	74.9	54.9	2.32	1.67	2.64	2.21	0.00097274		1
Q9NZ45	CISD1	3	139.9	84.5	102.3	60.1	115.5	97.7	2.33	0.73	1.05	1.37	0.48383803		1
Q8N5K1	CISD2	6	111.1	99.8	107	88.9	100.2	93	1.25	1.00	1.15	1.13	0.06297555		1
Q96JB5	CDK5RAP	5	91.8	140.9	131	108.2	59.1	69	0.85	2.38	1.90	1.71	0.11571677		1
Q9NXV6	CDKN2AI	3	122.9	130.3	121.2	77.1	69.7	78.8	1.59	1.87	1.54	1.67	0.00023158		1
Q8N163	CCAR2	32	117.9	113.8	123	82.1	86.2	77	1.44	1.32	1.60	1.45	0.00063476		1
Q92600	RQCD1	5	102.3	68.5	100.9	97.7	131.5	99.1	1.05	0.52	1.02	0.86	0.29347141		1
P60953	CDC42	8	112.8	105.3	108.9	87.2	94.7	91.1	1.29	1.11	1.20	1.20	0.00418767		1
P62633	CNBP	7	143	114.9	120.7	57	85.1	79.3	2.51	1.35	1.52	1.79	0.01238963		1
O43264	ZW10	5	145.4	155.1	164.5	54.6	44.9	35.5	2.66	3.45	4.63	3.58	0.00014658		1
Q96G23	CERS2	4	121.1	105.8	120.5	78.9	94.2	79.5	1.53	1.12	1.52	1.39	0.01110681		1
Q9NWW5	CLN6	2	89.2	103.7	105.3	110.8	96.3	94.7	0.81	1.08	1.11	1.00	0.87643084		1
Q9HD42	CHMP1A	3	116.2	108.3	132.3	83.8	91.7	67.7	1.39	1.18	1.95	1.51	0.01924095		1
Q9UQN3	CHMP2B	8	88.3	31.9	61.3	111.7	168.1	138.7	0.79	0.19	0.44	0.47	0.02653593		1
Q9Y3E7	CHMP3	5	68.5	76.6	50.1	131.5	123.4	149.9	0.52	0.62	0.33	0.49	0.003242		1
Q9H444	CHMP4B	5	94.6	76	112.1	105.4	124	87.9	0.90	0.61	1.28	0.93	0.47767856		1
Q9NZZ3	CHMP5	5	122.9	94.4	120.6	77.1	105.6	79.4	1.59	0.89	1.52	1.34	0.12231597		1
Q96FZ7	CHMP6	7	93.1	66.5	54.9	106.9	133.5	145.1	0.87	0.50	0.38	0.58	0.02349023		1
O00299	CLIC1	16	131.1	112.2	130.3	68.9	87.8	69.7	1.90	1.28	1.87	1.68	0.00492068		1
Q9Y696	CLIC4	16	139.2	100.9	142.8	60.8	99.1	57.2	2.29	1.02	2.50	1.93	0.04346661		1
P49585	PCYT1A	5	142.2	124.3	131.8	57.8	75.7	68.2	2.46	1.64	1.93	2.01	0.00086998		1
Q9Y3Y2	CHTOP	3	119.7	92.8	106.6	80.3	107.2	93.4	1.49	0.87	1.14	1.17	0.31080375		1
Q13185	CBX3	8	105.3	92.2	112.7	94.7	107.8	87.3	1.11	0.86	1.29	1.09	0.46736781		1
Q96JM3	CHAMP1	2	135.8	151.2	162.6	64.2	48.8	37.4	2.12	3.10	4.35	3.19	0.00081497		1
O75390	CS	15	109.3	115.6	110.5	90.7	84.4	89.5	1.21	1.37	1.23	1.27	0.00098665		1
Q00610	CLTC	53	135.1	124.5	130.5	64.9	75.5	69.5	2.08	1.65	1.88	1.87	0.00015798		1
Q14677	CLINT1	6	69	134.8	82	131	65.2	118	0.53	2.07	0.69	1.10	0.75606317		1
P09496	CLTA	10	95.1	75.2	89	104.9	124.8	111	0.91	0.60	0.80	0.77	0.0310956		1
O43809	NUDT21	13	98.1	99.6	84.6	101.9	100.4	115.4	0.96	0.99	0.73	0.90	0.15513424		1
Q12996	CSTF3	8	150.8	26.4	128.8	49.2	173.6	71.2	3.07	0.15	1.81	1.68	0.9447188		1
Q96KA5	CLPTM1L	2	94.6	71.3	92.5	105.4	128.7	107.5	0.90	0.55	0.86	0.77	0.05786335		1

Q7Z460	CLASP1	11	139	130.1	160.1	61	69.9	39.9	2.28	1.86	4.01	2.72	0.00238169	1
O75122	CLASP2	6	147.3	124.6	136.7	52.7	75.4	63.3	2.80	1.65	2.16	2.20	0.00145274	1
O75153	CLUH	31	109.3	92.7	106.8	90.7	107.3	93.2	1.21	0.86	1.15	1.07	0.46707568	1
Q14019	COTL1	14	130.9	125.4	137.3	69.1	74.6	62.7	1.89	1.68	2.19	1.92	0.0002126	1
P35606	COPB2	34	117.1	109.9	119.3	82.9	90.1	80.7	1.41	1.22	1.48	1.37	0.00153897	1
P48444	ARCN1	21	119.8	128	124.6	80.2	72	75.4	1.49	1.78	1.65	1.64	0.00013708	1
O14579	COPE	13	102.8	115	105.9	97.2	85	94.1	1.06	1.35	1.13	1.18	0.03796668	1
Q9Y678	COPG1	23	130.3	120	123.1	69.7	80	76.9	1.87	1.50	1.60	1.66	0.00034459	1
Q9UBF2	COPG2	5	143.1	113.5	133.2	56.9	86.5	66.8	2.51	1.31	1.99	1.94	0.00824319	1
P61923	COPZ1	6	124.1	108.3	121.4	75.9	91.7	78.6	1.64	1.18	1.54	1.45	0.00652615	1
P23528	CFL1	14	109	106.6	113.4	91	93.4	86.6	1.20	1.14	1.31	1.22	0.00235667	1
Q9Y281	CFL2	5	94.1	90.6	100.9	105.9	109.4	99.1	0.89	0.83	1.02	0.91	0.0881383	1
Q8N3U4	STAG2	6	142.4	141.5	158.7	57.6	58.5	41.3	2.47	2.42	3.84	2.91	0.00027403	1
Q96CT7	CCDC124	4	111.8	105.1	166.9	88.2	94.9	33.1	1.27	1.11	5.04	2.47	0.11380253	1
Q4VC31	CCDC58	4	129	129.6	110.7	71	70.4	89.3	1.82	1.84	1.24	1.63	0.00622468	1
Q16204	CCDC6	8	120.4	63.5	115.6	79.6	136.5	84.4	1.51	0.47	1.37	1.12	0.99029769	1
O75534	CSDE1	23	133.9	125.6	139.9	66.1	74.4	60.1	2.03	1.69	2.33	2.01	0.00034925	1
Q02388	COL7A1	8	5.6	4.6	50.4	194.4	195.4	149.6	0.03	0.02	0.34	0.13	0.00171456	1
Q9Y5P4	COL4A3B	6	123.9	121.9	135.5	76.1	78.1	64.5	1.63	1.56	2.10	1.76	0.00082956	1
Q07021	C1QBP	6	73.9	85.6	78.1	126.1	114.4	121.9	0.59	0.75	0.64	0.66	0.00100635	1
Q9NPL8	TIMMDC1	5	65.2	76.6	65.3	134.8	123.4	134.7	0.48	0.62	0.48	0.53	0.00031824	1
Q9BPX3	NCAPG	5	156.7	111.8	174.9	43.3	88.2	25.1	3.62	1.27	6.97	3.95	0.02265524	1
Q9NZB2	FAM120A	24	107.5	105	105.4	92.5	95	94.6	1.16	1.11	1.11	1.13	0.00040459	1
Q13098	GPS1	7	145.1	118.6	122.6	54.9	81.4	77.4	2.64	1.46	1.58	1.89	0.00785915	1
Q92905	COPS5	18	110.5	105.9	123.5	89.5	94.1	76.5	1.23	1.13	1.61	1.32	0.02339563	1
Q7L5N1	COPS6	14	119.5	105.5	118.5	80.5	94.5	81.5	1.48	1.12	1.45	1.35	0.01043692	1
Q99627	COPS8	3	127.4	110.8	145.7	72.6	89.2	54.3	1.75	1.24	2.68	1.89	0.01718699	1
Q99829	CPNE1	11	130.2	121.9	134.3	69.8	78.1	65.7	1.87	1.56	2.04	1.82	0.00036608	1
O75131	CPNE3	24	181.1	150.2	164.1	18.9	49.8	35.9	9.58	3.02	4.57	5.72	0.00049945	1
Q9NTM9	CUTC	6	116.3	97.9	77.7	83.7	102.1	122.3	1.39	0.96	0.64	0.99	0.74917999	1
Q9BR76	CORO1B	13	124.6	113.7	130.2	75.4	86.3	69.8	1.65	1.32	1.87	1.61	0.00263179	1
Q9ULV4	CORO1C	14	125.6	118	120.7	74.4	82	79.3	1.69	1.44	1.52	1.55	0.00016793	1
P57737	CORO7	6	129	117	130	71	83	70	1.82	1.41	1.86	1.69	0.00101456	1
Q9P1F3	ABRACL	1	133.3	123	159.8	66.7	77	40.2	2.00	1.60	3.98	2.52	0.00752675	1
Q92793	CREBBP	4	107	112.7	96.1	93	87.3	103.9	1.15	1.29	0.92	1.12	0.20088141	1
P46109	CRKL	9	148.6	89.7	138.5	51.4	110.3	61.5	2.89	0.81	2.25	1.99	0.11733838	1
P17812	CTPS1	14	121.2	114.3	122.6	78.8	85.7	77.4	1.54	1.33	1.58	1.49	0.000436	1
Q13616	CUL1	6	141.9	117.5	114.1	58.1	82.5	85.9	2.44	1.42	1.33	1.73	0.01671329	1
Q13617	CUL2	7	136.5	139.2	128.1	63.5	60.8	71.9	2.15	2.29	1.78	2.07	0.00012662	1
Q13618	CUL3	19	117.6	129.1	121.5	82.4	70.9	78.5	1.43	1.82	1.55	1.60	0.00067929	1
Q13620	CUL4B	11	122	125.9	131.9	78	74.1	68.1	1.56	1.70	1.94	1.73	0.00019811	1

P06493	CDK1	19	130.2	151.9	134.3	69.8	48.1	65.7	1.87	3.16	2.04	2.36	0.00118084		1
P21127	CDK11B	4	140.9	159.8	149.3	59.1	40.2	50.7	2.38	3.98	2.94	3.10	0.00020613		1
P11802	CDK4	5	147.3	128.6	108.7	52.7	71.4	91.3	2.80	1.80	1.19	1.93	0.02319936		1
Q00535	CDK5	10	93.6	133.2	81.9	106.4	66.8	118.1	0.88	1.99	0.69	1.19	0.80466449		1
P51946	CCNH	5	126.9	122.8	150.1	73.1	77.2	49.9	1.74	1.59	3.01	2.11	0.00520685		1
O75909	CCNK	4	140.5	128.4	132.7	59.5	71.6	67.3	2.36	1.79	1.97	2.04	0.00017299		1
P04080	CSTB	6	106.1	101.8	96	93.9	98.2	104	1.13	1.04	0.92	1.03	0.56392342		1
P21291	CSRP1	11	67.6	33.4	73.3	132.4	166.6	126.7	0.51	0.20	0.58	0.43	0.00893146		1
Q9UHD1	CHORDC1	16	128.5	116.7	125.3	71.5	83.3	74.7	1.80	1.40	1.68	1.63	0.00070427		1
P49589	CARS	18	103	97.8	104.4	97	102.2	95.6	1.06	0.96	1.09	1.04	0.28916325		1
P00156	MT-CYB	2	128.7	116.3	125.9	71.3	83.7	74.1	1.81	1.39	1.70	1.63	0.00088026		1
P22695	UQCRC2	13	143.7	96.2	100	56.3	103.8	100	2.55	0.93	1.00	1.49	0.28467324		1
O14949	UQCRQ	4	92.6	35	104	107.4	165	96	0.86	0.21	1.08	0.72	0.20558122		1
P47985	UQCRFS1	4	115.5	107.3	103.8	84.5	92.7	96.2	1.37	1.16	1.08	1.20	0.02242433		1
P99999	CYCS	10	154.9	135.2	150.7	45.1	64.8	49.3	3.43	2.09	3.06	2.86	0.00037743		1
Q9Y2R0	COA3	3	78.4	75.8	119.5	121.6	124.2	80.5	0.64	0.61	1.48	0.91	0.43049531		1
Q5RI15	COX20	1	117.4	114.6	149.2	82.6	85.4	50.8	1.42	1.34	2.94	1.90	0.02606231		1
P00403	MT-CO2	5	119.6	113.3	118.2	80.4	86.7	81.8	1.49	1.31	1.44	1.41	0.00022749		1
P14406	COX7A2	1	108	58.2	106.3	92	141.8	93.7	1.17	0.41	1.13	0.91	0.47156588		1
P15954	COX7C	1	115.6	104.5	109.2	84.4	95.5	90.8	1.37	1.09	1.20	1.22	0.01270824		1
O00483	NDUFA4	3	90.9	86.2	63	109.1	113.8	137	0.83	0.76	0.46	0.68	0.03066688		1
P53701	HCCS	11	137.7	113.6	141.8	62.3	86.4	58.2	2.21	1.31	2.44	1.99	0.00754824		1
P21399	ACO1	22	87.7	80.2	91.7	112.3	119.8	108.3	0.78	0.67	0.85	0.77	0.00483323		1
Q7L576	CYFIP1	10	134.4	128.3	141.8	65.6	71.7	58.2	2.05	1.79	2.44	2.09	0.00022688		1
Q07065	CKAP4	19	79.2	69.9	80.1	120.8	130.1	119.9	0.66	0.54	0.67	0.62	0.0005133		1
Q9H0P0	NT5C3A	7	83.4	91	79	116.6	109	121	0.72	0.83	0.65	0.73	0.00330684		1
O00154	ACOT7	13	164.4	140.8	163.1	35.6	59.2	36.9	4.62	2.38	4.42	3.81	0.0004903		1
P53384	NUBP1	4	87.6	91.9	101.7	112.4	108.1	98.3	0.78	0.85	1.03	0.89	0.10087259		1
Q9Y5Y2	NUBP2	5	69.8	88.1	85.4	130.2	111.9	114.6	0.54	0.79	0.75	0.69	0.00940321		1
P47712	PLA2G4A	17	5.2	5.6	117.8	194.8	194.4	82.2	0.03	0.03	1.43	0.50	0.09726561		1
Q96GG9	DCUN1D1	9	99.8	89.9	90.7	100.2	110.1	109.3	1.00	0.82	0.83	0.88	0.04367739		1
Q9BTE7	DCUN1D5	4	150.5	53.9	143.3	49.5	146.1	56.7	3.04	0.37	2.53	1.98	0.50929195		1
Q9H773	DCTPP1	4	79.8	93.1	102.2	120.2	106.9	97.8	0.66	0.87	1.04	0.86	0.1454404		1
P30046	DDT	5	132	130.2	145.9	68	69.8	54.1	1.94	1.87	2.70	2.17	0.00050637		1
Q96HY6	DDRGK1	6	138.1	127	129.8	61.9	73	70.2	2.23	1.74	1.85	1.94	0.00017818		1
Q9BTC0	DIDO1	9	119.1	100.2	101.5	80.9	99.8	98.5	1.47	1.00	1.03	1.17	0.18295232		1
Q96BY6	DOCK10	2	74.6	115.3	82.1	125.4	84.7	117.9	0.59	1.36	0.70	0.88	0.35073723		1
Q9BTZ2	DHRS4	4	20.7	63	21.2	179.3	137	178.8	0.12	0.46	0.12	0.23	0.00279118		1
Q6IAN0	DHRS7B	5	96.9	123.4	117.7	103.1	76.6	82.3	0.94	1.61	1.43	1.33	0.09017291		1
P30038	ALDH4A1	11	112.7	109.4	83.5	87.3	90.6	116.5	1.29	1.21	0.72	1.07	0.78912954		1
P54886	ALDH18A	20	112.5	104.4	107.4	87.5	95.6	92.6	1.29	1.09	1.16	1.18	0.00836966		1

P13716	ALAD	8	114.3	90.5	134.1	85.7	109.5	65.9	1.33	0.83	2.03	1.40	0.21938311	1
O43583	DENR	7	125.1	92.8	121.4	74.9	107.2	78.6	1.67	0.87	1.54	1.36	0.14367314	1
P27707	DCK	3	98.2	175.7	116.4	101.8	24.3	83.6	0.96	7.23	1.39	3.20	0.14298805	1
P32321	DCTD	5	121.1	110.3	130.4	78.9	89.7	69.6	1.53	1.23	1.87	1.55	0.00740523	1
Q16854	DGUOK	3	64.5	105.3	109.8	135.5	94.7	90.2	0.48	1.11	1.22	0.94	0.5410352	1
Q9BU89	DOHH	2	157.4	115.9	130.6	42.6	84.1	69.4	3.69	1.38	1.88	2.32	0.01571195	1
Q9Y3Z3	SAMHD1	8	83.1	155.8	155.3	116.9	44.2	44.7	0.71	3.52	3.47	2.57	0.13980298	1
Q9Y315	DERA	9	63.4	92.9	46.4	136.6	107.1	153.6	0.46	0.87	0.30	0.54	0.02787373	1
Q14126	DSG2	9	57.1	73.7	54.8	142.9	126.3	145.2	0.40	0.58	0.38	0.45	0.0008233	1
P15924	DSP	4	85.1	62.4	76.5	114.9	137.6	123.5	0.74	0.45	0.62	0.60	0.00563731	1
P60981	DSTN	8	64.1	61.5	44.7	135.9	138.5	155.3	0.47	0.44	0.29	0.40	0.0005499	1
Q9NR28	DIABLO	9	113.7	104.5	108	86.3	95.5	92	1.32	1.09	1.17	1.20	0.00997979	1
P09622	DLD	20	142.5	86.3	85.9	57.5	113.7	114.1	2.48	0.76	0.75	1.33	0.73111178	1
P09417	QDPR	11	62.6	77.1	55.6	137.4	122.9	144.4	0.46	0.63	0.39	0.49	0.00146055	1
Q9NY33	DPP3	19	102.2	96.1	101.6	97.8	103.9	98.4	1.04	0.92	1.03	1.00	0.98178794	1
Q9NZJ9	NUDT4	2	109	88.6	92.7	91	111.4	107.3	1.20	0.80	0.86	0.95	0.50367759	1
Q9P265	DIP2B	9	133.7	144	122.2	66.3	56	77.8	2.02	2.57	1.57	2.05	0.00170842	1
O14672	ADAM10	8	71.1	10	51.9	128.9	190	148.1	0.55	0.05	0.35	0.32	0.01202374	1
Q13443	ADAM9	5	53.5	112.4	94.9	146.5	87.6	105.1	0.37	1.28	0.90	0.85	0.3496501	1
Q12959	DLG1	5	21.1	98.9	103.5	178.9	101.1	96.5	0.12	0.98	1.07	0.72	0.24864863	1
Q2NKX8	ERCC6L	12	178.7	158.3	157	21.3	41.7	43	8.39	3.80	3.65	5.28	0.00020113	1
P18858	LIG1	9	122.1	113.1	90.2	77.9	86.9	109.8	1.57	1.30	0.82	1.23	0.27585682	1
P52701	MSH6	21	117.1	116.1	128	82.9	83.9	72	1.41	1.38	1.78	1.52	0.0016323	1
P09884	POLA1	9	161.3	148.7	169.5	38.7	51.3	30.5	4.17	2.90	5.56	4.21	0.0001515	1
P28340	POLD1	5	108.6	96.7	109.9	91.4	103.3	90.1	1.19	0.94	1.22	1.11	0.16320709	1
Q92878	RAD50	22	145.9	128.5	131.2	54.1	71.5	68.8	2.70	1.80	1.91	2.13	0.00077299	1
P25205	MCM3	26	145.9	131.5	138.3	54.1	68.5	61.7	2.70	1.92	2.24	2.29	0.00019524	1
P27695	APEX1	23	73.1	78.2	67.1	126.9	121.8	132.9	0.58	0.64	0.50	0.57	0.00027719	1
P19387	POLR2C	6	118.8	114.2	108.8	81.2	85.8	91.2	1.46	1.33	1.19	1.33	0.00241835	1
O00411	POLRMT	1	103.3	90.3	77.8	96.7	109.7	122.2	1.07	0.82	0.64	0.84	0.14099883	1
P19388	POLR2E	9	126.2	110.1	142.2	73.8	89.9	57.8	1.71	1.22	2.46	1.80	0.01621825	1
P52434	POLR2H	5	152.1	151.1	118.2	47.9	48.9	81.8	3.18	3.09	1.44	2.57	0.00679807	1
P25685	DNAJB1	11	123.9	81.8	125.9	76.1	118.2	74.1	1.63	0.69	1.70	1.34	0.35870762	1
Q8IXB1	DNAJC10	10	134.1	142.7	129.3	65.9	57.3	70.7	2.03	2.49	1.83	2.12	0.00021736	1
O75165	DNAJC13	7	103.4	144.6	125.4	96.6	55.4	74.6	1.07	2.61	1.68	1.79	0.04380835	1
Q99543	DNAJC2	8	132.4	109.7	122.7	67.6	90.3	77.3	1.96	1.21	1.59	1.59	0.00969583	1
O75937	DNAJC8	10	147.4	100.9	146.7	52.6	99.1	53.3	2.80	1.02	2.75	2.19	0.0436382	1
Q8WXX5	DNAJC9	9	111.1	178.4	109.5	88.9	21.6	90.5	1.25	8.26	1.21	3.57	0.10901013	1
Q5SXM8	DNLZ	2	117.2	105.1	117.8	82.8	94.9	82.2	1.42	1.11	1.43	1.32	0.01026566	1
P04843	RPN1	30	127.8	120.4	129	72.2	79.6	71	1.77	1.51	1.82	1.70	0.00017253	1
P61803	DAD1	3	109.4	104	109.4	90.6	96	90.6	1.21	1.08	1.21	1.17	0.00395177	1

P46977	STT3A	12	129.1	120.8	135.5	70.9	79.2	64.5	1.82	1.53	2.10	1.82	0.00069631	1
Q8TCJ2	STT3B	9	87.3	96.5	102.5	112.7	103.5	97.5	0.77	0.93	1.05	0.92	0.21778892	1
Q9Y673	ALG5	11	68.5	96.9	82.2	131.5	103.1	117.8	0.52	0.94	0.70	0.72	0.03945841	1
P49959	MRE11	13	179.1	131.2	135.8	20.9	68.8	64.2	8.57	1.91	2.12	4.20	0.01070831	1
P55265	ADAR	9	123.6	136.1	134.1	76.4	63.9	65.9	1.62	2.13	2.03	1.93	0.00033699	1
O14972	DSCR3	4	129.3	89.2	88.2	70.7	110.8	111.8	1.83	0.81	0.79	1.14	0.82696159	1
Q9UJU6	DBNL	17	45.8	124	98.7	154.2	76	101.3	0.30	1.63	0.97	0.97	0.55432389	1
Q8TEA8	DTD1	7	113.4	95.3	103.3	86.6	104.7	96.7	1.31	0.91	1.07	1.10	0.34082335	1
P51452	DUSP3	6	57.1	34	57.8	142.9	166	142.2	0.40	0.20	0.41	0.34	0.00080552	1
Q9UJW0	DCTN4	6	132	147.5	113.6	68	52.5	86.4	1.94	2.81	1.31	2.02	0.01099619	1
Q9BTE1	DCTN5	3	132.1	145.9	113.1	67.9	54.1	86.9	1.95	2.70	1.30	1.98	0.01068555	1
O00399	DCTN6	2	120.1	93.1	128.2	79.9	106.9	71.8	1.50	0.87	1.79	1.39	0.13971758	1
Q6XZF7	DNMBP	20	181.9	133.8	180.7	18.1	66.2	19.3	10.05	2.02	9.36	7.14	0.00426986	1
O60313	OPA1	10	156	146.4	139.6	44	53.6	60.4	3.55	2.73	2.31	2.86	0.00014803	1
P63167	DYNLL1	3	126.2	117.9	138.6	73.8	82.1	61.4	1.71	1.44	2.26	1.80	0.00292016	1
Q96FJ2	DYNLL2	2	89.8	94.9	105.2	110.2	105.1	94.8	0.81	0.90	1.11	0.94	0.35247832	1
Q9NP97	DYNLRB1	2	120	109.8	116.8	80	90.2	83.2	1.50	1.22	1.40	1.37	0.00187845	1
P51808	DYNLT3	1	97.4	83.9	81	102.6	116.1	119	0.95	0.72	0.68	0.78	0.02451645	1
Q8IZA0	KIAA0319	3	84.4	88.9	106.2	115.6	111.1	93.8	0.73	0.80	1.13	0.89	0.21953841	1
Q14258	TRIM25	18	130.3	123	140.3	69.7	77	59.7	1.87	1.60	2.35	1.94	0.00092007	1
Q9UKV5	AMFR	2	53.4	92.4	65.7	146.6	107.6	134.3	0.36	0.86	0.49	0.57	0.02227399	1
Q9Y4X5	ARIH1	3	113.3	122	66.9	86.7	78	133.1	1.31	1.56	0.50	1.12	0.95455394	1
Q5VTR2	RNF20	11	120.6	114.3	116.5	79.4	85.7	83.5	1.52	1.33	1.40	1.42	0.00019456	1
Q9UNE7	STUB1	11	109.6	101.7	100.7	90.4	98.3	99.3	1.21	1.03	1.01	1.09	0.11484443	1
Q9ULT8	HECTD1	10	109.1	89.7	104.1	90.9	110.3	95.9	1.20	0.81	1.09	1.03	0.82568947	1
Q7Z6Z7	HUWE1	47	126.1	115	117.9	73.9	85	82.1	1.71	1.35	1.44	1.50	0.00111547	1
P46934	NEDD4	6	123.1	97	101.3	76.9	103	98.7	1.60	0.94	1.03	1.19	0.27988851	1
Q63HN8	RNF213	30	95.8	85.1	93.1	104.2	114.9	106.9	0.92	0.74	0.87	0.84	0.01885768	1
Q9UPN9	TRIM33	4	129.1	123.6	138.5	70.9	76.4	61.5	1.82	1.62	2.25	1.90	0.00058819	1
Q14669	TRIP12	7	115.1	115.8	130.3	84.9	84.2	69.7	1.36	1.38	1.87	1.53	0.00433056	1
Q5T4S7	UBR4	22	155.5	112.1	150	44.5	87.9	50	3.49	1.28	3.00	2.59	0.01530451	1
O95071	UBR5	19	153.5	138.3	152.4	46.5	61.7	47.6	3.30	2.24	3.20	2.91	0.00015574	1
Q96JP5	ZFP91	1	96.1	94.5	101.7	103.9	105.5	98.3	0.92	0.90	1.03	0.95	0.17165694	1
O00423	EML1	1	111	64.7	104.7	89	135.3	95.3	1.25	0.48	1.10	0.94	0.55857813	1
O95834	EML2	6	105.7	129.4	63.4	94.3	70.6	136.6	1.12	1.83	0.46	1.14	0.97253386	1
Q9HC35	EML4	19	103.5	83.3	95.6	96.5	116.7	104.4	1.07	0.71	0.92	0.90	0.23088845	1
Q96C19	EFHD2	10	116	124	109.6	84	76	90.4	1.38	1.63	1.21	1.41	0.0049497	1
Q14657	LAGE3	3	157.5	121.5	156.2	42.5	78.5	43.8	3.71	1.55	3.57	2.94	0.00566923	1
Q15717	ELAVL1	13	124.2	105.3	121.9	75.8	94.7	78.1	1.64	1.11	1.56	1.44	0.01522671	1
P13804	ETFA	19	59.8	73	59.7	140.2	127	140.3	0.43	0.57	0.43	0.48	0.00032937	1
P38117	ETFB	21	59.5	76.1	65.9	140.5	123.9	134.1	0.42	0.61	0.49	0.51	0.00065645	1

Q05639	EEF1A2	12	85.5	102.1	100.1	114.5	97.9	99.9	0.75	1.04	1.00	0.93	0.32990448		1
P24534	EEF1B2	6	105.6	104.6	101.8	94.4	95.4	98.2	1.12	1.10	1.04	1.08	0.00762891		1
P29692	EEF1D	16	110	96.5	103.5	90	103.5	96.5	1.22	0.93	1.07	1.08	0.29311771		1
P26641	EEF1G	24	135.1	118.9	126.1	64.9	81.1	73.9	2.08	1.47	1.71	1.75	0.0012882		1
Q96RP9	GFM1	15	94.9	89.1	91.7	105.1	110.9	108.3	0.90	0.80	0.85	0.85	0.002404		1
Q9NYP7	ELOVL5	1	134.3	139.4	154.1	65.7	60.6	45.9	2.04	2.30	3.36	2.57	0.0005305		1
Q15370	ELOB	10	125.4	119.5	132.3	74.6	80.5	67.7	1.68	1.48	1.95	1.71	0.00059844		1
Q15369	ELOC	3	130.1	118.6	127	69.9	81.4	73	1.86	1.46	1.74	1.69	0.00048492		1
P50402	EMD	13	92.9	63.8	107.6	107.1	136.2	92.4	0.87	0.47	1.16	0.83	0.26108375		1
Q99961	SH3GL1	10	87.8	161.8	149.6	112.2	38.2	50.4	0.78	4.24	2.97	2.66	0.11074725		1
Q7Z2K6	ERMP1	7	65.9	54	53.3	134.1	146	146.7	0.49	0.37	0.36	0.41	0.00012731		1
P30040	ERP29	15	103.8	82.5	94.5	96.2	117.5	105.5	1.08	0.70	0.90	0.89	0.2159997		1
Q969X5	ERGIC1	10	56.8	116.6	58.3	143.2	83.4	141.7	0.40	1.40	0.41	0.74	0.17731559		1
Q9UPY3	DICER1	12	168.9	166.5	147.7	31.1	33.5	52.3	5.43	4.97	2.82	4.41	0.00020967		1
Q53H82	LACTB2	19	94.3	79.9	89.6	105.7	120.1	110.4	0.89	0.67	0.81	0.79	0.01579778		1
Q9UNN8	PROCR	5	46.4	82.4	61	153.6	117.6	139	0.30	0.70	0.44	0.48	0.00765524		1
Q96JJ3	ELMO2	3	126.7	107.6	119.7	73.3	92.4	80.3	1.73	1.16	1.49	1.46	0.01031584		1
P84090	ERH	3	141.3	169.6	148.9	58.7	30.4	51.1	2.41	5.58	2.91	3.63	0.0008777		1
Q9UHY7	ENOPH1	5	95.8	74	109.9	104.2	126	90.1	0.92	0.59	1.22	0.91	0.41131163		1
P42126	ECI1	9	132	81.1	112.2	68	118.9	87.8	1.94	0.68	1.28	1.30	0.46589476		1
O75521	ECI2	7	129	112.9	121.2	71	87.1	78.8	1.82	1.30	1.54	1.55	0.00306256		1
P30084	ECHS1	16	87.5	79.4	89.1	112.5	120.6	110.9	0.78	0.66	0.80	0.75	0.00230297		1
Q12929	EPS8	14	73	70.4	92	127	129.6	108	0.57	0.54	0.85	0.66	0.01104939		1
P42566	EPS15	10	124.8	118.6	143.8	75.2	81.4	56.2	1.66	1.46	2.56	1.89	0.00560836		1
Q9Y6I3	EPN1	10	59.5	45.8	61.8	140.5	154.2	138.2	0.42	0.30	0.45	0.39	0.00023238		1
O95208	EPN2	5	130.1	121.8	143.9	69.9	78.2	56.1	1.86	1.56	2.57	1.99	0.00218416		1
Q99808	SLC29A1	4	192.8	138.3	156.4	7.2	61.7	43.6	26.78	2.24	3.59	10.87	0.00527459		1
Q8N766	EMC1	11	122.3	114.2	113	77.7	85.8	87	1.57	1.33	1.30	1.40	0.00133047		1
Q15006	EMC2	8	90.3	114.8	99.8	109.7	85.2	100.2	0.82	1.35	1.00	1.06	0.76224963		1
Q9P0I2	EMC3	6	100.7	112.7	88	99.3	87.3	112	1.01	1.29	0.79	1.03	0.93071412		1
Q96HE7	ERO1A	20	83.4	78.3	87.3	116.6	121.7	112.7	0.72	0.64	0.77	0.71	0.00076695		1
P27105	STOM	17	89.2	38.2	91.1	110.8	161.8	108.9	0.81	0.24	0.84	0.63	0.0908616		1
P30042	C21orf33	10	131.5	76	127.9	68.5	124	72.1	1.92	0.61	1.77	1.44	0.40468941		1
Q9H0W9	C11orf54	4	96	60	76.4	104	140	123.6	0.92	0.43	0.62	0.66	0.03757156		1
Q8WZ82	OVCA2	5	68.5	93.5	84.7	131.5	106.5	115.3	0.52	0.88	0.73	0.71	0.02649218		1
Q8NBQ5	HSD17B11	15	74	81.8	45.2	126	118.2	154.8	0.59	0.69	0.29	0.52	0.01377282		1
Q9NTX5	ECHDC1	10	114.7	132.5	112.4	85.3	67.5	87.6	1.34	1.96	1.28	1.53	0.01147969		1
P15170	GSPT1	8	95	90.2	97.6	105	109.8	102.4	0.90	0.82	0.95	0.89	0.02010536		1
O00303	EIF3F	11	127.9	120.5	119	72.1	79.5	81	1.77	1.52	1.47	1.59	0.00032098		1
O15372	EIF3H	14	133.3	123.5	129.3	66.7	76.5	70.7	2.00	1.61	1.83	1.81	0.00014019		1
Q7L2H7	EIF3M	13	108.5	120.3	127.6	91.5	79.7	72.4	1.19	1.51	1.76	1.49	0.00878819		1

P78344	EIF4G2	32	130	121.5	134.8	70	78.5	65.2	1.86	1.55	2.07	1.82	0.00047174	1
P23588	EIF4B	18	129.4	116.5	120.8	70.6	83.5	79.2	1.83	1.40	1.53	1.58	0.00115524	1
P06730	EIF4E	6	116.6	89.9	102.4	83.4	110.1	97.6	1.40	0.82	1.05	1.09	0.61536682	1
O60573	EIF4E2	8	100	113.5	81.1	100	86.5	118.9	1.00	1.31	0.68	1.00	0.79986247	1
Q15056	EIF4H	11	147.5	105.5	152.4	52.5	94.5	47.6	2.81	1.12	3.20	2.38	0.02888385	1
P56537	EIF6	9	136.4	119.9	138.3	63.6	80.1	61.7	2.14	1.50	2.24	1.96	0.00158222	1
Q5RKV6	EXOSC6	9	136.7	113.3	157	63.3	86.7	43	2.16	1.31	3.65	2.37	0.0161966	1
Q13868	EXOSC2	10	132	92.3	141.3	68	107.7	58.7	1.94	0.86	2.41	1.74	0.10867896	1
Q9NQT5	EXOSC3	7	132.1	116.6	137.9	67.9	83.4	62.1	1.95	1.40	2.22	1.85	0.00302392	1
Q9NPD3	EXOSC4	5	143.8	102.8	119.8	56.2	97.2	80.2	2.56	1.06	1.49	1.70	0.05807072	1
Q15024	EXOSC7	10	132.6	101.7	141.4	67.4	98.3	58.6	1.97	1.03	2.41	1.80	0.04137144	1
Q96B26	EXOSC8	8	137.1	112	137.9	62.9	88	62.1	2.18	1.27	2.22	1.89	0.0085033	1
Q9NQT4	EXOSC5	6	123.1	104.5	167.4	76.9	95.5	32.6	1.60	1.09	5.13	2.61	0.07431771	1
Q9Y2L1	DIS3	6	100.2	122.9	139.6	99.8	77.1	60.4	1.00	1.59	2.31	1.64	0.06076643	1
O14980	XPO1	28	115.6	111.1	118.7	84.4	88.9	81.3	1.37	1.25	1.46	1.36	0.0006322	1
Q9UIA9	XPO7	10	89.2	95.3	81.4	110.8	104.7	118.6	0.81	0.91	0.69	0.80	0.01618157	1
O43592	XPOT	14	146.4	121.5	129.2	53.6	78.5	70.8	2.73	1.55	1.82	2.03	0.00340332	1
Q9Y5B9	SUPT16H	5	76	85	78	124	115	122	0.61	0.74	0.64	0.66	0.00045845	1
Q08945	SSRP1	4	78	73.6	108.7	122	126.4	91.3	0.64	0.58	1.19	0.80	0.16528588	1
P47755	CAPZA2	11	93	107.3	100.3	107	92.7	99.7	0.87	1.16	1.01	1.01	0.94866606	1
Q5VZK9	CARMIL1	5	87.2	100.2	111.3	112.8	99.8	88.7	0.77	1.00	1.25	1.01	0.9341095	1
Q96AE4	FUBP1	23	117.6	112	118.1	82.4	88	81.9	1.43	1.27	1.44	1.38	0.00032644	1
P14324	FDPS	11	112.4	137.6	100.9	87.6	62.4	99.1	1.28	2.21	1.02	1.50	0.09121948	1
P49327	FASN	121	131.2	122.8	131.6	68.8	77.2	68.4	1.91	1.59	1.92	1.81	0.00014829	1
Q0JRZ9	FCHO2	3	93.8	112.6	134.2	106.2	87.4	65.8	0.88	1.29	2.04	1.40	0.17639739	1
Q96EF6	FBXO17	5	132.2	182	168.9	67.8	18	31.1	1.95	10.11	5.43	5.83	0.00441908	1
Q9NRD1	FBXO6	6	154.1	122.3	166	45.9	77.7	34	3.36	1.57	4.88	3.27	0.00676151	1
Q96ME1	FBXL18	5	156	150.3	173.4	44	49.7	26.6	3.55	3.02	6.52	4.36	0.00025972	1
Q96AC1	FERMT2	7	129	79.9	107.4	71	120.1	92.6	1.82	0.67	1.16	1.21	0.61734508	1
Q9Y613	FHOD1	6	129.1	147.1	150.7	70.9	52.9	49.3	1.82	2.78	3.06	2.55	0.00086079	1
Q9Y2H6	FNDC3A	2	83.5	49.3	68.5	116.5	150.7	131.5	0.72	0.33	0.52	0.52	0.00930179	1
P21333	FLNA	141	103.3	96	102.9	96.7	104	97.1	1.07	0.92	1.06	1.02	0.6842097	1
Q5T1M5	FKBP15	18	122.3	117.4	139.1	77.7	82.6	60.9	1.57	1.42	2.28	1.76	0.00482348	1
Q8N3X1	FNBP4	2	119	78.7	78.8	81	121.3	121.2	1.47	0.65	0.65	0.92	0.45538064	1
Q13642	FHL1	12	186.9	147.4	182.2	13.1	52.6	17.8	14.27	2.80	10.24	9.10	0.00120923	1
Q14192	FHL2	10	99	113.2	93.2	101	86.8	106.8	0.98	1.30	0.87	1.05	0.69036006	1
P51114	FXR1	8	147.8	138.4	134.3	52.2	61.6	65.7	2.83	2.25	2.04	2.37	0.00014221	1
P51116	FXR2	5	127.8	137.3	123	72.2	62.7	77	1.77	2.19	1.60	1.85	0.00058791	1
O60353	FZD6	1	121.5	62.7	84.4	78.5	137.3	115.6	1.55	0.46	0.73	0.91	0.43718221	1
Q9H479	FN3K	4	64.6	131.5	47.5	135.4	68.5	152.5	0.48	1.92	0.31	0.90	0.35816239	1
Q9NQ88	TIGAR	11	96.6	99.6	100.8	103.4	100.4	99.2	0.93	0.99	1.02	0.98	0.32079244	1

P04075	ALDOA	28	89.3	81.3	90.4	110.7	118.7	109.6	0.81	0.68	0.82	0.77	0.00304132	1
P09972	ALDOC	4	98.2	84.8	92.3	101.8	115.2	107.7	0.96	0.74	0.86	0.85	0.03982627	1
P07954	FH	13	154.8	119.1	134.2	45.2	80.9	65.8	3.42	1.47	2.04	2.31	0.0078995	1
Q96GK7	FAHD2A	10	79.9	74.4	64.9	120.1	125.6	135.1	0.67	0.59	0.48	0.58	0.00096364	1
Q9BQS8	FYCO1	6	116.7	115.7	107.1	83.3	84.3	92.9	1.40	1.37	1.15	1.31	0.00362987	1
P09382	LGALS1	9	154.3	136.2	154	45.7	63.8	46	3.38	2.13	3.35	2.95	0.00033966	1
O95166	GABARAI	1	88.6	75.8	92.6	111.4	124.2	107.4	0.80	0.61	0.86	0.76	0.01612022	1
Q92820	GGH	10	74.4	65.9	96	125.6	134.1	104	0.59	0.49	0.92	0.67	0.02852429	1
O75223	GGCT	10	119	120.2	107.7	81	79.8	92.3	1.47	1.51	1.17	1.38	0.00514829	1
P19440	GGT1	9	9.2	39	18	190.8	161	182	0.05	0.24	0.10	0.13	0.00023797	1
Q12789	GTF3C1	8	113	94.6	84.1	87	105.4	115.9	1.30	0.90	0.73	0.97	0.66726367	1
P13984	GTF2F2	6	133.3	119.4	150.4	66.7	80.6	49.6	2.00	1.48	3.03	2.17	0.0056118	1
O60763	USO1	11	129.9	105.8	111.5	70.1	94.2	88.5	1.85	1.12	1.26	1.41	0.03766782	1
Q9NQX3	GPHN	4	106.2	117.2	117.5	93.8	82.8	82.5	1.13	1.42	1.42	1.32	0.0065784	1
P53611	RABGGTI	3	148.1	116.8	174.1	51.9	83.2	25.9	2.85	1.40	6.72	3.66	0.01673747	1
Q3V6T2	CCDC88A	8	157.5	177.1	180.5	42.5	22.9	19.5	3.71	7.73	9.26	6.90	0.00014493	1
P60983	GMFB	6	104.4	88.7	86.4	95.6	111.3	113.6	1.09	0.80	0.76	0.88	0.16269594	1
P04150	NR3C1	2	74.1	93.8	89.8	125.9	106.2	110.2	0.59	0.88	0.81	0.76	0.02946634	1
P46926	GNPDA1	11	125.7	89.7	124.9	74.3	110.3	75.1	1.69	0.81	1.66	1.39	0.18471453	1
Q8TDQ7	GNPDA2	6	163.4	128.1	169.1	36.6	71.9	30.9	4.46	1.78	5.47	3.91	0.00411817	1
Q6PCE3	PGM2L1	3	81.5	48.4	30.9	118.5	151.6	169.1	0.69	0.32	0.18	0.40	0.01148568	1
P06744	GPI	34	92.8	72.7	81.1	107.2	127.3	118.9	0.87	0.57	0.68	0.71	0.01245684	1
P00367	GLUD1	31	93.4	106.5	111.6	106.6	93.5	88.4	0.88	1.14	1.26	1.09	0.37384415	1
O94925	GLS	19	1.9	42.7	36.5	198.1	157.3	163.5	0.01	0.27	0.22	0.17	0.00124546	1
Q06210	GFPT1	19	81.4	78.2	84.6	118.6	121.8	115.4	0.69	0.64	0.73	0.69	0.00014133	1
Q2TAL8	QRICH1	4	136.5	128.7	143.9	63.5	71.3	56.1	2.15	1.81	2.57	2.17	0.00030318	1
P47897	QARS	25	115.5	105	128.1	84.5	95	71.9	1.37	1.11	1.78	1.42	0.02651314	1
P35754	GLRX	3	3.8	19	39.4	196.2	181	160.6	0.02	0.10	0.25	0.12	0.00040664	1
P21266	GSTM3	4	147.4	44	134.2	52.6	156	65.8	2.80	0.28	2.04	1.71	0.72915738	1
P78417	GSTO1	19	101.6	103.4	102.7	98.4	96.6	97.3	1.03	1.07	1.06	1.05	0.00227771	1
P09211	GSTP1	13	89.2	66.6	32.5	110.8	133.4	167.5	0.81	0.50	0.19	0.50	0.03305198	1
P04406	GAPDH	35	108	94.6	108.7	92	105.4	91.3	1.17	0.90	1.19	1.09	0.31017056	1
Q8N335	GPD1L	7	65.3	88.8	64.3	134.7	111.2	135.7	0.48	0.80	0.47	0.59	0.00861542	1
P43304	GPD2	38	104.8	94.4	106.1	95.2	105.6	93.9	1.10	0.89	1.13	1.04	0.53678681	1
A6NDG6	PGP	12	64.9	94.7	57.5	135.1	105.3	142.5	0.48	0.90	0.40	0.59	0.02635832	1
P23434	GCSH	1	98.9	72.2	86	101.1	127.8	114	0.98	0.56	0.75	0.77	0.05859613	1
P23378	GLDC	13	141.1	156.6	156.8	58.9	43.4	43.2	2.40	3.61	3.63	3.21	0.00015079	1
P06737	PYGL	22	103.1	105.2	95	96.9	94.8	105	1.06	1.11	0.90	1.03	0.64316339	1
P46976	GYG1	8	147	162.6	166.9	53	37.4	33.1	2.77	4.35	5.04	4.05	0.00016151	1
P30419	NMT1	15	106.2	101.9	115.7	93.8	98.1	84.3	1.13	1.04	1.37	1.18	0.05127156	1
Q9UBQ7	GRHPR	16	89.9	79.9	84.8	110.1	120.1	115.2	0.82	0.67	0.74	0.74	0.00176676	1

Q92896	GLG1	34	95.7	86.2	97.8	104.3	113.8	102.2	0.92	0.76	0.96	0.88	0.0551348	1
O00461	GOLIM4	6	113	113.2	123.9	87	86.8	76.1	1.30	1.30	1.63	1.41	0.00279381	1
Q9H4A6	GOLPH3	5	108.7	99	113.9	91.3	101	86.1	1.19	0.98	1.32	1.16	0.08006729	1
Q9H3P7	ACBD3	12	82.3	77.4	69.2	117.7	122.6	130.8	0.70	0.63	0.53	0.62	0.00093169	1
O95249	GOSR1	8	102.8	69.3	52.5	97.2	130.7	147.5	1.06	0.53	0.36	0.65	0.07402668	1
Q7L5D6	GET4	8	145.5	130.6	161.9	54.5	69.4	38.1	2.67	1.88	4.25	2.93	0.00197521	1
Q92805	GOLGA1	8	95.3	100.9	119.9	104.7	99.1	80.1	0.91	1.02	1.50	1.14	0.36560279	1
Q08378	GOLGA3	15	108.5	102.5	96.9	91.5	97.5	103.1	1.19	1.05	0.94	1.06	0.32850128	1
Q13439	GOLGA4	25	90.3	83	85	109.7	117	115	0.82	0.71	0.74	0.76	0.00083458	1
Q8TBA6	GOLGA5	14	128.8	141.2	116.2	71.2	58.8	83.8	1.81	2.40	1.39	1.87	0.00489487	1
Q14789	GOLGB1	67	96.6	96.5	101.6	103.4	103.5	98.4	0.93	0.93	1.03	0.97	0.21196863	1
Q96S52	PIGS	3	92.9	86.5	88	107.1	113.5	112	0.87	0.76	0.79	0.81	0.00135428	1
Q96CP6	GRAMD1A	5	9.2	124.2	22.1	190.8	75.8	177.9	0.05	1.64	0.12	0.60	0.13441284	1
P28676	GCA	7	179.3	105.2	144.1	20.7	94.8	55.9	8.66	1.11	2.58	4.12	0.04720743	1
P62993	GRB2	16	110	89.7	105.1	90	110.3	94.9	1.22	0.81	1.11	1.05	0.73015304	1
Q9H3K2	GHITM	3	110.8	101.8	112.6	89.2	98.2	87.4	1.24	1.04	1.29	1.19	0.02367071	1
Q9UIJ7	AK3	16	68.3	109.2	71.5	131.7	90.8	128.5	0.52	1.20	0.56	0.76	0.141114	1
P01116	KRAS	2	101.9	124.9	70.8	98.1	75.1	129.2	1.04	1.66	0.55	1.08	0.94592804	1
P01111	NRAS	5	117.1	150.4	104	82.9	49.6	96	1.41	3.03	1.08	1.84	0.07117382	1
P62826	RAN	16	117.6	110.5	120.4	82.4	89.5	79.6	1.43	1.23	1.51	1.39	0.00148643	1
Q15382	RHEB	11	127.4	126.4	80.2	72.6	73.6	119.8	1.75	1.72	0.67	1.38	0.36144562	1
Q9NR31	SAR1A	5	137.2	103	130.4	62.8	97	69.6	2.18	1.06	1.87	1.71	0.03340737	1
Q9Y6B6	SAR1B	6	123	144.2	130.3	77	55.8	69.7	1.60	2.58	1.87	2.02	0.00178624	1
Q14353	GAMT	6	70.2	114.3	68.9	129.8	85.7	131.1	0.54	1.33	0.53	0.80	0.214944	1
P04899	GNAI2	4	150.2	175.7	115.4	49.8	24.3	84.6	3.02	7.23	1.36	3.87	0.01891599	1
Q9UBI6	GNG12	6	108.4	98.9	107.7	91.6	101.1	92.3	1.18	0.98	1.17	1.11	0.0817345	1
P62873	GNB1	8	95.3	123.1	98.4	104.7	76.9	101.6	0.91	1.60	0.97	1.16	0.41881641	1
P62879	GNB2	7	89.5	120.5	109.5	110.5	79.5	90.5	0.81	1.52	1.21	1.18	0.36832526	1
P08754	GNAI3	3	105.4	129.3	131.5	94.6	70.7	68.5	1.11	1.83	1.92	1.62	0.02022616	1
Q5JWF2	GNAS	5	102.5	101.4	76.9	97.5	98.6	123.1	1.05	1.03	0.62	0.90	0.33966296	1
Q9HAV0	GNB4	6	136.7	171.1	111.7	63.3	28.9	88.3	2.16	5.92	1.27	3.11	0.0307427	1
Q9NPE3	NOP10	1	129.1	119.3	85.7	70.9	80.7	114.3	1.82	1.48	0.75	1.35	0.28838027	1
Q9BSH5	HDHD3	6	67.6	66.5	93.5	132.4	133.5	106.5	0.51	0.50	0.88	0.63	0.01801593	1
Q9P2D3	HEATR5B	5	101.4	96.3	104.8	98.6	103.7	95.2	1.03	0.93	1.10	1.02	0.6581738	1
P0DMV9	HSPA1B	29	84.8	80.5	88	115.2	119.5	112	0.74	0.67	0.79	0.73	0.0005342	1
Q00613	HSF1	2	110.4	102	116	89.6	98	84	1.23	1.04	1.38	1.22	0.03019347	1
Q12931	TRAP1	23	107.3	101	118.2	92.7	99	81.8	1.16	1.02	1.44	1.21	0.06773904	1
P54652	HSPA2	18	53.9	33.9	60.2	146.1	166.1	139.8	0.37	0.20	0.43	0.33	0.0008303	1
Q9H910	HN1L	9	115.9	106.9	114.4	84.1	93.1	85.6	1.38	1.15	1.34	1.29	0.00324601	1
P09601	HMOX1	18	53.6	89.1	47.2	146.4	110.9	152.8	0.37	0.80	0.31	0.49	0.01636362	1
P30519	HMOX2	13	142.3	120	119.8	57.7	80	80.2	2.47	1.50	1.49	1.82	0.00659184	1

Q5SSJ5	HP1BP3	4	74.4	118.6	105.4	125.6	81.4	94.6	0.59	1.46	1.11	1.05	0.95684701	1
Q99729	HNRNPAE	10	134.3	136.5	124.1	65.7	63.5	75.9	2.04	2.15	1.64	1.94	0.000304	1
Q13151	HNRNPA0	15	90.4	104.7	93.7	109.6	95.3	106.3	0.82	1.10	0.88	0.93	0.28902168	1
P51991	HNRNPA3	22	115.7	145.4	114.4	84.3	54.6	85.6	1.37	2.66	1.34	1.79	0.02454644	1
Q14103	HNRNPD	18	110.8	144.1	137.5	89.2	55.9	62.5	1.24	2.58	2.20	2.01	0.0128598	1
P52597	HNRNPF	12	138.1	123.2	132.5	61.9	76.8	67.5	2.23	1.60	1.96	1.93	0.00052525	1
P31943	HNRNPH1	13	120.9	144.1	154.8	79.1	55.9	45.2	1.53	2.58	3.42	2.51	0.00485135	1
P31942	HNRNPH3	16	123.7	107.6	129.4	76.3	92.4	70.6	1.62	1.16	1.83	1.54	0.01184092	1
Q8WVV9	HNRNPLL	10	104.5	126.6	132.6	95.5	73.4	67.4	1.09	1.72	1.97	1.60	0.0245709	1
Q1KMD3	HNRNPUL	24	133.2	120.5	132.7	66.8	79.5	67.3	1.99	1.52	1.97	1.83	0.00060569	1
P22626	HNRNPA2	26	102.9	100.2	102.6	97.1	99.8	97.4	1.06	1.00	1.05	1.04	0.03468367	1
P19367	HK1	18	67.9	62.7	74.9	132.1	137.3	125.1	0.51	0.46	0.60	0.52	0.00022814	1
P52789	HK2	7	134.3	184.6	184.2	65.7	15.4	15.8	2.04	11.99	11.66	8.56	0.00458473	1
P09429	HMGB1	16	123.4	131.5	121.2	76.6	68.5	78.8	1.61	1.92	1.54	1.69	0.00033146	1
P49773	HINT1	6	109	98.1	101.3	91	101.9	98.7	1.20	0.96	1.03	1.06	0.28806224	1
Q9BX68	HINT2	3	106.4	87.1	111.7	93.6	112.9	88.3	1.14	0.77	1.27	1.06	0.75940652	1
P12081	HARS	21	143.6	131.3	149.4	56.4	68.7	50.6	2.55	1.91	2.95	2.47	0.00039081	1
Q92769	HDAC2	4	123.1	127.5	146.3	76.9	72.5	53.7	1.60	1.76	2.72	2.03	0.00302449	1
Q9UBN7	HDAC6	6	104.9	105.8	109.1	95.1	94.2	90.9	1.10	1.12	1.20	1.14	0.00186197	1
O00422	SAP18	5	163.1	153.6	132.2	36.9	46.4	67.8	4.42	3.31	1.95	3.23	0.00154468	1
P10412	HIST1H1E	7	56	109.7	55.8	144	90.3	144.2	0.39	1.21	0.39	0.66	0.1080255	1
Q92522	H1FX	6	86.6	111.3	68	113.4	88.7	132	0.76	1.25	0.52	0.84	0.26916774	1
P0C0S8	HIST1H2A	2	115.2	118.5	123.5	84.8	81.5	76.5	1.36	1.45	1.61	1.48	0.0003649	1
Q93077	HIST1H2A	1	140.8	150.8	104.4	59.2	49.2	95.6	2.38	3.07	1.09	2.18	0.0325933	1
P0C0S5	H2AFZ	1	139.1	120.2	130.8	60.9	79.8	69.2	2.28	1.51	1.89	1.89	0.00148215	1
O60814	HIST1H2E	2	127	71.3	123.6	73	128.7	76.4	1.74	0.55	1.62	1.30	0.59750705	1
P68431	HIST1H3A	1	131.2	117.4	111.9	68.8	82.6	88.1	1.91	1.42	1.27	1.53	0.00766172	1
P62805	HIST1H4A	10	124	116.6	113.5	76	83.4	86.5	1.63	1.40	1.31	1.45	0.00121181	1
Q86X55	CARM1	7	143.6	132.8	152.8	56.4	67.2	47.2	2.55	1.98	3.24	2.59	0.00045867	1
Q16576	RBBP7	6	116.8	134.4	106.3	83.2	65.6	93.7	1.40	2.05	1.13	1.53	0.02975071	1
O43719	HTATSF1	10	76.5	71.9	85.1	123.5	128.1	114.9	0.62	0.56	0.74	0.64	0.00126055	1
P18462	HLA-A	2	143.2	125.3	113.9	56.8	74.7	86.1	2.52	1.68	1.32	1.84	0.0103763	1
P42858	HTT	7	127.4	177.4	161.4	72.6	22.6	38.6	1.75	7.85	4.18	4.60	0.00602791	1
Q16836	HADH	12	95.2	80.6	107.2	104.8	119.4	92.8	0.91	0.68	1.16	0.91	0.35625219	1
Q16775	HAGH	9	52.7	76.4	66.4	147.3	123.6	133.6	0.36	0.62	0.50	0.49	0.00200202	1
P35914	HMGCL	8	78.3	67.9	137.9	121.7	132.1	62.1	0.64	0.51	2.22	1.13	0.74837682	1
P00492	HPRT1	13	120.5	95	118.8	79.5	105	81.2	1.52	0.90	1.46	1.29	0.12094992	1
Q9Y5U9	IER3IP1	1	106.3	81.6	51	93.7	118.4	149	1.13	0.69	0.34	0.72	0.14609344	1
Q14974	KPNB1	32	133.7	124.5	133	66.3	75.5	67	2.02	1.65	1.99	1.88	0.00013013	1
Q8TEX9	IPO4	17	114	107.6	101.7	86	92.4	98.3	1.33	1.16	1.03	1.17	0.03648416	1
O95373	IPO7	26	103.2	99.9	106.5	96.8	100.1	93.5	1.07	1.00	1.14	1.07	0.07638197	1

Q96P70	IPO9	8	96.6	100.4	100.6	103.4	99.6	99.4	0.93	1.01	1.01	0.98	0.4336727	1
Q9H2U2	PPA2	16	92.9	70.3	91.8	107.1	129.7	108.2	0.87	0.54	0.85	0.75	0.04485673	1
Q15181	PPA1	17	106.2	98.1	103.1	93.8	101.9	96.9	1.13	0.96	1.06	1.05	0.2133841	1
Q9BY32	ITPA	7	115.2	77.6	106.2	84.8	122.4	93.8	1.36	0.63	1.13	1.04	0.9688202	1
P12268	IMPDH2	25	69.8	143.1	149	130.2	56.9	51	0.54	2.51	2.92	1.99	0.31588036	1
Q14573	ITPR3	4	132.8	115.1	79.4	67.2	84.9	120.6	1.98	1.36	0.66	1.33	0.45853548	1
O43314	PPIP5K2	3	139.4	150.1	164	60.6	49.9	36	2.30	3.01	4.56	3.29	0.00052829	1
P29218	IMPA1	12	149.2	109.2	152.6	50.8	90.8	47.4	2.94	1.20	3.22	2.45	0.01985898	1
P14735	IDE	18	79.3	88.6	60.5	120.7	111.4	139.5	0.66	0.80	0.43	0.63	0.01505059	1
Q9Y6M1	IGF2BP2	11	191.3	137.9	97.7	8.7	62.1	102.3	21.99	2.22	0.96	8.39	0.09196761	1
P17301	ITGA2	23	67.6	56.2	72.7	132.4	143.8	127.3	0.51	0.39	0.57	0.49	0.00056132	1
P26006	ITGA3	24	108	102.8	110	92	97.2	90	1.17	1.06	1.22	1.15	0.01026469	1
P23229	ITGA6	19	153.7	122.7	155.3	46.3	77.3	44.7	3.32	1.59	3.47	2.79	0.00425544	1
P05556	ITGB1	28	91.7	86.9	91.6	108.3	113.1	108.4	0.85	0.77	0.85	0.82	0.000892	1
P05362	ICAM1	6	138.6	121.7	128.3	61.4	78.3	71.7	2.26	1.55	1.79	1.87	0.00105363	1
P15260	IFNGR1	2	59.3	26.9	65.3	140.7	173.1	134.7	0.42	0.16	0.48	0.35	0.0042075	1
Q8IU81	IRF2BP1	4	79.5	71	101.6	120.5	129	98.4	0.66	0.55	1.03	0.75	0.06850385	1
P80217	IFI35	9	81.4	63.2	83.2	118.6	136.8	116.8	0.69	0.46	0.71	0.62	0.00597266	1
P13164	IFITM1	3	74	41.3	77.7	126	158.7	122.3	0.59	0.26	0.64	0.49	0.01205223	1
O75569	PRKRA	8	67.1	94	57.6	132.9	106	142.4	0.50	0.89	0.40	0.60	0.02454128	1
Q9NZM3	ITSN2	7	105.1	117.5	144.4	94.9	82.5	55.6	1.11	1.42	2.60	1.71	0.05282895	1
Q27J81	INF2	18	95.6	91.2	104.9	104.4	108.8	95.1	0.92	0.84	1.10	0.95	0.38746589	1
Q2TAA2	IAH1	8	23.4	71.4	51.3	176.6	128.6	148.7	0.13	0.56	0.34	0.34	0.00645881	1
Q9NSE4	IARS2	19	95.8	94	101.8	104.2	106	98.2	0.92	0.89	1.04	0.95	0.16838752	1
Q13907	IDI1	13	76.7	103.7	82.9	123.3	96.3	117.1	0.62	1.08	0.71	0.80	0.10147856	1
Q9Y624	F11R	3	39.4	50.3	73.9	160.6	149.7	126.1	0.25	0.34	0.59	0.39	0.00321577	1
Q9BWU0	SLC4A1AI	7	108.6	109.2	97.3	91.4	90.8	102.7	1.19	1.20	0.95	1.11	0.13974881	1
Q14533	KRT81	8	60.5	82.6	43.8	139.5	117.4	156.2	0.43	0.70	0.28	0.47	0.00900532	1
Q9HA64	FN3KRP	14	37.2	64.1	67.9	162.8	135.9	132.1	0.23	0.47	0.51	0.40	0.00309417	1
Q96EK5	KIF1BP	4	143.5	162.4	129.1	56.5	37.6	70.9	2.54	4.32	1.82	2.89	0.00273038	1
Q86UP2	KTN1	40	90.3	79.6	89.3	109.7	120.4	110.7	0.82	0.66	0.81	0.76	0.00487577	1
Q9H0B6	KLC2	8	72.8	50.2	57.1	127.2	149.8	142.9	0.57	0.34	0.40	0.44	0.0010733	1
Q9NSK0	KLC4	5	138.9	157.8	162.5	61.1	42.2	37.5	2.27	3.74	4.33	3.45	0.0004817	1
O00139	KIF2A	7	131.9	141.1	153.9	68.1	58.9	46.1	1.94	2.40	3.34	2.56	0.00072036	1
O15066	KIF3B	3	151.5	100	146.4	48.5	100	53.6	3.12	1.00	2.73	2.29	0.04797498	1
O14777	NDC80	8	170.6	187.3	114.4	29.4	12.7	85.6	5.80	14.75	1.34	7.30	0.02113775	1
O43291	SPINT2	7	49.1	113.6	79.5	150.9	86.4	120.5	0.33	1.31	0.66	0.77	0.21740993	1
Q04760	GLO1	11	154.5	144.5	164.3	45.5	55.5	35.7	3.40	2.60	4.60	3.53	0.00017586	1
P42167	TMPO	2	112.2	107.4	104.1	87.8	92.6	95.9	1.28	1.16	1.09	1.17	0.0089647	1
Q14739	LBR	4	114.4	93.7	116.6	85.6	106.3	83.4	1.34	0.88	1.40	1.21	0.18566578	1
P20700	LMNB1	23	132.5	150.6	124.8	67.5	49.4	75.2	1.96	3.05	1.66	2.22	0.00265254	1

O15230	LAMA5	23	53.8	57.3	72.1	146.2	142.7	127.9	0.37	0.40	0.56	0.44	0.00060355	1
P11047	LAMC1	16	125.8	113.9	115.9	74.2	86.1	84.1	1.70	1.32	1.38	1.47	0.00205194	1
P48449	LSS	10	95.4	99.3	78.2	104.6	100.7	121.8	0.91	0.99	0.64	0.85	0.12004624	1
Q6PKG0	LARP1	33	177.6	177.3	156.2	22.4	22.7	43.8	7.93	7.81	3.57	6.44	0.000149	1
Q71RC2	LARP4	5	123.2	137.1	143.9	76.8	62.9	56.1	1.60	2.18	2.57	2.12	0.0012846	1
Q92615	LARP4B	3	129.7	136.7	118.1	70.3	63.3	81.9	1.84	2.16	1.44	1.82	0.00183025	1
Q4G0J3	LARP7	6	139.3	159.9	149.1	60.7	40.1	50.9	2.29	3.99	2.93	3.07	0.0003	1
Q01650	SLC7A5	5	43.7	72.5	47.8	156.3	127.5	152.2	0.28	0.57	0.31	0.39	0.00204859	1
Q9UK59	DBR1	4	136.7	158.6	131.8	63.3	41.4	68.2	2.16	3.83	1.93	2.64	0.00189949	1
Q15334	LLGL1	7	142.2	149.1	110.1	57.8	50.9	89.9	2.46	2.93	1.22	2.20	0.01642873	1
Q86V48	LUZP1	5	187.3	128.9	179.6	12.7	71.1	20.4	14.75	1.81	8.80	8.45	0.00728912	1
P42704	LRPPRC	78	107	100.1	107.5	93	99.9	92.5	1.15	1.00	1.16	1.10	0.04490081	1
Q9UFC0	LRWD1	4	123	147.5	175.9	77	52.5	24.1	1.60	2.81	7.30	3.90	0.01069749	1
Q32MZ4	LRRFIP1	21	100	76.3	95.7	100	123.7	104.3	1.00	0.62	0.92	0.84	0.14443673	1
Q96AG4	LRRC59	19	70.8	100.5	68.6	129.2	99.5	131.4	0.55	1.01	0.52	0.69	0.05114861	1
Q9UIQ6	LNPEP	17	114.7	100.5	110.3	85.3	99.5	89.7	1.34	1.01	1.23	1.19	0.04573228	1
Q08722	CD47	2	101.4	95.5	98.6	98.6	104.5	101.4	1.03	0.91	0.97	0.97	0.28109961	1
P09960	LTA4H	20	104.6	95.1	98.7	95.4	104.9	101.3	1.10	0.91	0.97	0.99	0.79881054	1
P48059	LIMS1	5	79.8	98.7	73.7	120.2	101.3	126.3	0.66	0.97	0.58	0.74	0.04017104	1
Q14847	LASP1	21	57.2	31.7	55.9	142.8	168.3	144.1	0.40	0.19	0.39	0.33	0.00091056	1
Q9H6V9	LDAH	5	134.8	128.4	54.7	65.2	71.6	145.3	2.07	1.79	0.38	1.41	0.75912755	1
Q9P260	KIAA1468	5	111.7	96.6	91.6	88.3	103.4	108.4	1.27	0.93	0.85	1.01	0.99414811	1
P00338	LDHA	29	67.4	128	67.4	132.6	72	132.6	0.51	1.78	0.51	0.93	0.43430421	1
P07195	LDHB	21	101.8	128.5	101.9	98.2	71.5	98.1	1.04	1.80	1.04	1.29	0.1626841	1
P36776	LONP1	14	73.3	94.2	108	126.7	105.8	92	0.58	0.89	1.17	0.88	0.31605684	1
Q6P1M0	SLC27A4	6	93.9	100.1	119.6	106.1	99.9	80.4	0.89	1.00	1.49	1.12	0.45421993	1
P33121	ACSL1	9	96.4	85.4	50.9	103.6	114.6	149.1	0.93	0.75	0.34	0.67	0.08161205	1
O95573	ACSL3	26	103	96.3	106.4	97	103.7	93.6	1.06	0.93	1.14	1.04	0.41635528	1
O60488	ACSL4	21	103.2	95.7	102.2	96.8	104.3	97.8	1.07	0.92	1.04	1.01	0.83624112	1
P24666	ACP1	7	71	61.4	46.2	129	138.6	153.8	0.55	0.44	0.30	0.43	0.00137105	1
P01130	LDLR	7	116.1	131.1	164.5	83.9	68.9	35.5	1.38	1.90	4.63	2.64	0.02118387	1
P05455	SSB	28	114.7	104.3	118.7	85.3	95.7	81.3	1.34	1.09	1.46	1.30	0.0143636	1
Q7Z4W1	DCXR	7	47.8	84.6	42.1	152.2	115.4	157.9	0.31	0.73	0.27	0.44	0.01131671	1
Q5VWZ2	LYPLAL1	6	54.6	71.7	85.2	145.4	128.3	114.8	0.38	0.56	0.74	0.56	0.00922591	1
Q14108	SCARB2	10	67.3	62.8	74.3	132.7	137.2	125.7	0.51	0.46	0.59	0.52	0.00017578	1
P11279	LAMP1	6	91.6	81.7	88.4	108.4	118.3	111.6	0.85	0.69	0.79	0.78	0.00346249	1
P13473	LAMP2	1	80.6	65.3	79.8	119.4	134.7	120.2	0.68	0.48	0.66	0.61	0.00214069	1
Q96C86	DCPS	9	143.8	82	125.5	56.2	118	74.5	2.56	0.69	1.68	1.65	0.25746784	1
P14174	MIF	2	100.8	97.9	88.5	99.2	102.1	111.5	1.02	0.96	0.79	0.92	0.1794022	1
Q14728	MFSD10	1	128.7	120.1	130.6	71.3	79.9	69.4	1.81	1.50	1.88	1.73	0.00031694	1
P04156	PRNP	2	85.6	35.5	124.7	114.4	164.5	75.3	0.75	0.22	1.66	0.87	0.37832876	1

Q14764	MVP	45	87.9	78.4	86.7	112.1	121.6	113.3	0.78	0.64	0.77	0.73	0.00176261	1
P40926	MDH2	22	118	94.5	120.5	82	105.5	79.5	1.44	0.90	1.52	1.28	0.13352102	1
Q14165	MLEC	14	80.8	64	69.6	119.2	136	130.4	0.68	0.47	0.53	0.56	0.00122172	1
Q9UM22	EPDR1	3	35.6	70.8	41.8	164.4	129.2	158.2	0.22	0.55	0.26	0.34	0.00273662	1
Q9HD20	ATP13A1	11	105.9	140.5	148.7	94.1	59.5	51.3	1.13	2.36	2.90	2.13	0.02682531	1
O75352	MPDU1	1	88.5	82.3	100.5	111.5	117.7	99.5	0.79	0.70	1.01	0.83	0.0644865	1
Q13724	MOGS	6	109.3	120.3	104.1	90.7	79.7	95.9	1.21	1.51	1.09	1.27	0.02919532	1
P49006	MARCKSI	3	64.8	90.2	91.4	135.2	109.8	108.6	0.48	0.82	0.84	0.71	0.04354157	1
Q9NZD8	SPG21	6	101.9	137.9	115.3	98.1	62.1	84.7	1.04	2.22	1.36	1.54	0.06875331	1
Q14676	MDC1	17	197.2	179.6	170.2	2.8	20.4	29.8	70.43	8.80	5.71	28.31	0.00012415	1
Q15648	MED1	4	152.2	160.7	41	47.8	39.3	159	3.18	4.09	0.26	2.51	0.54595071	1
Q96PC5	MIA2	6	56.7	64.3	71.6	143.3	135.7	128.4	0.40	0.47	0.56	0.48	0.00029816	1
Q8N4V1	MMGT1	2	122.7	105.1	94.7	77.3	94.9	105.3	1.59	1.11	0.90	1.20	0.2640738	1
O00264	PGRMC1	13	84.9	88.4	91.8	115.1	111.6	108.2	0.74	0.79	0.85	0.79	0.0011724	1
O15173	PGRMC2	8	76.3	127.1	91	123.7	72.9	109	0.62	1.74	0.83	1.07	0.86964261	1
P55145	MANF	7	114.6	110	117.5	85.4	90	82.5	1.34	1.22	1.42	1.33	0.0008125	1
Q13505	MTX1	8	111.5	112.7	149.3	88.5	87.3	50.7	1.26	1.29	2.94	1.83	0.04915513	1
O75431	MTX2	5	76.5	42.1	73.7	123.5	157.9	126.3	0.62	0.27	0.58	0.49	0.01000773	1
Q9NZL9	MAT2B	12	84.2	42.2	93.8	115.8	157.8	106.2	0.73	0.27	0.88	0.63	0.07646681	1
P50579	METAP2	12	112.9	125.9	132.3	87.1	74.1	67.7	1.30	1.70	1.95	1.65	0.00419962	1
Q96RQ3	MCCC1	4	159.5	99.5	132	40.5	100.5	68	3.94	0.99	1.94	2.29	0.06866286	1
Q15800	MSMO1	2	99.7	87.2	83.6	100.3	112.8	116.4	0.99	0.77	0.72	0.83	0.04637356	1
Q96GX9	APIP	9	112.2	68.8	114.7	87.8	131.2	85.3	1.28	0.52	1.34	1.05	0.89836488	1
P22670	RFX1	5	146.9	162.3	166.7	53.1	37.7	33.3	2.77	4.31	5.01	4.03	0.00015917	1
Q8N3F8	MICALL1	11	158.7	146.6	169.9	41.3	53.4	30.1	3.84	2.75	5.64	4.08	0.00025291	1
Q9NX63	CHCHD3	14	72	57.4	84.6	128	142.6	115.4	0.56	0.40	0.73	0.57	0.00670499	1
Q9BUR5	APOO	4	124.5	109.9	124	75.5	90.1	76	1.65	1.22	1.63	1.50	0.00452743	1
Q16891	IMMT	14	96.3	92.1	98.3	103.7	107.9	101.7	0.93	0.85	0.97	0.92	0.02649292	1
O14880	MGST3	5	55.6	49.8	75	144.4	150.2	125	0.39	0.33	0.60	0.44	0.00177934	1
Q15691	MAPRE1	18	142.4	90.7	141.5	57.6	109.3	58.5	2.47	0.83	2.42	1.91	0.10866125	1
Q15555	MAPRE2	4	75.7	89.5	91.7	124.3	110.5	108.3	0.61	0.81	0.85	0.76	0.0153773	1
A6NCE7	MAP1LC3	2	135.4	118.9	114.9	64.6	81.1	85.1	2.10	1.47	1.35	1.64	0.00651814	1
Q8N183	NDUFAF2	7	126.3	115.6	97.3	73.7	84.4	102.7	1.71	1.37	0.95	1.34	0.09447858	1
Q8TCT9	HM13	7	128.4	122.9	133.4	71.6	77.1	66.6	1.79	1.59	2.00	1.80	0.00019212	1
Q02978	SLC25A11	14	60.5	95	65	139.5	105	135	0.43	0.90	0.48	0.61	0.0257937	1
Q7Z434	MAVS	12	60.2	63.5	72.9	139.8	136.5	127.1	0.43	0.47	0.57	0.49	0.00021393	1
O43772	SLC25A20	3	117.5	73.4	104.6	82.5	126.6	95.4	1.42	0.58	1.10	1.03	0.87912567	1
Q9UBX3	SLC25A10	9	65.5	115	53	134.5	85	147	0.49	1.35	0.36	0.73	0.17306752	1
Q9Y3D6	FIS1	2	122.1	109.9	122.5	77.9	90.1	77.5	1.57	1.22	1.58	1.46	0.00341476	1
Q9GZY8	MFF	7	104.9	32.8	90.1	95.1	167.2	109.9	1.10	0.20	0.82	0.71	0.19652277	1
Q9BQP7	MGME1	7	71.3	105.6	93.4	128.7	94.4	106.6	0.55	1.12	0.88	0.85	0.23555579	1

Q9H936	SLC25A22	9	78.5	108.3	64.4	121.5	91.7	135.6	0.65	1.18	0.47	0.77	0.15007505		1
Q9Y5L4	TIMM13	4	141.1	129.5	104.9	58.9	70.5	95.1	2.40	1.84	1.10	1.78	0.02897299		1
Q9Y3D7	PAM16	3	124.8	112.3	79	75.2	87.7	121	1.66	1.28	0.65	1.20	0.60829623		1
Q99595	TIMM17A	2	110.3	98	65.4	89.7	102	134.6	1.23	0.96	0.49	0.89	0.40708402		1
O60830	TIMM17B	4	95	84.7	155	105	115.3	45	0.90	0.73	3.44	1.69	0.49697479		1
Q15785	TOMM34	14	154.6	128.4	154.8	45.4	71.6	45.2	3.41	1.79	3.42	2.87	0.00177002		1
O94826	TOMM70	24	114.8	102.2	113.7	85.2	97.8	86.3	1.35	1.04	1.32	1.24	0.02292479		1
Q9Y619	SLC25A15	3	41	79.5	58.3	159	120.5	141.7	0.26	0.66	0.41	0.44	0.00682914		1
Q8IXI1	RHOT2	3	161.1	93.7	75.4	38.9	106.3	124.6	4.14	0.88	0.61	1.88	0.61387666		1
Q7L0Y3	TRMT10C	7	125.7	153.8	127.8	74.3	46.2	72.2	1.69	3.33	1.77	2.26	0.00500131		1
Q9Y2U5	MAP3K2	5	99.5	68.2	106.9	100.5	131.8	93.1	0.99	0.52	1.15	0.89	0.36982835		1
O95819	MAP4K4	4	125	152.2	164.4	75	47.8	35.6	1.67	3.18	4.62	3.16	0.00458745		1
O43684	BUB3	10	74.7	113.8	125.6	125.3	86.2	74.4	0.60	1.32	1.69	1.20	0.68793038		1
Q9Y6D9	MAD1L1	8	139.3	144.5	128.2	60.7	55.5	71.8	2.29	2.60	1.79	2.23	0.00039048		1
Q6NZ67	MZT2B	3	125.2	124.9	82.1	74.8	75.1	117.9	1.67	1.66	0.70	1.34	0.34881513		1
Q9BYG3	NIFK	4	100.1	80	110.2	99.9	120	89.8	1.00	0.67	1.23	0.97	0.63359349		1
Q96T76	MMS19	10	141.7	124.9	136.6	58.3	75.1	63.4	2.43	1.66	2.15	2.08	0.00061185		1
P53985	SLC16A1	5	134.1	133.7	121.2	65.9	66.3	78.8	2.03	2.02	1.54	1.86	0.00058271		1
O15427	SLC16A3	5	125.1	105.2	124.6	74.9	94.8	75.4	1.67	1.11	1.65	1.48	0.01681515		1
Q99685	MGLL	11	66.7	96.5	48	133.3	103.5	152	0.50	0.93	0.32	0.58	0.04138455		1
Q9H3R2	MUC13	2	111.2	13	30	88.8	187	170	1.25	0.07	0.18	0.50	0.08588514		1
P33527	ABCC1	37	17.1	52.2	15.4	182.9	147.8	184.6	0.09	0.35	0.08	0.18	0.00106852		1
Q9UI30	TRMT112	3	123.7	112.2	114.4	76.3	87.8	85.6	1.62	1.28	1.34	1.41	0.00254228		1
P22234	PAICS	21	97.3	113.5	107.4	102.7	86.5	92.6	0.95	1.31	1.16	1.14	0.14350345		1
O75970	MPDZ	7	118.3	107.6	90.7	81.7	92.4	109.3	1.45	1.16	0.83	1.15	0.38520682		1
Q96EY5	MVB12A	5	153.2	56.4	164.1	46.8	143.6	35.9	3.27	0.39	4.57	2.75	0.36749917		1
Q9BQG0	MYBBP1A	13	99.6	96.4	102.5	100.4	103.6	97.5	0.99	0.93	1.05	0.99	0.70864978		1
Q9NUJ1	ABHD10	9	109.7	82.4	109.5	90.3	117.6	90.5	1.21	0.70	1.21	1.04	0.93769928		1
Q9P2K5	MYEF2	8	183.7	150.3	176.6	16.3	49.7	23.4	11.27	3.02	7.55	7.28	0.00061434		1
Q96S97	MYADM	1	78.8	54.1	72.1	121.2	145.9	127.9	0.65	0.37	0.56	0.53	0.00371519		1
Q9NZM1	MYOF	133	105.9	98.7	105.6	94.1	101.3	94.4	1.13	0.97	1.12	1.07	0.11035157		1
P60660	MYL6	7	102.8	101.1	100.2	97.2	98.9	99.8	1.06	1.02	1.00	1.03	0.06429379		1
O14950	MYL12B	6	111.1	113.4	126.3	88.9	86.6	73.7	1.25	1.31	1.71	1.42	0.00716701		1
P58546	MTPN	3	102.1	105.8	131.6	97.9	94.2	68.4	1.04	1.12	1.92	1.36	0.11520907		1
P20933	AGA	3	90.6	77.5	67.8	109.4	122.5	132.2	0.83	0.63	0.51	0.66	0.01022914		1
O94760	DDAH1	7	103.4	138.9	120.5	96.6	61.1	79.5	1.07	2.27	1.52	1.62	0.04464614		1
O95865	DDAH2	4	149.1	132.3	161.8	50.9	67.7	38.2	2.93	1.95	4.24	3.04	0.00138771		1
Q9UJ70	NAGK	15	90.3	89.1	85.8	109.7	110.9	114.2	0.82	0.80	0.75	0.79	0.00025965		1
Q86SF2	GALNT7	4	32.7	64	34.8	167.3	136	165.2	0.20	0.47	0.21	0.29	0.0014135		1
P15586	GNS	10	132.8	118.7	141.7	67.2	81.3	58.3	1.98	1.46	2.43	1.96	0.00279069		1
O95671	ASMTL	8	105	150.5	142.5	95	49.5	57.5	1.11	3.04	2.48	2.21	0.03010226		1

Q13423	NNT	6	87.6	82.4	84.8	112.4	117.6	115.2	0.78	0.70	0.74	0.74	0.00014359	1
P15559	NQO1	21	31.8	64.6	28.1	168.2	135.4	171.9	0.19	0.48	0.16	0.28	0.00204324	1
P23368	ME2	17	130.3	127.1	117.5	69.7	72.9	82.5	1.87	1.74	1.42	1.68	0.00078173	1
Q9NXA8	SIRT5	4	133.4	106.9	110.8	66.6	93.1	89.2	2.00	1.15	1.24	1.46	0.04340936	1
Q9BU61	NDUFAF3	2	151.4	163.6	137.7	48.6	36.4	62.3	3.12	4.49	2.21	3.27	0.00065221	1
Q9P032	NDUFAF4	4	163.1	137.1	136.5	36.9	62.9	63.5	4.42	2.18	2.15	2.92	0.00182553	1
Q9UI09	NDUFA12	3	9.7	77.4	99.4	190.3	122.6	100.6	0.05	0.63	0.99	0.56	0.11848991	1
Q16718	NDUFA5	3	121.9	112.4	129.8	78.1	87.6	70.2	1.56	1.28	1.85	1.56	0.00386518	1
Q16795	NDUFA9	18	98.8	80.6	107.7	101.2	119.4	92.3	0.98	0.68	1.17	0.94	0.48824363	1
O43676	NDUFB3	3	98.4	96.3	104.5	101.6	103.7	95.5	0.97	0.93	1.09	1.00	0.88554698	1
O95169	NDUFB8	4	103.7	88	117.7	96.3	112	82.3	1.08	0.79	1.43	1.10	0.6327105	1
O75489	NDUFS3	14	116.4	98.8	113.2	83.6	101.2	86.8	1.39	0.98	1.30	1.22	0.06869547	1
P00387	CYB5R3	17	115.5	91.5	118.5	84.5	108.5	81.5	1.37	0.84	1.45	1.22	0.2321905	1
P28331	NDUFS1	17	108.5	89	103.8	91.5	111	96.2	1.19	0.80	1.08	1.02	0.92194992	1
P41227	NAA10	12	114.9	105.2	116.2	85.1	94.8	83.8	1.35	1.11	1.39	1.28	0.00786773	1
Q9BXJ9	NAA15	15	133.1	138.3	149.1	66.9	61.7	50.9	1.99	2.24	2.93	2.39	0.00027155	1
Q14CX7	NAA25	2	166.8	117.4	123	33.2	82.6	77	5.02	1.42	1.60	2.68	0.03179956	1
Q9GZZ1	NAA50	12	125.1	114.6	120.8	74.9	85.4	79.2	1.67	1.34	1.53	1.51	0.00072614	1
O43847	NRDC	24	129.6	120	136.8	70.4	80	63.2	1.84	1.50	2.16	1.84	0.00111441	1
E9PAV3	NACA	5	117.2	117.3	110.6	82.8	82.7	89.4	1.42	1.42	1.24	1.36	0.00066066	1
Q9Y2A7	NCKAP1	9	171.3	128.6	125.5	28.7	71.4	74.5	5.97	1.80	1.68	3.15	0.01612497	1
Q92692	NECTIN2	8	132.8	134.7	150.7	67.2	65.3	49.3	1.98	2.06	3.06	2.37	0.00060392	1
Q15843	NEDD8	4	103.7	99.3	111.3	96.3	100.7	88.7	1.08	0.99	1.25	1.11	0.12678575	1
Q13564	NAE1	7	143.3	118.7	114.8	56.7	81.3	85.2	2.53	1.46	1.35	1.78	0.0153724	1
P61081	UBE2M	9	89.8	58.2	84.1	110.2	141.8	115.9	0.81	0.41	0.73	0.65	0.03016355	1
O00401	WASL	3	117.9	103.2	169.8	82.1	96.8	30.2	1.44	1.07	5.62	2.71	0.10120039	1
Q9BYT8	NLN	6	92.4	80.3	85.1	107.6	119.7	114.9	0.86	0.67	0.74	0.76	0.00481797	1
Q8IY17	PNPLA6	4	147.9	125.3	115.3	52.1	74.7	84.7	2.84	1.68	1.36	1.96	0.01238175	1
Q14697	GANAB	48	123.9	115.7	124.7	76.1	84.3	75.3	1.63	1.37	1.66	1.55	0.00045839	1
Q8NBF2	NHLRC2	8	104.7	93.5	102.4	95.3	106.5	97.6	1.10	0.88	1.05	1.01	0.93797383	1
O60934	NBN	3	139.3	137.2	151.9	60.7	62.8	48.1	2.29	2.18	3.16	2.55	0.00019106	1
Q92542	NCSTN	7	89.3	99.2	77.7	110.7	100.8	122.3	0.81	0.98	0.64	0.81	0.06233735	1
P40261	NNMT	13	21.7	44.4	26.4	178.3	155.6	173.6	0.12	0.29	0.15	0.19	0.00014521	1
O15118	NPC1	11	108.9	104.4	107.6	91.1	95.6	92.4	1.20	1.09	1.16	1.15	0.00180755	1
Q9Y314	NOSIP	7	98.8	82.8	121	101.2	117.2	79	0.98	0.71	1.53	1.07	0.91721506	1
Q86X76	NIT1	8	109.8	73.9	78.7	90.2	126.1	121.3	1.22	0.59	0.65	0.82	0.19032819	1
Q15233	NONO	21	173.8	147.6	159.6	26.2	52.4	40.4	6.63	2.82	3.95	4.47	0.00035341	1
Q96MG7	NSMCE3	5	122.2	83.7	117.7	77.8	116.3	82.3	1.57	0.72	1.43	1.24	0.41176141	1
Q9UNZ2	NSFL1C	16	101.6	99.3	102.3	98.4	100.7	97.7	1.03	0.99	1.05	1.02	0.17129864	1
Q9BV86	NTMT1	6	117.2	121.8	125.2	82.8	78.2	74.8	1.42	1.56	1.67	1.55	0.00019867	1
P23497	SP100	2	102.8	97.3	78.1	97.2	102.7	121.9	1.06	0.95	0.64	0.88	0.24180567	1

Q53F19	NCBP3	1	125.7	116	123.5	74.3	84	76.5	1.69	1.38	1.61	1.56	0.0004707		1
O60524	NEMF	4	114	88.7	123.4	86	111.3	76.6	1.33	0.80	1.61	1.24	0.30075416		1
Q7Z417	NUFIP2	3	125.1	125.6	107.9	74.9	74.4	92.1	1.67	1.69	1.17	1.51	0.00898608		1
Q9Y266	NUDC	25	144.4	160	161.8	55.6	40	38.2	2.60	4.00	4.24	3.61	0.00014354		1
Q14980	NUMA1	72	122	115.7	123.2	78	84.3	76.8	1.56	1.37	1.60	1.51	0.00024759		1
O75694	NUP155	16	178.9	154.8	162.7	21.1	45.2	37.3	8.48	3.42	4.36	5.42	0.00019888		1
Q92621	NUP205	8	127	134.5	167.7	73	65.5	32.3	1.74	2.05	5.19	3.00	0.00821797		1
P35658	NUP214	17	139.8	109.4	138.3	60.2	90.6	61.7	2.32	1.21	2.24	1.92	0.01403709		1
Q99567	NUP88	6	114.5	135.4	151.9	85.5	64.6	48.1	1.34	2.10	3.16	2.20	0.01138081		1
Q8N1F7	NUP93	10	125.9	144.4	86.9	74.1	55.6	113.1	1.70	2.60	0.77	1.69	0.18679419		1
Q8TEM1	NUP210	9	102.7	100.7	115.6	97.3	99.3	84.4	1.06	1.01	1.37	1.15	0.12752637		1
Q8TAT6	NPLOC4	23	114.1	108.9	117.5	85.9	91.1	82.5	1.33	1.20	1.42	1.32	0.00158078		1
P61970	NUTF2	2	98.5	89	92.4	101.5	111	107.6	0.97	0.80	0.86	0.88	0.02704047		1
Q02818	NUCB1	12	84	66.7	84.1	116	133.3	115.9	0.72	0.50	0.73	0.65	0.00602811		1
Q9BZE4	GTPBP4	5	126.9	129.8	121.3	73.1	70.2	78.7	1.74	1.85	1.54	1.71	0.00012332		1
Q9Y2X3	NOP58	5	142.2	127.2	141.4	57.8	72.8	58.6	2.46	1.75	2.41	2.21	0.00042906		1
P19338	NCL	43	112.4	107.7	113.4	87.6	92.3	86.6	1.28	1.17	1.31	1.25	0.00084853		1
P06748	NPM1	16	114.7	125.9	116.9	85.3	74.1	83.1	1.34	1.70	1.41	1.48	0.00138096		1
Q8NFH4	NUP37	6	151.1	153.5	92.6	48.9	46.5	107.4	3.09	3.30	0.86	2.42	0.08283469		1
Q8NFH5	NUP35	10	124.1	51	119.2	75.9	149	80.8	1.64	0.34	1.48	1.15	0.91481057		1
Q96EE3	SEH1L	9	131	162.5	130.7	69	37.5	69.3	1.90	4.33	1.89	2.71	0.00515871		1
Q13232	NME3	6	77.5	63.2	80.1	122.5	136.8	119.9	0.63	0.46	0.67	0.59	0.00207173		1
P15531	NME1	5	100.9	99.6	90.7	99.1	100.4	109.3	1.02	0.99	0.83	0.95	0.26526388		1
P22392	NME2	9	107	101	115.1	93	99	84.9	1.15	1.02	1.36	1.18	0.05606849		1
P55209	NAP1L1	15	108.8	145.6	138.2	91.2	54.4	61.8	1.19	2.68	2.24	2.04	0.0177771		1
Q96RS6	NUDCD1	9	154.8	142.9	169	45.2	57.1	31	3.42	2.50	5.45	3.79	0.00047979		1
Q8WVJ2	NUDCD2	7	146.5	148.4	133.5	53.5	51.6	66.5	2.74	2.88	2.01	2.54	0.00020654		1
Q9Y530	OARD1	5	138.2	121	133.6	61.8	79	66.4	2.24	1.53	2.01	1.93	0.00104628		1
Q9Y3B8	REXO2	10	128.3	67.5	121.7	71.7	132.5	78.3	1.79	0.51	1.55	1.28	0.69048646		1
Q9NRP0	OSTC	1	132	123.1	132.9	68	76.9	67.1	1.94	1.60	1.98	1.84	0.0001867		1
Q9NQR4	NIT2	21	78.2	86	74.1	121.8	114	125.9	0.64	0.75	0.59	0.66	0.001133		1
Q9NZT2	OGFR	8	125.1	95.8	101.4	74.9	104.2	98.6	1.67	0.92	1.03	1.21	0.30672942		1
Q96CV9	OPTN	5	115	98.8	121.9	85	101.2	78.1	1.35	0.98	1.56	1.30	0.06982761		1
Q53FV1	ORMDL2	1	92.7	81.3	92.2	107.3	118.7	107.8	0.86	0.68	0.86	0.80	0.01280893		1
Q01804	OTUD4	3	128.4	161.8	143.8	71.6	38.2	56.2	1.79	4.24	2.56	2.86	0.00281705		1
Q8N6M0	OTUD6B	8	156.2	130.8	159.7	43.8	69.2	40.3	3.57	1.89	3.96	3.14	0.00161289		1
Q9BUP3	HTATIP2	11	22	78.7	9.4	178	121.3	190.6	0.12	0.65	0.05	0.27	0.01369245		1
P36551	CPOX	16	102.1	92.2	80.4	97.9	107.8	119.6	1.04	0.86	0.67	0.86	0.13001725		1
P22059	OSBP	16	115	113.1	121.4	85	86.9	78.6	1.35	1.30	1.54	1.40	0.00074559		1
Q9BXB4	OSBPL11	6	133.7	124.4	158.8	66.3	75.6	41.2	2.02	1.65	3.85	2.51	0.00583093		1
Q9BZF1	OSBPL8	12	164.5	146.5	168.2	35.5	53.5	31.8	4.63	2.74	5.29	4.22	0.0002281		1

Q96SU4	OSBPL9	6	136.1	121.4	116.4	63.9	78.6	83.6	2.13	1.54	1.39	1.69	0.00414855		1
Q96ST3	SIN3A	8	130.3	144.5	153.3	69.7	55.5	46.7	1.87	2.60	3.28	2.59	0.00083929		1
Q8WX93	PALLD	16	40.4	65.8	31.5	159.6	134.2	168.5	0.25	0.49	0.19	0.31	0.0017384		1
P50897	PPT1	7	161.6	126.2	139.3	38.4	73.8	60.7	4.21	1.71	2.29	2.74	0.00439968		1
Q9BRP8	PYM1	5	102.1	43.4	39.3	97.9	156.6	160.7	1.04	0.28	0.24	0.52	0.05538962		1
O75475	PSIP1	6	168.3	152.3	142.6	31.7	47.7	57.4	5.31	3.19	2.48	3.66	0.00050736		1
Q96HC4	PDLIM5	16	92.8	87.5	97.6	107.2	112.5	102.4	0.87	0.78	0.95	0.87	0.02333971		1
Q5EBL8	PDZD11	2	111.5	149.1	156.7	88.5	50.9	43.3	1.26	2.93	3.62	2.60	0.01671623		1
Q9UBV8	PEF1	7	124.9	90.3	124.8	75.1	109.7	75.2	1.66	0.82	1.66	1.38	0.17691155		1
Q96EY7	PTCD3	11	126.9	98.8	119.7	73.1	101.2	80.3	1.74	0.98	1.49	1.40	0.06400375		1
P23284	PPIB	19	113.8	102.7	114.8	86.2	97.3	85.2	1.32	1.06	1.35	1.24	0.019016		1
P30405	PPIF	5	97.5	98.8	98.4	102.5	101.2	101.6	0.95	0.98	0.97	0.97	0.0028912		1
P62942	FKBP1A	2	97.6	80.6	88.7	102.4	119.4	111.3	0.95	0.68	0.80	0.81	0.03358783		1
P26885	FKBP2	5	91.1	102.3	104.4	108.9	97.7	95.6	0.84	1.05	1.09	0.99	0.81401643		1
Q00688	FKBP3	16	65.5	81.9	69.5	134.5	118.1	130.5	0.49	0.69	0.53	0.57	0.00136572		1
Q02790	FKBP4	30	127.9	103.3	108.9	72.1	96.7	91.1	1.77	1.07	1.20	1.35	0.06402868		1
Q13526	PIN1	6	127.4	116.8	99	72.6	83.2	101	1.75	1.40	0.98	1.38	0.06985643		1
Q9Y237	PIN4	6	154.7	149.4	134.8	45.3	50.6	65.2	3.42	2.95	2.07	2.81	0.00038754		1
Q9Y3C6	PPIL1	5	133.8	138.7	151.5	66.2	61.3	48.5	2.02	2.26	3.12	2.47	0.0003776		1
Q9H2H8	PPIL3	3	114.9	148.1	133.1	85.1	51.9	66.9	1.35	2.85	1.99	2.06	0.00917481		1
Q8WUA2	PPIL4	5	146	170.3	127.2	54	29.7	72.8	2.70	5.73	1.75	3.39	0.00560845		1
Q96BP3	PPWD1	3	100.5	143	135.4	99.5	57	64.6	1.01	2.51	2.10	1.87	0.04675262		1
Q9Y3E5	PTRH2	5	91.5	85.8	89.4	108.5	114.2	110.6	0.84	0.75	0.81	0.80	0.00070452		1
Q14197	MRPL58	7	112.1	96.9	192.1	87.9	103.1	7.9	1.28	0.94	24.32	8.84	0.18181888		1
Q13610	PWP1	3	142.9	117.4	104.6	57.1	82.6	95.4	2.50	1.42	1.10	1.67	0.05310861		1
Q06830	PRDX1	21	89.6	85.7	88.8	110.4	114.3	111.2	0.81	0.75	0.80	0.79	0.00014165		1
Q13162	PRDX4	14	119.7	92.7	129.1	80.3	107.3	70.9	1.49	0.86	1.82	1.39	0.1473953		1
P30044	PRDX5	10	79	73.1	83.2	121	126.9	116.8	0.65	0.58	0.71	0.65	0.00048062		1
P30041	PRDX6	20	103	88.5	103.5	97	111.5	96.5	1.06	0.79	1.07	0.98	0.65682523		1
Q9NUI1	DECR2	8	54.7	69.8	49.1	145.3	130.2	150.9	0.38	0.54	0.33	0.41	0.0006479		1
Q15067	ACOX1	10	59.1	44.5	128.7	140.9	155.5	71.3	0.42	0.29	1.81	0.84	0.28658717		1
O96011	PEX11B	7	8.1	60.2	48.1	191.9	139.8	151.9	0.04	0.43	0.32	0.26	0.00533554		1
P51659	HSD17B4	28	71.8	80.1	79.9	128.2	119.9	120.1	0.56	0.67	0.67	0.63	0.00029919		1
Q6Y7W6	GIGYF2	22	110.9	99	113.3	89.1	101	86.7	1.24	0.98	1.31	1.18	0.0686794		1
O95571	ETHE1	11	122.4	67.1	140.1	77.6	132.9	59.9	1.58	0.50	2.34	1.47	0.56012637		1
O00541	PES1	6	108.2	98.8	122.6	91.8	101.2	77.4	1.18	0.98	1.58	1.25	0.11399381		1
Q8WW12	PCNP	10	123.4	98.2	131.2	76.6	101.8	68.8	1.61	0.96	1.91	1.49	0.06679966		1
Q7RTV0	PHF5A	3	124	111	98.4	76	89	101.6	1.63	1.25	0.97	1.28	0.10015618		1
Q9NSD9	FARSB	19	97.8	91.6	96.3	102.2	108.4	103.7	0.96	0.85	0.93	0.91	0.02256524		1
Q9H2J4	PDCL3	11	151.3	120.7	150.9	48.7	79.3	49.1	3.11	1.52	3.07	2.57	0.00463147		1
O75167	PHACTR2	1	133.1	150.3	137.9	66.9	49.7	62.1	1.99	3.02	2.22	2.41	0.00036707		1

Q8IZ21	PHACTR4	7	172.4	144.4	175.3	27.6	55.6	24.7	6.25	2.60	7.10	5.31	0.00077822	1
Q96BW9	TAMM41	3	131	146.1	117.8	69	53.9	82.2	1.90	2.71	1.43	2.01	0.00542748	1
P30086	PEBP1	14	95	84.9	94.3	105	115.1	105.7	0.90	0.74	0.89	0.84	0.0202085	1
Q9NTJ5	SACM1L	5	136.8	84.9	122.2	63.2	115.1	77.8	2.16	0.74	1.57	1.49	0.25152768	1
O15357	INPPL1	15	114.6	116.4	99	85.4	83.6	101	1.34	1.39	0.98	1.24	0.06264482	1
Q00169	PITPNA	13	80.4	90.5	60.1	119.6	109.5	139.9	0.67	0.83	0.43	0.64	0.02198762	1
P48739	PITPNB	16	137.8	116	141.8	62.2	84	58.2	2.22	1.38	2.44	2.01	0.00492368	1
Q13492	PICALM	4	146.1	125.4	124	53.9	74.6	76	2.71	1.68	1.63	2.01	0.00324262	1
Q6VY07	PACS1	6	111.6	111.5	122.7	88.4	88.5	77.3	1.26	1.26	1.59	1.37	0.00437022	1
P36871	PGM1	20	96.1	89.5	93.8	103.9	110.5	106.2	0.92	0.81	0.88	0.87	0.00738193	1
P00558	PGK1	33	105.5	98.2	105.3	94.5	101.8	94.7	1.12	0.96	1.11	1.06	0.15192513	1
P18669	PGAM1	23	105.3	98.4	114.8	94.7	101.6	85.2	1.11	0.97	1.35	1.14	0.14050375	1
Q8IV08	PLD3	8	135.5	151.2	144.5	64.5	48.8	55.5	2.10	3.10	2.60	2.60	0.00016934	1
O15162	PLSCR1	4	45.1	28	63.8	154.9	172	136.2	0.29	0.16	0.47	0.31	0.0017454	1
O15305	PMM2	8	56.4	83	46.6	143.6	117	153.4	0.39	0.71	0.30	0.47	0.00780632	1
O60256	PRPSAP2	9	118.8	117.5	105	81.2	82.5	95	1.46	1.42	1.11	1.33	0.01146271	1
O15067	PFAS	46	96	88.4	97.1	104	111.6	102.9	0.92	0.79	0.94	0.89	0.03326478	1
Q9H4Z3	PCIF1	3	119.3	109.5	138.8	80.7	90.5	61.2	1.48	1.21	2.27	1.65	0.02082454	1
P78330	PSPH	6	87.1	72.8	82.4	112.9	127.2	117.6	0.77	0.57	0.70	0.68	0.00295051	1
Q6NYC8	PPP1R18	9	185.5	176.6	157.6	14.5	23.4	42.4	12.79	7.55	3.72	8.02	0.00022929	1
P20020	ATP2B1	15	77.3	65.3	74	122.7	134.7	126	0.63	0.48	0.59	0.57	0.00039029	1
P23634	ATP2B4	6	143.3	117.2	132.8	56.7	82.8	67.2	2.53	1.42	1.98	1.97	0.00439277	1
P13797	PLS3	21	93.9	89.5	95.7	106.1	110.5	104.3	0.89	0.81	0.92	0.87	0.0058849	1
P68402	PAFAH1B	7	126.3	113.1	128.7	73.7	86.9	71.3	1.71	1.30	1.81	1.61	0.00270094	1
Q15149	PLEC	139	151.4	132.7	147.3	48.6	67.3	52.7	3.12	1.97	2.80	2.63	0.00040002	1
O15031	PLXNB2	12	57.6	50.4	78.7	142.4	149.6	121.3	0.40	0.34	0.65	0.46	0.00326409	1
P15151	PVR	5	115.6	84.4	123.9	84.4	115.6	76.1	1.37	0.73	1.63	1.24	0.40182064	1
Q9UKK3	PARP4	29	89.1	88.2	93.7	110.9	111.8	106.3	0.80	0.79	0.88	0.82	0.00130754	1
P51003	PAPOLA	7	122.8	146.1	146.9	77.2	53.9	53.1	1.59	2.71	2.77	2.36	0.0023047	1
O95453	PARN	4	63.4	88	102.3	136.6	112	97.7	0.46	0.79	1.05	0.77	0.12707841	1
Q9NX46	ADPRHL2	7	37.8	118.7	154.7	162.2	81.3	45.3	0.23	1.46	3.42	1.70	0.88599548	1
Q15365	PCBP1	10	95.8	86.6	100.5	104.2	113.4	99.5	0.92	0.76	1.01	0.90	0.11951962	1
Q15366	PCBP2	9	93.8	117.8	106.3	106.2	82.2	93.7	0.88	1.43	1.13	1.15	0.29029482	1
Q9H074	PAIP1	7	108.3	101.5	106.6	91.7	98.5	93.4	1.18	1.03	1.14	1.12	0.0193707	1
Q9Y2S7	POLDIP2	17	65.5	95.5	96.6	134.5	104.5	103.4	0.49	0.91	0.93	0.78	0.12130161	1
Q6NZI2	PTRF	11	90.3	105.7	112.6	109.7	94.3	87.4	0.82	1.12	1.29	1.08	0.57179035	1
Q10471	GALNT2	7	99	81.6	57.7	101	118.4	142.3	0.98	0.69	0.41	0.69	0.07201244	1
Q8N4A0	GALNT4	4	57.3	93.9	44.2	142.7	106.1	155.8	0.40	0.89	0.28	0.52	0.02950181	1
Q8TCS8	PNPT1	12	118.7	102.5	107.8	81.3	97.5	92.2	1.46	1.05	1.17	1.23	0.04561899	1
P14859	POU2F1	2	122.4	116.4	48.9	77.6	83.6	151.1	1.58	1.39	0.32	1.10	0.81774083	1
O60831	PRAF2	3	126.6	133.6	137.9	73.4	66.4	62.1	1.72	2.01	2.22	1.99	0.00014917	1

O75915	ARL6IP5	5	100.8	81.5	99.7	99.2	118.5	100.3	1.02	0.69	0.99	0.90	0.24662705		1
O60925	PFDN1	3	140.8	90	163.9	59.2	110	36.1	2.38	0.82	4.54	2.58	0.11028081		1
Q9UHV9	PFDN2	5	137.1	127.7	158.6	62.9	72.3	41.4	2.18	1.77	3.83	2.59	0.00313122		1
Q99471	PFDN5	7	137.1	116.5	114.5	62.9	83.5	85.5	2.18	1.40	1.34	1.64	0.01129517		1
O15212	PFDN6	5	151.1	136.1	138.8	48.9	63.9	61.2	3.09	2.13	2.27	2.50	0.00021036		1
O94906	PRPF6	13	151.6	165	176.2	48.4	35	23.8	3.13	4.71	7.40	5.08	0.00021593		1
Q9HCS7	XAB2	8	142.5	142.6	96.2	57.5	57.4	103.8	2.48	2.48	0.93	1.96	0.06816798		1
Q9UI14	RABAC1	4	122.6	108.7	103.3	77.4	91.3	96.7	1.58	1.19	1.07	1.28	0.04699802		1
Q2NL82	TSR1	10	134.4	126.9	142.2	65.6	73.1	57.8	2.05	1.74	2.46	2.08	0.00038191		1
Q969E8	TSR2	2	154	120.5	124.4	46	79.5	75.6	3.35	1.52	1.65	2.17	0.01161904		1
P49768	PSEN1	5	118.9	109.5	141.1	81.1	90.5	58.9	1.47	1.21	2.40	1.69	0.02495961		1
Q5JRX3	PITRM1	17	99.6	85.2	100.4	100.4	114.8	99.6	0.99	0.74	1.01	0.91	0.23062107		1
Q96IZ0	PAWR	5	60	100.6	94.9	140	99.4	105.1	0.43	1.01	0.90	0.78	0.1736717		1
Q92841	DDX17	19	139.5	136.1	150.1	60.5	63.9	49.9	2.31	2.13	3.01	2.48	0.00014868		1
O95786	DDX58	15	171.2	127.8	135.2	28.8	72.2	64.8	5.94	1.77	2.09	3.27	0.00917534		1
Q9UNQ2	DIMT1	6	104	157.7	102.4	96	42.3	97.6	1.08	3.73	1.05	1.95	0.17168946		1
P28370	SMARCA1	2	140.2	120.4	135.1	59.8	79.6	64.9	2.34	1.51	2.08	1.98	0.00160778		1
Q8TED1	GPX8	5	115.6	129.1	101.9	84.4	70.9	98.1	1.37	1.82	1.04	1.41	0.04892732		1
Q5W0Z9	ZDHHC20	3	84.2	87.6	97.6	115.8	112.4	102.4	0.73	0.78	0.95	0.82	0.02302606		1
Q9NPF4	OSGEP	6	55.3	98.5	122.1	144.7	101.5	77.9	0.38	0.97	1.57	0.97	0.59248181		1
Q9NWX6	THG1L	6	65.5	106	124.2	134.5	94	75.8	0.49	1.13	1.64	1.08	0.91261428		1
Q8NBJ5	COLGALT	12	116.1	113	120.9	83.9	87	79.1	1.38	1.30	1.53	1.40	0.00050946		1
Q02809	PLOD1	15	136.8	143.2	122.5	63.2	56.8	77.5	2.16	2.52	1.58	2.09	0.00139097		1
O60568	PLOD3	15	135.5	143.2	150.6	64.5	56.8	49.4	2.10	2.52	3.05	2.56	0.00015177		1
P07737	PFN1	15	87.1	80	86	112.9	120	114	0.77	0.67	0.75	0.73	0.00055751		1
P35080	PFN2	6	125.9	116	134.1	74.1	84	65.9	1.70	1.38	2.03	1.70	0.00238152		1
Q8WUM4	PDCD6IP	40	101	100.2	108.7	99	99.8	91.3	1.02	1.00	1.19	1.07	0.16013816		1
Q9BUL8	PDCD10	5	100.5	95.5	107.6	99.5	104.5	92.4	1.01	0.91	1.16	1.03	0.65406248		1
O75340	PDCD6	7	110.5	98.6	177.3	89.5	101.4	22.7	1.23	0.97	7.81	3.34	0.17165463		1
P35232	PHB	19	133.8	148.5	128.5	66.2	51.5	71.5	2.02	2.88	1.80	2.23	0.00094805		1
Q99623	PHB2	18	123.7	160.4	126.6	76.3	39.6	73.4	1.62	4.05	1.72	2.47	0.01142199		1
P12004	PCNA	18	76.4	96.4	80.8	123.6	103.6	119.2	0.62	0.93	0.68	0.74	0.02266084		1
O94903	PROSC	11	95.5	87.1	93.8	104.5	112.9	106.2	0.91	0.77	0.88	0.86	0.01226387		1
Q07954	LRP1	11	80.3	61.4	65.8	119.7	138.6	134.2	0.67	0.44	0.49	0.53	0.00157904		1
Q32P28	P3H1	7	136.8	139.6	153.6	63.2	60.4	46.4	2.16	2.31	3.31	2.60	0.00029589		1
P13674	P4HA1	11	120.2	106.1	122.7	79.8	93.9	77.3	1.51	1.13	1.59	1.41	0.01107313		1
O15460	P4HA2	9	68.6	62.2	82	131.4	137.8	118	0.52	0.45	0.69	0.56	0.00213841		1
P48147	PREP	22	139.6	125.4	136.9	60.4	74.6	63.1	2.31	1.68	2.17	2.05	0.00038361		1
Q9H7Z7	PTGES2	15	96.2	104.9	100.4	103.8	95.1	99.6	0.93	1.10	1.01	1.01	0.79229317		1
Q14914	PTGR1	23	27.1	52.5	32.6	172.9	147.5	167.4	0.16	0.36	0.19	0.24	0.00032923		1
Q9UL46	PSME2	12	74	83.1	76.4	126	116.9	123.6	0.59	0.71	0.62	0.64	0.00032502		1

P61289	PSME3	13	76.5	89.4	85.5	123.5	110.6	114.5	0.62	0.81	0.75	0.72	0.00388742	1
Q14997	PSME4	9	115.4	104.4	125.6	84.6	95.6	74.4	1.36	1.09	1.69	1.38	0.0249811	1
O95456	PSMG1	11	174.9	123.3	167.6	25.1	76.7	32.4	6.97	1.61	5.17	4.58	0.0083511	1
Q969U7	PSMG2	7	151	115.1	148.1	49	84.9	51.9	3.08	1.36	2.85	2.43	0.00947843	1
Q5JS54	PSMG4	3	171.2	139.9	170.4	28.8	60.1	29.6	5.94	2.33	5.76	4.68	0.00114815	1
Q92530	PSMF1	7	80.4	90.2	124	119.6	109.8	76	0.67	0.82	1.63	1.04	0.85654456	1
P25786	PSMA1	25	106.4	97.4	107.4	93.6	102.6	92.6	1.14	0.95	1.16	1.08	0.17217146	1
P25787	PSMA2	13	112.5	99.5	111.6	87.5	100.5	88.4	1.29	0.99	1.26	1.18	0.05672757	1
P25788	PSMA3	16	104.5	97.1	111.3	95.5	102.9	88.7	1.09	0.94	1.25	1.10	0.2122076	1
P25789	PSMA4	15	106.4	100.9	115.1	93.6	99.1	84.9	1.14	1.02	1.36	1.17	0.06300684	1
P28066	PSMA5	9	112.4	99.4	108.7	87.6	100.6	91.3	1.28	0.99	1.19	1.15	0.0668395	1
P60900	PSMA6	19	101.8	97	104.3	98.2	103	95.7	1.04	0.94	1.09	1.02	0.5325317	1
O14818	PSMA7	16	108.2	99.9	104.5	91.8	100.1	95.5	1.18	1.00	1.09	1.09	0.06863979	1
P49721	PSMB2	16	116.2	105.5	106.3	83.8	94.5	93.7	1.39	1.12	1.13	1.21	0.018526	1
P49720	PSMB3	12	109.9	99.8	101.9	90.1	100.2	98.1	1.22	1.00	1.04	1.08	0.15017973	1
P28070	PSMB4	9	116.1	134.3	85.6	83.9	65.7	114.4	1.38	2.04	0.75	1.39	0.29826682	1
P28074	PSMB5	17	115	117.1	110.1	85	82.9	89.9	1.35	1.41	1.22	1.33	0.00066017	1
P28072	PSMB6	8	114.2	108.4	115.4	85.8	91.6	84.6	1.33	1.18	1.36	1.29	0.00115683	1
Q99436	PSMB7	8	117.1	100.9	115.3	82.9	99.1	84.7	1.41	1.02	1.36	1.26	0.03757986	1
P28065	PSMB9	5	88	111	85.3	112	89	114.7	0.79	1.25	0.74	0.93	0.41539612	1
Q8NFV4	ABHD11	6	112	129.5	116.4	88	70.5	83.6	1.27	1.84	1.39	1.50	0.00654668	1
Q8IVF2	AHNAK2	177	112.8	106.7	114.1	87.2	93.3	85.9	1.29	1.14	1.33	1.26	0.00225943	1
Q99873	PRMT1	12	62.9	79.1	84.3	137.1	120.9	115.7	0.46	0.65	0.73	0.61	0.00572455	1
O60678	PRMT3	3	56.8	84.2	60.6	143.2	115.8	139.4	0.40	0.73	0.43	0.52	0.00564576	1
O14744	PRMT5	17	122.4	115.7	117.3	77.6	84.3	82.7	1.58	1.37	1.42	1.46	0.00020652	1
Q9Y2B0	CNPY2	7	128.6	118	114	71.4	82	86	1.80	1.44	1.33	1.52	0.00279568	1
Q9BT09	CNPY3	3	161.6	134.6	139.9	38.4	65.4	60.1	4.21	2.06	2.33	2.86	0.00148067	1
Q9UKY7	CDV3	6	155	144.8	169.7	45	55.2	30.3	3.44	2.62	5.60	3.89	0.00038055	1
Q9P003	CNIH4	1	97.2	87.9	99.6	102.8	112.1	100.4	0.95	0.78	0.99	0.91	0.11330563	1
P30101	PDIA3	43	136.7	124.6	131.2	63.3	75.4	68.8	2.16	1.65	1.91	1.91	0.00023811	1
P13667	PDIA4	52	105.2	98.1	105.2	94.8	101.9	94.8	1.11	0.96	1.11	1.06	0.1656949	1
Q15084	PDIA6	19	96	107.1	110.2	104	92.9	89.8	0.92	1.15	1.23	1.10	0.21949569	1
Q96JJ7	TMX3	5	149.7	138.5	170.8	50.3	61.5	29.2	2.98	2.25	5.85	3.69	0.00137837	1
Q99497	PARK7	18	135.4	117.4	135.3	64.6	82.6	64.7	2.10	1.42	2.09	1.87	0.00226276	1
Q86VR2	FAM134C	2	56.1	89.2	82.5	143.9	110.8	117.5	0.39	0.81	0.70	0.63	0.02807833	1
Q96A26	FAM162A	7	129.7	119.9	125.2	70.3	80.1	74.8	1.84	1.50	1.67	1.67	0.00023933	1
Q9GZU8	FAM192A	1	72.1	100.3	71.1	127.9	99.7	128.9	0.56	1.01	0.55	0.71	0.04967547	1
Q96ND0	FAM210A	2	147.2	125.3	125.2	52.8	74.7	74.8	2.79	1.68	1.67	2.05	0.0032547	1
Q92520	FAM3C	11	156.6	162.9	135.4	43.4	37.1	64.6	3.61	4.39	2.10	3.37	0.00092846	1
Q9NUQ9	FAM49B	17	88.9	91	96.6	111.1	109	103.4	0.80	0.83	0.93	0.86	0.00851945	1
Q14320	FAM50A	4	108.7	95.5	145.7	91.3	104.5	54.3	1.19	0.91	2.68	1.60	0.19248942	1

Q658Y4	FAM91A1	2	142.6	94.8	143.9	57.4	105.2	56.1	2.48	0.90	2.57	1.98	0.07661738	1
Q13045	FLII	24	111.2	105.2	112.2	88.8	94.8	87.8	1.25	1.11	1.28	1.21	0.00350793	1
O94992	HEXIM1	4	87.2	80	92.3	112.8	120	107.7	0.77	0.67	0.86	0.77	0.00588171	1
Q86VS8	HOOK3	4	75.4	68.9	117.3	124.6	131.1	82.7	0.61	0.53	1.42	0.85	0.29860363	1
P17252	PRKCA	10	42.2	34.8	54.4	157.8	165.2	145.6	0.27	0.21	0.37	0.28	0.00015495	1
Q9UNF0	PACSIN2	10	76	74.6	88.2	124	125.4	111.8	0.61	0.59	0.79	0.67	0.00261114	1
P41743	PRKCI	3	127.6	119.2	136.8	72.4	80.8	63.2	1.76	1.48	2.16	1.80	0.00149058	1
Q8TBQ9	TMEM167	2	92.4	95.3	102.5	107.6	104.7	97.5	0.86	0.91	1.05	0.94	0.19871006	1
Q969X1	TMBIM1	2	104	93.9	103.5	96	106.1	96.5	1.08	0.89	1.07	1.01	0.85064375	1
Q86UE4	MTDH	15	114.9	95.2	129.7	85.1	104.8	70.3	1.35	0.91	1.84	1.37	0.13365808	1
Q7Z3U7	MON2	7	126.1	96.3	118.6	73.9	103.7	81.4	1.71	0.93	1.46	1.36	0.09692111	1
Q9BPW8	NIPSNAP1	10	80.7	91.3	77.3	119.3	108.7	122.7	0.68	0.84	0.63	0.72	0.00477494	1
O75323	GBAS	7	52.5	98.7	50.8	147.5	101.3	149.2	0.36	0.97	0.34	0.56	0.04221177	1
Q9UFN0	NIPSNAP3	9	119.2	72.5	124	80.8	127.5	76	1.48	0.57	1.63	1.23	0.67562884	1
Q8IWE2	FAM114A	10	106.2	97.6	104.2	93.8	102.4	95.8	1.13	0.95	1.09	1.06	0.22029014	1
Q96A00	PPP1R14A	2	132.9	109	140	67.1	91	60	1.98	1.20	2.33	1.84	0.01464317	1
Q6ZMI0	PPP1R21	4	150.9	120.6	137.9	49.1	79.4	62.1	3.07	1.52	2.22	2.27	0.00418996	1
Q15435	PPP1R7	7	127.2	114.3	130.5	72.8	85.7	69.5	1.75	1.33	1.88	1.65	0.00235537	1
O15355	PPM1G	15	100.3	98	103.2	99.7	102	96.8	1.01	0.96	1.07	1.01	0.66282508	1
O15258	RER1	4	130.6	127.1	139.6	69.4	72.9	60.4	1.88	1.74	2.31	1.98	0.00024939	1
Q9BY42	RTFDC1	4	57.4	90.1	103.3	142.6	109.9	96.7	0.40	0.82	1.07	0.76	0.16435363	1
P60903	S100A10	3	59.8	63.6	80.3	140.2	136.4	119.7	0.43	0.47	0.67	0.52	0.00196188	1
P31949	S100A11	4	77.3	67.3	79.1	122.7	132.7	120.9	0.63	0.51	0.65	0.60	0.00060775	1
Q96ER3	SAAL1	4	129.1	120.8	129.4	70.9	79.2	70.6	1.82	1.53	1.83	1.73	0.00018663	1
Q9UPN6	SCAF8	7	153	150.7	168.5	47	49.3	31.5	3.26	3.06	5.35	3.89	0.00013072	1
O75880	SCO1	5	102.5	101.3	98.3	97.5	98.7	101.7	1.05	1.03	0.97	1.01	0.47237207	1
O43819	SCO2	5	93.6	120.9	152.1	106.4	79.1	47.9	0.88	1.53	3.18	1.86	0.13676033	1
Q14160	SCRIB	23	99.3	96.7	107.2	100.7	103.3	92.8	0.99	0.94	1.16	1.03	0.65773213	1
P55735	SEC13	10	109.2	102.9	105.8	90.8	97.1	94.2	1.20	1.06	1.12	1.13	0.00977118	1
Q9UBV2	SEL1L	7	91.7	82.4	84.3	108.3	117.6	115.7	0.85	0.70	0.73	0.76	0.00229758	1
Q01105	SET	8	118.8	134.4	131.7	81.2	65.6	68.3	1.46	2.05	1.93	1.81	0.00114297	1
Q9Y2Z0	SUGT1	12	145.8	134	135.6	54.2	66	64.4	2.69	2.03	2.11	2.28	0.00012396	1
Q9H0W8	SMG9	15	168.1	87.8	176.5	31.9	112.2	23.5	5.27	0.78	7.51	4.52	0.09185966	1
A3KN83	SBNO1	8	138.9	109.8	114.4	61.1	90.2	85.6	2.27	1.22	1.34	1.61	0.03011511	1
Q92734	TFG	9	119.9	114.5	121	80.1	85.5	79	1.50	1.34	1.53	1.46	0.00020192	1
Q15436	SEC23A	17	93.5	79.6	84.5	106.5	120.4	115.5	0.88	0.66	0.73	0.76	0.00798347	1
Q15437	SEC23B	11	161.7	145.4	128.4	38.3	54.6	71.6	4.22	2.66	1.79	2.89	0.00266359	1
O95486	SEC24A	8	133.7	127.8	98.3	66.3	72.2	101.7	2.02	1.77	0.97	1.58	0.0616884	1
P53992	SEC24C	14	118	106.9	112.6	82	93.1	87.4	1.44	1.15	1.29	1.29	0.00527187	1
O94979	SEC31A	33	129.5	122.4	131.9	70.5	77.6	68.1	1.84	1.58	1.94	1.78	0.00015751	1
P60468	SEC61B	2	48.7	104	96.1	151.3	96	103.9	0.32	1.08	0.92	0.78	0.23472938	1

Q93096	PTP4A1	2	105.5	87.1	91.5	94.5	112.9	108.5	1.12	0.77	0.84	0.91	0.24801379	1
Q9H3U1	UNC45A	10	96.4	95.4	110.7	103.6	104.6	89.3	0.93	0.91	1.24	1.03	0.82322842	1
Q5BJH7	YIF1B	3	142.2	172.4	152.7	57.8	27.6	47.3	2.46	6.25	3.23	3.98	0.0008772	1
Q969M3	YIPF5	1	106.7	92.6	128.6	93.3	107.4	71.4	1.14	0.86	1.80	1.27	0.27754407	1
P22061	PCMT1	10	149.1	106.3	145.5	50.9	93.7	54.5	2.93	1.13	2.67	2.24	0.02557602	1
Q04941	PLP2	2	108.8	114.5	123.5	91.2	85.5	76.5	1.19	1.34	1.61	1.38	0.00671751	1
P06454	PTMA	5	89.4	79.6	87.7	110.6	120.4	112.3	0.81	0.66	0.78	0.75	0.00250973	1
Q96PZ0	PUS7	12	102.1	110.7	107	97.9	89.3	93	1.04	1.24	1.15	1.14	0.0199897	1
Q9UBP9	GULP1	4	125.4	132.8	38.5	74.6	67.2	161.5	1.68	1.98	0.24	1.30	0.96148415	1
Q14671	PUM1	9	144.4	130	155.7	55.6	70	44.3	2.60	1.86	3.51	2.66	0.00117929	1
P00491	PNP	21	79.3	106.7	81.5	120.7	93.3	118.5	0.66	1.14	0.69	0.83	0.15628281	1
Q6P158	DHX57	3	68.9	81.3	76.1	131.1	118.7	123.9	0.53	0.68	0.61	0.61	0.00064143	1
Q5T1J5	CHCHD2P	2	123.2	114	127.9	76.8	86	72.1	1.60	1.33	1.77	1.57	0.00167575	1
Q6P1N9	TATDN1	12	144.4	118.7	139.7	55.6	81.3	60.3	2.60	1.46	2.32	2.12	0.00358078	1
Q5SRD1	TIMM23B	5	88.5	103.1	82.8	111.5	96.9	117.2	0.79	1.06	0.71	0.85	0.11659779	1
P58557	YBEY	1	106.9	48.9	134.1	93.1	151.1	65.9	1.15	0.32	2.03	1.17	0.85892901	1
Q9Y383	LUC7L2	4	166.9	141.9	145.7	33.1	58.1	54.3	5.04	2.44	2.68	3.39	0.00072437	1
A8MWD9	SNRPGP1	2	134.6	169.4	136.1	65.4	30.6	63.9	2.06	5.54	2.13	3.24	0.00435456	1
O00764	PDXK	14	50.9	85.5	52.8	149.1	114.5	147.2	0.34	0.75	0.36	0.48	0.00965387	1
Q6P996	PDXDC1	8	67.8	52.5	43.7	132.2	147.5	156.3	0.51	0.36	0.28	0.38	0.00080686	1
Q9NVS9	PNPO	7	113	79.3	102.9	87	120.7	97.1	1.30	0.66	1.06	1.01	0.83183618	1
P32322	PYCR1	7	105.1	125.3	101	94.9	74.7	99	1.11	1.68	1.02	1.27	0.12005238	1
Q96C36	PYCR2	8	94.8	129.4	90.1	105.2	70.6	109.9	0.90	1.83	0.82	1.18	0.61532944	1
Q53H96	PYCRL	7	50.8	117.9	54.8	149.2	82.1	145.2	0.34	1.44	0.38	0.72	0.17235	1
P11177	PDHB	21	92.4	87.4	105	107.6	112.6	95	0.86	0.78	1.11	0.91	0.2430026	1
Q8NCN5	PDPR	8	123.3	109.2	131.4	76.7	90.8	68.6	1.61	1.20	1.92	1.58	0.00970365	1
Q08257	CRYZ	20	78.5	86.5	75	121.5	113.5	125	0.65	0.76	0.60	0.67	0.00114509	1
Q53FA7	TP53I3	16	33.4	82.9	30.3	166.6	117.1	169.7	0.20	0.71	0.18	0.36	0.01322438	1
P31150	GDI1	15	109.9	86.8	118.9	90.1	113.2	81.1	1.22	0.77	1.47	1.15	0.48464985	1
P50395	GDI2	28	106.5	100.5	105.5	93.5	99.5	94.5	1.14	1.01	1.12	1.09	0.03369595	1
Q15042	RAB3GAP	12	129.7	120.1	129	70.3	79.9	71	1.84	1.50	1.82	1.72	0.00027448	1
Q9H2M9	RAB3GAP	10	95.2	119	135.2	104.8	81	64.8	0.91	1.47	2.09	1.49	0.11549246	1
Q3YEC7	RABL6	6	100	73	127.9	100	127	72.1	1.00	0.57	1.77	1.12	0.97992611	1
P35241	RDX	35	107.4	105.6	110	92.6	94.4	90	1.16	1.12	1.22	1.17	0.00105558	1
Q14699	RFTN1	5	170.2	137.5	136.6	29.8	62.5	63.4	5.71	2.20	2.15	3.36	0.00353686	1
Q6IAA8	LAMTOR1	5	107.4	100.6	96.7	92.6	99.4	103.3	1.16	1.01	0.94	1.04	0.51764431	1
Q9UHA4	LAMTOR3	2	124.5	104.1	139	75.5	95.9	61	1.65	1.09	2.28	1.67	0.03457756	1
Q9H6Z4	RANBP3	9	129.4	127.7	143.3	70.6	72.3	56.7	1.83	1.77	2.53	2.04	0.00066377	1
P43487	RANBP1	10	142.8	118	141.5	57.2	82	58.5	2.50	1.44	2.42	2.12	0.00392037	1
Q8TEU7	RAPGEF6	4	134.3	132	148.5	65.7	68	51.5	2.04	1.94	2.88	2.29	0.00046708	1
P20936	RASA1	11	166.5	128.3	155.1	33.5	71.7	44.9	4.97	1.79	3.45	3.40	0.00335881	1

P46940	IQGAP1	88	121.8	114.5	122.4	78.2	85.5	77.6	1.56	1.34	1.58	1.49	0.00040258	1
Q13576	IQGAP2	10	5.6	34.4	15	194.4	165.6	185	0.03	0.21	0.08	0.11	0.00016823	1
Q15404	RSU1	17	89.8	110.7	85.4	110.2	89.3	114.6	0.81	1.24	0.75	0.93	0.44236284	1
Q70E73	RAPH1	4	48.5	64.9	67.2	151.5	135.1	132.8	0.32	0.48	0.51	0.44	0.00066876	1
P63000	RAC1	8	100.8	90.7	100.8	99.2	109.3	99.2	1.02	0.83	1.02	0.95	0.34164044	1
Q7L523	RRAGA	10	137.8	110	84.9	62.2	90	115.1	2.22	1.22	0.74	1.39	0.37005757	1
P61026	RAB10	14	72.8	57.9	72.7	127.2	142.1	127.3	0.57	0.41	0.57	0.52	0.00077564	1
P62491	RAB11A	2	100.5	86.1	93.4	99.5	113.9	106.6	1.01	0.76	0.88	0.88	0.08591981	1
Q6IQ22	RAB12	4	140.2	88.9	157	59.8	111.1	43	2.34	0.80	3.65	2.27	0.11858469	1
P51153	RAB13	6	86.5	82	90.2	113.5	118	109.8	0.76	0.69	0.82	0.76	0.00119856	1
P61106	RAB14	21	103.4	96.9	105.2	96.6	103.1	94.8	1.07	0.94	1.11	1.04	0.3618257	1
Q9NP72	RAB18	9	97.9	107.2	104.4	102.1	92.8	95.6	0.96	1.16	1.09	1.07	0.17932443	1
P62820	RAB1A	8	90.5	95.4	90.7	109.5	104.6	109.3	0.83	0.91	0.83	0.86	0.0023263	1
Q9H0U4	RAB1B	8	112.6	110.6	116.7	87.4	89.4	83.3	1.29	1.24	1.40	1.31	0.00046921	1
Q9UL25	RAB21	11	130.9	135.4	123.9	69.1	64.6	76.1	1.89	2.10	1.63	1.87	0.00022082	1
P61019	RAB2A	9	138.7	129.4	144.3	61.3	70.6	55.7	2.26	1.83	2.59	2.23	0.00025962	1
Q13637	RAB32	9	112.4	90.6	129.9	87.6	109.4	70.1	1.28	0.83	1.85	1.32	0.2441784	1
Q9BZG1	RAB34	6	44.9	57.4	61	155.1	142.6	139	0.29	0.40	0.44	0.38	0.00018979	1
P20337	RAB3B	5	153.1	159.4	100.2	46.9	40.6	99.8	3.26	3.93	1.00	2.73	0.04733597	1
P20339	RAB5A	7	104.9	105	95.1	95.1	95	104.9	1.10	1.11	0.91	1.04	0.5125432	1
P51148	RAB5C	8	79	42.3	76.5	121	157.7	123.5	0.65	0.27	0.62	0.51	0.01522919	1
P20340	RAB6A	7	104.8	81.2	109.6	95.2	118.8	90.4	1.10	0.68	1.21	1.00	0.8247873	1
Q9H0N0	RAB6C	1	106.1	130.6	99.3	93.9	69.4	100.7	1.13	1.88	0.99	1.33	0.14873642	1
P61006	RAB8A	6	119.2	104.4	121.9	80.8	95.6	78.1	1.48	1.09	1.56	1.38	0.01691424	1
Q92930	RAB8B	5	79	25.1	79.2	121	174.9	120.8	0.65	0.14	0.66	0.48	0.0377964	1
P11233	RALA	4	113.8	106.7	117.1	86.2	93.3	82.9	1.32	1.14	1.41	1.29	0.00445941	1
P11234	RALB	7	111.5	111.7	102.2	88.5	88.3	97.8	1.26	1.27	1.04	1.19	0.01876643	1
P61224	RAP1B	4	123.3	114.6	125.5	76.7	85.4	74.5	1.61	1.34	1.68	1.54	0.00085073	1
P61225	RAP2B	5	127.2	119.4	125.6	72.8	80.6	74.4	1.75	1.48	1.69	1.64	0.00013858	1
P62070	RRAS2	5	86.5	97.2	90.5	113.5	102.8	109.5	0.76	0.95	0.83	0.84	0.0175955	1
Q00765	REEP5	8	95.7	88.3	96.1	104.3	111.7	103.9	0.92	0.79	0.92	0.88	0.02085065	1
P63244	RACK1	26	91.2	118.1	101.9	108.8	81.9	98.1	0.84	1.44	1.04	1.11	0.53655624	1
O00559	EBAG9	2	116.9	47.3	95.3	83.1	152.7	104.7	1.41	0.31	0.91	0.88	0.40582667	1
Q9NQG5	RPRD1B	11	130.5	100.7	130	69.5	99.3	70	1.88	1.01	1.86	1.58	0.04287301	1
Q5VT52	RPRD2	12	105.6	110.8	134.9	94.4	89.2	65.1	1.12	1.24	2.07	1.48	0.0552657	1
O43665	RGS10	7	142.8	147.5	130.8	57.2	52.5	69.2	2.50	2.81	1.89	2.40	0.00032846	1
Q96DB5	RMDN1	10	165.5	95.1	180.3	34.5	104.9	19.7	4.80	0.91	9.15	4.95	0.06486091	1
Q8WUF5	PPP1R13L	18	54.9	60.3	41.3	145.1	139.7	158.7	0.38	0.43	0.26	0.36	0.00027938	1
Q96T23	RSF1	4	113.8	123.3	131.4	86.2	76.7	68.6	1.32	1.61	1.92	1.61	0.00315293	1
O75787	ATP6AP2	4	118	131.1	84	82	68.9	116	1.44	1.90	0.72	1.36	0.32857964	1
P35249	RFC4	11	158.9	126.7	139.4	41.1	73.3	60.6	3.87	1.73	2.30	2.63	0.0032585	1

P15927	RPA2	11	134.1	121.4	135.4	65.9	78.6	64.6	2.03	1.54	2.10	1.89	0.00065934	1
O95197	RTN3	3	82	75.8	87.3	118	124.2	112.7	0.69	0.61	0.77	0.69	0.00146621	1
Q9HB40	SCPEP1	3	125.1	101.1	125.7	74.9	98.9	74.3	1.67	1.02	1.69	1.46	0.03917131	1
Q8TC12	RDH11	15	120.8	106.9	119.5	79.2	93.1	80.5	1.53	1.15	1.48	1.39	0.00738684	1
Q8NBN7	RDH13	3	84.3	13.2	61.6	115.7	186.8	138.4	0.73	0.07	0.45	0.41	0.03392665	1
Q9HBH5	RDH14	9	88.6	85.9	82.4	111.4	114.1	117.6	0.80	0.75	0.70	0.75	0.00034707	1
P52565	ARHGDIA	10	102.7	105.5	96.6	97.3	94.5	103.4	1.06	1.12	0.93	1.04	0.43768974	1
Q8N392	ARHGAP1	6	109.8	106.7	101.9	90.2	93.3	98.1	1.22	1.14	1.04	1.13	0.0195299	1
Q13017	ARHGAP5	4	57.9	68.8	91.1	142.1	131.2	108.9	0.41	0.52	0.84	0.59	0.0165948	1
Q92888	ARHGEF1	8	118.5	115	69.1	81.5	85	130.9	1.45	1.35	0.53	1.11	0.94231356	1
O15013	ARHGEF1	8	100.3	94.4	137.1	99.7	105.6	62.9	1.01	0.89	2.18	1.36	0.32460792	1
Q9NZN5	ARHGEF1	4	117.5	121	172.6	82.5	79	27.4	1.42	1.53	6.30	3.09	0.04237556	1
Q92974	ARHGEF2	7	155.3	183.8	136.8	44.7	16.2	63.2	3.47	11.35	2.16	5.66	0.00372988	1
Q7L0Q8	RHOU	1	102.5	99.2	104	97.5	100.8	96	1.05	0.98	1.08	1.04	0.13095187	1
O75792	RNASEH2	6	126.4	109.6	109.8	73.6	90.4	90.2	1.72	1.21	1.22	1.38	0.01786715	1
P13489	RNH1	16	48.7	78.1	96.2	151.3	121.9	103.8	0.32	0.64	0.93	0.63	0.05864252	1
O75817	POP7	1	114.9	43.5	138.4	85.1	156.5	61.6	1.35	0.28	2.25	1.29	0.96037425	1
O95707	POP4	5	115.4	102.1	91	84.6	97.9	109	1.36	1.04	0.83	1.08	0.60033829	1
P78346	RPP30	6	113.7	93.1	85.5	86.3	106.9	114.5	1.32	0.87	0.75	0.98	0.68875396	1
P52758	HRSP12	5	122.4	131.9	122.8	77.6	68.1	77.2	1.58	1.94	1.59	1.70	0.00030353	1
Q99575	POP1	13	129.9	120.1	131.8	70.1	79.9	68.2	1.85	1.50	1.93	1.76	0.00044225	1
P23921	RRM1	23	88.2	85.2	93.9	111.8	114.8	106.1	0.79	0.74	0.89	0.81	0.00378543	1
P49247	RPIA	8	120.4	102.8	138	79.6	97.2	62	1.51	1.06	2.23	1.60	0.04690651	1
P60891	PRPS1	10	88.6	105.7	83.8	111.4	94.3	116.2	0.80	1.12	0.72	0.88	0.19529688	1
P11908	PRPS2	5	93.1	104.4	99.5	106.9	95.6	100.5	0.87	1.09	0.99	0.98	0.68782846	1
O76021	RSL1D1	6	157.9	160.4	110.9	42.1	39.6	89.1	3.75	4.05	1.24	3.02	0.01938452	1
P51812	RPS6KA3	10	103.9	89.1	113.9	96.1	110.9	86.1	1.08	0.80	1.32	1.07	0.67500626	1
Q92979	EMG1	11	128.3	115.3	135.5	71.7	84.7	64.5	1.79	1.36	2.10	1.75	0.00322848	1
Q14137	BOP1	4	89.5	83	89.9	110.5	117	110.1	0.81	0.71	0.82	0.78	0.00137157	1
Q9Y3A5	SBDS	16	145.2	129.9	150.5	54.8	70.1	49.5	2.65	1.85	3.04	2.51	0.00066152	1
Q9P2E9	RRBP1	57	77.1	66.1	74.7	122.9	133.9	125.3	0.63	0.49	0.60	0.57	0.00031666	1
Q969S9	GFM2	2	117.3	103.1	123	82.7	96.9	77	1.42	1.06	1.60	1.36	0.02586937	1
P16083	NQO2	10	118.7	105.6	110.1	81.3	94.4	89.9	1.46	1.12	1.22	1.27	0.01347946	1
Q9Y5B0	CTDP1	6	141.6	125	137	58.4	75	63	2.42	1.67	2.17	2.09	0.00059124	1
Q9NP77	SSU72	7	137.2	112.1	170.3	62.8	87.9	29.7	2.18	1.28	5.73	3.06	0.02869514	1
Q8N7H5	PAF1	8	132.4	121.7	133.1	67.6	78.3	66.9	1.96	1.55	1.99	1.83	0.00036908	1
Q6PD62	CTR9	5	113.1	106.7	150.9	86.9	93.3	49.1	1.30	1.14	3.07	1.84	0.07302532	1
Q8IZ73	RPUSD2	2	118.2	79.3	121.7	81.8	120.7	78.3	1.44	0.66	1.55	1.22	0.54178557	1
P98175	RBM10	5	159.2	135.8	176.6	40.8	64.2	23.4	3.90	2.12	7.55	4.52	0.00238562	1
Q5T8P6	RBM26	4	139.4	134	148.2	60.6	66	51.8	2.30	2.03	2.86	2.40	0.00015745	1
Q9P2N5	RBM27	3	179.3	159.4	182.2	20.7	40.6	17.8	8.66	3.93	10.24	7.61	0.00013039	1

Q9BWF3	RBM4	6	139.2	124.5	126.7	60.8	75.5	73.3	2.29	1.65	1.73	1.89	0.00074091		1
Q96DH6	MSI2	3	75.5	36.5	63.4	124.5	163.5	136.6	0.61	0.22	0.46	0.43	0.00700055		1
Q9NRX1	PNO1	10	89.3	127	97.6	110.7	73	102.4	0.81	1.74	0.95	1.17	0.59736809		1
Q9UKM9	RALY	9	155.5	142.7	182.3	44.5	57.3	17.7	3.49	2.49	10.30	5.43	0.0018792		1
P22087	FBL	15	127.5	138.5	111	72.5	61.5	89	1.76	2.25	1.25	1.75	0.01047956		1
Q5JTH9	RRP12	15	106.1	115.5	126.1	93.9	84.5	73.9	1.13	1.37	1.71	1.40	0.01765537		1
Q96T51	RUFY1	9	103	94.2	121.1	97	105.8	78.9	1.06	0.89	1.53	1.16	0.33720077		1
P82979	SARNP	7	117.6	66.6	101.6	82.4	133.4	98.4	1.43	0.50	1.03	0.99	0.67966477		1
P16615	ATP2A2	35	90.9	82.4	86.5	109.1	117.6	113.5	0.83	0.70	0.76	0.77	0.00151454		1
Q14151	SAFB2	6	182	158.2	172.3	18	41.8	27.7	10.11	3.78	6.22	6.71	0.00013161		1
Q8WVM8	SCFD1	12	125.4	115	120.8	74.6	85	79.2	1.68	1.35	1.53	1.52	0.0006612		1
Q9Y6Y8	SEC23IP	17	112.6	103.4	110.2	87.4	96.6	89.8	1.29	1.07	1.23	1.20	0.01096685		1
O15126	SCAMP1	7	73.4	73.7	86.6	126.6	126.3	113.4	0.58	0.58	0.76	0.64	0.00198929		1
O15127	SCAMP2	4	99.5	91.7	108.9	100.5	108.3	91.1	0.99	0.85	1.20	1.01	0.99288977		1
O14828	SCAMP3	7	89.8	72.3	81.8	110.2	127.7	118.2	0.81	0.57	0.69	0.69	0.00639018		1
Q6UXD5	SEZ6L2	3	150.1	129.5	136.9	49.9	70.5	63.1	3.01	1.84	2.17	2.34	0.00080347		1
Q15019	2-Sep	17	156.8	126.6	121.7	43.2	73.4	78.3	3.63	1.72	1.55	2.30	0.01070425		1
Q9UHD8	9-Sep	25	100.2	89.2	95.3	99.8	110.8	104.7	1.00	0.81	0.91	0.91	0.0860262		1
Q13501	SQSTM1	10	123.2	57.7	90.6	76.8	142.3	109.4	1.60	0.41	0.83	0.95	0.5166049		1
O43464	HTRA2	5	145.2	126.9	162.9	54.8	73.1	37.1	2.65	1.74	4.39	2.93	0.00360285		1
Q9UQ35	SRRM2	24	163	149.2	170.3	37	50.8	29.7	4.41	2.94	5.73	4.36	0.00015507		1
Q07955	SRSF1	12	108.6	98.8	110.7	91.4	101.2	89.3	1.19	0.98	1.24	1.13	0.08053965		1
Q05519	SRSF11	4	144.4	125.2	157.7	55.6	74.8	42.3	2.60	1.67	3.73	2.67	0.00313013		1
Q01130	SRSF2	4	105.2	91.2	106.7	94.8	108.8	93.3	1.11	0.84	1.14	1.03	0.7819054		1
Q13243	SRSF5	2	153.4	143	183.3	46.6	57	16.7	3.29	2.51	10.98	5.59	0.00217684		1
Q16629	SRSF7	8	119.6	79.9	114.8	80.4	120.1	85.2	1.49	0.67	1.35	1.17	0.61858529		1
O94804	STK10	4	89.9	167	158	110.1	33	42	0.82	5.06	3.76	3.21	0.09006905		1
Q9Y5S2	CDC42BPI	4	91.5	93.6	107.2	108.5	106.4	92.8	0.84	0.88	1.16	0.96	0.50166223		1
Q16512	PKN1	4	123.8	101.3	97.1	76.2	98.7	102.9	1.62	1.03	0.94	1.20	0.27535238		1
Q8TD19	NEK9	5	23.4	108.4	119.1	176.6	91.6	80.9	0.13	1.18	1.47	0.93	0.48716206		1
O96013	PAK4	8	125.8	132.4	136.6	74.2	67.6	63.4	1.70	1.96	2.15	1.94	0.00014212		1
Q96Q15	SMG1	1	142.3	111.5	114	57.7	88.5	86	2.47	1.26	1.33	1.68	0.03179057		1
Q9H4A3	WNK1	29	140.6	128.2	138.8	59.4	71.8	61.2	2.37	1.79	2.27	2.14	0.00019534		1
Q96QC0	PPP1R10	2	71.5	143.4	157.5	128.5	56.6	42.5	0.56	2.53	3.71	2.27	0.26921552		1
Q14738	PPP2R5D	7	144.5	139.2	161.7	55.5	60.8	38.3	2.60	2.29	4.22	3.04	0.00054227		1
P30153	PPP2R1A	22	110.1	103.1	105.5	89.9	96.9	94.5	1.22	1.06	1.12	1.14	0.0127226		1
Q15257	PPP2R4	4	132.8	162.5	122	67.2	37.5	78	1.98	4.33	1.56	2.62	0.01028586		1
P67775	PPP2CA	5	109	114.2	108	91	85.8	92	1.20	1.33	1.17	1.23	0.00156643		1
Q08209	PPP3CA	8	88	83.7	93	112	116.3	107	0.79	0.72	0.87	0.79	0.00345715		1
P60510	PPP4C	6	133.9	118.8	129.2	66.1	81.2	70.8	2.03	1.46	1.82	1.77	0.00098082		1
Q9NY27	PPP4R2	5	116.5	94.1	127.3	83.5	105.9	72.7	1.40	0.89	1.75	1.34	0.14166099		1

O00743	PPP6C	8	108	114.5	116.2	92	85.5	83.8	1.17	1.34	1.39	1.30	0.00187118	1
Q5H9R7	PPP6R3	4	128.2	143.5	128	71.8	56.5	72	1.79	2.54	1.78	2.03	0.00079018	1
Q9BRF8	CPPED1	12	11.7	54.4	19.3	188.3	145.6	180.7	0.06	0.37	0.11	0.18	0.0015363	1
P62136	PPP1CA	5	127.6	104.5	122.5	72.4	95.5	77.5	1.76	1.09	1.58	1.48	0.02132385	1
P62140	PPP1CB	7	125.4	100.8	121.4	74.6	99.2	78.6	1.68	1.02	1.54	1.41	0.04221125	1
P49591	SARS	21	131.6	134.1	150.6	68.4	65.9	49.4	1.92	2.03	3.05	2.34	0.00077611	1
P35237	SERPINB6	20	144.8	124.5	154.5	55.2	75.5	45.5	2.62	1.65	3.40	2.56	0.00272632	1
P50454	SERPINH1	16	72.1	102.8	105	127.9	97.2	95	0.56	1.06	1.11	0.91	0.42266558	1
Q9UBL3	ASH2L	1	140.1	128.1	115.6	59.9	71.9	84.4	2.34	1.78	1.37	1.83	0.00504048	1
P10768	ESD	15	100.4	90	100.6	99.6	110	99.4	1.01	0.82	1.01	0.95	0.29220021	1
A1X283	SH3PXD2I	3	117	94	74.9	83	106	125.1	1.41	0.89	0.60	0.97	0.61400448	1
Q9H299	SH3BGRL	2	168.6	90.5	172.4	31.4	109.5	27.6	5.37	0.83	6.25	4.15	0.08090128	1
Q96B97	SH3KBP1	16	155	130.4	140.3	45	69.6	59.7	3.44	1.87	2.35	2.56	0.00115501	1
A0MZ66	SHTN1	13	78.3	75	86.5	121.7	125	113.5	0.64	0.60	0.76	0.67	0.00115017	1
O75911	DHRS3	9	35.1	3.4	29.9	164.9	196.6	170.1	0.21	0.02	0.18	0.14	0.00037172	1
Q9NR45	NANS	5	145.4	127.7	162.3	54.6	72.3	37.7	2.66	1.77	4.31	2.91	0.00307905	1
Q9NRA2	SLC17A5	2	115.2	138.4	120.2	84.8	61.6	79.8	1.36	2.25	1.51	1.70	0.00784387	1
Q9H9B4	SFXN1	17	85.3	93.7	89.4	114.7	106.3	110.6	0.74	0.88	0.81	0.81	0.00356172	1
Q9BWM7	SFXN3	13	80.3	101.7	72.9	119.7	98.3	127.1	0.67	1.03	0.57	0.76	0.06953485	1
Q99720	SIGMAR1	4	99.5	104.4	97.1	100.5	95.6	102.9	0.99	1.09	0.94	1.01	0.83704377	1
P67812	SEC11A	5	107.1	97.1	112.6	92.9	102.9	87.4	1.15	0.94	1.29	1.13	0.15581472	1
Q9Y6A9	SPCS1	3	108	97.3	114.8	92	102.7	85.2	1.17	0.95	1.35	1.16	0.13635719	1
P37108	SRP14	7	141.2	106.1	143.9	58.8	93.9	56.1	2.40	1.13	2.57	2.03	0.0242047	1
P09132	SRP19	5	105.9	84.8	108.9	94.1	115.2	91.1	1.13	0.74	1.20	1.02	0.98135249	1
P49458	SRP9	3	121	65	104.3	79	135	95.7	1.53	0.48	1.09	1.03	0.79659371	1
Q9Y5M8	SRPRB	15	129.9	121.4	130.9	70.1	78.6	69.1	1.85	1.54	1.89	1.76	0.00021099	1
Q9UHB9	SRP68	10	122.8	114.9	125	77.2	85.1	75	1.59	1.35	1.67	1.54	0.00064794	1
P40763	STAT3	12	56.7	55.1	104.5	143.3	144.9	95.5	0.40	0.38	1.09	0.62	0.07162437	1
Q92783	STAM	4	122.8	113.5	121.3	77.2	86.5	78.7	1.59	1.31	1.54	1.48	0.00070817	1
Q04837	SSBP1	9	90.9	95.7	100.6	109.1	104.3	99.4	0.83	0.92	1.01	0.92	0.09745119	1
Q29RF7	PDS5A	17	165.2	29.5	165.2	34.8	170.5	34.8	4.75	0.17	4.75	3.22	0.56629878	1
O00193	SMAP	3	84.8	73.5	105	115.2	126.5	95	0.74	0.58	1.11	0.81	0.13362863	1
Q0VAQ4	SMAGP	1	186.5	120.2	75.9	13.5	79.8	124.1	13.81	1.51	0.61	5.31	0.29233914	1
O43765	SGTA	10	143.7	132.6	154.6	56.3	67.4	45.4	2.55	1.97	3.41	2.64	0.00062821	1
Q8N5G0	SMIM20	2	140.4	63.1	116.5	59.6	136.9	83.5	2.36	0.46	1.40	1.40	0.70104853	1
P62318	SNRPD3	3	128.7	128.5	143.4	71.3	71.5	56.6	1.81	1.80	2.53	2.05	0.00065488	1
P14678	SNRPB	1	123.7	78.3	104.7	76.3	121.7	95.3	1.62	0.64	1.10	1.12	0.82217681	1
P61956	SUMO2	3	119.4	151.8	156.9	80.6	48.2	43.1	1.48	3.15	3.64	2.76	0.00677942	1
O95295	SNAPIN	2	112.2	83.4	106.2	87.8	116.6	93.8	1.28	0.72	1.13	1.04	0.92759813	1
Q13573	SNW1	7	158.5	139.7	152.5	41.5	60.3	47.5	3.82	2.32	3.21	3.12	0.00021381	1
Q9Y6M7	SLC4A7	8	127.7	107.9	143.8	72.3	92.1	56.2	1.77	1.17	2.56	1.83	0.02265081	1

P05023	ATP1A1	33	106.2	97.9	106.9	93.8	102.1	93.1	1.13	0.96	1.15	1.08	0.14725949	1
P54709	ATP1B3	9	147.7	132.2	149	52.3	67.8	51	2.82	1.95	2.92	2.57	0.00035439	1
Q8NBW4	SLC38A9	2	116.1	84	105.7	83.9	116	94.3	1.38	0.72	1.12	1.08	0.7868363	1
P11166	SLC2A1	6	82.5	76.8	83.5	117.5	123.2	116.5	0.70	0.62	0.72	0.68	0.00020688	1
Q96BI1	SLC22A18	1	75.9	81.2	93.9	124.1	118.8	106.1	0.61	0.68	0.89	0.73	0.01239645	1
Q13596	SNX1	7	97.2	95.8	101	102.8	104.2	99	0.95	0.92	1.02	0.96	0.14276239	1
Q9UMY4	SNX12	7	108.2	108.2	137.3	91.8	91.8	62.7	1.18	1.18	2.19	1.52	0.05943658	1
O60749	SNX2	16	130.7	131.9	122.5	69.3	68.1	77.5	1.89	1.94	1.58	1.80	0.00017014	1
O60493	SNX3	9	135.6	143.2	128.3	64.4	56.8	71.7	2.11	2.52	1.79	2.14	0.00030143	1
Q9Y5X3	SNX5	8	33.7	81.6	110.6	166.3	118.4	89.4	0.20	0.69	1.24	0.71	0.19424875	1
Q8N0X7	SPG20	8	109.3	98.4	120.5	90.7	101.6	79.5	1.21	0.97	1.52	1.23	0.10558428	1
Q9NUQ6	SPATS2L	1	129.3	119.8	133.2	70.7	80.2	66.8	1.83	1.49	1.99	1.77	0.0006199	1
Q13813	SPTAN1	66	91.6	76.5	82.7	108.4	123.5	117.3	0.85	0.62	0.71	0.72	0.00611764	1
Q01082	SPTBN1	23	102.9	94.8	99.1	97.1	105.2	100.9	1.06	0.90	0.98	0.98	0.55421968	1
Q96R06	SPAG5	3	177.1	127.7	121.7	22.9	72.3	78.3	7.73	1.77	1.55	3.68	0.02734222	1
P19623	SRM	18	95.6	101	90.6	104.4	99	109.4	0.92	1.02	0.83	0.92	0.11488509	1
P63208	SKP1	10	124	172.6	113.7	76	27.4	86.3	1.63	6.30	1.32	3.08	0.04579336	1
O15121	DEGS1	3	117.2	102.7	100.3	82.8	97.3	99.7	1.42	1.06	1.01	1.16	0.14558938	1
Q13838	DDX39B	14	146.2	130.6	133.2	53.8	69.4	66.8	2.72	1.88	1.99	2.20	0.00042508	1
Q15637	SF1	16	115.2	115.1	120.4	84.8	84.9	79.6	1.36	1.36	1.51	1.41	0.00016656	1
Q15459	SF3A1	33	139.5	130.6	143.5	60.5	69.4	56.5	2.31	1.88	2.54	2.24	0.00014905	1
Q15393	SF3B3	45	128.1	119.6	128.1	71.9	80.4	71.9	1.78	1.49	1.78	1.68	0.00022756	1
Q9Y3B4	SF3B6	4	135.7	132	95.6	64.3	68	104.4	2.11	1.94	0.92	1.66	0.08005758	1
Q01081	U2AF1	5	69.6	96.3	73.5	130.4	103.7	126.5	0.53	0.93	0.58	0.68	0.0265142	1
P26368	U2AF2	8	152.6	134	140.4	47.4	66	59.6	3.22	2.03	2.36	2.54	0.00039182	1
O95104	SCAF4	3	152.2	147.8	169.2	47.8	52.2	30.8	3.18	2.83	5.49	3.84	0.00025711	1
Q8WXA9	SREK1	3	119.4	138.8	136.2	80.6	61.2	63.8	1.48	2.27	2.13	1.96	0.00185377	1
P37268	FDFT1	11	165	132.6	109.2	35	67.4	90.8	4.71	1.97	1.20	2.63	0.0358008	1
Q15020	SART3	18	145.9	116.9	138.4	54.1	83.1	61.6	2.70	1.41	2.25	2.12	0.00536552	1
Q9GZT3	SLIRP	5	99.7	86.4	65.8	100.3	113.6	134.2	0.99	0.76	0.49	0.75	0.08300132	1
Q14247	CTTN	31	106.2	99.1	110.7	93.8	100.9	89.3	1.13	0.98	1.24	1.12	0.08923577	1
Q7KZF4	SND1	47	93	87.6	92.5	107	112.4	107.5	0.87	0.78	0.86	0.84	0.00181467	1
Q9HD15	SRA1	7	161.6	125.3	155.5	38.4	74.7	44.5	4.21	1.68	3.49	3.13	0.00392639	1
Q15738	NSDHL	7	121.1	133.2	88.9	78.9	66.8	111.1	1.53	1.99	0.80	1.44	0.19829307	1
Q9UJZ1	STOML2	18	167.2	116.9	124	32.8	83.1	76	5.10	1.41	1.63	2.71	0.03160918	1
P38646	HSPA9	46	127.1	118.8	124.7	72.9	81.2	75.3	1.74	1.46	1.66	1.62	0.00017442	1
P31948	STIP1	55	113.4	106.9	114.3	86.6	93.1	85.7	1.31	1.15	1.33	1.26	0.00219603	1
P31040	SDHA	23	116.6	104.8	114.5	83.4	95.2	85.5	1.40	1.10	1.34	1.28	0.0096136	1
P21912	SDHB	11	130.6	105.8	129.9	69.4	94.2	70.1	1.88	1.12	1.85	1.62	0.01855951	1
P53597	SUCLG1	12	64.3	102.9	65.3	135.7	97.1	134.7	0.47	1.06	0.48	0.67	0.06642155	1
Q8NBJ7	SUMF2	11	123.3	91	126.5	76.7	109	73.5	1.61	0.83	1.72	1.39	0.1650492	1

Q6ZRP7	QSOX2	7	99.1	71.9	102.6	100.9	128.1	97.4	0.98	0.56	1.05	0.87	0.26890198	1
P0DMN0	SULT1A4	17	19.8	89.3	28	180.2	110.7	172	0.11	0.81	0.16	0.36	0.02485168	1
Q9UBE0	SAE1	15	116.9	100.2	117.1	83.1	99.8	82.9	1.41	1.00	1.41	1.27	0.04506832	1
Q9UBT2	UBA2	29	123.4	115.7	123	76.6	84.3	77	1.61	1.37	1.60	1.53	0.00030546	1
P63279	UBE2I	5	108.8	85.1	126.7	91.2	114.9	73.3	1.19	0.74	1.73	1.22	0.46541032	1
P42285	SKIV2L2	9	128.9	130	157.9	71.1	70	42.1	1.81	1.86	3.75	2.47	0.00438714	1
P00441	SOD1	8	111	99.1	112.4	89	100.9	87.6	1.25	0.98	1.28	1.17	0.06579221	1
O15260	SURF4	3	102.1	94.8	104.4	97.9	105.2	95.6	1.04	0.90	1.09	1.01	0.84264107	1
Q16637	SMN1	4	108.2	27.1	71.5	91.8	172.9	128.5	1.18	0.16	0.56	0.63	0.13423674	1
Q92925	SMARCD2	2	169.3	109	165.8	30.7	91	34.2	5.51	1.20	4.85	3.85	0.02544598	1
Q9UH65	SWAP70	8	134.8	109.8	100.3	65.2	90.2	99.7	2.07	1.22	1.01	1.43	0.10880028	1
Q92797	SYMPK	22	152.5	136.2	146.9	47.5	63.8	53.1	3.21	2.13	2.77	2.70	0.00018105	1
Q99536	VAT1	15	38.7	69.9	82.6	161.3	130.1	117.4	0.24	0.54	0.70	0.49	0.01706813	1
O43760	SYNGR2	5	69.6	98.2	70.5	130.4	101.8	129.5	0.53	0.96	0.54	0.68	0.03627287	1
O95721	SNAP29	5	117.5	62.6	110.3	82.5	137.4	89.7	1.42	0.46	1.23	1.04	0.80574681	1
Q9NPQ8	RIC8A	4	107.4	114.2	113.6	92.6	85.8	86.4	1.16	1.33	1.31	1.27	0.00158122	1
Q9UMZ2	SYNRG	3	127.7	105.1	118.5	72.3	94.9	81.5	1.77	1.11	1.45	1.44	0.02110007	1
O60499	STX10	5	152.8	108.9	149.3	47.2	91.1	50.7	3.24	1.20	2.94	2.46	0.02056995	1
Q86Y82	STX12	6	122.3	94.9	119.2	77.7	105.1	80.8	1.57	0.90	1.48	1.32	0.11870183	1
Q12846	STX4	8	27.3	34.8	101.7	172.7	165.2	98.3	0.16	0.21	1.03	0.47	0.05326728	1
O43752	STX6	5	164.9	129.2	84.6	35.1	70.8	115.4	4.70	1.82	0.73	2.42	0.18546237	1
O15400	STX7	8	151.2	134.5	159.1	48.8	65.5	40.9	3.10	2.05	3.89	3.01	0.00070986	1
P61764	STXBP1	3	1.3	66.7	60.1	198.7	133.3	139.9	0.01	0.50	0.43	0.31	0.01756776	1
Q15833	STXBP2	2	108.8	97.6	123.7	91.2	102.4	76.3	1.19	0.95	1.62	1.26	0.13374664	1
O00186	STXBP3	3	107.9	85.1	125.2	92.1	114.9	74.8	1.17	0.74	1.67	1.20	0.5010075	1
O00560	SDCBP	8	122.9	101.4	121.4	77.1	98.6	78.6	1.59	1.03	1.54	1.39	0.03592083	1
Q9Y490	TLN1	138	123.4	117.1	124.3	76.6	82.9	75.7	1.61	1.41	1.64	1.56	0.00017489	1
Q9Y4G6	TLN2	10	36.8	32.6	74.7	163.2	167.4	125.3	0.23	0.19	0.60	0.34	0.00536587	1
Q13148	TARDBP	11	139	125.1	136.5	61	74.9	63.5	2.28	1.67	2.15	2.03	0.00037665	1
O60784	TOM1	9	97.8	69.3	90.5	102.2	130.7	109.5	0.96	0.53	0.83	0.77	0.07950924	1
P82094	TMF1	4	125.8	100.3	124.2	74.2	99.7	75.8	1.70	1.01	1.64	1.45	0.04522033	1
Q8TC07	TBC1D15	9	147.7	166.2	163.1	52.3	33.8	36.9	2.82	4.92	4.42	4.05	0.0001285	1
Q9HA65	TBC1D17	2	41.4	71.3	109.5	158.6	128.7	90.5	0.26	0.55	1.21	0.67	0.13624404	1
Q92609	TBC1D5	4	140.7	110	131.4	59.3	90	68.6	2.37	1.22	1.92	1.84	0.0130723	1
Q16650	TBR1	1	112.7	103.3	108.5	87.3	96.7	91.5	1.29	1.07	1.19	1.18	0.01317698	1
P78371	CCT2	45	160.1	140.3	145.2	39.9	59.7	54.8	4.01	2.35	2.65	3.00	0.00032332	1
O43156	TTI1	3	158.7	133.2	133.3	41.3	66.8	66.7	3.84	1.99	2.00	2.61	0.00224318	1
Q9BUR4	WRAP53	2	57.1	128.6	128	142.9	71.4	72	0.40	1.80	1.78	1.33	0.79900586	1
Q9Y4R8	TELO2	2	89.6	52.4	111	110.4	147.6	89	0.81	0.36	1.25	0.80	0.26527498	1
Q9NYB0	TERF2IP	1	136.8	121.4	105.7	63.2	78.6	94.3	2.16	1.54	1.12	1.61	0.02843127	1
Q68CZ2	TNS3	22	8.6	42.6	21.6	191.4	157.4	178.4	0.04	0.27	0.12	0.15	0.00041497	1

Q6PGP7	TTC37	7	97.8	71.9	115.6	102.2	128.1	84.4	0.96	0.56	1.37	0.96	0.61396785	1
Q8N5M4	TTC9C	5	119.5	27.6	96.8	80.5	172.4	103.2	1.48	0.16	0.94	0.86	0.39282337	1
P52888	THOP1	8	162.8	139.4	153.8	37.2	60.6	46.2	4.38	2.30	3.33	3.34	0.00041822	1
P51580	TPMT	11	146.3	109.8	130.6	53.7	90.2	69.4	2.72	1.22	1.88	1.94	0.01804931	1
O95881	TXNDC12	3	140.2	128.2	80.7	59.8	71.8	119.3	2.34	1.79	0.68	1.60	0.27162362	1
O14530	TXNDC9	6	165.6	129.9	173.6	34.4	70.1	26.4	4.81	1.85	6.58	4.41	0.00404282	1
O43396	TXNL1	18	137.4	108.2	131.7	62.6	91.8	68.3	2.19	1.18	1.93	1.77	0.01512598	1
Q9H3N1	TMX1	9	81.4	72.2	90.7	118.6	127.8	109.3	0.69	0.56	0.83	0.69	0.00794868	1
Q9Y320	TMX2	4	77.2	85.4	3.9	122.8	114.6	196.1	0.63	0.75	0.02	0.46	0.07205548	1
Q16762	TST	11	18.3	40.8	36.2	181.7	159.2	163.8	0.10	0.26	0.22	0.19	0.00014848	1
Q96FV9	THOC1	7	163.4	177.5	155.9	36.6	22.5	44.1	4.46	7.89	3.54	5.30	0.00012624	1
Q86V81	ALYREF	5	100.9	91.5	108.8	99.1	108.5	91.2	1.02	0.84	1.19	1.02	0.91537831	1
Q9NXG2	THUMPD1	8	51.8	70.9	62	148.2	129.1	138	0.35	0.55	0.45	0.45	0.00059581	1
Q9BV44	THUMPD3	4	113	103	118.5	87	97	81.5	1.30	1.06	1.45	1.27	0.02307029	1
P04183	TK1	10	148.3	77.5	141	51.7	122.5	59	2.87	0.63	2.39	1.96	0.23392119	1
Q07157	TJP1	46	109.3	100.4	111.2	90.7	99.6	88.8	1.21	1.01	1.25	1.16	0.04156662	1
Q9H0E2	TOLLIP	7	65.9	67	52.2	134.1	133	147.8	0.49	0.50	0.35	0.45	0.00034033	1
Q6ZVM7	TOM1L2	7	145.8	48.8	111.3	54.2	151.2	88.7	2.69	0.32	1.25	1.42	0.92666527	1
O14656	TOR1A	9	110.7	111.3	121.3	89.3	88.7	78.7	1.24	1.25	1.54	1.35	0.00403396	1
Q5JTV8	TOR1AIP1	8	55	140	110.5	145	60	89.5	0.38	2.33	1.23	1.32	0.92213496	1
Q8NFQ8	TOR1AIP2	5	142.9	122.9	111	57.1	77.1	89	2.50	1.59	1.25	1.78	0.01769223	1
O14657	TOR1B	2	133.2	114.4	112.6	66.8	85.6	87.4	1.99	1.34	1.29	1.54	0.01256449	1
Q96S44	TP53RK	5	99.5	123	104.2	100.5	77	95.8	0.99	1.60	1.09	1.23	0.15444723	1
P37837	TALDO1	28	38.5	56.4	37	161.5	143.6	163	0.24	0.39	0.23	0.29	0.00022031	1
P23193	TCEA1	18	144.9	122.2	134.2	55.1	77.8	65.8	2.63	1.57	2.04	2.08	0.00188855	1
Q7KZ85	SUPT6H	31	114	99.6	106.7	86	100.4	93.3	1.33	0.99	1.14	1.15	0.08275745	1
Q96K17	BTF3L4	4	153.9	130.9	162.9	46.1	69.1	37.1	3.34	1.89	4.39	3.21	0.00186489	1
P20290	BTF3	13	125.1	123.7	132.4	74.9	76.3	67.6	1.67	1.62	1.96	1.75	0.00014312	1
O15164	TRIM24	4	127.8	116.6	101.2	72.2	83.4	98.8	1.77	1.40	1.02	1.40	0.04942008	1
Q13263	TRIM28	30	122.1	116.9	127.1	77.9	83.1	72.9	1.57	1.41	1.74	1.57	0.00045133	1
P46937	YAP1	8	159.8	159.1	138.5	40.2	40.9	61.5	3.98	3.89	2.25	3.37	0.00044493	1
P49711	CTCF	4	119.5	105.6	113.9	80.5	94.4	86.1	1.48	1.12	1.32	1.31	0.01039204	1
Q9Y4P3	TBL2	2	88.4	113.8	118.4	111.6	86.2	81.6	0.79	1.32	1.45	1.19	0.35665127	1
P02786	TFRC	24	102	75.5	93.9	98	124.5	106.1	1.04	0.61	0.89	0.84	0.16061883	1
Q13595	TRA2A	3	81.2	112.3	135.1	118.8	87.7	64.9	0.68	1.28	2.08	1.35	0.43676949	1
P62995	TRA2B	8	172.1	116	161	27.9	84	39	6.17	1.38	4.13	3.89	0.01487903	1
O95359	TACC2	3	72.6	59.9	104.2	127.4	140.1	95.8	0.57	0.43	1.09	0.70	0.08614028	1
P61586	RHOA	5	114.1	97	112.7	85.9	103	87.3	1.33	0.94	1.29	1.19	0.11009608	1
P37802	TAGLN2	18	73.5	62.4	72.9	126.5	137.6	127.1	0.58	0.45	0.57	0.54	0.00028297	1
O43493	TGOLN2	9	128.7	118.2	125.5	71.3	81.8	74.5	1.81	1.44	1.68	1.64	0.00039035	1
Q14232	EIF2B1	17	96.9	116	102.9	103.1	84	97.1	0.94	1.38	1.06	1.13	0.25707087	1

Q9Y2S6	TMA7	4	141.4	140.6	112.7	58.6	59.4	87.3	2.41	2.37	1.29	2.02	0.0090977	1
Q9BSH4	TACO1	10	96.1	110.6	87.1	103.9	89.4	112.9	0.92	1.24	0.77	0.98	0.6913918	1
P13693	TPT1	8	126.9	162.8	125.8	73.1	37.2	74.2	1.74	4.38	1.70	2.60	0.01099219	1
Q15631	TSN	14	120.5	108.5	122.1	79.5	91.5	77.9	1.52	1.19	1.57	1.42	0.00495035	1
P43307	SSR1	4	119.4	91.8	120.2	80.6	108.2	79.8	1.48	0.85	1.51	1.28	0.18804468	1
P51571	SSR4	5	124.4	124.5	151.6	75.6	75.5	48.4	1.65	1.65	3.13	2.14	0.00636233	1
Q9UNL2	SSR3	2	118.7	112.6	136.6	81.3	87.4	63.4	1.46	1.29	2.15	1.63	0.01129533	1
P30408	TM4SF1	1	89	78	68.8	111	122	131.2	0.80	0.64	0.52	0.66	0.006592	1
O15321	TM9SF1	4	83.1	67.8	78.5	116.9	132.2	121.5	0.71	0.51	0.65	0.62	0.00183119	1
Q99805	TM9SF2	9	99.9	95.6	98.7	100.1	104.4	101.3	1.00	0.92	0.97	0.96	0.09972606	1
Q9HD45	TM9SF3	9	103.6	90.1	102.9	96.4	109.9	97.1	1.07	0.82	1.06	0.98	0.73340884	1
Q92544	TM9SF4	9	103.2	104.7	110.4	96.8	95.3	89.6	1.07	1.10	1.23	1.13	0.01705361	1
Q71RG4	TMUB2	3	17.1	26.2	84.8	182.9	173.8	115.2	0.09	0.15	0.74	0.33	0.01877852	1
Q13445	TMED1	2	109.7	91.2	111.3	90.3	108.8	88.7	1.21	0.84	1.25	1.10	0.42296384	1
P49755	TMED10	11	91.4	85.4	93.6	108.6	114.6	106.4	0.84	0.75	0.88	0.82	0.00469844	1
Q7Z7H5	TMED4	7	101.3	114.3	98.6	98.7	85.7	101.4	1.03	1.33	0.97	1.11	0.2393573	1
Q9Y3B3	TMED7	8	93.7	45	97.2	106.3	155	102.8	0.88	0.29	0.95	0.71	0.14732905	1
Q9BVK6	TMED9	6	89.4	93.8	75.4	110.6	106.2	124.6	0.81	0.88	0.61	0.77	0.02449227	1
Q9BVC6	TMEM109	5	74.6	1.9	42.3	125.4	198.1	157.7	0.59	0.01	0.27	0.29	0.01532682	1
Q9H061	TMEM126	2	97.6	88.3	88.2	102.4	111.7	111.8	0.95	0.79	0.79	0.84	0.0172873	1
Q9HC07	TMEM165	3	115.7	114.3	119.7	84.3	85.7	80.3	1.37	1.33	1.49	1.40	0.00013223	1
Q7Z7N9	TMEM179	2	45.1	76.7	135.9	154.9	123.3	64.1	0.29	0.62	2.12	1.01	0.4953408	1
Q6NUQ4	TMEM214	9	76.6	70.7	81.9	123.4	129.3	118.1	0.62	0.55	0.69	0.62	0.00049781	1
Q9H0R3	TMEM222	3	87.4	68.8	82.2	112.6	131.2	117.8	0.78	0.52	0.70	0.67	0.00633558	1
Q96A57	TMEM230	3	75.5	60	62.2	124.5	140	137.8	0.61	0.43	0.45	0.50	0.00057079	1
P61165	TMEM258	1	120	114.3	129.2	80	85.7	70.8	1.50	1.33	1.82	1.55	0.00231815	1
Q96GE9	TMEM261	1	133	120.6	126.9	67	79.4	73.1	1.99	1.52	1.74	1.75	0.00044819	1
Q9BTV4	TMEM43	8	137.9	107.9	117.2	62.1	92.1	82.8	2.22	1.17	1.42	1.60	0.02857897	1
Q9BUB7	TMEM70	4	118.1	125.3	99.1	81.9	74.7	100.9	1.44	1.68	0.98	1.37	0.06239416	1
Q92973	TNPO1	22	115.4	111.8	110.1	84.6	88.2	89.9	1.36	1.27	1.22	1.29	0.00035514	1
Q9Y5L0	TNPO3	8	124.3	95.4	111.8	75.7	104.6	88.2	1.64	0.91	1.27	1.27	0.15064205	1
Q13428	TCOF1	30	132.9	146.1	152.1	67.1	53.9	47.9	1.98	2.71	3.18	2.62	0.00040254	1
P53007	SLC25A1	9	85.1	104.5	78.5	114.9	95.5	121.5	0.74	1.09	0.65	0.83	0.1262184	1
P40939	HADHA	33	100.1	95.1	99.8	99.9	104.9	100.2	1.00	0.91	1.00	0.97	0.2191382	1
P55084	HADHB	20	122.8	85.2	100.9	77.2	114.8	99.1	1.59	0.74	1.02	1.12	0.71997463	1
Q9NVV0	TMEM38E	2	113.8	105.5	119	86.2	94.5	81	1.32	1.12	1.47	1.30	0.01008644	1
P60174	TPI1	26	108.7	83.3	105.1	91.3	116.7	94.9	1.19	0.71	1.11	1.00	0.8715801	1
Q96LD4	TRIM47	9	47.6	63	66.4	152.4	137	133.6	0.31	0.46	0.50	0.42	0.00055662	1
O14773	TPP1	4	29.2	93.7	88.4	170.8	106.3	111.6	0.17	0.88	0.79	0.61	0.1131643	1
Q7Z2T5	TRMT1L	1	102.4	75.9	83.2	97.6	124.1	116.8	1.05	0.61	0.71	0.79	0.08325598	1
Q96FX7	TRMT61A	9	129.9	111.3	140.4	70.1	88.7	59.6	1.85	1.25	2.36	1.82	0.01064934	1

Q08J23	NSUN2	22	119.4	111.7	123.9	80.6	88.3	76.1	1.48	1.27	1.63	1.46	0.00189293	1
Q9UBP6	METTL1	9	107.5	95.9	87.9	92.5	104.1	112.1	1.16	0.92	0.78	0.96	0.51090517	1
Q9BSV6	TSEN34	4	83.1	85.8	91.6	116.9	114.2	108.4	0.71	0.75	0.85	0.77	0.00175557	1
Q13641	TPBG	4	107	93.2	87.8	93	106.8	112.2	1.15	0.87	0.78	0.94	0.37841462	1
Q9NYL9	TMOD3	8	40.1	92	51.1	159.9	108	148.9	0.25	0.85	0.34	0.48	0.02518719	1
P09493	TPM1	5	62.1	45.1	62.1	137.9	154.9	137.9	0.45	0.29	0.45	0.40	0.00040615	1
P06753	TPM3	9	118.7	88.6	121.1	81.3	111.4	78.9	1.46	0.80	1.53	1.26	0.26962245	1
P67936	TPM4	17	102.9	62.3	107.8	97.1	137.7	92.2	1.06	0.45	1.17	0.89	0.42726154	1
P07951	TPM2	2	57	136.3	126.9	143	63.7	73.1	0.40	2.14	1.74	1.42	0.72279604	1
Q71U36	TUBA1A	3	117.4	129.9	127.9	82.6	70.1	72.1	1.42	1.85	1.77	1.68	0.00079367	1
Q9BQE3	TUBA1C	3	133	117.7	129.4	67	82.3	70.6	1.99	1.43	1.83	1.75	0.00121873	1
P68366	TUBA4A	8	118.4	125.5	119.7	81.6	74.5	80.3	1.45	1.68	1.49	1.54	0.0001627	1
P07437	TUBB	6	131.4	124.2	122.6	68.6	75.8	77.4	1.92	1.64	1.58	1.71	0.00016821	1
Q13885	TUBB2A	2	163.7	114.9	141.6	36.3	85.1	58.4	4.51	1.35	2.42	2.76	0.01591309	1
Q13509	TUBB3	14	107.1	100.6	107.5	92.9	99.4	92.5	1.15	1.01	1.16	1.11	0.03277297	1
Q99426	TBCB	17	125	109.6	134.9	75	90.4	65.1	1.67	1.21	2.07	1.65	0.01123831	1
O75347	TBCA	9	135.5	130.8	151.4	64.5	69.2	48.6	2.10	1.89	3.12	2.37	0.00088008	1
Q9BTW9	TBCD	6	93	146	110.5	107	54	89.5	0.87	2.70	1.23	1.60	0.20882402	1
Q14166	TTLL12	13	137.8	125.1	134.3	62.2	74.9	65.7	2.22	1.67	2.04	1.98	0.00026772	1
Q9Y2W6	TDRKH	3	119.3	80.5	104	80.7	119.5	96	1.48	0.67	1.08	1.08	0.88155489	1
Q8NHU6	TDRD7	3	126.2	113	105.6	73.8	87	94.4	1.71	1.30	1.12	1.38	0.02477582	1
P55327	TPD52	12	97.8	80.7	100.5	102.2	119.3	99.5	0.96	0.68	1.01	0.88	0.18552534	1
O43399	TPD52L2	11	124.6	84.2	113.5	75.4	115.8	86.5	1.65	0.73	1.31	1.23	0.43225017	1
Q6IBS0	TWF2	5	59.4	176.2	152.5	140.6	23.8	47.5	0.42	7.40	3.21	3.68	0.3087267	1
Q6RW13	AGTRAP	2	158.4	129.3	144.8	41.6	70.7	55.2	3.81	1.83	2.62	2.75	0.00175166	1
Q06124	PTPN11	19	58	105.5	117.8	142	94.5	82.2	0.41	1.12	1.43	0.99	0.65401981	1
Q05209	PTPN12	7	97	71.7	88.9	103	128.3	111.1	0.94	0.56	0.80	0.77	0.05525448	1
Q9H3S7	PTPN23	6	63	6.9	149.5	137	193.1	50.5	0.46	0.04	2.96	1.15	0.41145551	1
P54577	YARS	36	175.3	141.8	152.2	24.7	58.2	47.8	7.10	2.44	3.18	4.24	0.00128566	1
P09012	SNRPA	8	93.1	90.2	124.4	106.9	109.8	75.6	0.87	0.82	1.65	1.11	0.75688744	1
P09234	SNRPC	4	134	104.8	91.7	66	95.2	108.3	2.03	1.10	0.85	1.33	0.31421644	1
P09661	SNRPA1	12	98.7	81.9	105.1	101.3	118.1	94.9	0.97	0.69	1.11	0.93	0.38501679	1
P08579	SNRPB2	8	88.7	82.8	127.4	111.3	117.2	72.6	0.80	0.71	1.75	1.09	0.97220377	1
O15042	U2SURP	24	144.2	130.1	144.2	55.8	69.9	55.8	2.58	1.86	2.58	2.34	0.00028699	1
Q53GS9	USP39	5	112.4	123.1	177.6	87.6	76.9	22.4	1.28	1.60	7.93	3.60	0.05751981	1
O75643	SNRNP20(	59	150.4	133.6	149.4	49.6	66.4	50.6	3.03	2.01	2.95	2.67	0.00032011	1
Q96DI7	SNRNP40	8	136.3	98.4	124.1	63.7	101.6	75.9	2.14	0.97	1.64	1.58	0.06809601	1
Q9Y333	LSM2	3	141.1	160.9	162.9	58.9	39.1	37.1	2.40	4.12	4.39	3.63	0.00036528	1
Q9Y4Z0	LSM4	3	153	128.5	137.6	47	71.5	62.4	3.26	1.80	2.21	2.42	0.00142106	1
P62312	LSM6	4	147.5	176.5	109.5	52.5	23.5	90.5	2.81	7.51	1.21	3.84	0.03155253	1
Q9NRR5	UBQLN4	3	113.7	127.6	139.2	86.3	72.4	60.8	1.32	1.76	2.29	1.79	0.00675304	1

O75208	COQ9	5	57.9	85.6	83	142.1	114.4	117	0.41	0.75	0.71	0.62	0.01720332	1
P51784	USP11	4	115.2	103.1	114.5	84.8	96.9	85.5	1.36	1.06	1.34	1.25	0.01692395	1
Q9UPU5	USP24	9	46.2	136.5	155.8	153.8	63.5	44.2	0.30	2.15	3.52	1.99	0.61956685	1
Q96RU2	USP28	5	153.7	177.8	105.8	46.3	22.2	94.2	3.32	8.01	1.12	4.15	0.03770188	1
Q13107	USP4	6	173	51.2	163.1	27	148.8	36.9	6.41	0.34	4.42	3.72	0.35144316	1
Q96K76	USP47	18	98.3	92.1	112.6	101.7	107.9	87.4	0.97	0.85	1.29	1.04	0.82720663	1
Q93009	USP7	29	105.1	98.4	109	94.9	101.6	91	1.11	0.97	1.20	1.09	0.12968606	1
P40818	USP8	13	104.8	98.7	112.3	95.2	101.3	87.7	1.10	0.97	1.28	1.12	0.13117206	1
P09936	UCHL1	13	110	83.1	109.5	90	116.9	90.5	1.22	0.71	1.21	1.05	0.8969428	1
P15374	UCHL3	4	90.9	119.3	91.3	109.1	80.7	108.7	0.83	1.48	0.84	1.05	0.94365256	1
Q9Y5K5	UCHL5	15	66.8	126.8	67.1	133.2	73.2	132.9	0.50	1.73	0.50	0.91	0.40564826	1
Q96FW1	OTUB1	12	105.4	129.8	114.5	94.6	70.2	85.5	1.11	1.85	1.34	1.43	0.03018733	1
Q5T6F2	UBAP2	10	124.5	132.4	142.5	75.5	67.6	57.5	1.65	1.96	2.48	2.03	0.0008455	1
O00762	UBE2C	6	172.3	154.6	118.9	27.7	45.4	81.1	6.22	3.41	1.47	3.70	0.01190729	1
P61077	UBE2D3	2	124.2	161.1	128	75.8	38.9	72	1.64	4.14	1.78	2.52	0.01035526	1
P61088	UBE2N	7	124	127.7	112.8	76	72.3	87.2	1.63	1.77	1.29	1.56	0.00245915	1
Q712K3	UBE2R2	5	146.5	128.9	130	53.5	71.1	70	2.74	1.81	1.86	2.14	0.00094898	1
Q92575	UBXN4	5	112.1	104.5	112.8	87.9	95.5	87.2	1.28	1.09	1.29	1.22	0.00645046	1
O94888	UBXN7	7	159.5	108.1	152.7	40.5	91.9	47.3	3.94	1.18	3.23	2.78	0.02449434	1
Q14376	GALE	17	60.3	78.5	57.2	139.7	121.5	142.8	0.43	0.65	0.40	0.49	0.00179781	1
Q9NYU2	UGGT1	49	111.2	107.3	110.1	88.8	92.7	89.9	1.25	1.16	1.22	1.21	0.00031418	1
Q16222	UAP1	29	120.7	189.6	190.3	79.3	10.4	9.7	1.52	18.23	19.62	13.12	0.01489603	1
P30085	CMPK1	14	101.6	98	104.4	98.4	102	95.6	1.03	0.96	1.09	1.03	0.3662538	1
Q9BQ61	C19orf43	6	131.3	107.8	129.5	68.7	92.2	70.5	1.91	1.17	1.84	1.64	0.01282192	1
O43795	MYO1B	4	76.5	111.8	78.9	123.5	88.2	121.1	0.62	1.27	0.65	0.85	0.24607888	1
O00159	MYO1C	11	137.7	146.4	154.7	62.3	53.6	45.3	2.21	2.73	3.42	2.79	0.00018302	1
Q12965	MYO1E	7	88.9	73.9	72.1	111.1	126.1	127.9	0.80	0.59	0.56	0.65	0.00449931	1
Q9HB07	C12orf10	9	138.5	114.1	128.1	61.5	85.9	71.9	2.25	1.33	1.78	1.79	0.00576311	1
Q5T6V5	C9orf64	11	102.5	121.5	115.3	97.5	78.5	84.7	1.05	1.55	1.36	1.32	0.02960534	1
Q9Y224	C14orf166	18	93.4	91.9	87.4	106.6	108.1	112.6	0.88	0.85	0.78	0.83	0.00203662	1
A6NDU8	C5orf51	5	96.4	95.8	91.7	103.6	104.2	108.3	0.93	0.92	0.85	0.90	0.00679587	1
Q8IYL3	C1orf174	2	162.5	117.9	128	37.5	82.1	72	4.33	1.44	1.78	2.52	0.01935759	1
O95848	NUDT14	2	100	74.7	86	100	125.3	114	1.00	0.60	0.75	0.78	0.06453929	1
Q16831	UPP1	12	140.2	101.5	143.6	59.8	98.5	56.4	2.34	1.03	2.55	1.97	0.0408175	1
Q9BZX2	UCK2	5	109.4	150.5	143.6	90.6	49.5	56.4	1.21	3.04	2.55	2.26	0.01846555	1
Q9BZF9	UACA	7	90.5	105.3	95	109.5	94.7	105	0.83	1.11	0.90	0.95	0.37818207	1
Q3ZAQ7	VMA21	4	137.1	150.4	124.9	62.9	49.6	75.1	2.18	3.03	1.66	2.29	0.00197652	1
O75436	VPS26A	9	101.7	136.8	108.1	98.3	63.2	91.9	1.03	2.16	1.18	1.46	0.11152416	1
Q9UK41	VPS28	6	117.7	109.2	126	82.3	90.8	74	1.43	1.20	1.70	1.45	0.00678253	1
Q9UBQ0	VPS29	4	112.3	85.3	113.3	87.7	114.7	86.7	1.28	0.74	1.31	1.11	0.60519159	1
Q96QK1	VPS35	21	121.6	115.3	117.1	78.4	84.7	82.9	1.55	1.36	1.41	1.44	0.00016986	1

Q5VIR6	VPS53	3	123.6	115.1	114.7	76.4	84.9	85.3	1.62	1.36	1.34	1.44	0.00097239	1
Q9NP79	VTA1	7	155.1	145	135.5	44.9	55	64.5	3.45	2.64	2.10	2.73	0.00035004	1
Q9BRG1	VPS25	3	113.3	81.5	142.1	86.7	118.5	57.9	1.31	0.69	2.45	1.48	0.37650995	1
Q96GC9	VMP1	1	94.9	85.4	97.3	105.1	114.6	102.7	0.90	0.75	0.95	0.87	0.04384144	1
Q86WA6	BPHL	3	90	130.3	118.1	110	69.7	81.9	0.82	1.87	1.44	1.38	0.20383055	1
P50552	VASP	23	124.3	159.6	162.4	75.7	40.4	37.6	1.64	3.95	4.32	3.30	0.00491122	1
P49748	ACADVL	27	105.2	98.2	112.8	94.8	101.8	87.2	1.11	0.96	1.29	1.12	0.14430525	1
Q9P035	HACD3	6	81.8	96.8	101.1	118.2	103.2	98.9	0.69	0.94	1.02	0.88	0.17721196	1
Q53GQ0	HSD17B12	14	52.9	98.8	55	147.1	101.2	145	0.36	0.98	0.38	0.57	0.04240911	1
Q9NZ01	TECR	5	113.4	91.7	104.6	86.6	108.3	95.4	1.31	0.85	1.10	1.08	0.50823355	1
Q9Y3E0	GOLT1B	4	128.2	121.4	116.6	71.8	78.6	83.4	1.79	1.54	1.40	1.58	0.00075204	1
Q9UEU0	VTI1B	5	100	78.4	76.2	100	121.6	123.8	1.00	0.64	0.62	0.75	0.04790026	1
P63027	VAMP2	2	122	123.8	112.8	78	76.2	87.2	1.56	1.62	1.29	1.49	0.0012573	1
Q15836	VAMP3	2	131.2	118.2	105.4	68.8	81.8	94.6	1.91	1.44	1.11	1.49	0.02561873	1
Q9P0L0	VAPA	14	117.4	87.1	131.1	82.6	112.9	68.9	1.42	0.77	1.90	1.37	0.26627236	1
P46459	NSF	14	109.9	104.2	112.7	90.1	95.8	87.3	1.22	1.09	1.29	1.20	0.00722266	1
O75396	SEC22B	12	125.2	110	121.4	74.8	90	78.6	1.67	1.22	1.54	1.48	0.00428002	1
Q12907	LMAN2	19	122.5	97.9	131	77.5	102.1	69	1.58	0.96	1.90	1.48	0.07110216	1
Q00341	HDLBP	53	102.5	95.4	101.7	97.5	104.6	98.3	1.05	0.91	1.03	1.00	0.93710525	1
P08670	VIM	34	99.3	150.8	182.9	100.7	49.2	17.1	0.99	3.07	10.70	4.92	0.06165628	1
Q9H0V9	LMAN2L	9	104.1	90.3	92.8	95.9	109.7	107.2	1.09	0.82	0.87	0.92	0.22825499	1
Q9BQB6	VKORC1	1	100.6	103.4	116	99.4	96.6	84	1.01	1.07	1.38	1.15	0.11736133	1
Q8N0U8	VKORC1L	2	21.1	97.9	96	178.9	102.1	104	0.12	0.96	0.92	0.67	0.18826931	1
P21796	VDAC1	20	84.8	60	87.7	115.2	140	112.3	0.74	0.43	0.78	0.65	0.02235683	1
P45880	VDAC2	15	111.9	74.7	117.6	88.1	125.3	82.4	1.27	0.60	1.43	1.10	0.89010058	1
Q9Y277	VDAC3	13	79.3	54.5	85.1	120.7	145.5	114.9	0.66	0.37	0.74	0.59	0.01517186	1
P54289	CACNA2D	9	64	54.1	74.2	136	145.9	125.8	0.47	0.37	0.59	0.48	0.00094036	1
P27449	ATP6V0C	1	100.9	99.1	71	99.1	100.9	129	1.02	0.98	0.55	0.85	0.23075518	1
P38606	ATP6V1A	25	109.4	97.5	108.9	90.6	102.5	91.1	1.21	0.95	1.20	1.12	0.12776107	1
P36543	ATP6V1E1	12	96.4	95	126.4	103.6	105	73.6	0.93	0.90	1.72	1.18	0.45860771	1
O75348	ATP6V1G1	2	98.8	134.1	76.2	101.2	65.9	123.8	0.98	2.03	0.62	1.21	0.81157118	1
Q9Y4E1	WASHC2C	10	45.4	73.3	86.5	154.6	126.7	113.5	0.29	0.58	0.76	0.54	0.02104112	1
Q9Y3C0	WASHC3	1	68.4	91.6	89.7	131.6	108.4	110.3	0.52	0.85	0.81	0.73	0.03326799	1
Q12768	WASHC5	3	124.3	111.8	90.5	75.7	88.2	109.5	1.64	1.27	0.83	1.25	0.2726594	1
Q5JSH3	WDR44	8	139	144.8	159.2	61	55.2	40.8	2.28	2.62	3.90	2.93	0.00035859	1
Q9GZS3	WDR61	10	73	104.3	91.2	127	95.7	108.8	0.57	1.09	0.84	0.83	0.1771377	1
Q6UXN9	WDR82	8	92.2	158.9	102.2	107.8	41.1	97.8	0.86	3.87	1.04	1.92	0.29295551	1
Q9Y2W2	WBP11	10	129.7	151	142.2	70.3	49	57.8	1.84	3.08	2.46	2.46	0.00072107	1
Q969T9	WBP2	8	86.9	91.7	105.6	113.1	108.3	94.4	0.77	0.85	1.12	0.91	0.25479276	1
Q9BTA9	WAC	1	103.4	124.5	84.6	96.6	75.5	115.4	1.07	1.65	0.73	1.15	0.63606716	1
P12955	PEPD	19	124.1	106.3	115.5	75.9	93.7	84.5	1.64	1.13	1.37	1.38	0.01358483	1

P13010	XRCC5	32	125.4	116.7	124.9	74.6	83.3	75.1	1.68	1.40	1.66	1.58	0.00036204	1
P12956	XRCC6	32	128.5	120.8	129.3	71.5	79.2	70.7	1.80	1.53	1.83	1.72	0.00016571	1
Q86U90	YRDC	5	154.9	148.1	184.7	45.1	51.9	15.3	3.43	2.85	12.07	6.12	0.00140723	1
Q9Y5A9	YTHDF2	5	96.1	136.7	149.9	103.9	63.3	50.1	0.92	2.16	2.99	2.03	0.07369473	1
Q7Z739	YTHDF3	7	130.1	162.2	129.5	69.9	37.8	70.5	1.86	4.29	1.84	2.66	0.00602295	1
Q5T200	ZC3H13	5	189.1	178.1	149.3	10.9	21.9	50.7	17.35	8.13	2.94	9.48	0.0010042	1
Q8WU90	ZC3H15	5	124.7	129.3	120.3	75.3	70.7	79.7	1.66	1.83	1.51	1.66	0.00017523	1
Q9UPT8	ZC3H4	12	105.7	106	111.3	94.3	94	88.7	1.12	1.13	1.25	1.17	0.00397525	1
Q7Z2W4	ZC3HAV1	18	123	108.7	106.9	77	91.3	93.1	1.60	1.19	1.15	1.31	0.02332103	1
Q6NZY4	ZCCHC8	4	172.9	145	163	27.1	55	37	6.38	2.64	4.41	4.47	0.00047517	1
Q14966	ZNF638	7	101	171.8	85.6	99	28.2	114.4	1.02	6.09	0.75	2.62	0.35820921	1
Q8NEW0	SLC30A7	1	132.1	132.4	176.8	67.9	67.6	23.2	1.95	1.96	7.62	3.84	0.0109443	1

Supplementary Table. S2 Differentially expressed proteins regulated by MEST overexpression

Uniprot IDs	Gene name	Protein name	Fold Change
O00291	HIP1	Huntingtin-interacting protein 1	35.54
Q92736	RYR2	Ryanodine receptor 2	6.03
P09913	IFIT2	Interferon-induced protein with tetratricopeptide repeats 2	4.35
O14879	IFIT3	Interferon-induced protein with tetratricopeptide repeats 3	3.66
Q96BM9	ARL8A	ADP-ribosylation factor-like protein 8A	3.65
Q7Z7A1	CNTRL	Centriolin	3.57
P20591	MX1	Interferon-induced GTP-binding protein Mx1	3.52
P09914	IFIT1	Interferon-induced protein with tetratricopeptide repeats 1	3.49
Q9C002	NMES1	Normal mucosa of esophagus-specific gene 1 protein	3.45
Q96JH7	VCPIP1	Deubiquitinating protein VCIP135	3.37
P55957	BID	BH3-interacting domain death agonist	3.30
P49750	YLPM1	YLP motif-containing protein 1	2.85
P13164	IFITM1	Interferon-induced transmembrane protein 1	2.63
Q9Y586	MAB21L2	Protein mab-21-like 2	2.56
P09417	QDPR	Dihydropteridine reductase	2.54
A4D1E9	GTPBP10	GTP-binding protein 10	2.44
Q96E11	MRRF	Ribosome-recycling factor, mitochondrial	2.44
Q9H0P0	NT5C3A	Cytosolic 5	2.12
Q8WXH0	SYNE2	Nesprin-2	2.09
P05161	ISG15	Ubiquitin-like protein ISG15	2.03
Q9H8S9	MOB1A	MOB kinase activator 1A	1.99
P29992	GNA11	Guanine nucleotide-binding protein subunit alpha-11	1.92
P16188	HLA-A	HLA class I histocompatibility antigen, A-30 alpha chain	1.91
Q96AZ6	ISG20	Interferon-stimulated gene 20 kDa protein	1.90
P23381	WARS	Tryptophan--tRNA ligase, cytoplasmic	1.88
Q9H7L9	SUDS3	Sin3 histone deacetylase corepressor complex component SDS3	1.86
Q03519	TAP2	Antigen peptide transporter 2	1.85
O95786	DDX58	Probable ATP-dependent RNA helicase DDX58	1.74
Q14146	URB2	Unhealthy ribosome biogenesis protein 2 homolog	1.71
P04179	SOD2	Superoxide dismutase [Mn], mitochondrial	1.71
P42224	STAT1	Signal transducer and activator of transcription 1-alpha/beta	1.71
Q9BQE5	APOL2	Apolipoprotein L2	1.68
P30453	HLA-A	HLA class I histocompatibility antigen, A-34 alpha chain	1.67
Q63HN8	RNF213	E3 ubiquitin-protein ligase RNF213	1.65
P28838	LAP3	Cytosol aminopeptidase	1.63
Q9Y6K5	OAS3	2'-5'-oligoadenylate synthase 3	1.60
P28907	CD38	ADP-ribosyl cyclase/cyclic ADP-ribose hydrolase 1	1.59
Q92620	DHX38	Pre-mRNA-splicing factor ATP-dependent RNA helicase PRP16	1.56
Q9BYX4	IFIH1	Interferon-induced helicase C domain-containing protein 1	1.55
P35354	PTGS2	Prostaglandin G/H synthase 2	1.55
P19784	CSNK2A2	Casein kinase II subunit alpha	1.53
Q9UII4	HERC5	E3 ISG15--protein ligase HERC5	1.53
P02792	FTL	Ferritin light chain	1.53
Q03518	TAP1	Antigen peptide transporter 1	1.51
Q14669	TRIP12	E3 ubiquitin-protein ligase TRIP12	1.51
Q8TDB6	DTX3L	E3 ubiquitin-protein ligase DTX3L	1.49
Q70UQ0	IKBIP	Inhibitor of nuclear factor kappa-B kinase-interacting protein	1.49
O76071	CIAO1	Probable cytosolic iron-sulfur protein assembly protein CIAO1	1.48
Q9NZT2	OGFR	Opioid growth factor receptor	1.47
P62745	RHOB	Rho-related GTP-binding protein RhoB	1.47
P21980	TGM2	Protein-glutamine gamma-glutamyltransferase 2	1.47
Q9UMX5	NENF	Neudesin	1.47
O75608	LYPLA1	Acyl-protein thioesterase 1	1.47
P50453	SERPINB9	Serpin B9	1.47
P40305	IFI27	Interferon alpha-inducible protein 27, mitochondrial	1.46
Q8TAT6	NPLOC4	Nuclear protein localization protein 4 homolog	1.45
P48307	TFPI2	Tissue factor pathway inhibitor 2	1.45
P30508	HLA-C	HLA class I histocompatibility antigen, Cw-12 alpha chain	1.44
Q15646	OASL	2'-5'-oligoadenylate synthase-like protein	1.43
Q9H223	EHD4	EH domain-containing protein 4	1.43
Q16740	CLPP	ATP-dependent Clp protease proteolytic subunit, mitochondrial	1.41
P09543	CNP	2',3'-cyclic-nucleotide 3'-phosphodiesterase (CNP) (CNPase) (EC 3.1.4.37)	1.40
Q9Y3C6	PPIL1	Peptidyl-prolyl cis-trans isomerase-like 1	1.40

P05231	IL6	Interleukin-6	1.39
Q9NQW7	XPNPEP1	Xaa-Pro aminopeptidase 1	1.38
Q6FI81	CIAPIN1	Anamorsin	1.38
Q9NVI1	FANCI	Fanconi anemia group I protein	1.38
Q8TCS8	PNPT1	Polyribonucleotide nucleotidyltransferase 1, mitochondrial	1.38
Q01628	IFITM3	Interferon-induced transmembrane protein 3	1.37
P62256	UBE2H	Ubiquitin-conjugating enzyme E2 H	1.37
P00973	OAS1	2'-5'-oligoadenylate synthase 1	1.36
Q9BZL6	PRKD2	Serine/threonine-protein kinase D2	1.35
Q8WVX9	FAR1	Fatty acyl-CoA reductase 1	1.35
O60287	URB1	Nucleolar pre-ribosomal-associated protein 1	1.34
O15155	BET1	BET1 homolog	1.33
P62633	CNBP	Cellular nucleic acid-binding protein	1.33
P40763	STAT3	Signal transducer and activator of transcription 3	1.32
P26885	FKBP2	Peptidyl-prolyl cis-trans isomerase FKBP2	1.32
Q16352	INA	Alpha-internexin	1.32
Q15121	PEA15	Astrocytic phosphoprotein PEA-15	1.32
Q9NUQ6	SPATS2L	SPATS2-like protein	1.32
Q09028	RBBP4	Histone-binding protein RBBP4	1.31
P07305	H1F0	Histone H1.0	1.31
P19525	EIF2AK2	Interferon-induced, double-stranded RNA-activated protein kinase	1.31
Q9P2R3	ANKFY1	Rabankyrin-5	1.31
O95758	PTBP3	Polypyrimidine tract-binding protein 3	1.30
O75431	MTX2	Metaxin-2	0.77
Q9Y5X3	SNX5	Sorting nexin-5	0.77
P17612	PRKACA	cAMP-dependent protein kinase catalytic subunit alpha	0.77
Q5T6F2	UBAP2	Ubiquitin-associated protein 2	0.76
Q99595	TIMM17A	Mitochondrial import inner membrane translocase subunit Tim17-A	0.76
O43633	CHMP2A	Charged multivesicular body protein 2a	0.76
Q9UG63	ABCF2	ATP-binding cassette sub-family F member 2	0.75
Q969G6	RFK	Riboflavin kinase	0.75
P16949	STMN1	Stathmin	0.74
Q9NXP7	GIN1	Gypsy retrotransposon integrase-like protein 1	0.74
Q14498	RBM39	RNA-binding protein 39	0.74
Q9NWU5	MRPL22	39S ribosomal protein L22, mitochondrial	0.73
P15927	RPA2	Replication protein A 32 kDa subunit	0.73
Q86XA9	HEATR5A	HEAT repeat-containing protein 5A	0.72
Q6UX53	METTL7B	Methyltransferase-like protein 7B	0.70
Q3LXA3	TKFC	Triokinase/FMN cyclase	0.70
P83111	LACTB	Serine beta-lactamase-like protein LACTB, mitochondrial	0.70
P61011	SRP54	Signal recognition particle 54 kDa protein	0.70
Q13907	IDI1	Isopentenyl-diphosphate Delta-isomerase 1	0.70
P55263	ADK	Adenosine kinase	0.69
Q00535	CDK5	Cyclin-dependent-like kinase 5	0.68
Q9UBI6	GNG12	Guanine nucleotide-binding protein G(I)/G(S)/G(O) subunit gamma-12	0.67
Q5TFE4	NT5DC1	5'-nucleotidase domain-containing protein 1 (EC 3.1.3.-)	0.67
P31937	HIBADH	3-hydroxyisobutyrate dehydrogenase, mitochondrial	0.67
P28072	PSMB6	Proteasome subunit beta type-6	0.67
P55011	SLC12A2	Solute carrier family 12 member 2	0.66
Q8N4V1	MMGT1	Membrane magnesium transporter 1	0.65
Q9BW30	TPPP3	Tubulin polymerization-promoting protein family member 3	0.65
P61769	B2M	Beta-2-microglobulin	0.65
O00566	MPHOSPH10	U3 small nucleolar ribonucleoprotein protein MPP10	0.52
P27216	ANXA13	Annexin A13	0.52
Q8N3U4	STAG2	Cohesin subunit SA-2	0.51
Q8TE73	DNAH5	Dynein heavy chain 5, axonemal	0.48
Q9Y4I1	MYO5A	Unconventional myosin-Va	0.44
Q9UNS2	COPS3	COP9 signalosome complex subunit 3	0.23
Q96HU8	DIRAS2	GTP-binding protein Di-Ras2	0.22
O76041	NEBL	Nebulette	0.08
P52306	RAP1GDS1	Rap1 GTPase-GDP dissociation stimulator 1	0.08
Q6NUN7	C11orf63	Uncharacterized protein C11orf63	0.07
Q9ULC6	PADI1	Protein-arginine deiminase type-1	0.01
Q16512	PKN1	Serine/threonine-protein kinase N1	0.01

Supplementary Table. S3 Potential MEST binding proteins identified by co-immunoprecipitation MS.

Uniprot IDs	Gene symbol	Protein name	Mol. weight [kDa]	pI
P55072	VCP	Transitional endoplasmic reticulum ATPase	89.32	4.89
P05141	SLC25A5	ADP/ATP translocase 2	32.85	10.18
Q13151	HNRNPA0	Heterogeneous nuclear ribonucleoprotein A0	30.84	9.66
P62913	RPL11	60S ribosomal protein L11	20.25	10.14
Q9BRZ2	TRIM56	E3 ubiquitin-protein ligase TRIM56	81.49	7.78
Q9UBS4	DNAJB11	DnaJ homolog subfamily B member 11	40.51	6.07
P67809	YBX1	Nuclease-sensitive element-binding protein 1	35.92	10.31
Q00325	SLC25A3	Phosphate carrier protein, mitochondrial	40.09	9.79
Q13459	MYO9B	Unconventional myosin-Ixb	243.40	9.06
Q9Y2W1	THRAP3	Thyroid hormone receptor-associated protein 3	108.66	10.82
Q9Y2P8	RCL1	RNA 3'-terminal phosphate cyclase-like protein	40.84	9.67
Q9P035	PTPLAD1	Very-long-chain (3R)-3-hydroxyacyl-CoA dehydratase 3	43.16	9.25
Q8N3C0	ASCC3	Activating signal cointegrator 1 complex subunit 3	251.46	7.10
P55786	NPEPPS	Puromycin-sensitive aminopeptidase	103.28	5.44
P09661	SNRPA1	U2 small nuclear ribonucleoprotein A	28.42	9.00
O75688	PPM1B	Protein phosphatase 1B	52.64	4.69
P53007	SLC25A1	Tricarboxylate transport protein, mitochondrial	34.01	10.44
P35579	MYH9	Myosin-9	226.53	5.29
Q15149	PLEC	Plectin	531.79	5.76
Q14204	DYNC1H1	Cytoplasmic dynein 1 heavy chain 1	532.40	6.34
Q7Z3U7	MON2	Protein MON2 homolog	190.36	5.95
Q92945	KHSRP	Far upstream element-binding protein 2	73.12	7.34
Q13148	TARDBP	TAR DNA-binding protein 43	44.74	6.07
Q06265	EXOSC9	Exosome complex component RRP45	48.95	4.93
Q7Z2W4	ZC3HAV1	Zinc finger CCCH-type antiviral protein 1	101.43	8.47
P25205	MCM3	DNA replication licensing factor MCM3	90.98	5.48
Q9UII4	HERC5	E3 ISG15--protein ligase HERC5	116.85	7.70
Q96QR8	PURB	Transcriptional activator protein Pur-beta	33.24	5.06
P35580	MYH10	Myosin-10	229.00	5.22
Q9BUJ2	HNRNPUL1	Heterogeneous nuclear ribonucleoprotein U-like protein 1	95.74	6.90
Q9NXF1	TEX10	Testis-expressed sequence 10 protein	105.67	9.86
P48651	PTDSS1	Phosphatidylserine synthase 1	55.53	8.52
